# Supplementary material for: Synthesis of sialylated human milk oligosaccharides by automated glycan assembly
Source: Nat Commun. 2026 May 10;17:4214. doi: 10.1038/s41467-026-73028-w (PMC13157500; doi:10.1038/s41467-026-73028-w)
Supplement: Supplementary file 1 — Supplementary Information [file 41467_2026_73028_MOESM1_ESM.pdf]

Supplementary Information

Synthesis of Sialylated Human Oligosaccharides by Automated Glycan Assembly

Yan-Ting Kuo[1,2,3], Kim Le Mai Hoang[3*], and Peter H. Seeberger[1,2*]

[1]Department of Biomolecular Systems, Max Planck Institute of Colloids and Interfaces, 14476, Potsdam, Germany.

[2]Institute of Chemistry and Biochemistry, Freie Universität Berlin, 14195, Berlin, Germany.

[3]GlycoUniverse GmbH & Co. KGaA, 14476, Potsdam, Germany.

*Corresponding authors: Kim.Lemaihoang@glycouniverse.de & Peter.Seeberger@mpikg.mpg.de

Contents

1 General Information 1

2 Supplementary Figures 2

2.1 Optimization of leaving groups and temperatures for on-resin sialylation 2

2.2 Protecting groups on galactose affect α(2,6)-sialylation.................................... 3

2.3 Optimization of AGA programs for multi-cycle glycosylation 4

2.4 AGA Optimization of pentasaccharide **11** 5

2.5 Protecting groups on glucosamine affect α(2,6)-sialylation 6

2.6 AGA Optimization of protected DSLNF II **17** (Route A1 and A2) 7

2.7 AGA Optimization of protected DSLNF II **17** (Route B1).................................. 8

2.8 AGA Optimization of protected DSLNF II **17** (Route B2).................................. 9

2.9 Optimization of global deprotection for pentasaccharide **9** 10

3 Synthesis of Building Blocks 11

3.1 Sialic acid building blocks 11

3.2 Galactose Building Blocks.. 23

3.3 Glucosamine Building Blocks... 40

4 Materials and Conditions for Automated Synthesis.. 48

4.1 Materials and Measurements.. 48

4.2 Preparation of Stock Solutions.. 48

4.3 Modules for Automated Synthesis 50

4.4 Post-synthesizer Manipulation 52

5 Automated Glycan Assembly of Protected Sialylated Glycans 57

5.1 AGA of Disaccharide **4**.. 57

5.2 AGA of Disaccharide **5b** 61

5.3 AGA of Pentasaccharide **9**.. 64

5.4 AGA of Pentasaccharide **10** ... 69
5.5 AGA of Pentasaccharide **11** ... 73
5.6 AGA of Pentasaccharide **12** ... 78
5.7 AGA of Hexasaccharide **13** ... 82
5.8 AGA of Hexasaccharide **14** ... 87
5.9 AGA of Hexasaccharide **15** ... 92
5.10 AGA of Hexasaccharide **16** ... 97
5.11 AGA of Heptasaccharide **17** ... 102
5.12 AGA of Hexasaccharide **18** ... 107
5.13 AGA of Pentasaccharide **S2** ... 112
5.14 AGA of Pentasaccharide **S3** ... 116
6 Global Deprotection of Sialylated Glycans ... 121
6.1 Disaccharide **19** ... 121
6.2 Sialyllacto-*N*-neotetraose c (LSTc) **20** ... 125
6.3 Sialyllacto-*N*-neotetraose **21** ... 129
6.4 Sialyllacto-*N*-neotetraose **22** ... 133
6.5 Disaccharide **23** ... 137
6.6 Sialyllacto-N-neotetraose **24** ... 141
6.7 Disialyllacto-*N*-neotetraose (DSLNnT) **25** ... 145
6.8 Disialyllacto-*N*-tetraose (DSLNT) **26** ... 149
6.9 Sialyllacto-*N*-fucopentaose III (Sialyl LNFP III) **27** ... 153
6.10 Sialyllacto-*N*-fucopentaose III (Sialyl LNFP III) **28** ... 157
6.11 Fucosyldisialyllacto-N-tetraose (DSLNF II) **29** ... 161
7 References ... 165

1 General Information

All chemicals were reagent grade and used as supplied unless otherwise noted. All solvents for chemical reactions were commercially purchased in p.a. quality. If stated, residual water was first removed by passing through a solvent dispensing system (J.C. Meyer) equipped with adsorption columns and was further dried with 4 Å molecular sieves granules following the described protocol (**Section 4.2**). For HPLC and MS spectrometry, solvents with corresponding quality grades were used. Thioglycosyl donors were purchased from GlycoUniverse GmbH & Co. KGaA or synthesized if stated. All solutions were freshly prepared and kept under argon during the automation process. All reagent lines involved were washed and primed before dispensing.

Reaction progress and identity of all compounds were determined by analytical thin-layer chromatography (TLC) for synthesized building blocks and intermediates. TLC was performed on Merck silica gel 60 F254 plates (0.25 mm). Compounds were visualized by UV irradiation (254 nm) or stained (Hanessian's Stain: 235 mL of distilled water, 12 g of ammonium molybdate, 0.5 g of ceric ammonium molybdate, and 15 mL sulfuric acid). Flash column chromatography was performed on Kieselgel 60 with 230-400 mesh (Sigma-Aldrich, St. Louis, USA). ^{1}H, ^{13}C-NMR spectra were recorded on a Bruker Ascend-400 (400 MHz), Varian 600-MR (600 MHz) and Bruker Ascend-700 (700 MHz) spectrometer. Chemical shifts (δ) are reported in parts per million (ppm) relative to the respective residual solvent peaks ($CDCl_3$: δ 7.26 in ^{1}H and 77.00 in ^{13}C; CD_3OD: δ 3.31 in ^{1}H and 49.00 in ^{13}C; *d6*-DMSO: δ 2.50 in ^{1}H and 39.52 in ^{13}C; D_2O: δ 4.79 in ^{1}H). Bidimensional and non-decoupled experiments were performed to assign identities of peaks showing relevant structural features. The following abbreviations are used to indicate peak multiplicities: br. (broad), s (singlet), d (doublet), dd (doublet of doublets), ddd (doublet of doublet of doublets), t (triplet), dt (doublet of triplets), td (triplet of doublets), m (multiplet). Coupling constants (*J*) are reported in Hertz (Hz). NMR spectra were processed using MestreNova 14.1 (MestreLab Research). Assignments were supported by COSY, HSQC and HMBC experiments. High resolution mass spectra were obtained using a Xevo G2-XS QTof mass spectrometer (HRMS, Waters).

2 Supplementary Figures

2.1 Optimization of leaving groups and temperatures for on-resin sialylation

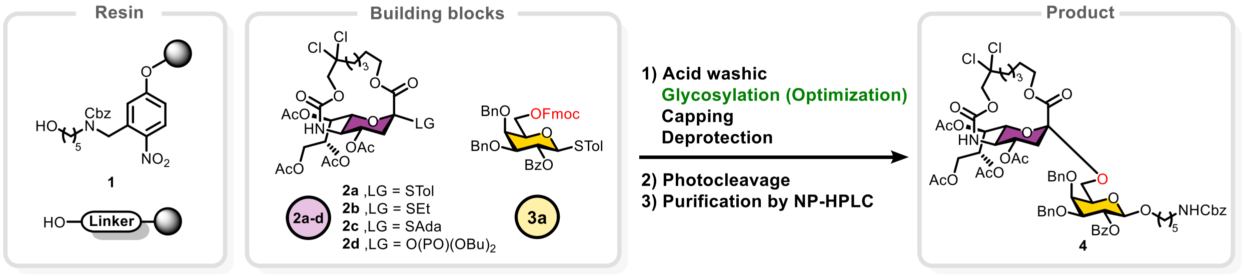

Entry	Sia	Temperature (min)	Yield
1	**2a** (10.0 eq. x1)	-40 °C (30 min) → -20 °C (10 min)	46%
2		-60 °C (180 min) → -40 °C (10 min)	33%
3	**2b** (10.0 eq. x1)	-40 °C (30 min) → -20 °C (10 min)	29%
4		-60 °C (180 min) → -40 °C (10 min)	27%
5	**2c** (10.0 eq. x1)	-40 °C (30 min) → -20 °C (10 min)	25%
6		-60 °C (180 min) → -40 °C (10 min)	34%
7	**2d** (10.0 eq. x1)	-40 °C (30 min) → -20 °C (10 min)	n.d.
8		-60 °C (60 min) → -40 °C (10 min)	n.d.

Supplementary Fig. 1 Optimization of α(2,6)-sialylation with sialic acid building blocks **2a-d** and temperatures on AGA platform. Sia: Sialic acid building blocks, n.d.: not detected.

2.2 Protecting groups on galactose affect α(2,6)-sialylation

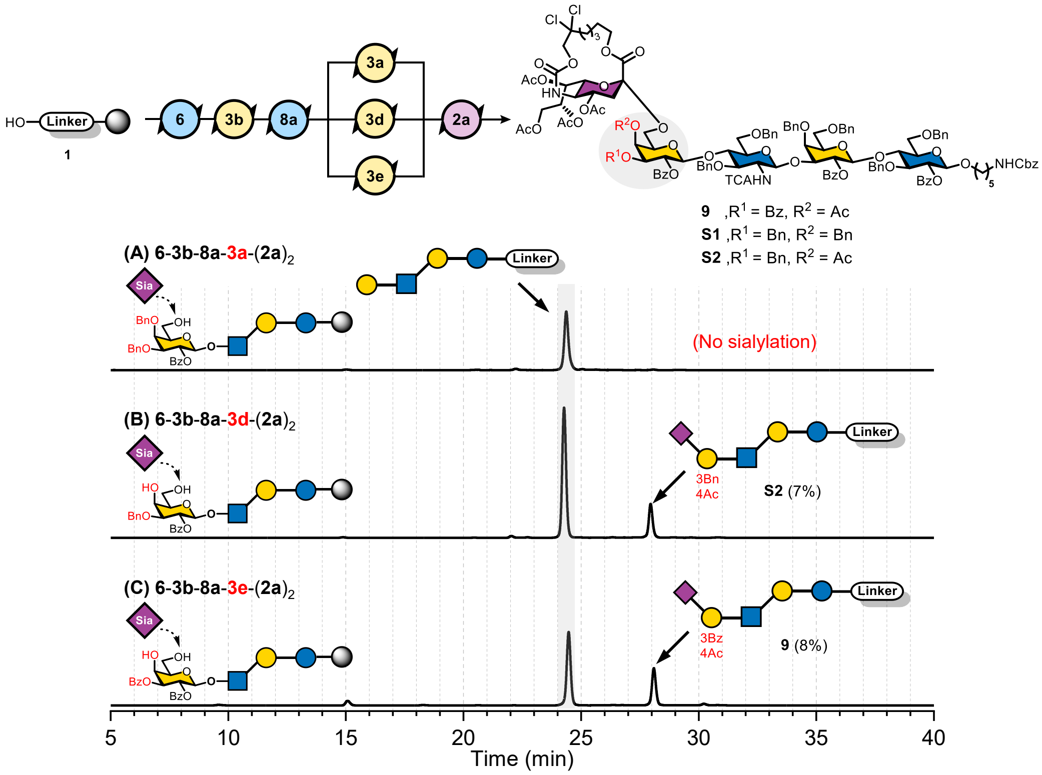

Supplementary Fig. 2 Protecting groups on galactose affect α(2,6)-sialylation. (A) Using building block **3a**; (B) Using building block **3d**; (C) Using building block **3e**; Isolated yields after NP-HPLC purification are shown in parentheses. Source data are provided as a Source Data file.

2.3 Optimization of AGA programs for multi-cycle glycosylation

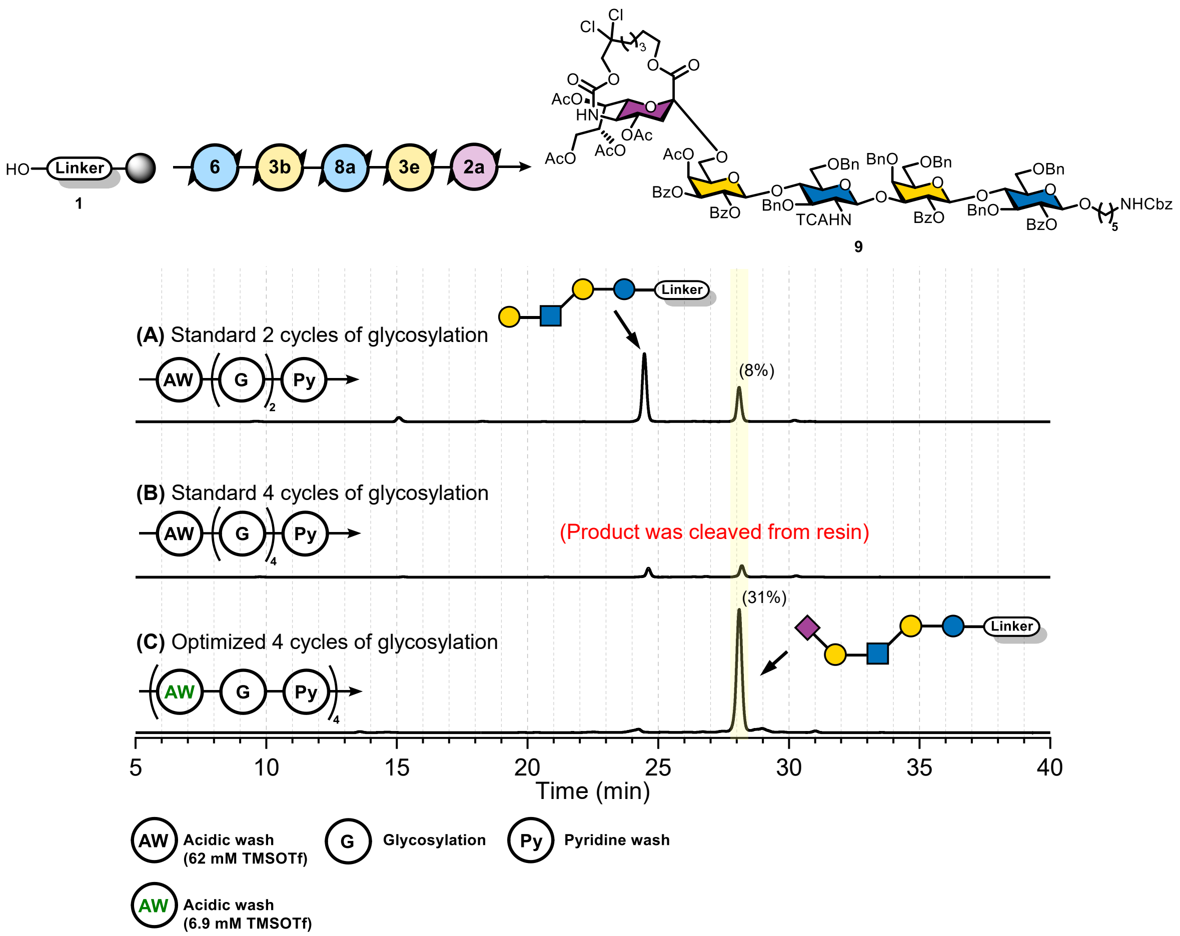

Supplementary Fig. 3 AGA optimization of pentasaccharide **9** with different synthetic programs. (A) Standard two cycles of glycosylation; (B) Standard four cycles of glycosylation; (C) Optimized four cycles of glycosylation. Isolated yields after NP-HPLC purification are shown in parentheses. Source data are provided as a Source Data file.

2.4 AGA Optimization of pentasaccharide **11**

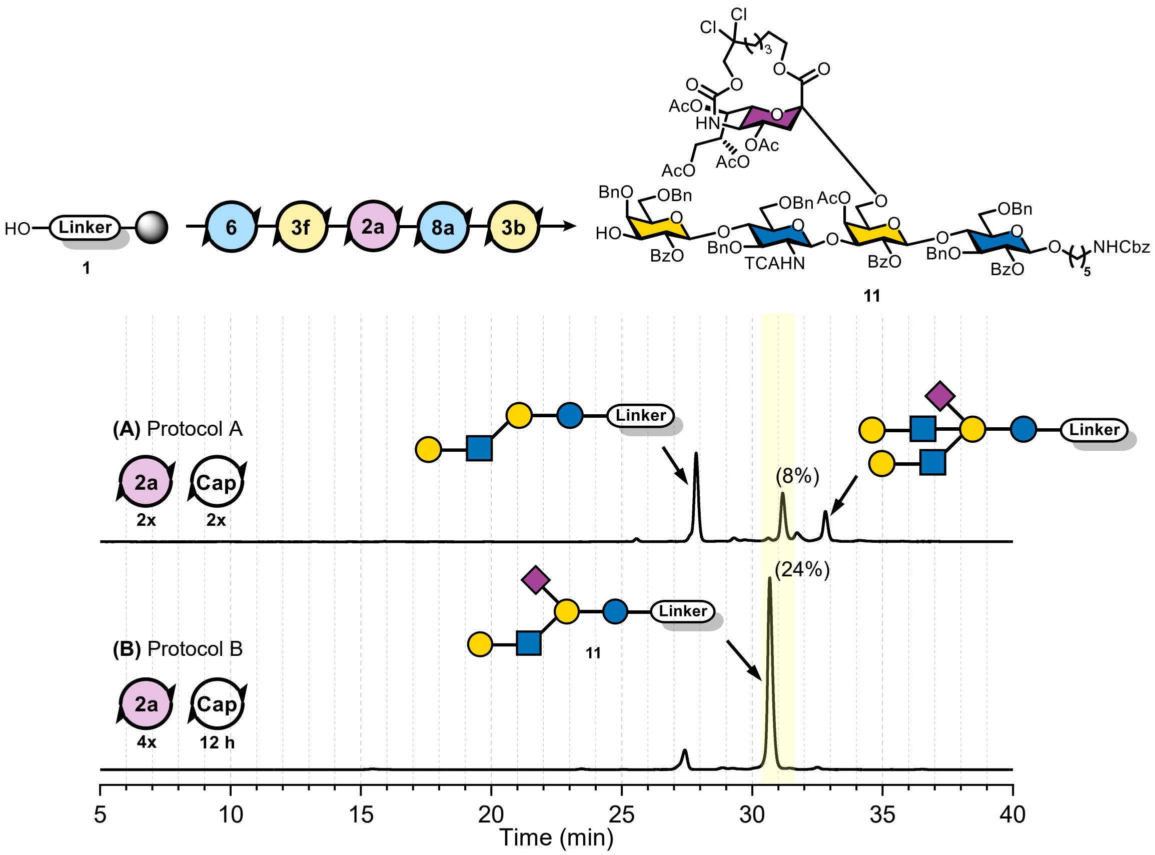

Supplementary Fig. 4 AGA Optimization of pentasaccharide **11** (A) Two cycles of sialylation and two standard capping (Ac_2O, MsOH, CH_2Cl_2, 20 min); (B) Four cycles of sialylation and prolonged basic capping (Ac_2O, pyr., CH_2Cl_2, 12 h). Isolated yields after NP-HPLC purification are shown in parentheses. Source data are provided as a Source Data file.

2.5 Protecting groups on glucosamine affect α(2,6)-sialylation

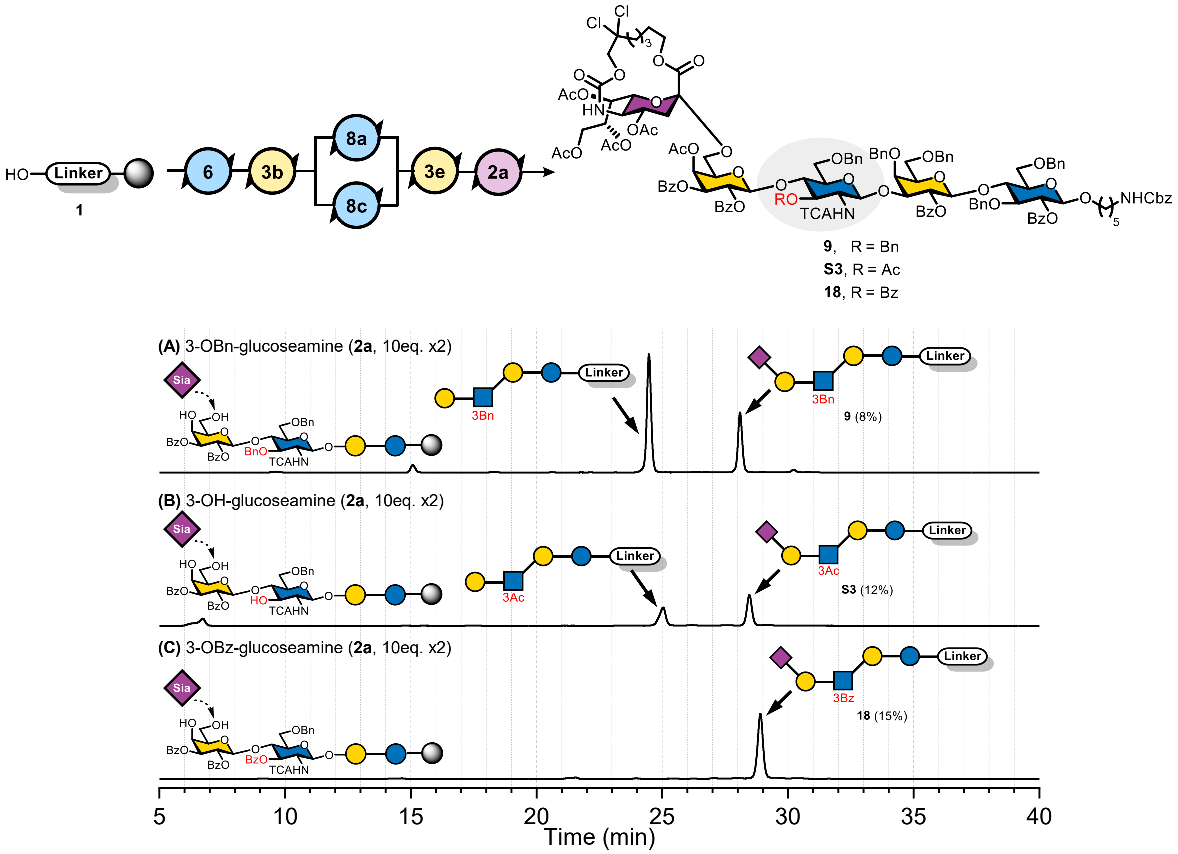

Supplementary Fig. 5 Protecting groups on glucosamine affect α(2,6)-sialylation. (A) 3-OBn while sialylation; (B) 3-OH while sialylation; (C) 3-OBz while sialylation. Isolated yields after NP-HPLC purification are shown in parentheses. Source data are provided as a Source Data file.

2.6 AGA Optimization of protected DSLNF II **17** (Route A1 and A2)

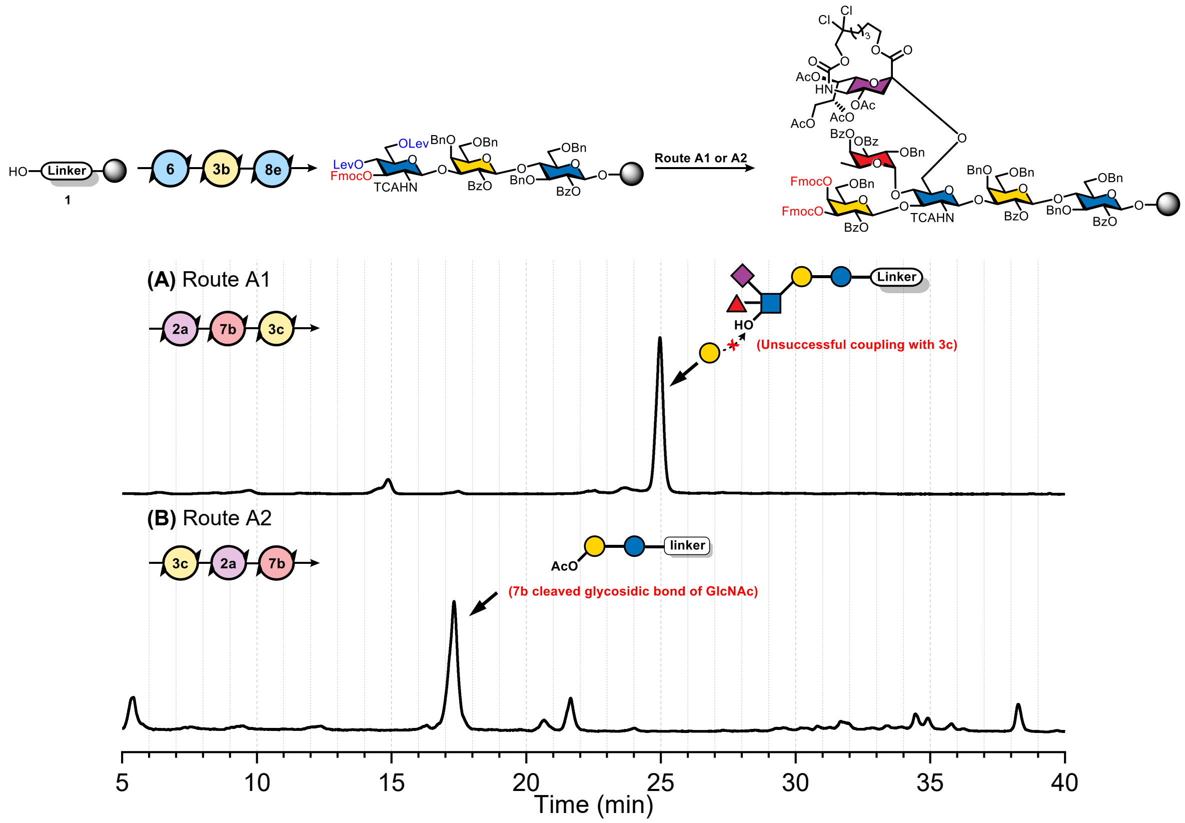

Supplementary Fig. 6 AGA optimization of protected DSLNF II **17**. (A) The NP-HPLC trace of route A1: Sia **2a**-Fuc **7b**-Gal **3c**; (B) The NP-HPLC trace of route A2: Gal **3c**-Sia **2a**-Fuc **7b**. Source data are provided as a Source Data file.

2.7 AGA Optimization of protected DSLNF II **17** (Route B1)

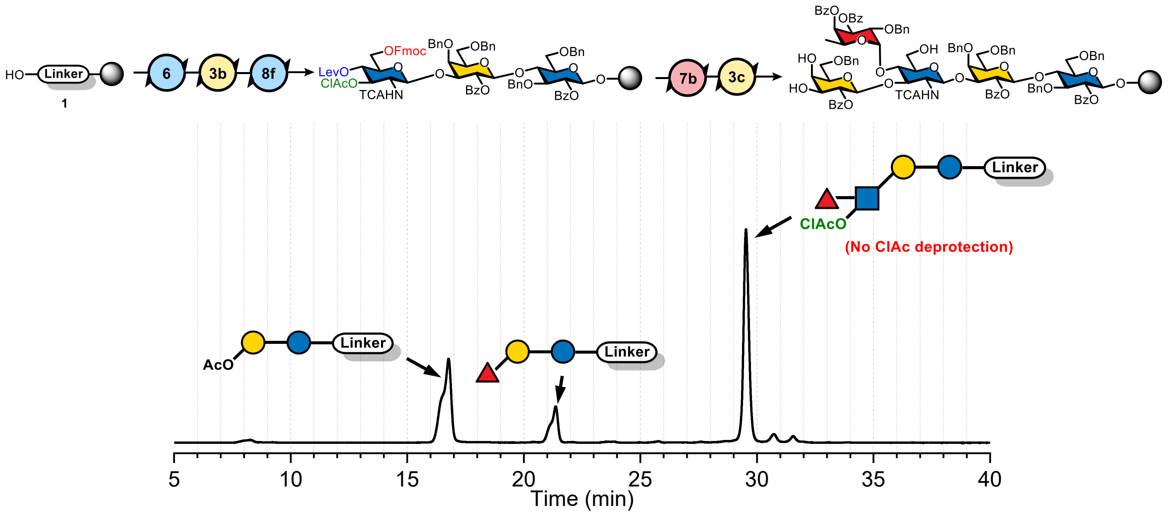

Supplementary Fig. 7 The NP-HPLC trace of the AGA synthesis for protected DSLNF II **17** using route B1. Source data are provided as a Source Data file.

2.8 AGA Optimization of protected DSLNF II **17** (Route B2)

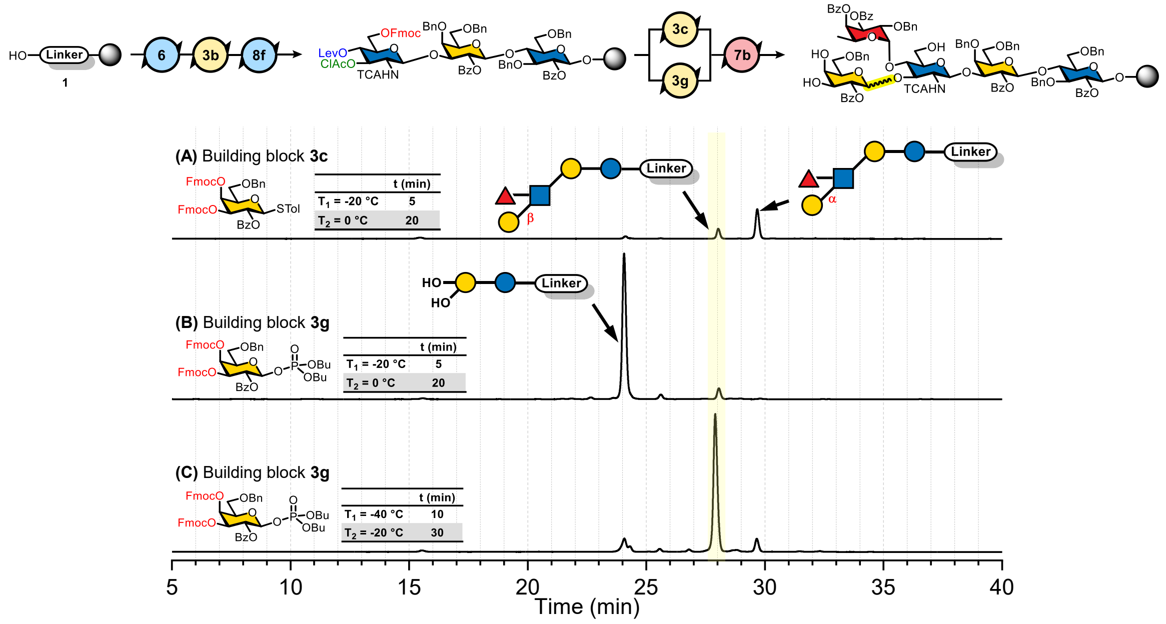

Supplementary Fig. 8 AGA of pentasaccharide intermediate for protected DSLNF II **17** using different galactose building blocks and temperatures. (A) Using building block **3c**; (B) Using building block **3g** under higher temperature range; (C) Using building block **3g** under lower temperature range. Source data are provided as a Source Data file.

2.9 Optimization of global deprotection of pentasaccharide **9**

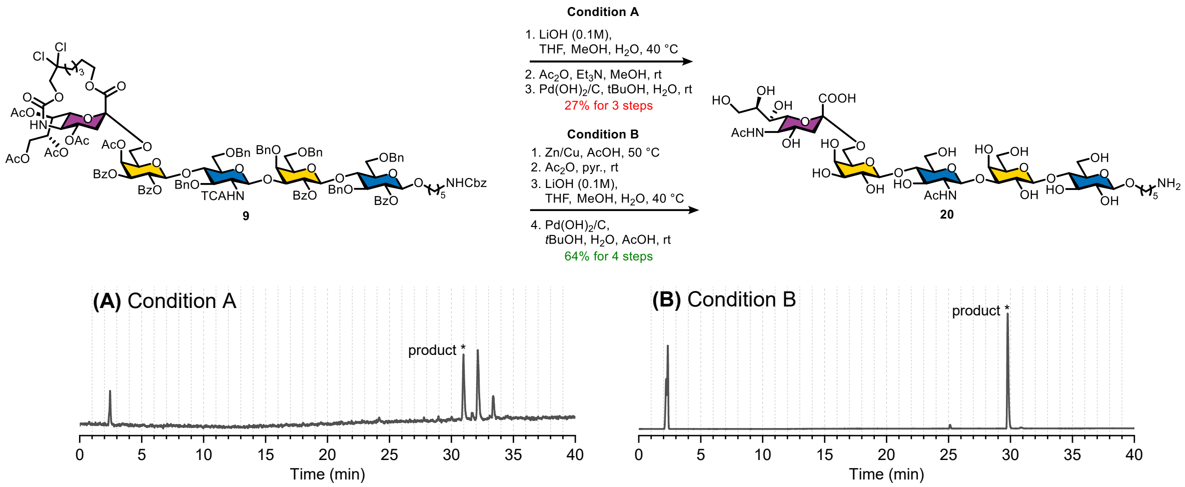

Supplementary Fig. 9 Comparison of global deprotection of pentasaccharide **9** using different protocols. (A) The RP-HPLC trace of condition A; (B) The RP-HPLC trace of condition B. Source data are provided as a Source Data file.

3 Synthesis of Building Blocks

3.1 Sialic Acid Building Blocks

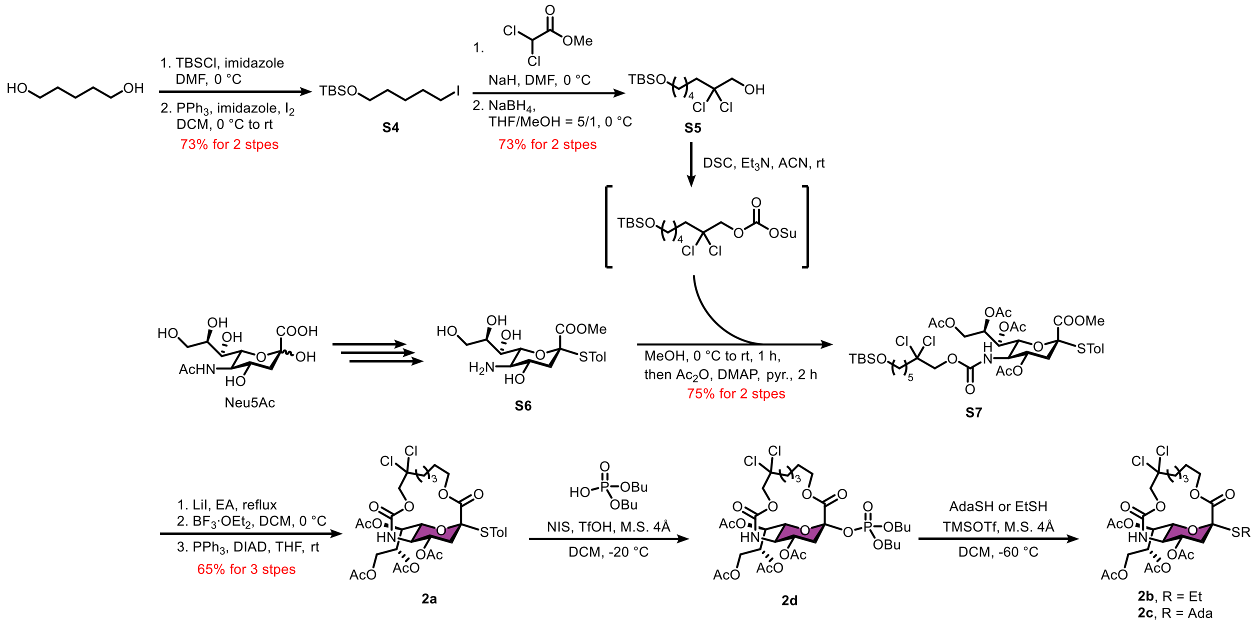

Iodo-5-(*tert*-butyldimethylsilyloxy)-pentane (Compound S4)[1]

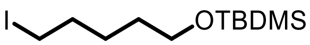

S4

To a solution of 1.5-pentanediol (20.8 mL, 199.05 mmol, 3.0 equiv.) and imidazole (9.03 g, 132.70 mmol, 2.0 equiv.) in anhydrous DMF (133 mL) was added *tert*-butyldimethylsilyl chloride (TBSCl, 10.00 g, 66.35 mmol, 1.0 equiv.) in anhydrous DMF (27 mL) dropwise over one hour at 0 °C under nitrogen atmosphere, and the suspension was warmed to room temperature. After stirring for one hour at room temperature, the suspension was extracted with EtOAc, and the resulting solution was washed saturated $NH_4HCO_{3(aq)}$ and brine. The organic layer was dried over Na_2SO_4, filtered, and concentrated. The crude was dissolved in CH_2Cl_2 (133 mL) and cooled to 0 °C. Triphenylphosphine (PPh_3, 17.40 g, 66.35 mmol, 1.0 equiv.), imidazole (5.42 g, 79.62 mmol, 1.2 equiv.) and iodine (16.84 g, 66.35 mmol, 1.0 equiv.) was added to the mixture at 0 °C, and the suspension was warmed to room temperature. After stirring for one hour, the mixture was quenched by addition of saturated $Na_2S_2O_{3(aq)}$ at 0 °C. The residue was extracted with EtOAc, and the resulting solution was washed with H_2O and brine. The organic layer was dried over

Na_2SO_4, filtered, concentrated, and purified by flash column chromatography (SiO_2, Hex/EtOAc = 6:1) to obtain compound **S4** (16.03 g, 73%).

^{1}H NMR (400 MHz, $CDCl_3$): δ 3.61 (td, *J* = 6.2, 1.2 Hz, 1H), 3.19 (td, *J* = 7.0, 1.3 Hz, 1H), 1.84 (pd, *J* = 7.0, 1.2 Hz, 1H), 1.58 – 1.49 (m, 1H), 1.48 – 1.39 (m, 1H), 0.89 (s, 9H), 0.04 (s, 6H).

^{13}C NMR (101 MHz, $CDCl_3$): δ 63.0, 33.5, 31.8, 27.1, 26.1, 18.5, 7.2, 7.2, -5.1.

^{1}H-NMR (400 MHz, $CDCl_3$)

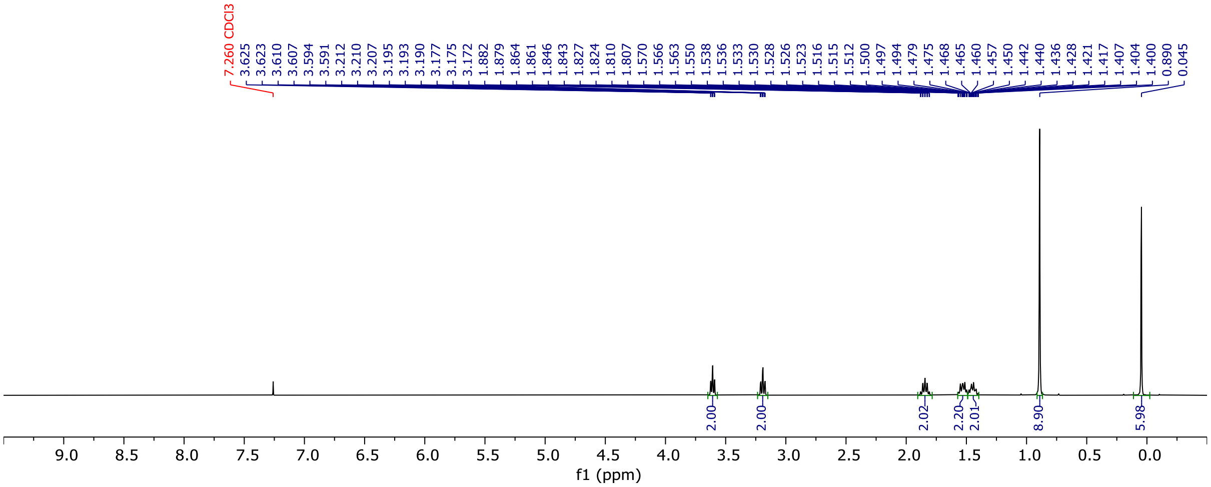

^{13}C-NMR (101 MHz, $CDCl_3$)

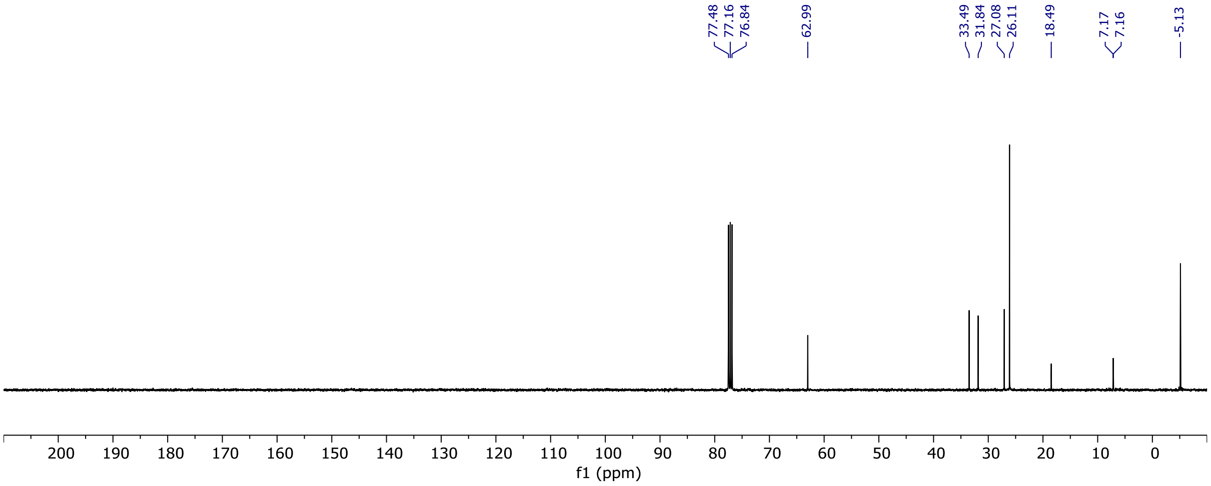

2,2-Dichloro-7-(*tert*-butyldimethylsilyloxy)-1-heptanol (Compound S5)[1]

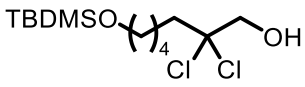

S5

To a solution of compound **S4** (15.19 g, 46.26 mmol, 1.0 equiv.) and methyl dichloroacetate (19.2 mL, 185.04 mmol, 4.0 equiv.) in anhydrous DMF (231 mL) was added sodium hydride (60 % dispersion in mineral oil) (NaH, 7.40 g, 185.04 mmol, 4.0 equiv.) in four portions at 0 °C under nitrogen atmosphere. After stirring for two hours at 0 °C, the suspension was quenched by addition of saturated $NH_4HCO_{3(aq)}$ at 0 °C. The residue was extracted with CH_2Cl_2, and the resulting solution was washed H_2O and brine. The organic layer was dried over Na_2SO_4, filtered, and concentrated. The crude was dissolved in THF/MeOH (193 mL/39 mL) and cooled to 0 °C. Sodium borohydride ($NaBH_4$, 7.00 g, 185.04 mmol, 4.0 equiv.) was added to the mixture in four portions at 0 °C. After stirring for one hour, the mixture was quenched by addition of saturated $NH_4HCO_{3(aq)}$ at 0 °C. The residue was extracted with CH_2Cl_2, and the resulting solution was washed with H_2O and brine. The organic layer was dried over Na_2SO_4, filtered, concentrated, and purified by flash column chromatography (SiO_2, Hex/EtOAc = 10:1) to obtain compound **S5** (10.61 g, 73%).

^{1}H NMR (400 MHz, $CDCl_3$): δ 3.90 (s, 2H), 3.62 (d, *J* = 6.6 Hz, 1H), 2.25 – 2.18 (m, 2H), 1.72 – 1.61 (m, 2H), 1.59 – 1.51 (m, 2H), 1.45 – 1.36 (m, 2H), 0.89 (s, 9H), 0.04 (s, 6H).

^{13}C NMR (101 MHz, $CDCl_3$): δ 94.7, 72.3, 63.1, 43.7, 32.6, 26.1, 25.5, 24.8, 18.5, -5.1.

^{1}H-NMR (400 MHz, $CDCl_3$)

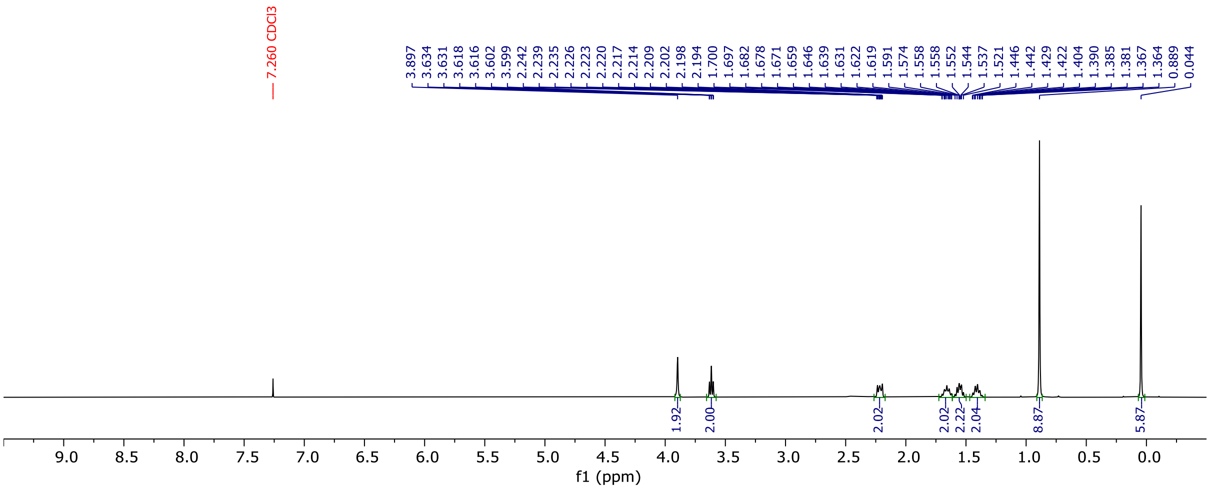

^{13}C-NMR (101 MHz, $CDCl_3$)

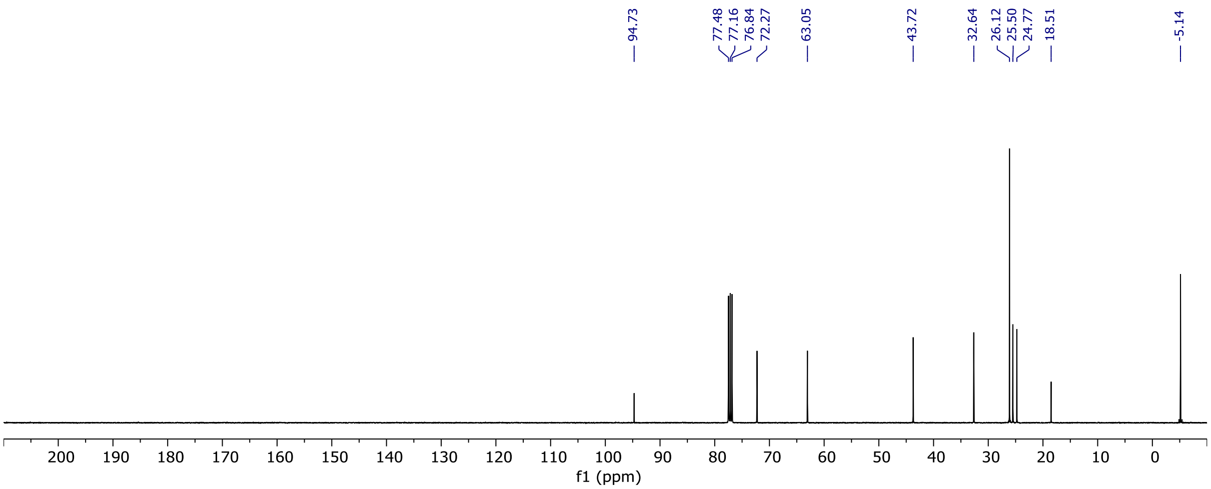

Methyl *p*-tolyl 5-(2,2-dichloro-7-(*tert*-butyldimethylsilyloxy)-heptoxycarbonylamino)-4,7,8,9-tetra-*O*-acetyl-3,5-dideoxy-2-thio-D-glycero-α-D-galacto-2-nonulopyranosonate (Compound S7)

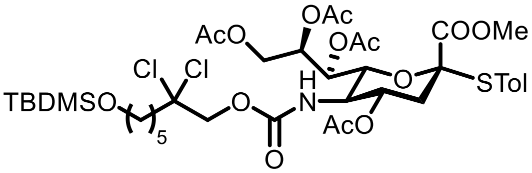

S7

To a solution of compound **S5** (38.81 g, 123.06 mmol, 3.0 equiv.) in anhydrous MeCN (308 mL) was added *N*,*N'*-disuccinimidyl carbonate (DSC, 31.52 g, 123.06 mmol, 3.0 equiv.) and triethylamine (Et_3N, 34.30 mL, 246.12 mmol, 6 equiv.) at room temperature under nitrogen atmosphere. After stirring for one hour at room temperature, the resulting mixture of activated carbonate was used without further purification.

Next, to a solution of compound **S6**[2-4] (20.00 g, 41.02 mmol, 1.0 equiv.) in anhydrous MeOH (308 mL) was added previous mixture of activated carbonate dropwise over one hour at 0 °C under nitrogen atmosphere, and the suspension was warmed to room temperature. After stirring for one hour, the suspension was concentrated *in vacuo*. The residue was extracted with EtOAc, and the resulting solution was washed with H_2O and brine. The organic layer was dried over Na_2SO_4, filtered, and concentrated. The crude was dissolved in CH_2Cl_2 (308 mL) and cooled to 0 °C. Pyridine (33.0 mL, 410.20 mmol, 10.0 equiv.), acetic anhydride (Ac_2O, 38.7 mL, 410.20 mmol, 10.0 equiv.) and 4-

dimethylaminopyridine (DMAP, 501 mg, 4.10 mmol, 0.1 equiv.) was added to the mixture at 0 °C, and the suspension was warmed to room temperature. After stirring for twelve hours, the suspension was concentrated *in vacuo*. The residue was extracted with EtOAc, and the resulting solution was washed with 1N $HCl_{(aq)}$, saturated $NaHCO_{3(aq)}$ and brine. The organic layer was dried over Na_2SO_4, filtered, concentrated, and purified by flash column chromatography (SiO_2, Hex/EtOAc = 2:1) to obtain compound **S7** (27.50 g, 75%).

^{1}H NMR (400 MHz, $CDCl_3$): δ 7.38 (d, *J* = 8.1 Hz, 2H), 7.13 (d, *J* = 8.1 Hz, 2H), 5.35 (dd, *J* = 7.6, 1.9 Hz, 1H), 5.27 (ddd, *J* = 7.7, 5.3, 2.7 Hz, 1H), 4.89 (td, *J* = 10.9, 4.6 Hz, 1H), 4.66 (d, *J* = 9.9 Hz, 1H), 4.61 (d, *J* = 11.8 Hz, 1H), 4.38 (dd, *J* = 12.5, 2.7 Hz, 1H), 4.25 – 4.17 (m, 2H), 3.93 (dd, *J* = 10.7, 1.8 Hz, 1H), 3.67 – 3.51 (m, 6H), 2.82 (dd, *J* = 12.9, 4.7 Hz, 1H), 2.36 (s, 3H), 2.17 – 2.07 (m, 5H), 2.05 (s, 3H), 2.04 (s, 3H), 2.00 (s, 3H), 1.93 (t, *J* = 12.2 Hz, 1H), 1.69 – 1.59 (m, 2H), 1.59 – 1.50 (m, 2H), 1.44 – 1.34 (m, 2H), 0.88 (s, 9H), 0.04 (s, 6H).

^{13}C NMR (101 MHz, $CDCl_3$): δ 170.8, 170.6, 170.1, 170.0, 168.0, 154.8, 140.4, 136.6, 129.8, 125.2, 90.4, 87.4, 77.5, 77.4, 77.2, 76.8, 74.5, 71.7, 69.7, 69.5, 67.9, 63.0, 62.0, 52.9, 51.6, 44.3, 38.3, 32.7, 26.1, 25.4, 24.7, 21.5, 21.1, 21.0, 21.0, 20.9, 18.5, -5.2.

HRMS (QToF): Calcd for $C_{39}H_{59}Cl_2NNaO_{14}SSi$ $[M + Na]^+$ 918.2700; found 918.2753.

^{1}H-NMR (400 MHz, $CDCl_3$)

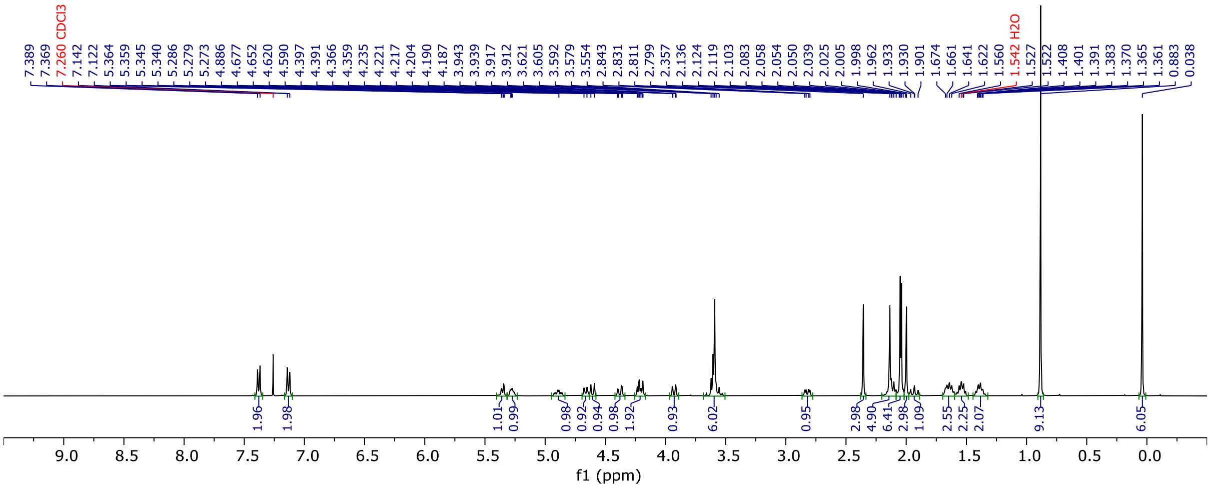

^{13}C-NMR (101 MHz, $CDCl_3$)

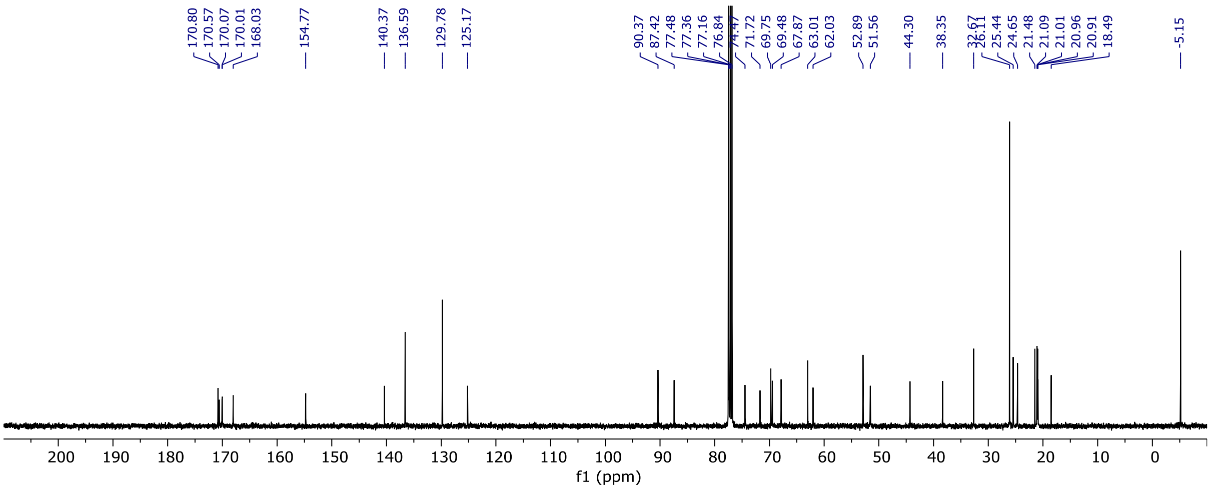

***p*-Tolyl 5-(((2',2'-dichloroheptoxy)oxy)carbonylamino)-4,7,8,9-tetra-*O*-acetyl-3,5-dideoxy-2-thio-D-glycero-α-D-galacto-1,7'-lactono-2-nonulopyranosonate (Compound 2a)**

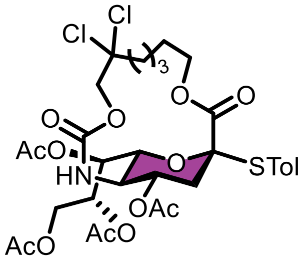

2a

To a solution of compound **S7** (4.50 g, 5.02 mmol, 1.0 equiv.) in EtOAc (50 mL) was added lithium iodide (LiI, 3.36 g, 25.09 mmol, 5.0 equiv.) at room temperature under nitrogen atmosphere. After refluxing for 18 hours, the suspension was concentrated *in vacuo*. The residue was extracted with EtOAc, and the resulting solution was washed with 1N $HCl_{(aq)}$ and brine. The organic layer was dried over Na_2SO_4, filtered, and concentrated. The crude was dissolved in CH_2Cl_2 (50 mL) and cooled to 0 °C. Boron trifluoride etherate ($BF_3 \cdot OEt_2$, 743 μL, 6.02 mmol, 1.2 equiv.) was added to the mixture at 0 °C. After stirring for one hour, the suspension was quenched by addition of saturated $NaHCO_{3(aq)}$ at 0 °C. The residue was extracted with EtOAc, and the resulting solution was washed with 1N $HCl_{(aq)}$ and brine. The organic layer was dried over Na_2SO_4, filtered, and concentrated. To a solution of triphenylphosphine (PPh_3, 10.53 g, 40.14 mmol, 8.0 equiv.) in THF (1000 mL) was added a solution of the crude from previous step and diisopropyl azodicarboxylate

(DIAD, 7.9 mL, 40.14 mmol, 8.0 equiv.) dropwise over two hours at room temperature under nitrogen atmosphere. After stirring for one hour, the suspension was quenched by addition of AcOH and MeOH. The suspension was concentrated *in vacuo*, and purified by flash column chromatography (SiO_2, Hex/EtOAc = 2:1) and size-exclusion chromatography with LH-20 (CH_2Cl_2/MeOH = 1:1) to obtain compound **2a** (2.44 g, 65%).

^{1}H NMR (700 MHz, *d6*-DMSO, 70 °C): δ 7.38 (d, *J* = 8.1 Hz, 2H), 7.19 (d, *J* = 7.7 Hz, 2H), 5.32 (br, 1H), 5.22 (dd, *J* = 5.5, 1.9 Hz, 1H), 5.16 (ddd, *J* = 6.9, 5.5, 3.1 Hz, 1H), 4.52 – 4.45 (m, 1H), 4.44 – 4.40 (m, 1H), 4.38 (dd, *J* = 12.2, 3.2 Hz, 1H), 4.34 – 4.25 (m, 1H), 4.24 – 4.20 (m, 1H), 4.18 (dd, *J* = 12.2, 6.8 Hz, 1H), 3.99 – 3.92 (m, 1H), 2.86 – 2.74 (m, 2H), 2.33 (s, 4H), 2.20 – 2.14 (m, 1H), 2.13 (s, 3H), 2.01 (s, 3H), 2.00 (s, 3H), 1.98 (s, 3H), 1.78 – 1.65 (m, 3H), 1.58 – 1.41 (m, 4H).

^{13}C NMR (176 MHz, *d6*-DMSO, 70 °C): δ 169.5, 169.0, 169.0, 168.9, 167.0, 152.8, 139.5, 135.8, 129.2, 124.6, 90.2, 86.6, 72.9, 69.9, 68.1, 66.9, 64.4, 61.4, 51.4, 44.2, 37.7, 26.1, 23.7, 22.0, 20.4, 20.2, 20.2, 20.2, 20.0.

HRMS (QToF): Calcd for $C_{32}H_{41}Cl_2NNaO_{13}S$ [M + Na]$^+$ 772.1573; found 772.1606.

^{1}H-NMR (700 MHz, *d6*-DMSO, 70 °C)

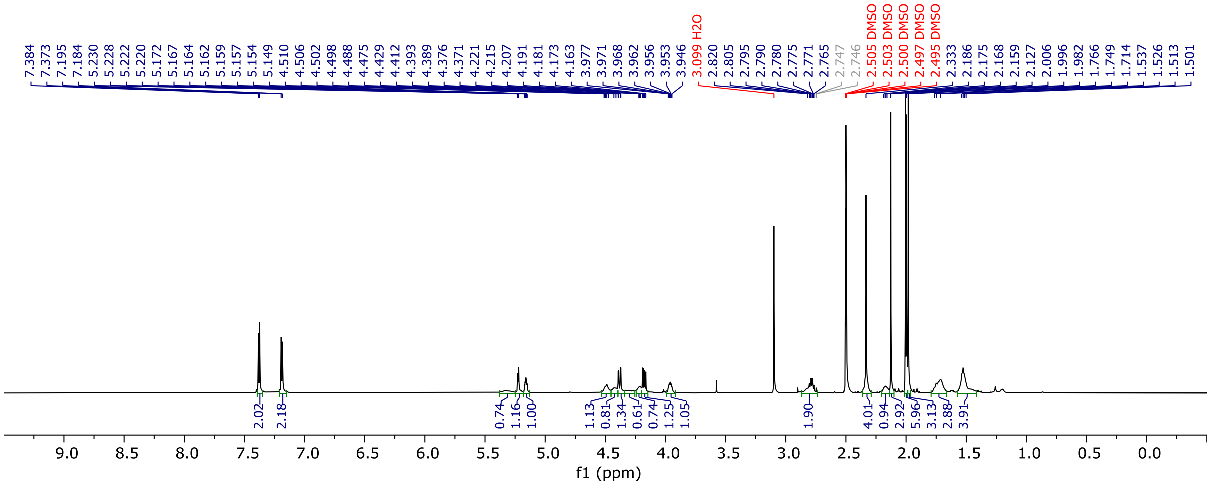

^{13}C-NMR (176 MHz, *d6*-DMSO, 70 °C)

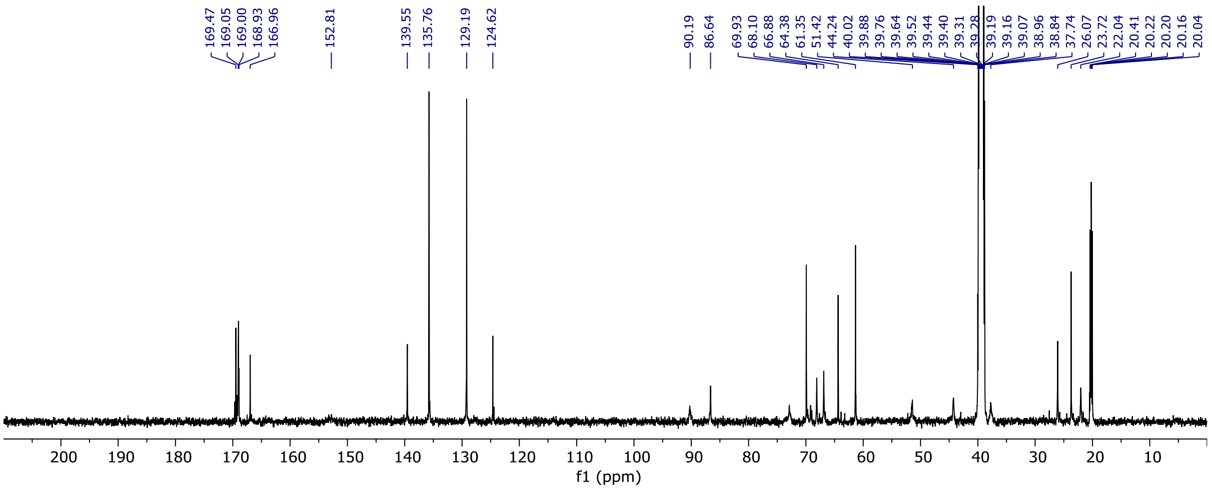

Dibutyl 5-(((2',2'-dichloroheptoxy)oxy)carbonylamino)-4,7,8,9-tetra-*O*-acetyl-3,5-dideoxy-2-phosphate-D-glycero-α-D-galacto-1,7'-lactono-2-nonulopyranosonate (Compound 2d)

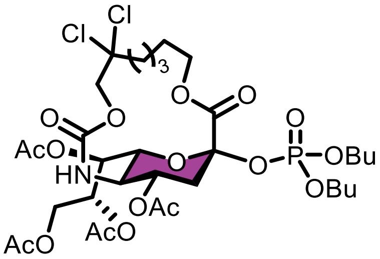

2d

To a solution of compound **2a** (100 mg, 0.13 mmol, 1.0 equiv.), dibutyl phosphate (66 μL, 0.33 mmol, 2.5 equiv.) and 4 Å molecular sieves (200 mg) in anhydrous CH_2Cl_2 (2 mL) was stirred at room temperature for 30 min and cooled to -20 °C. *N*-iodosuccinimide (NIS, 60 mg, 0.27 mmol, 2.0 equiv.) and trifluoromethanesulfonic acid (TfOH, 1.8 μL, 0.02 mmol, 0.15 equiv.) was added at -20 °C under nitrogen atmosphere. After stirring for one hour, the suspension was quenched by addition of Et_3N at -20 °C and filtered through a pad of celite. The residue was extracted with EtOAc, and the resulting solution was washed with $Na_2S_2O_{3(aq)}$, H_2O and brine. The organic layer was dried over Na_2SO_4, filtered, concentrated, and purified by flash column chromatography (SiO_2, Hex/EtOAc = 1:2) to obtain compound **2d** (110 mg, 98%).

^{1}H NMR (400 MHz, $CDCl_3$): δ 5.48 (br, 1H), 5.31 (s, 3H), 4.86 (br, 1H), 4.53 (br, 1H), 4.38 – 4.24 (m, 3H), 4.16 – 3.89 (m, 5H), 2.95 (dd, *J* = 12.7, 5.4 Hz, 1H), 2.76 (ddd, *J* = 11.1,

10.1, 6.6 Hz, 1H), 2.42 (br, 1H), 2.36 – 2.21 (m, 2H), 2.16 (s, 3H), 2.10 (s, 3H), 2.04 (s, 6H), 2.01 – 1.91 (m, 1H), 1.79 – 1.48 (m, 9H), 1.47 – 1.31 (m, 4H), 0.91 (t, *J* = 7.0 Hz, 3H), 0.89 (t, *J* = 7.8 Hz, 3H).

^{13}C NMR (101 MHz, $CDCl_3$): δ 171.4, 170.8, 170.1, 169.7, 165.9, 165.8, 154.0, 98.2, 89.0, 77.4, 71.2, 70.6, 68.6, 68.2, 68.0, 68.0, 66.4, 64.1, 61.6, 52.4, 44.9, 37.4, 32.2, 32.2, 32.2, 32.1, 29.8, 26.8, 23.4, 22.0, 21.1, 21.0, 20.9, 18.7, 18.7, 13.7, 13.7.

HRMS (QToF): Calcd for $C_{33}H_{52}Cl_2NNaO_{17}P$ $[M + Na]^+$ 858.2248; found 858.2281.

^{1}H-NMR (400 MHz, $CDCl_3$)

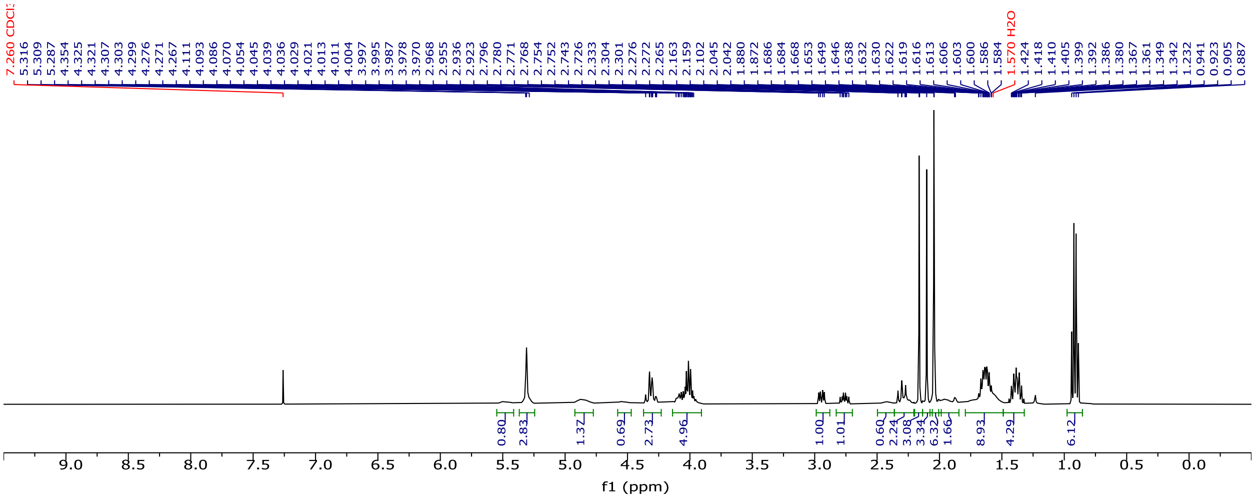

^{13}C-NMR (101 MHz, $CDCl_3$)

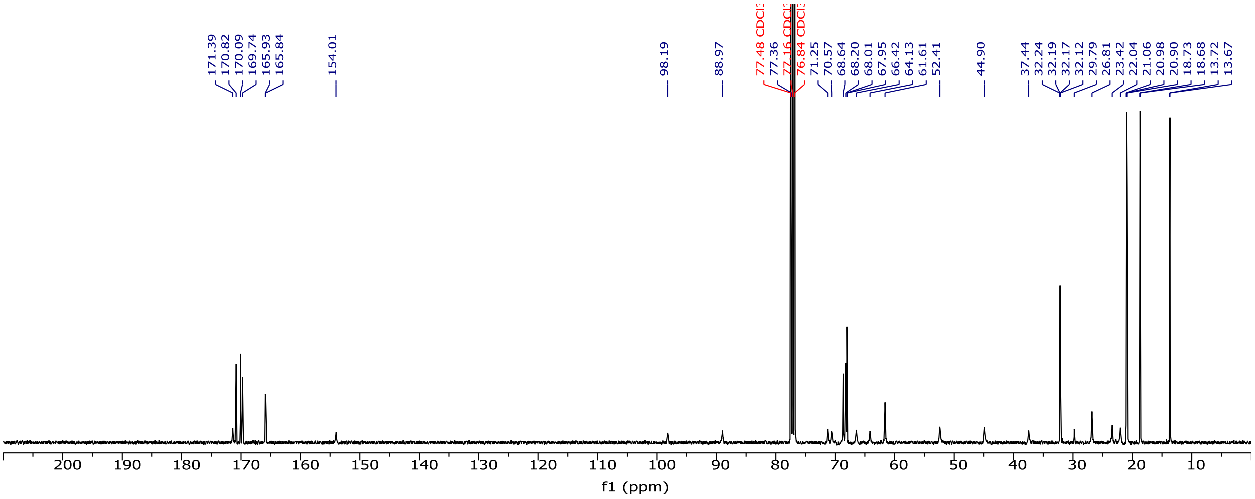

Ethyl 5-(((2',2'-dichloroheptoxy)oxy)carbonylamino)-4,7,8,9-tetra-*O*-acetyl-3,5-dideoxy-2-thio-D-glycero-α-D-galacto-1,7'-lactono-2-nonulopyranosonate (Compound 2b)

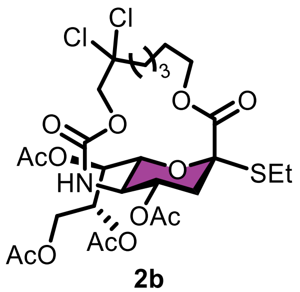

2b

To a solution of compound **2d** (335 mg, 0.40 mmol, 1.0 equiv.), ethanethiol (58 μL, 0.80 mmol, 2.0 equiv.) and 4 Å molecular sieves (800 mg) in anhydrous CH_2Cl_2 (8 mL) was stirred at room temperature for 30 min and cooled to -60 °C. Trimethylsilyl trifluoromethanesulfonate (TMSOTf, 87 μL, 0.48 mmol, 1.2 equiv.) was added at -60 °C under nitrogen atmosphere. After stirring for one hour, the suspension was quenched by addition of Et_3N at -60 °C and filtered through a pad of celite. The residue was extracted with EtOAc, and the resulting solution was washed with H_2O and brine. The organic layer was dried over Na_2SO_4, filtered, concentrated, and purified by flash column chromatography (SiO_2, Hex/EtOAc = 2:1) to obtain compound **2b** (205 mg, 75%).

^{1}H NMR (700 MHz, *d6*-DMSO, 70 °C): δ 5.41 – 5.27 (m, 1H), 5.26 – 5.16 (m, 2H), 4.70 – 4.37 (m, 3H), 4.37 – 4.15 (m, 2H), 4.07 (dd, *J* = 11.1, 4.6 Hz, 1H), 4.00 (dt, *J* = 10.8, 5.1 Hz, 1H), 2.93 – 2.84 (m, 1H), 2.83 – 2.75 (m, 1H), 2.75 – 2.67 (m, 1H), 2.64 – 2.56 (m, 1H), 2.42 – 2.31 (m, 1H), 2.27 – 2.17 (m, 1H), 2.12 (s, 3H), 2.05 (s, 3H), 2.02 (s, 3H), 1.98 (s, 3H), 1.84 – 1.76 (m, 2H), 1.74 (t, *J* = 12.0 Hz, 1H), 1.63 – 1.46 (m, 4H), 1.13 (t, *J* = 7.5 Hz, 3H).

^{13}C NMR (176 MHz, *d6*-DMSO, 70 °C): δ 169.5, 169.1, 169.0, 168.9, 167.4, 153.2, 90.4, 82.6, 72.1, 67.8, 67.1, 64.4, 61.5, 51.6, 44.2, 37.7, 26.2, 23.4, 22.3, 22.0, 20.4, 20.2, 20.0, 13.8.

HRMS (QToF): Calcd for $C_{27}H_{39}Cl_2NNaO_{13}S$ $[M + Na]^+$ 710.1417; found 710.1444.

^{1}H-NMR (700 MHz, *d6*-DMSO, 70 °C)

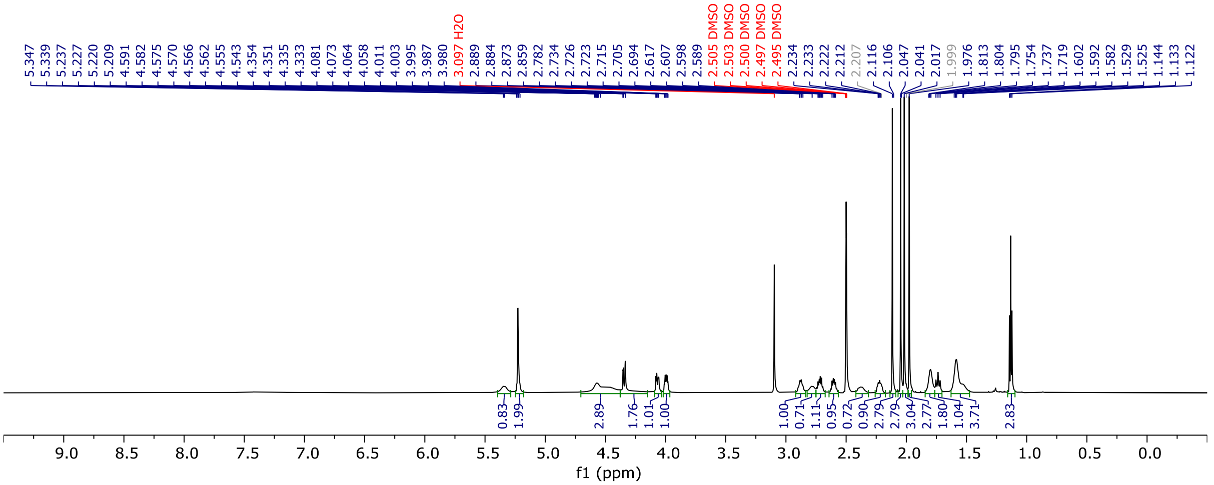

^{13}C-NMR (176 MHz, *d6*-DMSO, 70 °C)

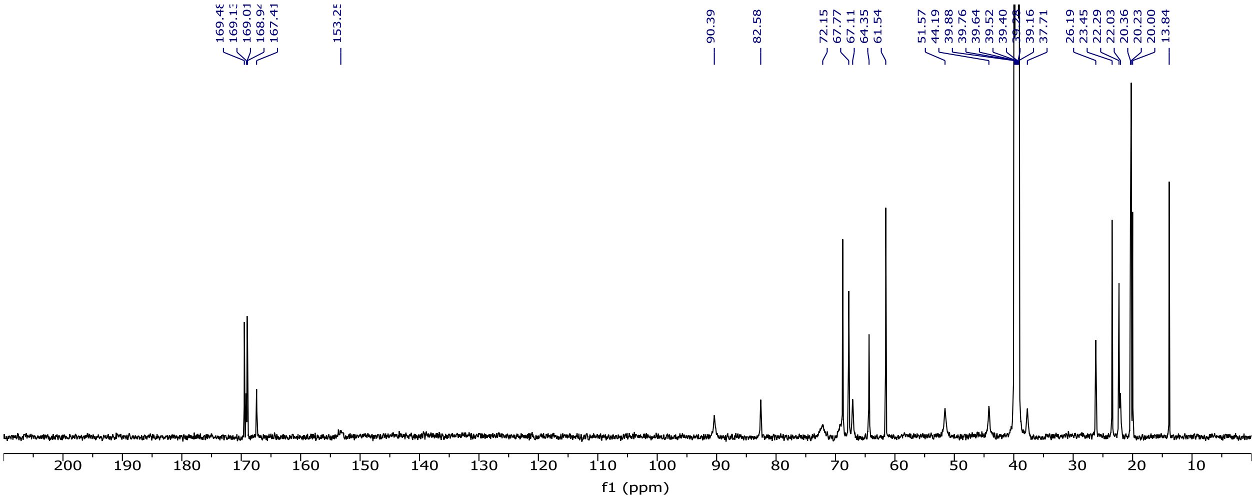

1-Adamantyl 5-(((2',2'-dichloroheptoxy)oxy)carbonylamino)-4,7,8,9-tetra-*O*-acetyl-3,5-dideoxy-2-thio-D-glycero-α-D-galacto-1,7'-lactono-2-nonulopyranosonate (Compound 2c)

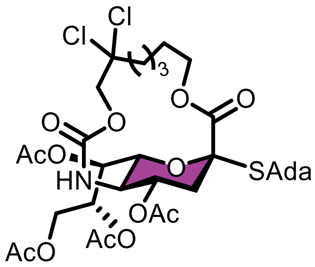

2c

To a solution of compound **2d** (335 mg, 0.40 mmol, 1.0 equiv.), 1-adamantanethiol (134 mg, 0.80 mmol, 2.0 equiv.) and 4 Å molecular sieves (800 mg) in anhydrous CH_2Cl_2 (8 mL) was stirred at room temperature for 30 min and cooled to -60 °C. Trimethylsilyl trifluoromethanesulfonate (TMSOTf, 87 μL, 0.48 mmol, 1.2 equiv.) was added at -60 °C under nitrogen atmosphere. After stirring for one hour, the suspension was quenched by addition of Et_3N at -60 °C and filtered through a pad of celite. The residue was extracted with EtOAc, and the resulting solution was washed with H_2O and brine. The organic layer was dried over Na_2SO_4, filtered, concentrated, and purified by flash column chromatography (SiO_2, Hex/EtOAc = 2:1) to obtain compound **2c** (265 mg, 83%).

^{1}H NMR (700 MHz, *d6*-DMSO, 70 °C): δ 5.34 – 5.24 (m, 1H), 5.24 – 5.18 (m, 2H), 4.70 – 4.52 (m, 2H), 4.51 – 4.35 (m, 2H), 4.33 (dd, *J* = 12.0, 2.3 Hz, 1H), 4.08 (dd, *J* = 11.6, 3.9 Hz, 1H), 4.01 – 3.92 (m, 1H), 2.85 (td, *J* = 10.5, 6.8 Hz, 1H), 2.80 – 2.72 (m, 1H), 2.44 – 2.35 (m, 1H), 2.24 – 2.16 (m, 1H), 2.12 (s, 3H), 2.06 (s, 3H), 2.01 (s, 3H), 1.99 – 1.93 (m, 9H), 1.90 – 1.85 (m, 3H), 1.83 – 1.79 (m, 2H), 1.75 (t, *J* = 12.0 Hz, 1H), 1.67 – 1.61 (m, 6H), 1.61 – 1.48 (m, 4H).

^{13}C NMR (176 MHz, *d6*-DMSO, 70 °C): δ 169.4, 169.1, 169.0, 168.9, 168.7, 153.2, 90.3, 84.2, 71.9, 68.9, 67.9, 66.8, 64.1, 61.4, 51.5, 50.5, 44.2, 42.8, 35.3, 29.1, 26.2, 23.4, 22.0, 20.4, 20.3, 20.2, 20.0.

HRMS (QToF): Calcd for $C_{35}H_{49}Cl_2NNaO_{13}S$ $[M + Na]^+$ 816.2199; found 816.2236.

^{1}H-NMR (700 MHz, *d6*-DMSO, 70 °C)

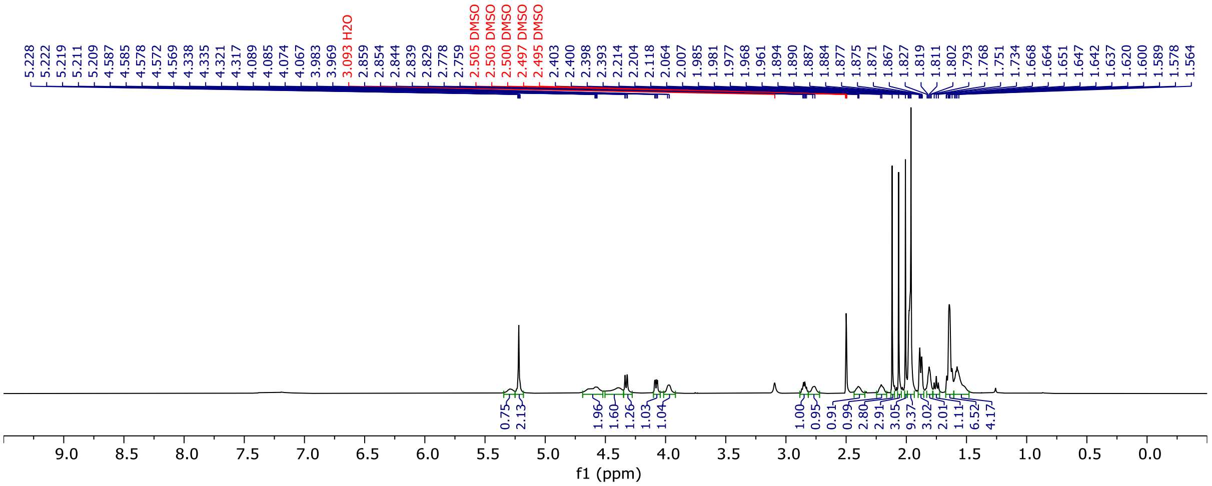

^{13}C-NMR (176 MHz, *d6*-DMSO, 70 °C)

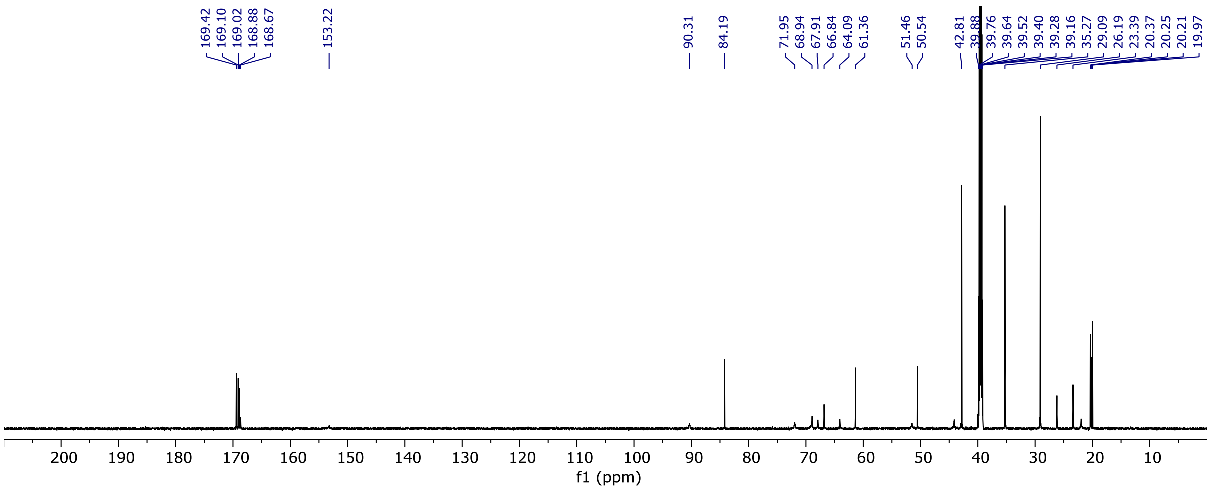

3.2 Galactose Building Blocks

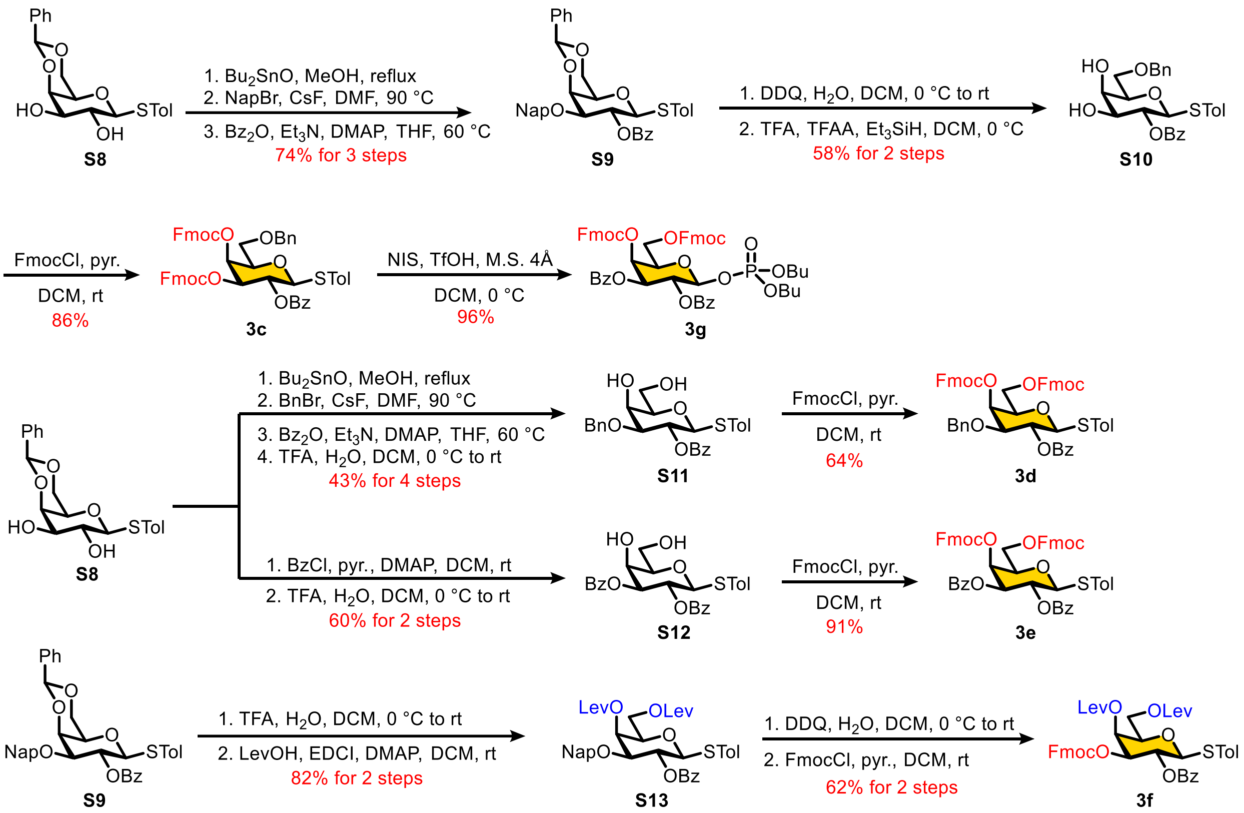

***p*-Methylphenyl 2-*O*-benzoyl-4,6-*O*-benzylidene-3-*O*-(2-naphthalenylmethyl)-1-thio-β-D-galatopyranoside (Compound S9)**

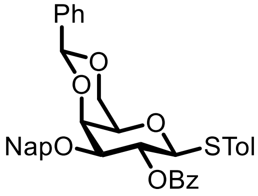

S9

To a solution of compound **S8** (30.00 g, 80.12 mmol, 1.0 equiv.) in anhydrous MeOH (400 mL) was added dibutyltin oxide (Bu_2SnO, 23.93 g, 96.14 mmol, 1.2 equiv.) at room temperature under nitrogen atmosphere. After refluxing for three hours, the suspension was concentrated *in vacuo*. The crude dissolved in DMF (400 mL) was added caesium fluoride (CsF, 18.25 g, 120.18 mmol, 1.5 equiv.) and 2-(bromomethyl)naphthalene (NapBr, 21.26 g, 14.78 mmol, 1.2 equiv.) at room temperature under nitrogen atmosphere, and the suspension was heated to 90 °C. After stirring for 18 hours, the mixture was extracted with EtOAc, and the resulting solution was washed with H_2O and brine. The organic layer was dried over Na_2SO_4, filtered, and concentrated. To a solution of the crude in anhydrous THF (400 mL) was added triethylamine (Et_3N, 22.3 mL, 160.24 mmol, 2.0 equiv.), benzoic anhydride (Bz_2O, 36.25 g, 160.24 mmol, 2.0 equiv.) and 4-dimethylaminopyridine (DMAP, 979 mg, 8.01 mmol, 0.1 equiv.) at 0 °C under nitrogen atmosphere, and the suspension was warmed to 60 °C. After stirring for twelve hours, the suspension was concentrated *in vacuo*. The residue was extracted with EtOAc, and the resulting solution was washed with 1N $HCl_{(aq)}$, saturated $NaHCO_{3(aq)}$ and brine. The organic layer was dried over Na_2SO_4, filtered, concentrated, and purified by flash column chromatography (SiO_2, Hex/EtOAc = 2:1) to obtain compound **S9** (36.50 g, 74%).

^{1}H NMR (600 MHz, $CDCl_3$): δ 8.06 – 8.02 (m, 2H), 7.74 (dd, *J* = 8.0, 1.5 Hz, 1H), 7.63 – 7.59 (m, 2H), 7.58 – 7.55 (m, 2H), 7.50 – 7.39 (m, 8H), 7.39 – 7.35 (m, 3H), 7.29 (dd, *J* = 8.4, 1.7 Hz, 1H), 7.05 (d, *J* = 7.9 Hz, 2H), 5.54 (t, *J* = 9.7 Hz, 1H), 5.48 (s, 1H), 4.79 (d, *J* = 12.9 Hz, 1H), 4.74 (d, *J* = 9.8 Hz, 1H), 4.71 (d, *J* = 13.0 Hz, 1H), 4.37 (dd, *J* = 12.2, 1.6 Hz, 1H), 4.26 (dd, *J* = 3.4, 1.1 Hz, 1H), 4.00 (dd, *J* = 12.2, 1.7 Hz, 1H), 3.80 (dd, *J* = 9.6, 3.3 Hz, 1H), 3.46 (q, *J* = 1.5 Hz, 1H), 2.32 (s, 3H).

^{13}C NMR (151 MHz, $CDCl_3$): δ 165.1, 138.3, 137.8, 135.4, 134.6, 133.2, 133.1, 133.1, 130.4, 130.0, 129.6, 129.2, 128.5, 128.3, 128.2, 127.9, 127.8, 127.6, 126.8, 126.6, 126.2, 126.0, 125.8, 101.5, 85.6, 78.3, 73.3, 71.2, 70.2, 69.5, 69.2, 21.4.

HRMS (QToF): Calcd for $C_{38}H_{34}NaO_6S$ $[M + Na]^+$ 641.1974; found 641.1990

^{1}H-NMR (600 MHz, $CDCl_3$)

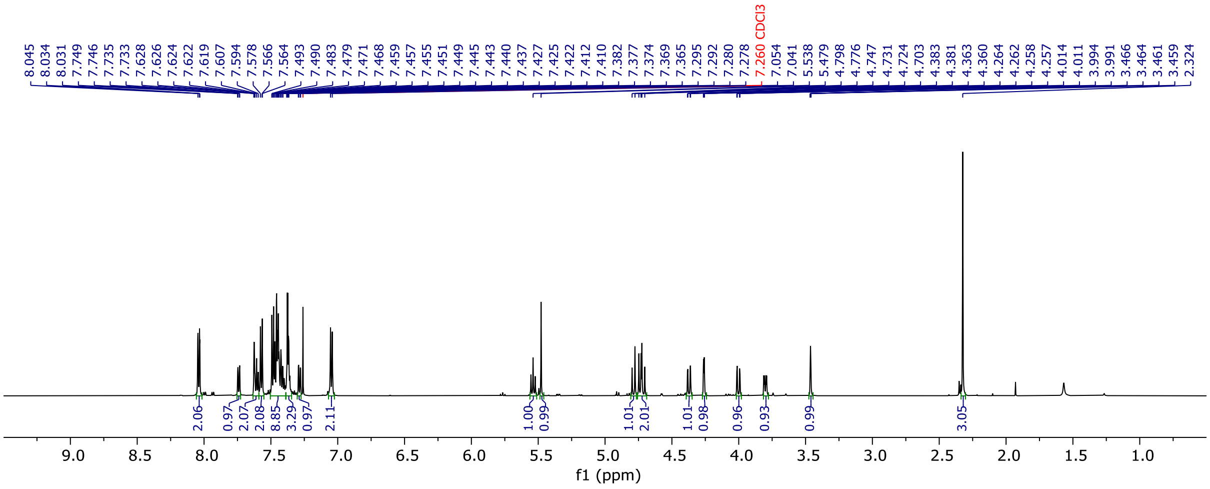

^{13}C-NMR (151 MHz, $CDCl_3$)

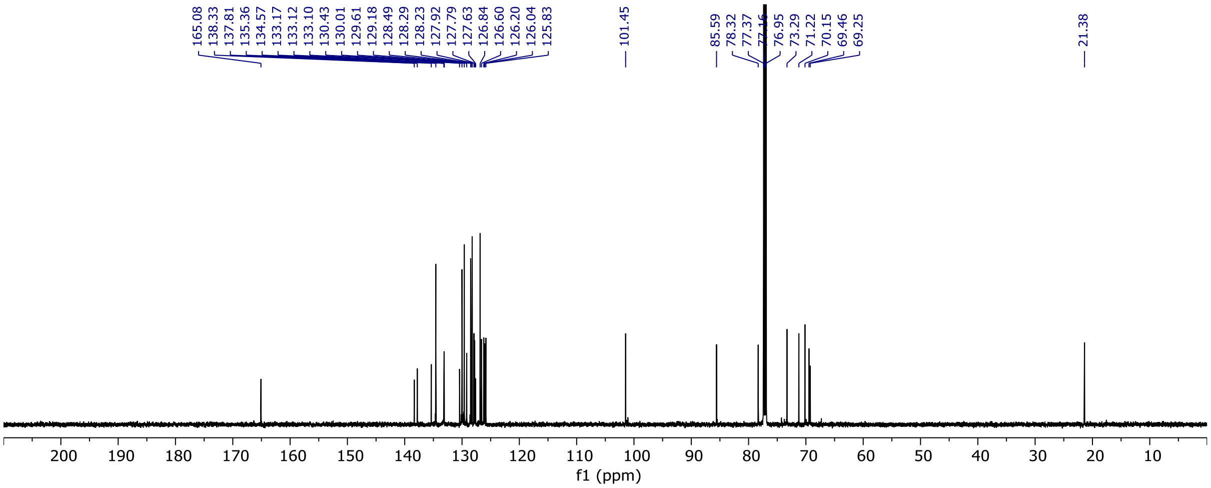

***p*-Methylphenyl 2-*O*-benzoyl-6-*O*-benzyl-1-thio-β-D-galatopyranoside (Compound S10)**

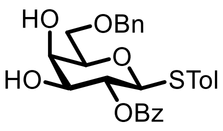

S10

To a solution of compound **S9** (21.00 g, 33.94 mmol, 1.0 equiv.) in CH_2Cl_2/H_2O (170 mL/17 mL) was added 2,3-dichloro-5,6-dicyano-1,4-benzoquinone (DDQ, 11.56 g, 50.91 mmol, 1.5 equiv.) at 0 °C. After stirring for one hour, the mixture was quenched by addition of saturated $Na_2S_2O_{3(aq)}$. The residue was extracted with CH_2Cl_2, and the resulting solution was washed with saturated $NaHCO_{3(aq)}$ and brine. The organic layer was dried over Na_2SO_4, filtered, and concentrated. To a solution of the crude in anhydrous CH_2Cl_2 (170 mL) was added triethylsilane (Et_3SiH, 27.1 mL, 169.70 mmol, 5.0 equiv.), trifluoroacetic anhydride (TFAA, 4.7 mL, 33.94 mmol, 1.0 equiv.) and trifluoroacetic acid (TFA, 13.0 mL, 169.70 mmol, 5.0 equiv.) at 0 °C under nitrogen atmosphere, and the suspension was warmed to room temperature. After stirring for one hour, the mixture was quenched by addition of saturated $NaHCO_{3(aq)}$ at 0 °C. The residue was extracted with CH_2Cl_2, and the resulting solution was washed with H_2O and brine. The organic layer was dried over Na_2SO_4, filtered, concentrated, and purified by flash column chromatography (SiO_2, Hex/EtOAc = 1:1) to obtain compound **S10** (9.53 g, 58%).

^{1}H NMR (600 MHz, $CDCl_3$): δ 8.11 – 8.05 (m, 2H), 7.62 – 7.56 (m, 1H), 7.49 – 7.43 (m, 2H), 7.40 – 7.29 (m, 7H), 7.04 (d, *J* = 8.0 Hz, 2H), 5.20 (t, *J* = 9.6 Hz, 1H), 4.74 (d, *J* = 9.9 Hz, 1H), 4.59 (s, 2H), 4.09 (dd, *J* = 3.4, 1.1 Hz, 1H), 3.84 (d, *J* = 5.3 Hz, 2H), 3.79 (dd, *J* = 9.3, 3.3 Hz, 1H), 3.72 (td, *J* = 5.3, 1.1 Hz, 1H), 2.31 (s, 3H).

^{13}C NMR (151 MHz, $CDCl_3$): δ 167.0, 138.3, 137.9, 133.5, 133.3, 130.2, 129.8, 129.7, 128.8, 128.6, 128.5, 128.0, 127.9, 86.4, 77.5, 74.1, 73.9, 72.3, 69.9, 69.8, 21.3.

HRMS (QToF): Calcd for $C_{27}H_{28}NaO_6S$ $[M + Na]^+$ 503.1504; found 503.1502

^{1}H-NMR (600 MHz, $CDCl_3$)

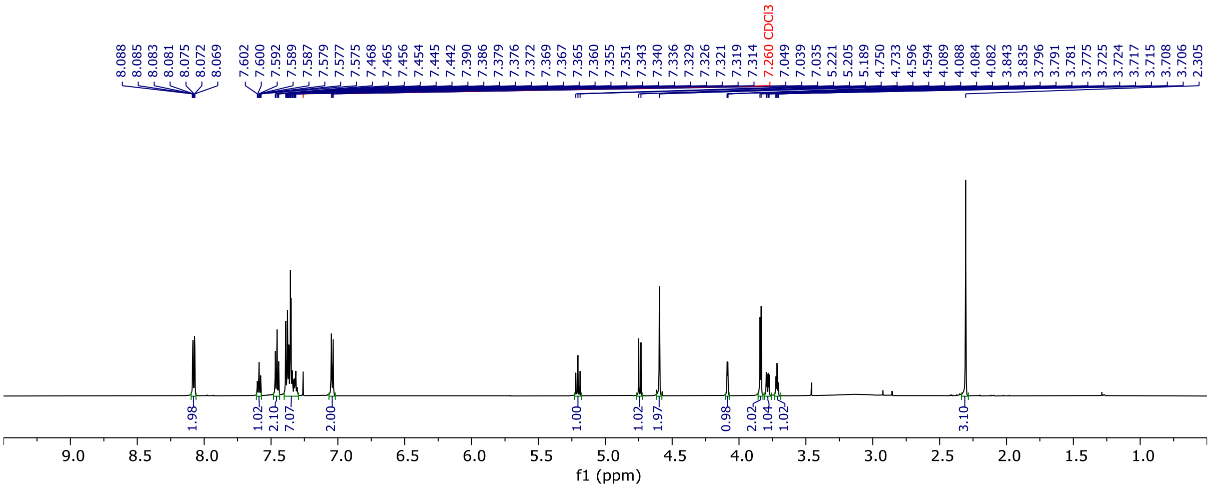

^{13}C-NMR (151 MHz, $CDCl_3$)

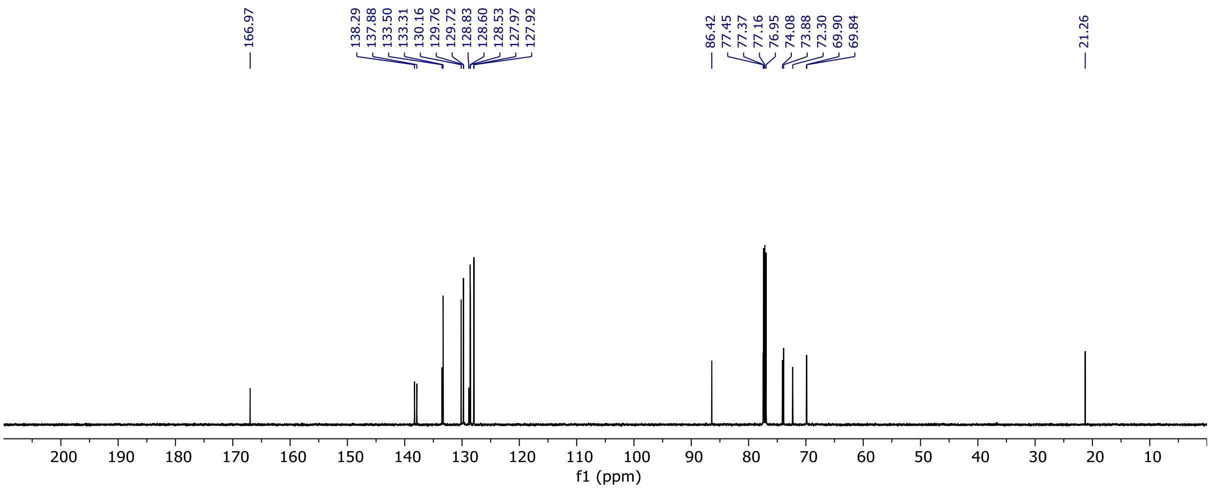

_p_-Methylphenyl 2-_O_-benzoyl-6-_O_-benzyl-3,4-di-O-fluorenylmethoxycarbonyl-1-thio-β-D-galatopyranoside (Compound 3c)

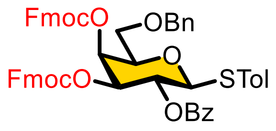

3c

To a solution of compound **S10** (5.00 g, 10.40 mmol, 1.0 equiv.) in anhydrous CH_2Cl_2 (50 mL) was added pyridine (3.4 mL, 41.62 mmol, 4.0 equiv.), fluorenylmethyloxycarbonyl chloride (FmocCl, 10.77 g, 41.62 mmol, 4.0 equiv.) at 0 °C under nitrogen atmosphere, and the suspension was warmed to room temperature. After stirring for twelve hours at room temperature, the suspension was concentrated *in vacuo*. The residue was extracted with EtOAc, and the resulting solution was washed with 1N $HCl_{(aq)}$, saturated $NaHCO_{3(aq)}$ and brine. The organic layer was dried over Na_2SO_4, filtered, concentrated, and purified by flash column chromatography (SiO_2, Toluene/EtOAc = 50:1) to obtain compound **3c** (8.28 g, 86%).

^{1}H NMR (600 MHz, $CDCl_3$): δ 8.08 – 8.04 (m, 2H), 7.75 (dt, *J* = 7.6, 0.9 Hz, 1H), 7.73 (dt, *J* = 7.5, 1.0 Hz, 1H), 7.63 – 7.60 (m, 3H), 7.56 – 7.50 (m, 2H), 7.44 – 7.37 (m, 5H), 7.36 – 7.32 (m, 4H), 7.30 (td, *J* = 7.4, 1.2 Hz, 1H), 7.27 – 7.19 (m, 7H), 7.06 – 7.02 (m, 3H), 6.97 (td, *J* = 7.5, 1.1 Hz, 1H), 5.70 (t, *J* = 10.0 Hz, 1H), 5.56 (dd, *J* = 3.3, 1.0 Hz, 1H), 5.14 (dd, *J* = 10.0, 3.3 Hz, 1H), 4.86 (d, *J* = 10.0 Hz, 1H), 4.51 (d, *J* = 11.8 Hz, 1H), 4.45 (d, *J* = 11.8 Hz, 1H), 4.41 (dd, *J* = 8.1, 5.1 Hz, 1H), 4.29 (dd, *J* = 10.3, 7.0 Hz, 1H), 4.28 – 4.22

(m, 2H), 4.14 (dd, *J* = 10.5, 8.0 Hz, 1H), 4.03 (t, *J* = 7.6 Hz, 1H), 3.97 (td, *J* = 6.4, 1.2 Hz, 1H), 3.73 (dd, *J* = 9.7, 6.2 Hz, 1H), 3.65 (dd, *J* = 9.7, 6.6 Hz, 1H), 2.27 (s, 3H).

^{13}C NMR (151 MHz, $CDCl_3$): δ 165.1, 155.1, 154.3, 143.6, 143.5, 143.2, 143.0, 141.4, 141.4, 141.2, 141.2, 138.4, 137.7, 133.5, 133.1, 130.1, 129.8, 129.6, 129.1, 128.6, 128.6, 128.0, 128.0, 127.9, 127.9, 127.8, 127.5, 127.5, 127.2, 127.2, 125.7, 125.4, 125.3, 125.3, 120.1, 119.9, 87.5, 76.5, 76.1, 73.8, 71.7, 70.6, 70.6, 68.1, 67.9, 46.6, 46.5, 21.3.

HRMS (QToF): Calcd for $C_{57}H_{48}NaO_{10}S$ $[M + Na]^+$ 947.2866; found 947.2900.

^{1}H-NMR (600 MHz, $CDCl_3$)

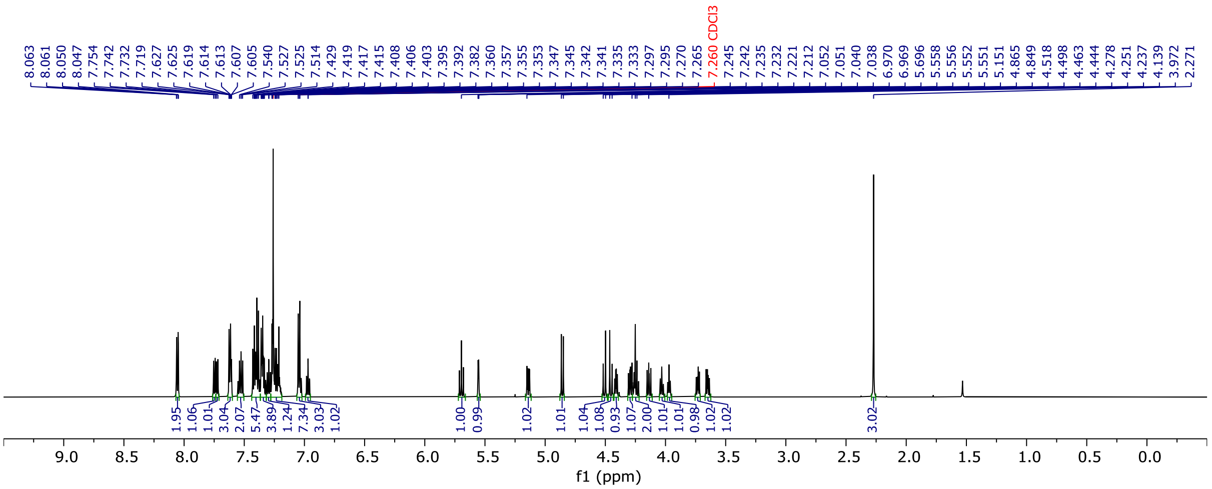

^{13}C-NMR (151 MHz, $CDCl_3$)

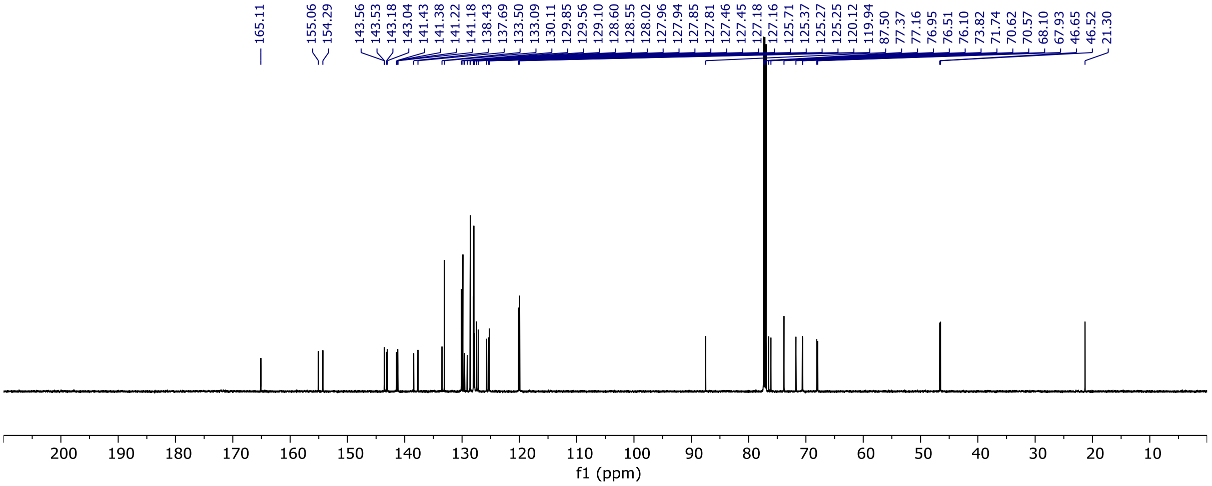

Dibutyl 2-*O*-benzyl-2-*O*-beznyl-3,4-di-*O*-fluorenylmethoxycarbonyl-1-phosphate-β-D-galatopyranoside (Compound 3g)

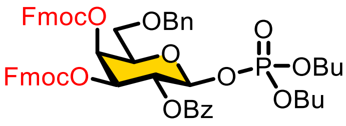

3g

To a solution of compound **3c** (500 mg, 0.54 mmol, 1.0 equiv.), dibutyl phosphate (268 μL, 1.35 mmol, 2.5 equiv.) 4 Å and molecular sieves (500 mg) in anhydrous CH_2Cl_2 (10 mL) was stirred at room temperature for 30 min and cooled to 0 °C. *N*-iodosuccinimide (NIS, 243 mg, 1.08 mmol, 2.0 equiv.) and trifluoromethanesulfonic acid (TfOH, 7.2 μL, 0.08 mmol, 0.15 equiv.) was added at 0 °C under nitrogen atmosphere. After stirring for one hour, the suspension was quenched by addition of pyridine at 0 °C and filtered through a pad of celite. The residue was extracted with EtOAc, and the resulting solution was washed with $Na_2S_2O_{3(aq)}$, H_2O and brine. The organic layer was dried over Na_2SO_4, filtered, concentrated, and purified by flash column chromatography (SiO_2, Hex/EtOAc = 1:2) to obtain compound **3g** (525 mg, 96%).

^{1}H NMR (400 MHz, $CDCl_3$): δ 8.08 (d, *J* = 7.7 Hz, 2H), 7.75 (t, *J* = 8.3 Hz, 2H), 7.67 – 7.59 (m, 3H), 7.59 – 7.50 (m, 2H), 7.48 – 7.15 (m, 15H), 7.03 (t, *J* = 7.5 Hz, 1H), 6.96 (t, *J* = 7.5 Hz, 1H), 5.81 (dd, *J* = 10.5, 8.0 Hz, 1H), 5.59 (d, *J* = 3.3 Hz, 1H), 5.50 (t, *J* = 7.7 Hz, 1H), 5.13 (dd, *J* = 10.6, 3.3 Hz, 1H), 4.54 – 4.40 (m, 3H), 4.37 – 4.23 (m, 3H), 4.18 (dd, *J* = 10.1, 7.8 Hz, 1H), 4.13 – 3.99 (m, 4H), 3.81 – 3.62 (m, 4H), 1.65 – 1.56 (m, 2H), 1.44 – 1.21 (m, 4H), 1.08 – 0.97 (m, 2H), 0.89 (t, *J* = 7.4 Hz, 3H), 0.69 (t, *J* = 7.4 Hz, 3H).

^{13}C NMR (101 MHz, $CDCl_3$): δ 165.0, 155.0, 154.2, 143.5, 143.5, 143.2, 143.0, 141.4, 141.4, 141.2, 141.2, 137.5, 133.7, 130.1, 129.2, 128.7, 128.6, 128.0, 128.0, 127.9, 127.9, 127.8, 127.5, 127.4, 127.2, 127.1, 125.7, 125.3, 125.2, 125.2, 120.1, 120.0, 96.9, 96.9, 77.4, 75.1, 73.8, 72.7, 71.1, 70.7, 70.7, 69.4, 69.3, 68.9, 68.3, 68.2, 68.1, 68.1, 67.0, 53.9, 46.6, 46.5, 32.2, 32.2, 32.1, 31.9, 31.9, 29.4, 18.7, 18.4, 13.7, 13.5.

HRMS (QToF): Calcd for $C_{58}H_{59}NaO_{14}P$ $[M + Na]^+$ 1033.3540; found 1033.3574.

^{1}H-NMR (400 MHz, $CDCl_3$)

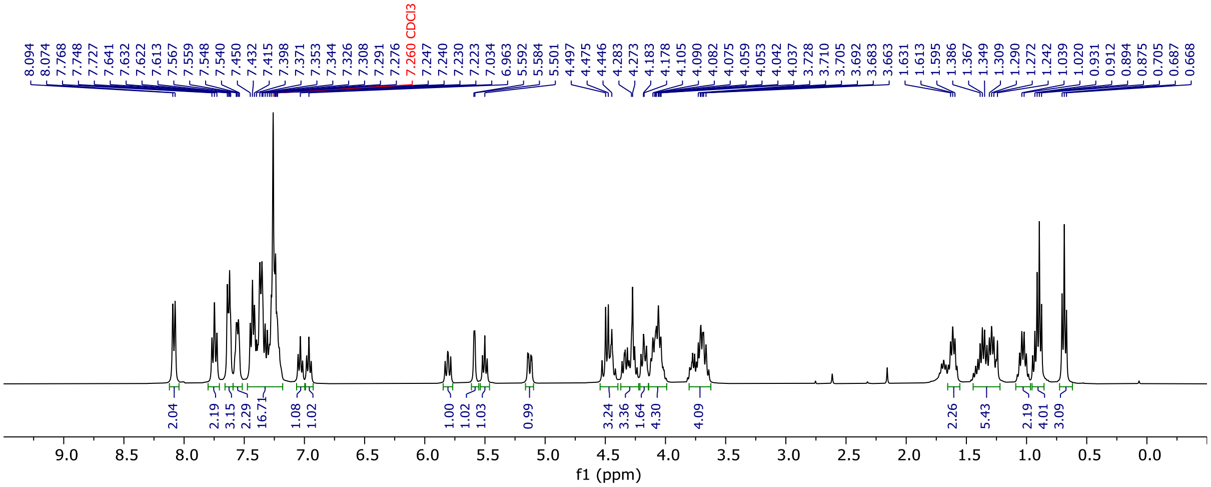

^{13}C-NMR (101 MHz, $CDCl_3$)

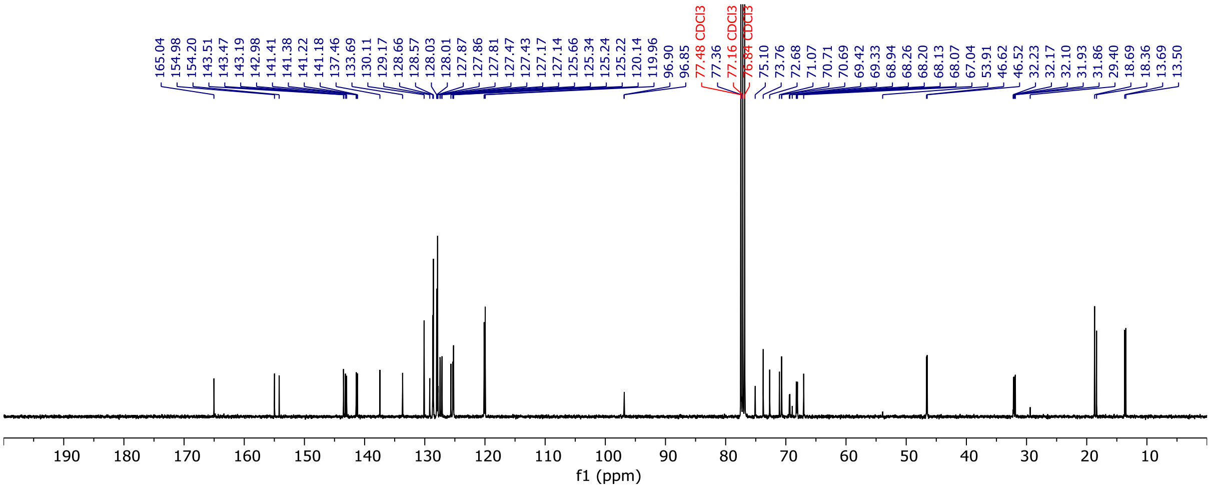

_p_-Methylphenyl 2-_O_-benzoyl-3-_O_-benzyl-1-thio-β-D-galatopyranoside (Compound S11)

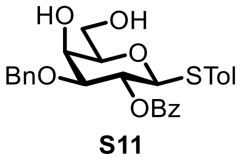

S11

To a solution of compound **S8** (1.00 g, 2.67 mmol, 1.0 equiv.) in anhydrous MeOH (27 mL) was added dibutyltin oxide (Bu_2SnO, 798 mg, 3.21 mmol, 1.2 equiv.) at room temperature under nitrogen atmosphere. After refluxing for three hours, the suspension was concentrated *in vacuo*. The crude dissolved in DMF (27 mL) was added caesium fluoride (CsF, 609 mg, 4.01 mmol, 1.5 equiv.) and benzyl bromide (BnBr, 381 μL, 3.21

mmol, 1.2 equiv.) at room temperature under nitrogen atmosphere, and the suspension was heated to 90 °C. After stirring for 17 hours, the mixture was extracted with EtOAc, and the resulting solution was washed with H_2O and brine. The organic layer was dried over Na_2SO_4, filtered, and concentrated. To a solution of the crude in anhydrous THF (27 mL) was added triethylamine (Et_3N, 745 μL, 5.34 mmol, 2 equiv.), benzoic anhydride (Bz_2O, 1.21 g, 5.34 mmol, 2.0 equiv.) and 4-dimethylaminopyridine (DMAP, 32.6 mg, 0.27 mmol, 0.1 equiv.) at 0 °C under nitrogen atmosphere, and the suspension was warmed to 60 °C. After stirring for twelve hours, the suspension was concentrated *in vacuo*. The residue was extracted with EtOAc, and the resulting solution was washed with 1N $HCl_{(aq)}$, saturated $NaHCO_{3(aq)}$ and brine. The organic layer was dried over Na_2SO_4, filtered, and concentrated. The crude was dissolved in CH_2Cl_2/H_2O (27 mL/1.35 mL) and cooled to 0 °C. Trifluoroacetic acid (2.7 mL) was added to the mixture at 0 °C, and the suspension was warmed to room temperature. After stirring for one hour, the mixture was quenched by addition of saturated $NaHCO_{3(aq)}$ at 0 °C. The residue was extracted with CH_2Cl_2, and the resulting solution was washed with H_2O and brine. The organic layer was dried over Na_2SO_4, filtered, concentrated, and purified by flash column chromatography (SiO_2, Hex/EtOAc = 1:2) to obtain compound **S11** (554 mg, 43%).

^{1}H NMR (600 MHz, $CDCl_3$): δ 8.08 – 7.99 (m, 2H), 7.65 – 7.58 (m, 1H), 7.47 (t, *J* = 7.7 Hz, 2H), 7.38 – 7.30 (m, 2H), 7.22 – 7.12 (m, 5H), 7.07 (d, *J* = 7.9 Hz, 2H), 5.46 (t, *J* = 9.6 Hz, 1H), 4.70 (d, *J* = 10.0 Hz, 1H), 4.65 (d, *J* = 12.2 Hz, 1H), 4.52 (d, *J* = 12.1 Hz, 1H), 4.12 (d, *J* = 3.0 Hz, 1H), 4.03 (dd, *J* = 11.8, 6.9 Hz, 1H), 3.83 (dd, *J* = 11.8, 4.5 Hz, 1H), 3.67 (dd, *J* = 9.3, 3.3 Hz, 1H), 3.58 (t, *J* = 5.7 Hz, 1H), 2.30 (s, 3H).

^{13}C NMR (151 MHz, $CDCl_3$): δ 165.4, 138.3, 137.0, 133.3, 133.2, 130.1, 129.8, 129.1, 128.6, 128.5, 128.2, 128.0, 86.9, 79.4, 78.5, 71.6, 69.9, 67.0, 62.8, 21.3.

HRMS (QToF): Calcd for $C_{27}H_{28}NaO_6S$ $[M + Na]^+$ 503.1504; found 503.1498.

^{1}H-NMR (600 MHz, $CDCl_3$)

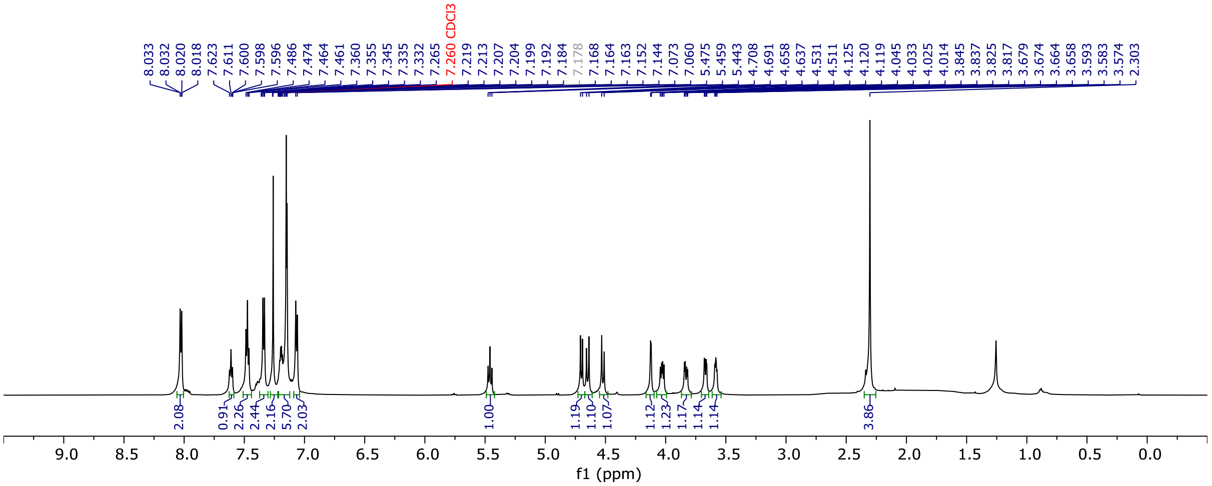

^{13}C-NMR (151 MHz, $CDCl_3$)

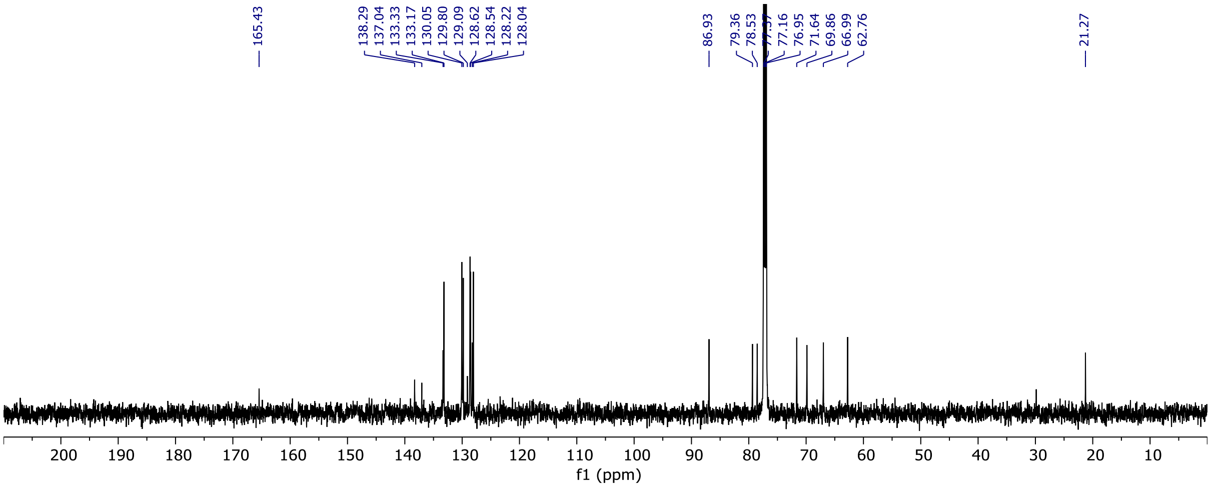

p-Methylphenyl 2-*O*-benzoyl-3-*O*-benzyl-4,6-di-O-fluorenylmethoxycarbonyl-1-thio-β-D-galatopyranoside (Compound 3d)

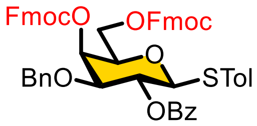

3d

To a solution of compound **S11** (554 mg, 1.15 mmol, 1.0 equiv.) in anhydrous CH_2Cl_2 (12 mL) was added pyridine (464 μL, 5.76 mmol, 5.0 equiv.), fluorenylmethyloxycarbonyl chloride (FmocCl, 895 mg, 3.46 mmol, 3.0 equiv.) at 0 °C under nitrogen atmosphere, and the suspension was warmed to room temperature. After stirring for twelve hours at room temperature, the suspension was concentrated *in vacuo*. The residue was extracted with

EtOAc, and the resulting solution was washed with 1N $HCl_{(aq)}$, saturated $NaHCO_{3(aq)}$ and brine. The organic layer was dried over Na_2SO_4, filtered, concentrated, and purified by flash column chromatography (SiO_2, Toluene/EtOAc = 50:1) to obtain compound **3d** (680 mg, 64%).

^{1}H NMR (600 MHz, $CDCl_3$): δ 8.06 – 7.99 (m, 2H), 7.80 – 7.75 (m, 4H), 7.69 (d, *J* = 7.3 Hz, 1H), 7.65 – 7.60 (m, 4H), 7.50 (t, *J* = 7.8 Hz, 2H), 7.46 – 7.37 (m, 7H), 7.36 – 7.28 (m, 3H), 7.13 – 7.09 (m, 3H), 7.07 (d, *J* = 7.6 Hz, 2H), 7.03 (t, *J* = 7.5 Hz, 2H), 5.60 (t, *J* = 9.7 Hz, 1H), 5.48 (d, *J* = 3.5 Hz, 1H), 4.77 (d, *J* = 10.0 Hz, 1H), 4.69 (d, *J* = 12.4 Hz, 1H), 4.50 – 4.37 (m, 6H), 4.34 – 4.25 (m, 3H), 3.97 (t, *J* = 6.5 Hz, 1H), 3.77 (dd, *J* = 9.6, 3.3 Hz, 1H), 2.27 (s, 3H).

^{13}C NMR (151 MHz, $CDCl_3$): δ 165.2, 155.1, 154.9, 143.7, 143.4, 143.3, 143.3, 141.4, 141.4, 141.3, 141.3, 138.3, 138.3, 137.1, 137.1, 133.3, 133.1, 133.1, 130.1, 129.8, 129.8, 129.3, 128.5, 128.4, 128.1, 128.0, 127.9, 127.5, 127.4, 125.8, 125.5, 125.3, 120.2, 120.0, 87.6, 74.6, 74.6, 71.3, 71.3, 70.6, 70.4, 69.4, 65.8, 46.8, 46.7, 21.3.

HRMS (QToF): Calcd for $C_{57}H_{48}NaO_{10}S$ $[M + Na]^+$ 947.2866; found 947.2916.

^{1}H-NMR (600 MHz, $CDCl_3$)

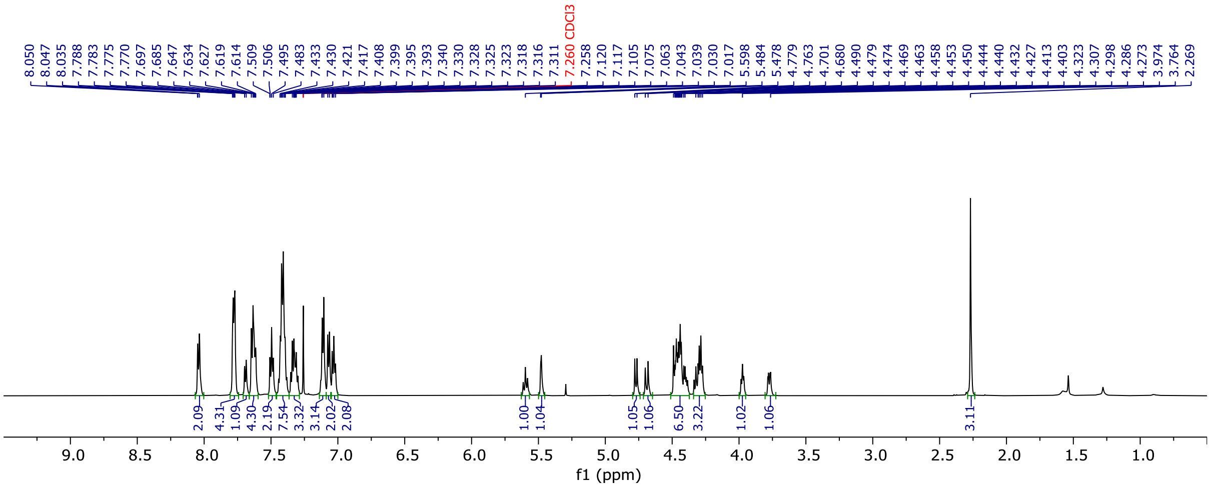

^{13}C-NMR (151 MHz, $CDCl_3$)

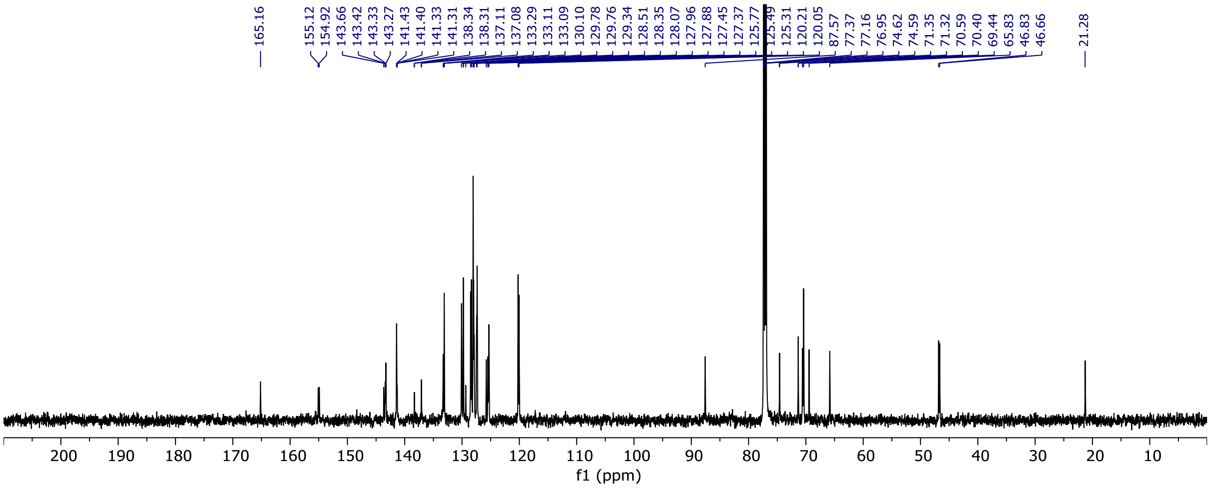

***p*-Methylphenyl 2,3-di-*O*-benzoyl-1-thio-β-D-galatopyranoside (Compound S12)**

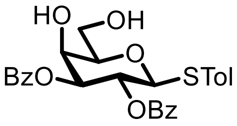

S12

To a solution of compound **S8** (1.00 g, 2.67 mmol, 1.0 equiv.) in anhydrous CH_2Cl_2 (27 mL) was added pyridine (645 μL, 8.01 mmol, 3.0 equiv.), benzoyl chloride (BzCl, 923 μL, 8.01 mmol, 3.0 equiv.) and 4-dimethylaminopyridine (DMAP, 33 mg, 0.27 mmol, 0.1 equiv.) at 0 °C under nitrogen atmosphere, and the suspension was warmed to room temperature. After stirring for three hours at room temperature, the suspension was concentrated *in vacuo*. The residue was extracted with EtOAc, and the resulting solution was washed with 1N $HCl_{(aq)}$, saturated $NaHCO_{3(aq)}$ and brine. The organic layer was dried over Na_2SO_4, filtered, and concentrated. The crude was dissolved in CH_2Cl_2/H_2O (27 mL/1.35 mL) and cooled to 0 °C. Trifluoroacetic acid (2.7 mL) was added to the mixture at 0 °C, and the suspension was warmed to room temperature. After stirring for one hour, the mixture was quenched by addition of saturated $NaHCO_{3(aq)}$ at 0 °C. The residue was extracted with CH_2Cl_2, and the resulting solution was washed with H_2O and brine. The organic layer was dried over Na_2SO_4, filtered, concentrated, and purified by flash column chromatography (SiO_2, Hex/EtOAc = 1:2) to obtain compound **S12** (789 mg, 60%).

^{1}H NMR (600 MHz, $CDCl_3$): δ 7.99 – 7.97 (m, 2H), 7.97 – 7.94 (m, 2H), 7.56 – 7.48 (m, 2H), 7.42 – 7.33 (m, 6H), 7.15 – 7.08 (m, 2H), 5.76 (t, *J* = 9.9 Hz, 1H), 5.31 (dd, *J* = 9.9,

3.0 Hz, 1H), 4.90 (d, *J* = 9.9 Hz, 1H), 4.41 – 4.37 (m, 1H), 4.03 (dd, *J* = 11.9, 5.9 Hz, 1H), 3.93 (dd, *J* = 12.0, 4.2 Hz, 1H), 3.80 (ddd, *J* = 5.6, 4.2, 1.1 Hz, 1H), 2.34 (s, 3H).

^{13}C NMR (151 MHz, $CDCl_3$): δ 166.0, 165.4, 138.7, 133.6, 133.6, 133.4, 130.0, 130.0, 130.0, 129.9, 129.6, 129.1, 128.6, 128.5, 128.4, 87.0, 78.2, 75.7, 68.8, 68.1, 63.1, 21.3.

HRMS (QToF): Calcd for $C_{27}H_{26}NaO_7S$ $[M + Na]^+$ 517.1297; found 517.1293

^{1}H-NMR (600 MHz, $CDCl_3$)

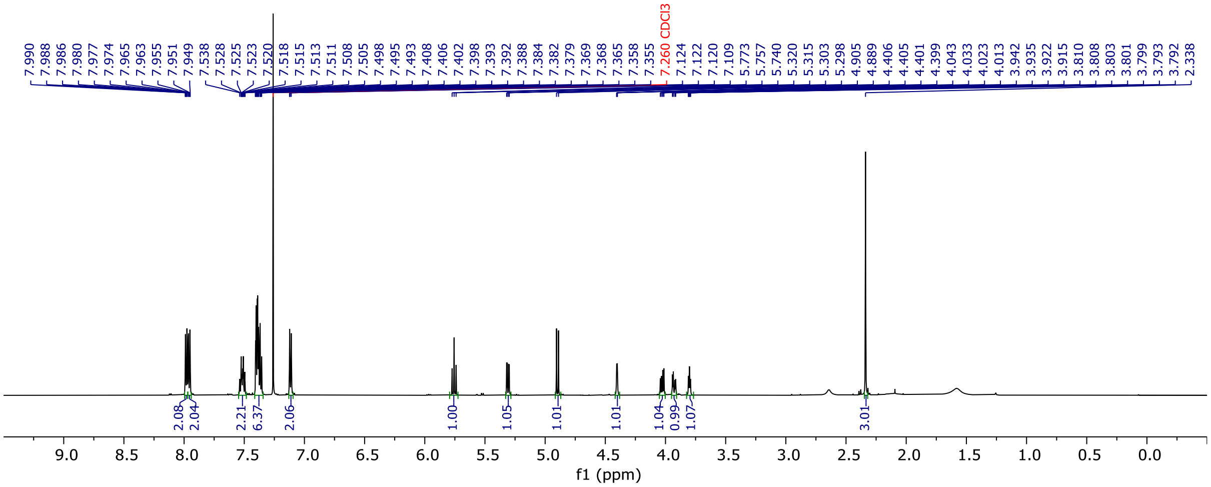

^{13}C-NMR (151 MHz, $CDCl_3$)

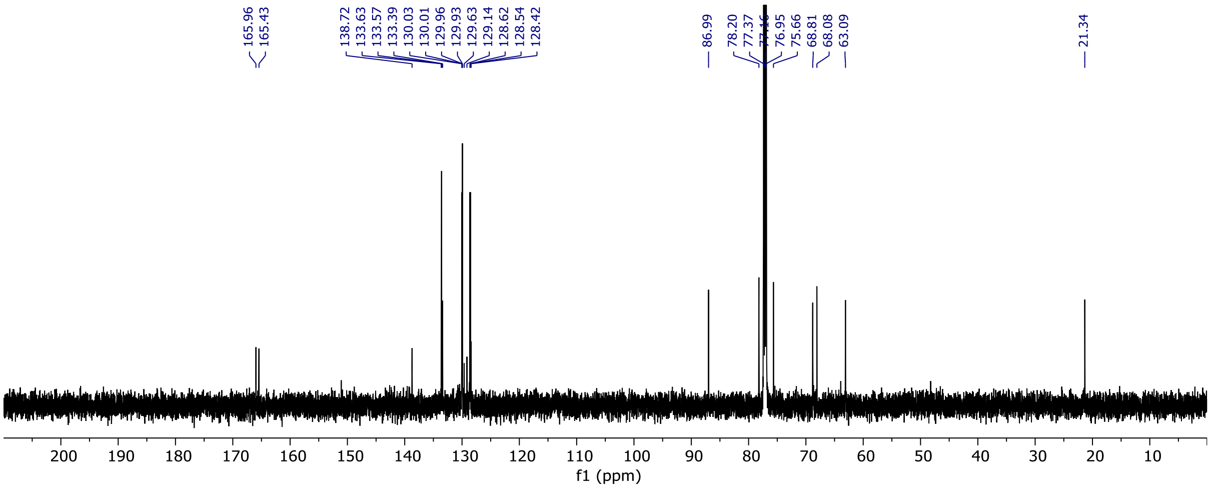

_p_-Methylphenyl 2,3-di-_O_-benzyl-4,6-di-_O_-fluorenylmethoxycarbonyl-1-thio-β-D-galatopyranoside (Compound 3e)

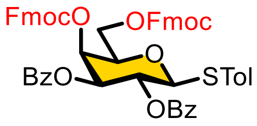

3e

To a solution of compound **S12** (5.00 g, 14.39 mmol, 1.0 equiv.) in anhydrous CH_2Cl_2 (144 mL) was added pyridine (5.7 mL, 71.95 mmol, 5.0 equiv.), fluorenylmethyloxycarbonyl chloride (FmocCl, 11.16 g, 43.17 mmol, 3.0 equiv.) at 0 °C under nitrogen atmosphere, and the suspension was warmed to room temperature. After stirring for twelve hours at room temperature, the suspension was concentrated *in vacuo*. The residue was extracted with EtOAc, and the resulting solution was washed with 1N $HCl_{(aq)}$, saturated $NaHCO_{3(aq)}$ and brine. The organic layer was dried over Na_2SO_4, filtered, concentrated, and purified by flash column chromatography (SiO_2, Toluene/EtOAc = 50:1) to obtain compound **3e** (12.24 g, 91%).

^{1}H NMR (600 MHz, $CDCl_3$): δ 8.02 (tt, *J* = 7.1, 1.5 Hz, 2H), 7.79 (t, *J* = 6.6 Hz, 6H), 7.66 – 7.57 (m, 4H), 7.57 – 7.51 (m, 1H), 7.49 – 7.29 (m, 13H), 7.13 (d, *J* = 7.9 Hz, 2H), 7.09 – 7.03 (m, 2H), 5.86 (t, *J* = 10.0 Hz, 1H), 5.59 – 5.55 (m, 2H), 5.52 (dd, *J* = 10.0, 3.3 Hz, 1H), 4.97 (d, *J* = 10.0 Hz, 1H), 4.51 (dd, *J* = 11.3, 6.9 Hz, 1H), 4.46 – 4.42 (m, 3H), 4.39 (dd, *J* = 11.3, 5.9 Hz, 1H), 4.31 – 4.24 (m, 2H), 4.22 – 4.12 (m, 3H), 2.30 (s, 3H).

^{13}C NMR (151 MHz, $CDCl_3$): δ 165.6, 165.1, 154.9, 154.7, 143.5, 143.4, 143.3, 143.1, 141.4, 141.4, 141.4, 141.3, 138.7, 133.5, 133.5, 133.4, 130.0, 129.9, 129.9, 129.5, 128.8, 128.6, 128.4, 128.1, 127.7, 127.5, 127.4, 127.4, 125.6, 125.4, 125.4, 125.3, 120.2, 120.1, 120.1, 87.3, 74.5, 73.0, 71.7, 70.7, 70.4, 67.7, 65.4, 46.8, 46.6, 21.3.

HRMS (QToF): Calcd for $C_{57}H_{46}NaO_{11}S$ $[M + Na]^+$ 961.2659; found 961.2704.

^{1}H-NMR (600 MHz, $CDCl_3$)

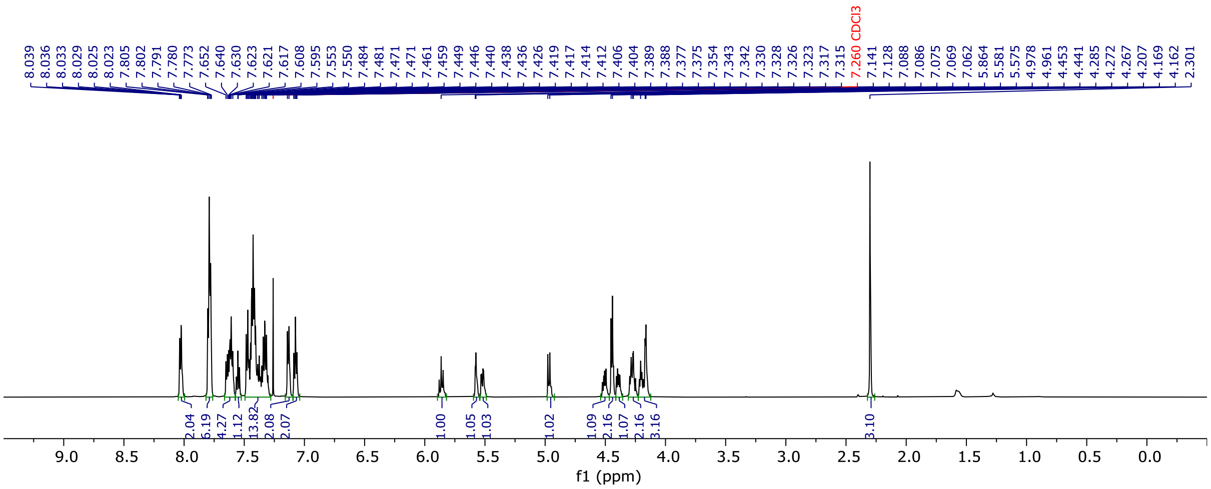

^{13}C-NMR (151 MHz, $CDCl_3$)

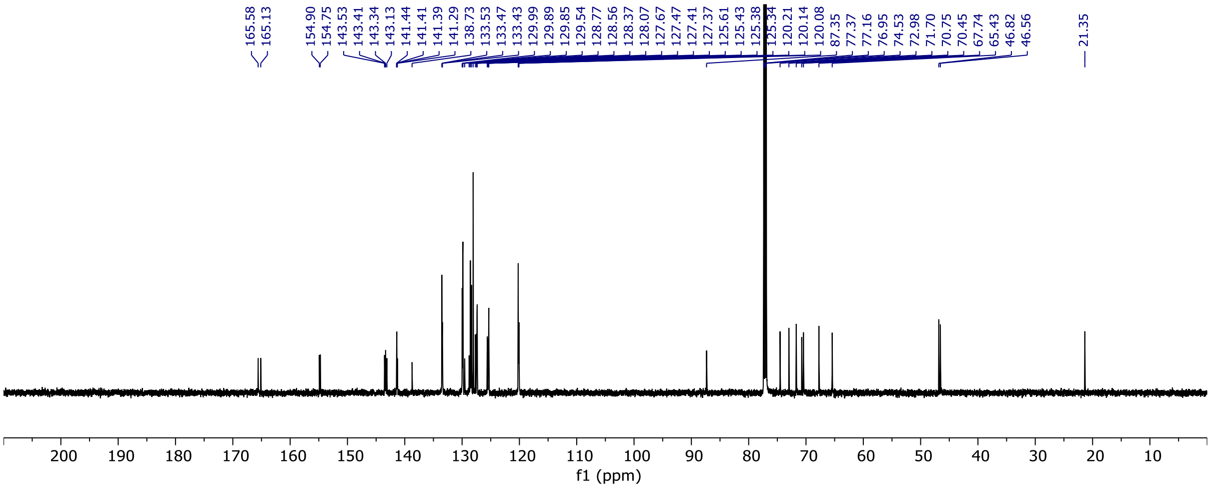

_p_-Methylphenyl 2-_O_-benzoyl-4,6-O-levulinoyl-3-_O_-(2-naphthalenylmethyl)-1-thio-β-D-galatopyranoside (Compound S13)

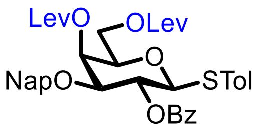

S13

To a solution of compound **S9** (2.00 g, 3.23 mmol, 1.0 equiv.) in CH_2Cl_2/H_2O (32 mL/1.6 mL) was added trifluoroacetic acid (3.2 mL) at 0 °C, and the suspension was warmed to room temperature. After stirring for one hour, the mixture was quenched by addition of saturated $NaHCO_{3(aq)}$ at 0 °C. The residue was extracted with CH_2Cl_2, and the resulting solution was washed with H_2O and brine. The organic layer was dried over Na_2SO_4, filtered, and concentrated. To a solution of the crude in anhydrous CH_2Cl_2 (32 mL) was added levulinic acid (LevOH, 1.3 mL, 12.93 mmol, 4.0 equiv.), 1-ethyl-3-(3-dimethylaminopropyl)carbodiimide hydrochloride (EDC·HCl, 2.48 g, 12.93 mmol, 4.0 equiv.) and 4-dimethylaminopyridine (DMAP, 197 mg, 1.62 mmol, 0.5 equiv.) at 0 °C under nitrogen atmosphere, and the suspension was warmed to room temperature. After stirring for twelve hours, the suspension was concentrated *in vacuo*. The residue was extracted with EtOAc, and the resulting solution was washed with 1N $HCl_{(aq)}$, saturated $NaHCO_{3(aq)}$ and brine. The organic layer was dried over Na_2SO_4, filtered, concentrated, and purified by flash column chromatography (SiO_2, Hex/EtOAc = 1:2) to obtain compound **S13** (1.93 g, 82%).

^{1}H NMR (700 MHz, $CDCl_3$): δ 7.94 (dd, *J* = 8.0, 1.4 Hz, 2H), 7.70 (d, *J* = 8.0 Hz, 1H), 7.63 – 7.58 (m, 2H), 7.53 (d, *J* = 8.0 Hz, 1H), 7.47 – 7.37 (m, 5H), 7.36 – 7.31 (m, 2H), 7.17 (dd, *J* = 8.4, 1.7 Hz, 1H), 7.06 (d, *J* = 7.9 Hz, 2H), 5.64 (d, *J* = 2.9 Hz, 1H), 5.38 (t, *J* = 9.8 Hz, 1H), 4.75 (d, *J* = 12.7 Hz, 1H), 4.67 (d, *J* = 10.1 Hz, 1H), 4.55 (d, *J* = 12.7 Hz, 1H), 4.30 (dd, *J* = 11.3, 6.9 Hz, 1H), 4.17 (dd, *J* = 11.3, 6.4 Hz, 1H), 3.86 (t, *J* = 6.7 Hz, 1H), 3.71 (dd, *J* = 9.5, 3.3 Hz, 1H), 2.84 – 2.52 (m, 8H), 2.30 (s, 3H), 2.21 (s, 3H), 2.18 (s, 3H).
^{13}C NMR (176 MHz, $CDCl_3$): δ 206.8, 206.4, 172.5, 172.2, 165.3, 138.3, 134.7, 133.3, 133.3, 133.1, 133.1, 130.1, 129.9, 129.7, 129.0, 128.5, 128.3, 128.0, 127.7, 127.2, 126.2, 126.1, 126.0, 87.0, 77.0, 74.5, 71.0, 69.5, 66.2, 62.2, 38.2, 38.1, 30.0, 29.9, 28.3, 28.0, 21.3.
HRMS (QToF): Calcd for $C_{41}H_{42}NaO_{10}S$ $[M + Na]^+$ 749.2396; found 749.2426.

^{1}H-NMR (700 MHz, $CDCl_3$)

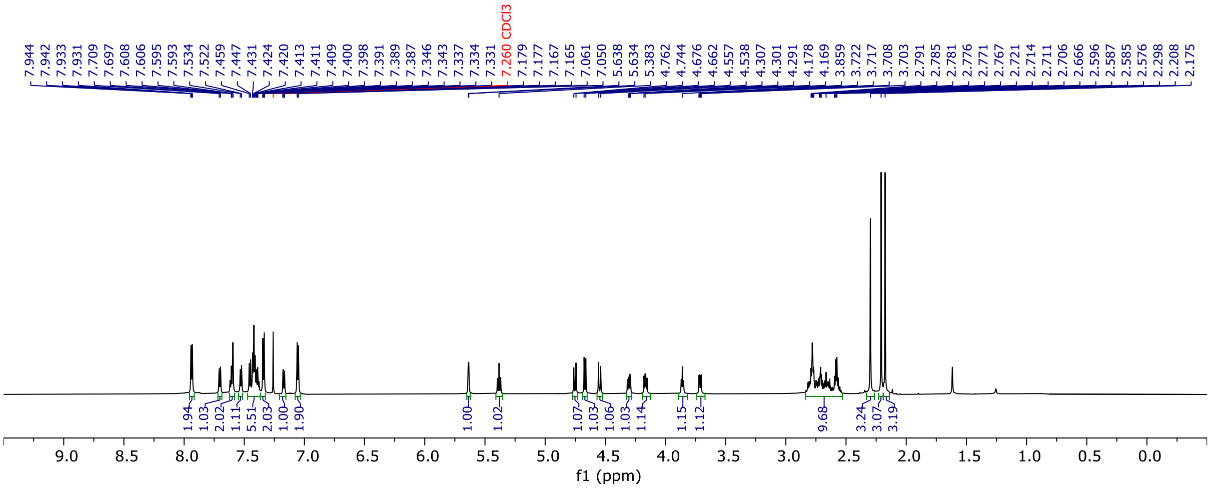

^{13}C-NMR (176 MHz, $CDCl_3$)

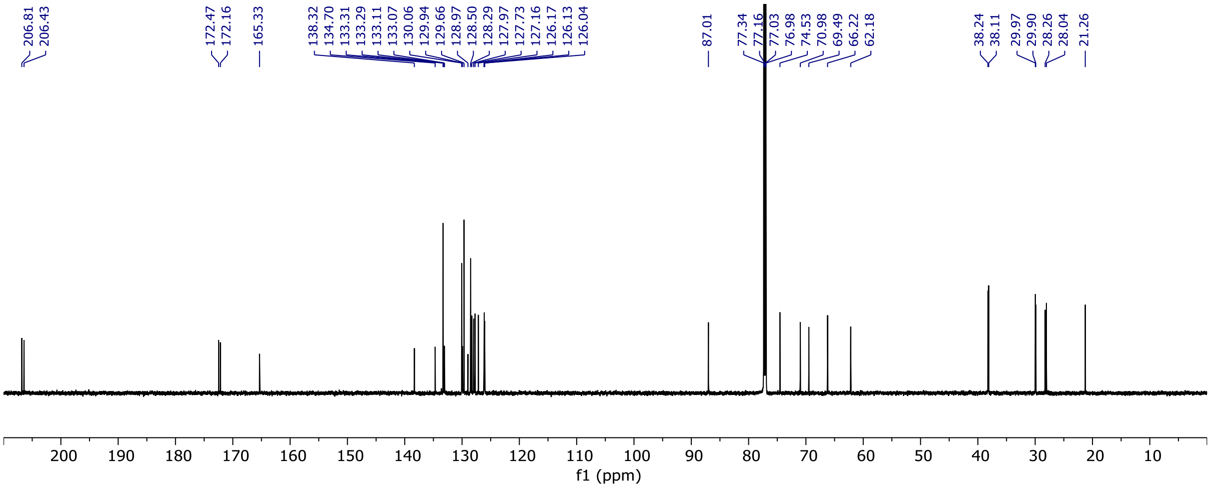

_p_-Methylphenyl 2-_O_-benzoyl-4,6-O-levulinoyl-3-_O_-fluorenylmethoxycarbonyl 1-thio-β-D-galatopyranoside (Compound 3f)

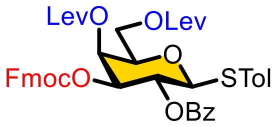

3f

To a solution of compound **S13** (1.91 g, 2.68 mmol, 1.0 equiv.) in CH_2Cl_2/H_2O (27 mL/2.7 mL) was added 2,3-dichloro-5,6-dicyano-1,4-benzoquinone (DDQ, 1.22 g, 5.35 mmol, 2.0 equiv.) at 0 °C. After stirring for one hour, the mixture was quenched by addition of saturated $Na_2S_2O_{3(aq)}$. The residue was extracted with CH_2Cl_2, and the resulting solution was washed with saturated $NaHCO_{3(aq)}$ and brine. The organic layer was dried over Na_2SO_4, filtered, and concentrated. To a solution of the crude in anhydrous CH_2Cl_2 (27 mL) was added pyridine (863 μL, 10.71 mmol, 4.0 equiv.), fluorenylmethyloxycarbonyl chloride (FmocCl, 1.38 g, 5.35 mmol, 2.0 equiv.) at 0 °C under nitrogen atmosphere, and the suspension was warmed to room temperature. After stirring for twelve hours at room temperature, the suspension was concentrated *in vacuo*. The residue was extracted with EtOAc, and the resulting solution was washed with 1N $HCl_{(aq)}$, saturated $NaHCO_{3(aq)}$ and brine. The organic layer was dried over Na_2SO_4, filtered, concentrated, and purified by flash column chromatography (SiO_2, Hex/EtOAc = 1:1) to obtain compound **3f** (1.32 g, 62%).

^{1}H NMR (600 MHz, $CDCl_3$): δ 8.07 – 8.03 (m, 2H), 7.71 – 7.68 (m, 2H), 7.59 – 7.56 (m, 1H), 7.47 – 7.42 (m, 4H), 7.40 (d, *J* = 8.1 Hz, 2H), 7.37 – 7.30 (m, 2H), 7.21 (td, *J* = 7.5, 1.1 Hz, 1H), 7.11 (d, *J* = 8.0 Hz, 2H), 7.08 (td, *J* = 7.5, 1.1 Hz, 1H), 5.61 (dd, *J* = 3.4, 1.1 Hz, 1H), 5.53 (t, *J* = 10.0 Hz, 1H), 5.09 (dd, *J* = 10.0, 3.4 Hz, 1H), 4.84 (d, *J* = 10.0 Hz, 1H), 4.34 (dd, *J* = 10.4, 7.2 Hz, 1H), 4.31 (dd, *J* = 11.3, 6.8 Hz, 1H), 4.22 – 4.15 (m, 2H), 4.13 (t, *J* = 7.5 Hz, 1H), 4.01 (td, *J* = 6.7, 1.2 Hz, 1H), 2.82 – 2.63 (m, 6H), 2.57 (t, *J* = 6.4 Hz, 2H), 2.34 (s, 3H), 2.16 (s, 3H).

^{13}C NMR (151 MHz, $CDCl_3$): δ 206.6, 206.0, 172.3, 172.1, 165.2, 154.2, 143.6, 143.1, 141.3, 141.2, 138.7, 133.6, 133.6, 130.1, 129.8, 129.5, 128.6, 128.5, 127.9, 127.9, 127.2, 127.2, 125.3, 120.0, 120.0, 87.0, 76.2, 74.6, 70.6, 68.0, 67.4, 61.8, 46.6, 38.1, 38.0, 29.9, 29.8, 28.1, 27.9, 21.3.

HRMS (QToF): Calcd for $C_{41}H_{42}NaO_{10}S$ $[M + Na]^+$ 831.2451; found 831.2491.

^{1}H-NMR (600 MHz, $CDCl_3$)

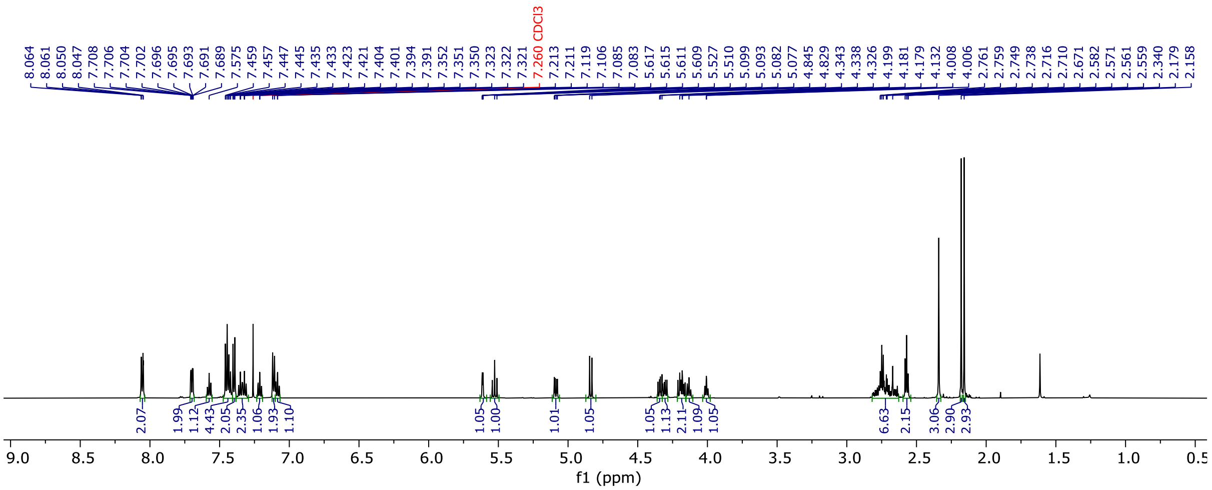

^{13}C-NMR (151 MHz, $CDCl_3$)

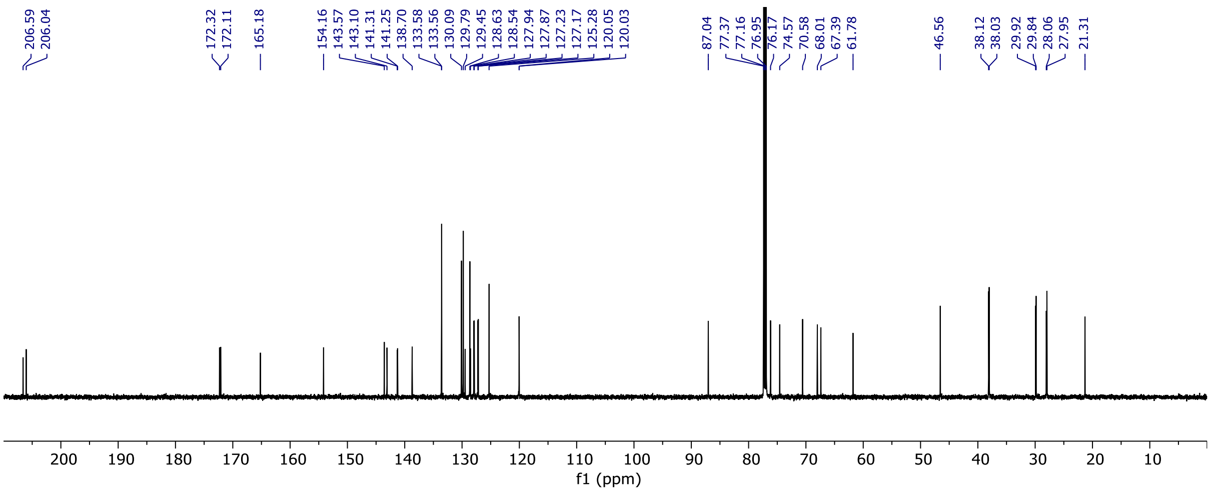

3.3 Glucosamine Building Blocks

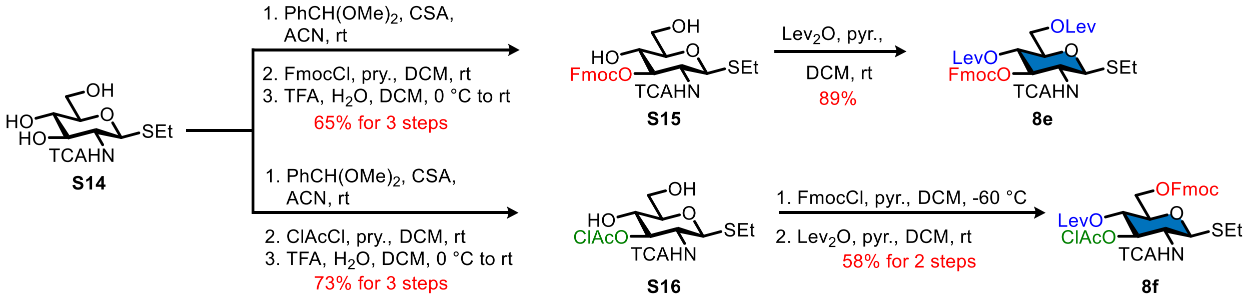

Ethyl 2-deoxy-3-*O*-fluorenylmethoxycarbonyl-1-thio-2-((2,2,2-trichloroacetyl)amino) β-D-glucopyranoside (Compound S15)

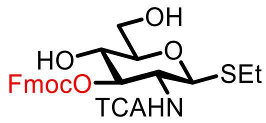

S15

To a solution of compound **S14** (2.00 g, 5.43 mmol, 1.0 equiv.) in MeCN (27 mL) was added benzaldehyde dimethyl acetal ($PhCH(OMe)_2$, 977 µL, 6.51 mmol, 1.2 equiv.) and camphorsulfonic acid (CSA, 378 mg, 1.63 mmol, 0.3 equiv.) at room temperature. After stirring for one hour, the mixture was quenched by addition of Et_3N. The suspension was concentrated *in vacuo*. The residue was extracted with EtOAc, and the resulting solution was washed with H_2O and brine. The organic layer was dried over Na_2SO_4, filtered, and concentrated. The crude was dissolved in anhydrous CH_2Cl_2 (27 mL) and cooled to 0 °C. Pyridine (874 µL, 10.85 mmol, 2.0 equiv.) and fluorenylmethyloxycarbonyl chloride (FmocCl, 2.81 g, 10.85 mmol, 2.0 equiv.) was added to the mixture at 0 °C, and the suspension was warmed to room temperature. After stirring for twelve hours at room temperature, the suspension was concentrated *in vacuo*. The residue was extracted with EtOAc, and the resulting solution was washed with 1N $HCl_{(aq)}$, saturated $NaHCO_{3(aq)}$ and brine. The organic layer was dried over Na_2SO_4, filtered, and concentrated. The crude was dissolved in CH_2Cl_2/H_2O (27 mL/1.35 mL) and cooled to 0 °C. Trifluoroacetic acid (2.7 mL) was added to the mixture at 0 °C, and the suspension was warmed to room temperature. After stirring for one hour, the mixture was quenched by addition of saturated $NaHCO_{3(aq)}$ at 0 °C. The residue was extracted with CH_2Cl_2, and the resulting solution was washed with H_2O and brine. The organic layer was dried over Na_2SO_4, filtered, concentrated, and purified by flash column chromatography (SiO_2, Hex/EtOAc = 1:1) to obtain compound **S15** (2.07 g, 65%).

^{1}H NMR (400 MHz, $CDCl_3$): δ 7.74 (d, *J* = 7.6 Hz, 2H), 7.54 (d, *J* = 7.6 Hz, 2H), 7.39 (t, *J* = 7.5 Hz, 2H), 7.33 – 7.19 (m, 3H), 5.21 (t, *J* = 9.7 Hz, 1H), 4.76 (d, *J* = 10.3 Hz, 1H), 4.36 (d, *J* = 8.0 Hz, 2H), 4.20 (t, *J* = 7.4 Hz, 1H), 4.07 (q, *J* = 9.9 Hz, 1H), 3.97 – 3.75 (m, 3H), 3.61 – 3.55 (m, 1H), 2.67 (q, *J* = 7.3 Hz, 2H), 1.20 (t, *J* = 7.4 Hz, 3H).

^{13}C NMR (101 MHz, $CDCl_3$): δ 162.3, 155.9, 143.0, 142.9, 141.3, 141.3, 128.2, 127.5, 127.4, 125.2, 120.3, 120.3, 92.3, 83.6, 80.1, 79.3, 70.9, 68.9, 62.3, 55.0, 46.5, 24.6, 15.0.

HRMS (QToF): Calcd for $C_{25}H_{26}Cl_3NNaO_7S$ $[M + Na]^+$ 612.0393; found 612.0373.

^{1}H-NMR (400 MHz, $CDCl_3$)

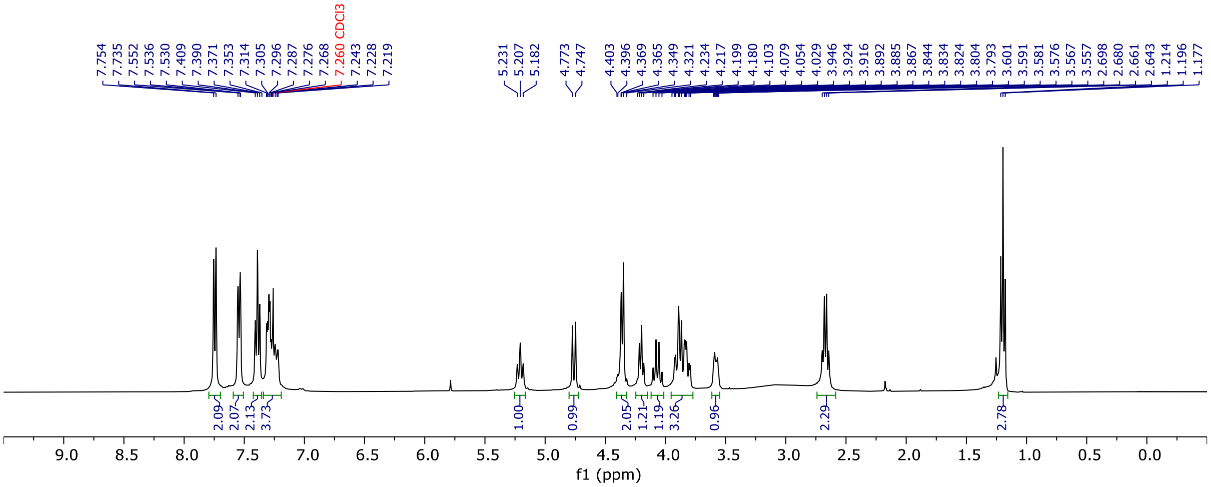

^{13}C-NMR (101 MHz, $CDCl_3$)

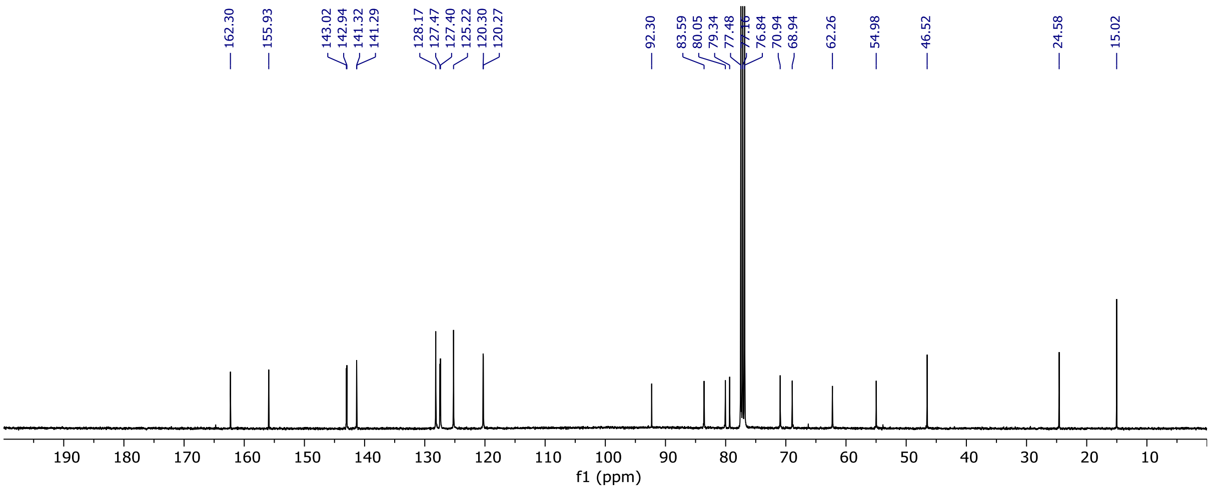

Ethyl 2-deoxy-3-*O*-fluorenylmethoxycarbonyl-4,6-O-levulinoyl-1-thio-2-((2,2,2-trichloroacetyl)amino) β-D-glucopyranoside (Compound 8e)

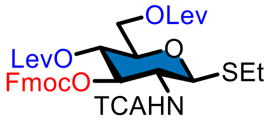

8e

To a solution of levulinic acid (2.8 mL, 27.08 mmol, 8.0 equiv.) in anhydrous CH_2Cl_2 (17 mL) was added *N*,*N'*-Dicyclohexylcarbodiimide (DCC, 2.79 g, 13.54 mmol, 4.0 equiv.) at 0 °C under nitrogen atmosphere, and the suspension was warmed to room temperature.

After stirring for ten minutes at room temperature, the suspension was filtered, and the resulting levulinic anhydride solution was used without further purification[5].

To a solution of compound **S15** (2.00 g, 3.39 mmol, 1.0 equiv.) in anhydrous CH_2Cl_2 (17 mL) was added pyridine (1.1 mL, 13.54 mmol, 4.0 equiv.) and freshly prepared levulinic anhydride solution at 0 °C under nitrogen atmosphere, and the suspension was warmed to room temperature. After stirring for twelve hours at room temperature, the suspension was concentrated *in vacuo*. The residue was extracted with EtOAc, and the resulting solution was washed with 1N $HCl_{(aq)}$, saturated $NaHCO_{3(aq)}$ and brine. The organic layer was dried over Na_2SO_4, filtered, concentrated, and purified by flash column chromatography (SiO_2, Hex/EtOAc = 1:1.5) to obtain compound **8e** (2.36 g, 89%).

^{1}H NMR (400 MHz, $CDCl_3$): δ 7.77 – 7.71 (m, 2H), 7.56 (ddd, *J* = 7.5, 2.0, 1.1 Hz, 2H), 7.39 (td, *J* = 7.5, 1.2 Hz, 2H), 7.32 – 7.26 (m, 2H), 7.12 (d, *J* = 9.1 Hz, 1H), 5.32 (dd, *J* = 10.3, 9.5 Hz, 1H), 5.22 (t, *J* = 9.7 Hz, 1H), 4.83 (d, *J* = 10.3 Hz, 1H), 4.45 (dd, *J* = 9.2, 6.5 Hz, 1H), 4.31 – 4.21 (m, 4H), 4.19 (p, *J* = 9.0 Hz, 1H), 4.20 – 4.08 (m, 1H), 3.84 (ddd, *J* = 10.0, 4.5, 3.0 Hz, 1H), 2.79 – 2.66 (m, 6H), 2.61 – 2.56 (m, 2H), 2.49 – 2.43 (m, 2H), 2.19 (s, 3H), 2.01 (s, 3H), 1.24 (t, *J* = 7.5 Hz, 3H).

^{13}C NMR (101 MHz, $CDCl_3$): δ 206.8, 206.3, 172.5, 171.4, 162.0, 155.2, 143.3, 143.1, 141.3, 141.2, 128.0, 127.4, 127.3, 125.4, 125.4, 120.1, 120.1, 92.2, 83.7, 76.0, 71.1, 68.3, 62.6, 55.1, 46.4, 38.0, 37.9, 30.0, 29.6, 27.9, 27.9, 24.5, 15.0.

HRMS (QToF): Calcd for $C_{35}H_{38}Cl_3NNaO_{11}S$ $[M + Na]^+$ 808.1129; found 808.1159.

^{1}H-NMR (400 MHz, $CDCl_3$)

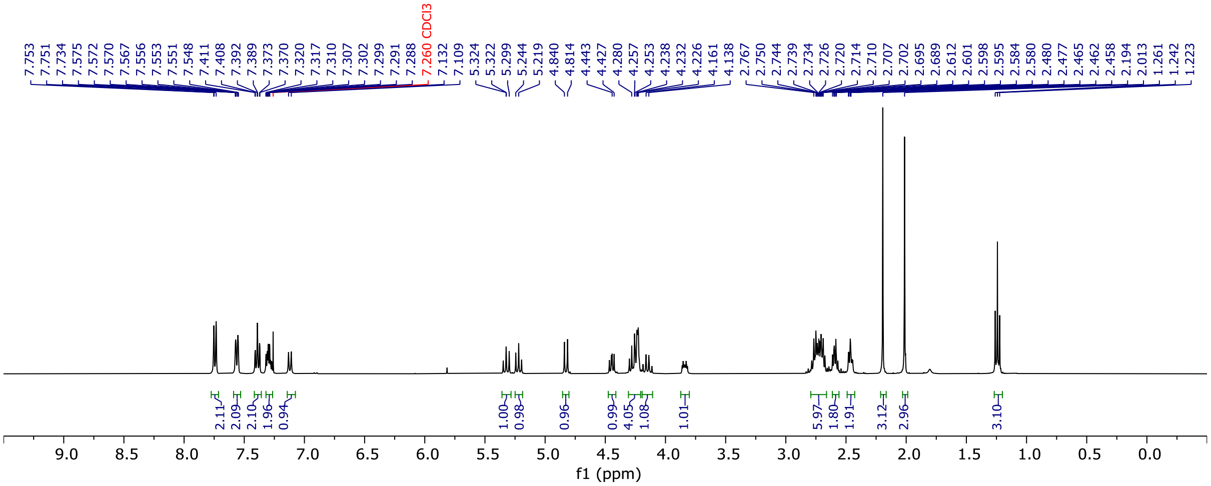

^{13}C-NMR (101 MHz, $CDCl_3$)

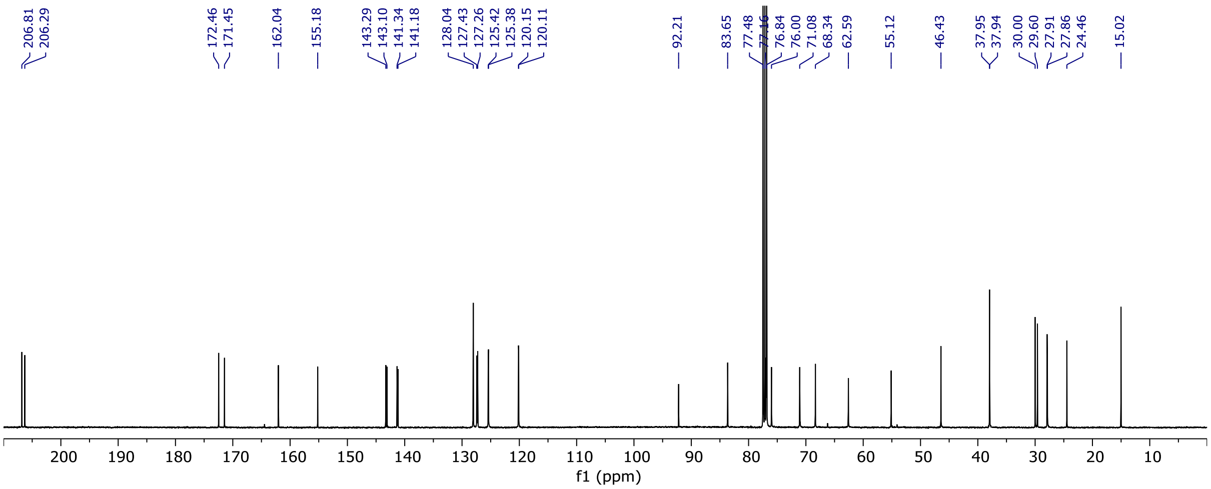

Ethyl 3-*O*-(2-chloroacetyl)-2-deoxy-1-thio-2-((2,2,2-trichloroacetyl)amino) β-D-glucopyranoside (Compound S16)

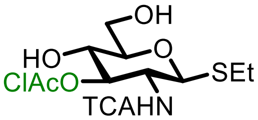

S16

To a solution of compound **S14** (3.00 g, 8.14 mmol, 1.0 equiv.) in MeCN (41 mL) was added benzaldehyde dimethyl acetal ($PhCH(OMe)_2$, 1.5 mL, 9.77 mmol, 1.2 equiv.) and camphorsulfonic acid (CSA, 567 mg, 2.44 mmol, 0.3 equiv.) at room temperature. After stirring for one hour, the mixture was quenched by addition of Et_3N. The suspension was concentrated *in vacuo*. The residue was extracted with EtOAc, and the resulting solution was washed with H_2O and brine. The organic layer was dried over Na_2SO_4, filtered, and concentrated. The crude was dissolved in anhydrous CH_2Cl_2 (41 mL) and cooled to 0 °C. Pyridine (1.3 mL, 16.28 mmol, 2.0 equiv.) and chloroacetyl chloride (ClAcCl, 1.2 mL, 16.28 mmol, 2.0 equiv.) were added to the mixture at 0 °C, and the suspension was warmed to room temperature. After stirring for twelve hours at room temperature, the suspension was concentrated *in vacuo*. The residue was extracted with EtOAc, and the resulting solution was washed with 1N $HCl_{(aq)}$, saturated $NaHCO_{3(aq)}$ and brine. The organic layer was dried over Na_2SO_4, filtered, concentrated. The crude was dissolved in CH_2Cl_2/H_2O (40 mL/2 mL) and cooled to 0 °C. Trifluoroacetic acid (4 mL) was added to the mixture at 0 °C, and the suspension was warmed to room temperature. After stirring for one hour,

the mixture was quenched by addition of saturated $NaHCO_{3(aq)}$ at 0 °C. The residue was extracted with CH_2Cl_2, and the resulting solution was washed with H_2O and brine. The organic layer was dried over Na_2SO_4, filtered, concentrated, and purified by flash column chromatography (SiO_2, Hex/EtOAc = 2:1) to obtain compound **S16** (2.65 g, 73%).

^{1}H NMR (400 MHz, CD_3OD): δ 5.26 (dd, *J* = 10.2, 9.0 Hz, 1H), 4.81 (d, *J* = 10.4 Hz, 1H), 4.24 (d, *J* = 15.3 Hz, 1H), 4.13 (d, *J* = 15.3 Hz, 1H), 3.95 – 3.86 (m, 2H), 3.72 (dd, *J* = 12.1, 5.5 Hz, 1H), 3.60 (dd, *J* = 9.9, 9.1 Hz, 1H), 3.42 (ddd, *J* = 9.9, 5.4, 2.2 Hz, 1H), 2.84 – 2.64 (m, 2H), 1.26 (t, *J* = 7.5 Hz, 3H).

^{13}C NMR (101 MHz, CD_3OD): δ 168.7, 164.0, 93.8, 84.2, 82.0, 79.2, 69.5, 62.4, 56.0, 41.7, 24.9, 15.2.

HRMS (QToF): Calcd for $C_{12}H_{17}Cl_4NNaO_6S$ $[M + Na]^+$ 465.9428; found 465.9422

^{1}H-NMR (400 MHz, CD_3OD)

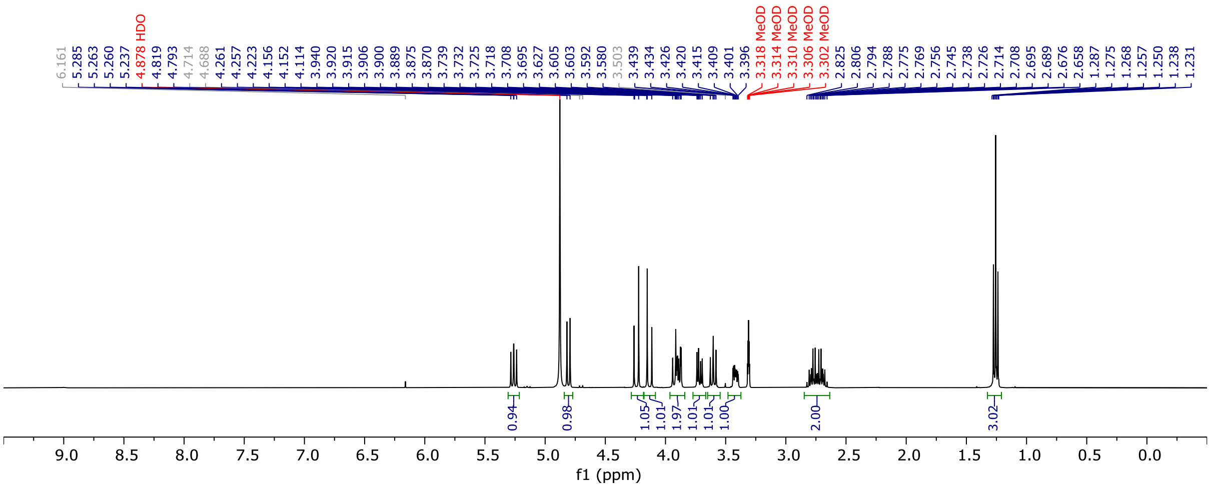

^{13}C-NMR (101 MHz, CD_3OD)

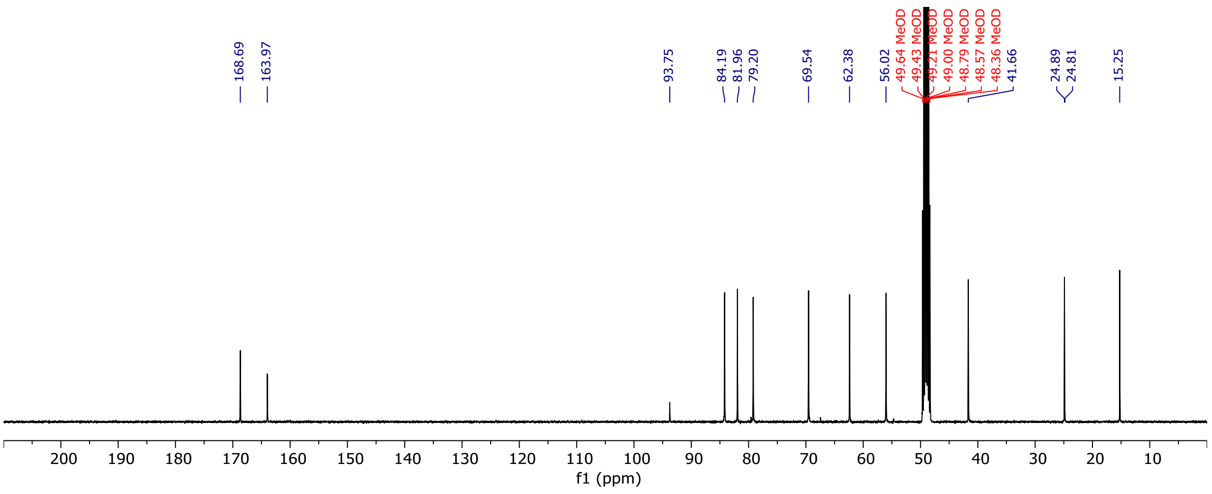

Ethyl 3-*O*-(2-chloroacetyl)-2-deoxy-6-*O*-fluorenylmethoxycarbonyl-4-O-levulinoyl-1-thio-2-((2,2,2-trichloroacetyl)amino) β-D-glucopyranoside (Compound 8f)

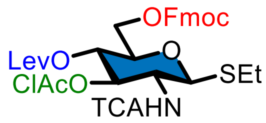

8f

To a solution of compound **S16** (1.20 g, 2.70 mmol, 1.0 equiv.) in anhydrous CH_2Cl_2 (27 mL) was added pyridine (434 μL, 5.39 mmol, 2.0 equiv.) and fluorenylmethyloxycarbonyl chloride (FmocCl, 837 mg, 3.24 mmol, 1.2 equiv.) at -60 °C under nitrogen atmosphere. After stirring for one hour at -60 °C, the suspension was quenched by addition of 1N $HCl_{(aq)}$. The residue was extracted with CH_2Cl_2, and the resulting solution was washed with saturated $NaHCO_{3(aq)}$ and brine. The organic layer was dried over Na_2SO_4, filtered, and concentrated, and purified by flash column chromatography (SiO_2, Hex/EtOAc = 2:1) to obtain **6-O-Fmoc product** (1.21 g, 67%).

Next, to a solution of levulinic acid (1.1 mL, 10.89 mmol, 6 equiv.) in anhydrous CH_2Cl_2 (18 mL) was added *N*,*N'*-dicyclohexylcarbodiimide (DCC, 1.12 g, 5.45 mmol, 3.0 equiv.) at 0 °C under nitrogen atmosphere, and the suspension was warmed to room temperature. After stirring for ten minutes at room temperature, the suspension was filtered, and the resulting levulinic anhydride solution was used without further purification[5].

To a solution of **6-O-Fmoc product** (1.21 g, 1.0 equiv.) in anhydrous CH_2Cl_2 (18 mL) was added pyridine (439 μL, 5.45 mmol, 3.0 equiv.) and freshly prepared levulinic anhydride solution at 0 °C under nitrogen atmosphere, and the suspension was warmed to room temperature. After stirring for twelve hours at room temperature, the suspension was concentrated *in vacuo*. The residue was extracted with EtOAc, and the resulting solution was washed with 1N $HCl_{(aq)}$, saturated $NaHCO_{3(aq)}$ and brine. The organic layer was dried over Na_2SO_4, filtered, concentrated, and purified by flash column chromatography (SiO_2, Hex/EtOAc = 2:1) to obtain compound **8f** (1.21 g, 87%).

^{1}H NMR (600 MHz, $CDCl_3$): δ 7.79 – 7.74 (m, 2H), 7.63 – 7.57 (m, 2H), 7.44 – 7.38 (m, 2H), 7.35 – 7.30 (m, 2H), 7.08 (d, *J* = 9.3 Hz, 1H), 5.52 (dd, *J* = 10.4, 9.4 Hz, 1H), 5.18 (t, *J* = 9.8 Hz, 1H), 4.77 (d, *J* = 10.3 Hz, 1H), 4.46 (dd, *J* = 10.5, 7.1 Hz, 1H), 4.39 (dd, *J* = 10.5, 7.3 Hz, 1H), 4.32 (dd, *J* = 12.0, 5.9 Hz, 1H), 4.29 – 4.21 (m, 3H), 4.16 (td, *J* = 10.3, 9.3 Hz, 1H), 4.07 (d, *J* = 15.2 Hz, 1H), 3.84 (ddd, *J* = 10.1, 5.9, 2.8 Hz, 1H), 2.80 – 2.65

(m, 3H), 2.64 – 2.55 (m, 1H), 2.44 (ddd, J = 16.9, 8.9, 4.0 Hz, 1H), 2.35 (ddd, J = 17.0, 6.8, 4.2 Hz, 1H), 2.08 (s, 3H), 1.25 (t, J = 7.4 Hz, 3H).

^{13}C NMR (151 MHz, $CDCl_3$): δ 206.7, 171.6, 168.3, 162.2, 154.9, 143.5, 143.3, 141.4, 141.4, 128.1, 127.3, 125.3, 125.2, 120.2, 92.2, 83.8, 75.9, 74.6, 70.1, 68.5, 66.3, 54.8, 46.8, 40.9, 38.0, 29.6, 27.9, 24.6, 15.1.

HRMS (QToF): Calcd for $C_{32}H_{33}Cl_4NNaO_{10}S$ $[M + Na]^+$ 786.0477; found 786.0510.

^{1}H-NMR (600 MHz, $CDCl_3$)

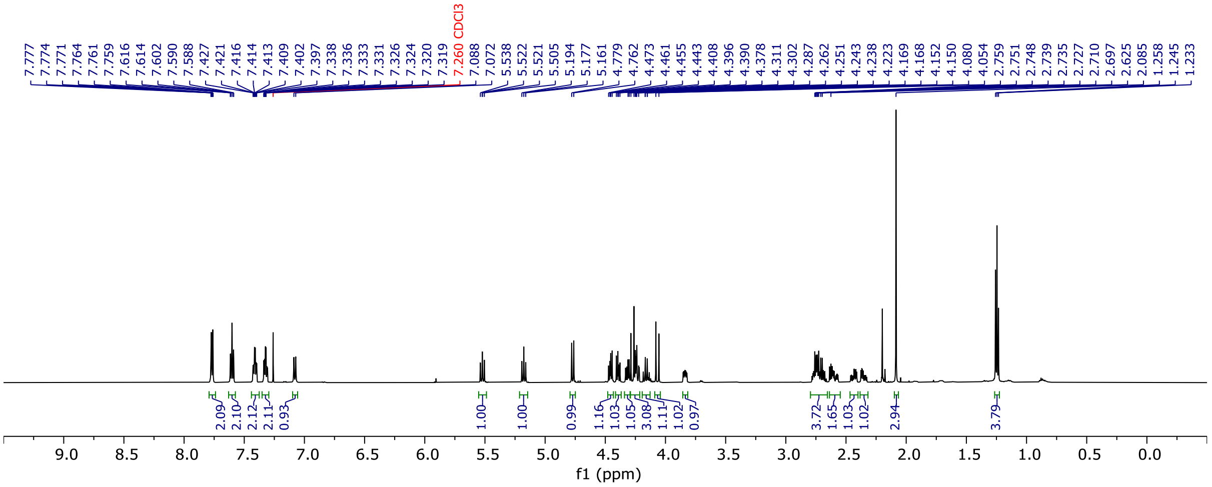

^{13}C-NMR (151 MHz, $CDCl_3$)

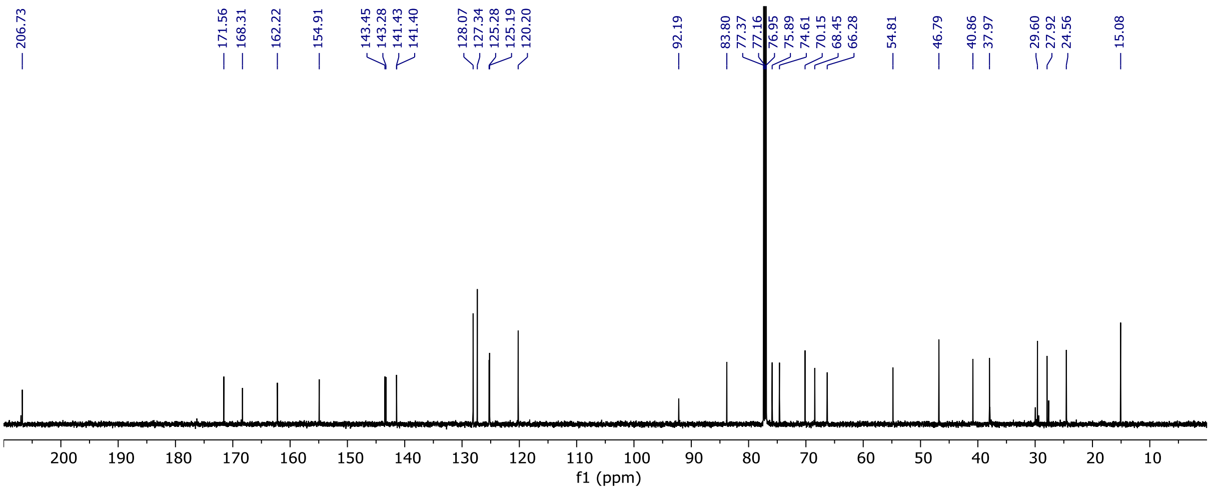

4 Materials and Conditions for Automated Synthesis

4.1 Materials and Measurements

Solvents used for dissolving all building blocks and making of various solutions were taken from Solvent Dispensing System (J.C. Meyer). Wash solvents were HPLC grade. The building blocks were purchased from GlycoUniverse GmbH & Co. KGaA or synthesized if stated. Prior to automated synthesis, the building blocks were weighed and co-evaporated three times with anhydrous toluene and dried overnight under high vacuum prior to use. All solutions were freshly prepared and kept under argon during the automation process. Isolated yields of products were calculated based on resin loading. Functionalized resin was synthesized as previously reported and resin loading (0.33 mmol/g) was determined following a published protocol[6]. Resin was placed in the reaction vessel and was swollen in dichloromethane for 20 minutes at room temperature before starting the first module. During this time, all reagent lines involved in the synthesis were washed and primed.

4.2 Preparation of Stock Solutions

Preparation of anhydrous CH_2Cl_2 (1 ppm): The storage bottle with a capacity of 1 L was dried overnight in the oven. After 24 h, the 4Å molecular sieves were dried using a microwave at 700 W for seven minutes, then cooled down *in vacuo*. The drying and cooling process was repeated three times. Afterward, the 4Å MS were transferred to the storage bottle and cooled down again *in vacuo*. Under argon atmosphere, CH_2Cl_2 from the solvent dispensing system was added to the 1 L storage bottle, and the solvent was left to stand for 24 h. Finally, the water content of CH_2Cl_2 was measured using a KF-Gerat 756 coulometer.

Building Block Solution: Building block was dissolved in 1 mL (per cycle) of anhydrous CH_2Cl_2.

Acidic Wash Solution: TMSOTf (50 µL, 0.28 mmol) was added to 40 mL of anhydrous CH_2Cl_2.

Thioglycoside Activator Solution: Recrystallized NIS (1.45 g, 6.45 mmol) was dissolved in 40 mL of a 4:1 mixture of anhydrous CH_2Cl_2/dioxane, followed by addition of triflic acid (100 μL, 1.11 mmol). The solution was kept under ice-bath cooling for the duration of the automated run.

Phosphate Donor Activator Solution: TMSOTf (450 μL, 2.45 mmol) was added to 40 mL of anhydrous CH_2Cl_2.

Pre-capping Solution: Pyridine (10 mL) was added to 90 mL of DMF.

Capping Solution: Methanesulfonic acid (0.6 mL, 9.24 mmol) and acetic anhydride (6 mL, 63.5 mmol) were added to 50 mL of anhydrous CH_2Cl_2.

Basic Capping Solution: Acetic anhydride (1 mL) and pyridine (1 mL) were added to 2 mL of anhydrous CH_2Cl_2.

Bz Capping Solution: Benzoyl chloride (0.5 mL) and pyridine (0.5 mL) were added to 3 mL of anhydrous CH_2Cl_2.

Lev Deprotection Solution: N_2H_4 HOAc (725 mg, 7.87 mmol) was dissolved in 50 mL of a 4:1:0.25 mixture of pyridine/acetic acid/water.

Fmoc Deprotection Solution: Piperidine (20 mL) was added to 80 mL Dimethylformamide (DMF).

ClAc Deprotection Solution: Thiourea (1.0 g) and pyridine (2 mL) were added to 20 mL 2-methoxyenthanol.

4.3 Modules for Automated Synthesis

Initiation: The resin loaded in the reaction vessel is washed with DMF, THF, and CH_2Cl_2 (3 x 3 mL for 15 s, respectively). The resin is then swollen in 2 mL CH_2Cl_2 for 20 minutes while the temperature of the reaction vessel is cooled to -20 °C.

Acidic Washing: Once the temperature of the reaction vessel has adjusted to -20 °C by the cooling device, 1 mL of the **Acidic Wash Solution** is delivered to the reaction vessel. After three minutes, the solution is drained. Finally, the resin is washed with 3 mL CH_2Cl_2 (bubbling = 15 s) and drained.

Thioglycoside Glycosylation: Upon draining the CH_2Cl_2 in the reaction vessel, 1 mL of **Building Block Solution** containing the appropriate building block is delivered from the building block storing component to the reaction vessel through. After the temperature reaches the desired temperature (T_1), **Thioglycoside Activator Solution** (1 mL) is delivered to the reaction vessel from the respective activator storing component to the reaction vessel. The glycosylation mixture is incubated for the selected duration (t_1) at the desired T_1, then the reaction temperature is linearly ramped to T_2. Once T_2 is reached, it is maintained, and the reaction mixture is incubated for an additional time (t_2). Once the incubation time is finished, the reaction mixture is drained and the resin is washed with CH_2Cl_2 (1 x 3 mL for 15 s), then dioxane (1 x 3 mL for 15 s), and finally CH_2Cl_2 (2 x 3 mL for 15 s).

Phosphate Glycosylation: Upon draining the CH_2Cl_2 in the reaction vessel, 1 mL of **Building Block Solution** containing the appropriate building block is delivered from the building block storing component to the reaction vessel through. After the temperature reaches the desired temperature (T_1), **Phosphate Donor Activator Solution** (1 mL) is delivered to the reaction vessel from the respective activator storing component to the reaction vessel. The glycosylation mixture is incubated for the selected duration (t_1) at the desired T_1, then the reaction temperature is linearly ramped to T_2. Once T_2 is reached, it is maintained, and the reaction mixture is incubated for an additional time (t_2). Once the

incubation time is finished, the reaction mixture is drained, and the resin is washed with CH_2Cl_2 (3 x 3 mL for 15 s).

Pyridine wash: The resin is washed with DMF (2 x 3 mL for 15 s). Then **Pre-capping Solution** (2 mL) is delivered into the reaction vessel and incubated for three minutes. The resin is then washed with CH_2Cl_2 (3 x 3 mL for 15 s).

Capping: The resin is washed with DMF (2 x 3 mL for 15 s). Then **Pre-capping Solution** (2 mL) is delivered into the reaction vessel and incubated for three minutes. The resin is then washed with CH_2Cl_2 (3 x 3 mL for 15 s). Upon washing, **Capping Solution** (4 mL) is delivered, and the temperature is adjusted and maintained 25 °C. The resin and the reagents are incubated for 20 minutes. The solution is then drained from the reactor vessel, and the resin is washed with CH_2Cl_2 (3 x 3 mL for 15 s).

Basic Capping: The resin is washed with DMF (2 x 3 mL for 15 s). Then **Pre-capping Solution** (2 mL) is delivered into the reaction vessel and incubated for three minutes. The resin is then washed with CH_2Cl_2 (3 x 3 mL for 15 s). Upon washing **Basic Capping Solution** (4 mL) is delivered, and the temperature is adjusted and maintained 25 °C. The resin and the reagents are incubated for twelve hours. The solution is then drained from the reactor vessel, and the resin is washed with CH_2Cl_2 (3 x 3 mL for 15 s).

Bz Capping: The resin is washed with DMF (2 x 3 mL for 15 s). Then **Pre-capping Solution** (2 mL) is delivered into the reaction vessel and incubated for three minutes. The resin is then washed with CH_2Cl_2 (3 x 3 mL for 15 s). Upon washing **Bz Capping Solution** (4 mL) is delivered, and the temperature is adjusted and maintained 25 °C. The resin and the reagents are incubated for one hour. The solution is then drained from the reactor vessel, and the resin is washed with CH_2Cl_2 (3 x 3 mL for 15 s).

Fmoc Deprotection: The resin is first washed with DMF (3 x 3 mL for 15 s), and then **Fmoc Deprotection Solution** (2 mL) is delivered to the reaction vessel. After five minutes

the reaction solution is drained and the resin is washed with DMF (3 x 3 mL for 15 s) and CH_2Cl_2 (3 x 3 mL for 15 s).

Lev Deprotection: The resin was washed with DMF (3×30 s) and CH_2Cl_2 (1.3 mL) added to the reaction vessel. **Lev Deprotection Solution** (0.8 mL) was added to the reaction vessel, and the temperature was adjusted to 25 °C. After 30 minutes, the reaction solution was drained, and the entire cycle was repeated twice more. After Lev deprotection was completed, the resin was washed with DMF (3 x 3 mL for 15 s), THF (3 x 3 mL for 15 s) and CH_2Cl_2 (3 x 3 mL for 15 s).

ClAc Deprotection: The resin is first washed with CH_2Cl_2 (3 x 2 mL for 15 s) then **ClAc Deprotection Solution** (2 mL) was delivered to the reaction vessel. The temperature of the reagents inside the reactor vessel is then adjusted to 70 °C. After 20 min, the reaction solution is drained from the reactor vessel. The resin is washed with DMF (3 x 2 mL for 15 s). Then fresh **ClAc Deprotection Solution** (2 mL) is delivered and the process is repeated twice more. Then, the resin is washed with DMF (3 x 3 mL for 15 s) and CH_2Cl_2 (5 x 3 mL for 15 s).

4.4 Post-synthesizer Manipulation

Method A: Cleavage from Solid Support for Protected Oligosaccharides

Vapourtec® E-Series UV-150 Photoreactor Flow Chemistry System was used. The medium pressure mercury lamp was filtered by the commercial long-pass filter (No.3, red), adjusted to 80% power, and maintained at 20 °C by a cold nitrogen flow (dried ice reservoir). The resin was suspended in CH_2Cl_2 (around 10 mL) and loaded into a PTFE syringe. The suspension was pushed by a syringe pump (PHD2000, Harvard Aparatus) at 1 mL/min into the reactor coil (10 mL, 1/8-inch o.d. FEP tubing) inside the UV-chamber. After 15 min, the resin was pushed out by CH_2Cl_2 (2 mL/min). The blackened resin was filtered, and the reactor was washed with CH_2Cl_2 (10 mL). The solvent was evaporated *in vacuo*.

Method B: Capping

To the suspension of the residue form photocleavage in pyridine (2 mL) was added acetic anhydride (2 mL) at room temperature. The suspension was stirred overnight, and the solvent was evaporated *in vacuo*. The residue was subjected to NP-HPLC purification.

Method C: General Deprotection Procedure

To a suspension of protected glycan in AcOH (5 mL) was added Zn/Cu (500 mg) and stirred at 50 °C overnight. The suspension was filtered, and the solvent was evaporated *in vacuo*. Then, the crude compound was dissolved in Ac_2O/pyridine (4 mL, 1:1) and stirred overnight. Upon the completion of acetylation, the solvent was evaporated *in vacuo*. The acetylated crude compound was dissolved in THF/MeOH/H_2O (5 mL, 2:2:1) and added LiOH (12 mg). The mixture was stirred at 40 °C overnight. Amberlite IR-120 (H^+ form) was then added to neutralize. After neutralization, the reaction mixture was filtered, and the solvent was evaporated *in vacuo*. The crude compound was used for hydrogenolysis without further purification. The crude compound was dissolved in ^{t}BuOH:H_2O (3:1, 4 mL) with AcOH (0.1 mL). The suspension was added $Pd(OH)_2$/C (10%) purged with N_2 and H_2 for ten minutes. The suspension was stirred under H_2 balloon overnight. The insoluble material was removed by a CHROMAFIL ®Xtra, RC 0.45 syringe filter. The solid was washed once with MeOH and several times with water. The filtrate was collected and concentrated *in vacuo*.

Method N1: NP-HPLC of Crude Material

Analytical HPLC: Crude products were dissolved in HPLC-grade EtOAc (4 mL). A YMC-Diol-300-NP column (150 mm x 4.60 mm I.D.) was used with a flow rate of 1.00 mL/min and hexane/EtOAc as eluent.

Preparative HPLC: Crude products were dissolved in HPLC-grade EtOAc/hexane 1:1 (2 mL). A YMC-Diol-300-NP column (150 mm x 20 mm I.D.) was used at a flow rate of 15.00 mL/min with hexane/EtOAc as eluent.

Gradient of eluents:

t (min)	EtOAc in hexane (%)
0	20
5	20
35	60
45	100

Method N2: NP-HPLC of Crude Material

Analytical HPLC: Crude products were dissolved in HPLC-grade EtOAc (4 mL). A YMC-Diol-300-NP column (150 mm x 4.60 mm I.D.) was used with a flow rate of 1.00 mL/min and hexane/EtOAc as eluent.

Preparative HPLC: Crude products were dissolved in HPLC-grade EtOAc/hexane 1:1 (2 mL). A YMC-Diol-300-NP column (150 mm x 20 mm I.D.) was used at a flow rate of 15.00 mL/min with hexane/EtOAc as eluent.

Gradient of eluents:

t (min)	EtOAc in hexane (%)
0	20
5	20
35	80
45	100

Method R1: RP-HPLC of Crude Material

Analytical HPLC: Crude products were dissolved in milliQ-grade water (3 mL). A Thermo-Scientific Hypercarb column (150 mm x 4.60 mm I.D.) was used for analytical RP-HPLC with a flow rate of 0.70 mL/min with water (0.1% HCO_2H)/acetonitrile as eluents.

Preparative HPLC: Crude products were dissolved in milliQ-grade water (3 mL). A Thermo-Scientific Hypercarb column (150 mm x 4.60 mm I.D.) was used for analytical RP-HPLC with a flow rate of 3 mL/min with H_2O (0.1% HCO_2H)/acetonitrile as eluents.

Gradient of eluents:

t (min)	MeCN in H_2O (%)
0	0
5	0
35	30
45	100

Method R2: RP-HPLC of Crude Material

Analytical HPLC: Crude products were dissolved in milliQ-grade water (3 mL). A Thermo-Scientific Hypercarb column (150 mm x 4.60 mm I.D.) was used for analytical RP-HPLC with a flow rate of 0.70 mL/min with water (0.1% HCO_2H)/acetonitrile as eluents.

Preparative HPLC: Crude products were dissolved in milliQ-grade water (3 mL). A Thermo-Scientific Hypercarb column (150 mm x 4.60 mm I.D.) was used for analytical RP-HPLC with a flow rate of 3 mL/min with H_2O (0.1% HCO_2H)/acetonitrile as eluents.

Gradient of eluents:

t (min)	MeCN in H_2O (%)
0	0
5	0
35	20
45	100

Method R3: RP-HPLC of Crude Material

Analytical HPLC: Crude products were dissolved in milliQ-grade water (3 mL). A Thermo-Scientific Hypercarb column (150 mm x 4.60 mm I.D.) was used for analytical RP-HPLC with a flow rate of 0.70 mL/min with water (0.1% HCO_2H)/acetonitrile as eluents.

Preparative HPLC: Crude products were dissolved in milliQ-grade water (3 mL). A Thermo-Scientific Hypercarb column (150 mm x 4.60 mm I.D.) was used for analytical RP-HPLC with a flow rate of 3 mL/min with H_2O (0.1% HCO_2H)/acetonitrile as eluents.

Gradient of eluents:

t (min)	MeCN in H_2O (%)
0	0
5	0
35	25
45	100

Method R4: RP-HPLC of Crude Material

Analytical HPLC: Crude products were dissolved in milliQ-grade water (3 mL). A Thermo-Scientific Hypercarb column (150 mm x 4.60 mm I.D.) was used for analytical RP-HPLC with a flow rate of 0.70 mL/min with water (0.1% HCO_2H)/acetonitrile as eluents.

Preparative HPLC: Crude products were dissolved in milliQ-grade water (3 mL). A Thermo-Scientific Hypercarb column (150 mm x 4.60 mm I.D.) was used for analytical RP-HPLC with a flow rate of 3 mL/min with H_2O (0.1% HCO_2H)/acetonitrile as eluents.

Gradient of eluents:

t (min)	MeCN in H_2O (%)
0	0
5	0
35	40
45	100

Method R5: RP-HPLC of Crude Material

Analytical HPLC: Crude products were dissolved in milliQ-grade water (3 mL). A Synergi Hydro RP18 column (250 mm x 2.60 mm I.D.) was used for analytical RP-HPLC with a flow rate of 1.00 mL/min with water (0.1% HCO_2H)/acetonitrile as eluents.

Preparative HPLC: Crude products were dis1solved in milliQ-grade water (3 mL). A Synergi Hydro RP18 column (250 mm x 10 mm I.D.) was used for analytical RP-HPLC with a flow rate of 4 mL/min with H_2O (0.1% HCO_2H)/acetonitrile as eluents.

Gradient of eluents:

t (min)	MeCN in H_2O (%)
0	0
5	0
35	15
45	100

5 Automated Glycan Assembly of Protected Sialylated Glycans

5.1 AGA of Disaccharide **4**

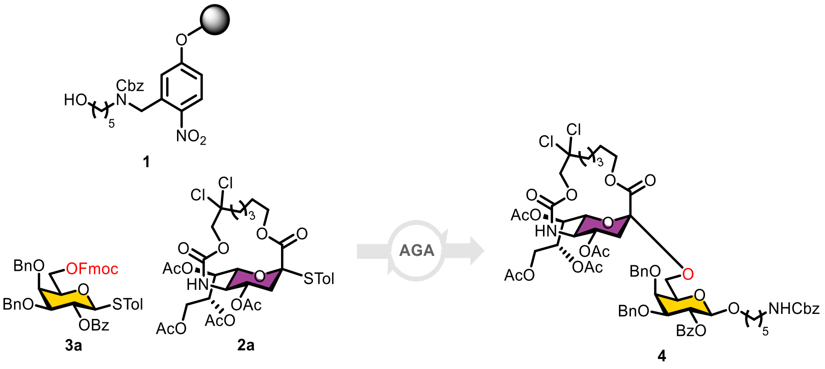

	Building blocks	Modules	Glycosylation condition
AGA		**Initiation** (40 mg resin)	
AGA	Gal 3a	Acidic wash<br>Thioglycoside glycosylation<br>Capping<br>Fmoc deprotection	6.5 eq. / t (min)<br>T_1 = -20 °C / 5<br>T_2 = 0 °C / 20
AGA	Sia 2a	Acidic wash<br>Thioglycoside glycosylation x2<br>Capping	10 eq. / t (min)<br>T_1 = -40 °C / 30<br>T_2 = -20 °C / 10
Post AGA		Photocleavage<br>NP-HPLC Purification	

Protected compound **4** (11.7 mg, 8.89 µmol, 67%) was obtained as a colorless oil by purification using preparative NP-HPLC (**Method N1**).

^{1}**H NMR** (600 MHz, $CDCl_3$, 50 °C): δ 8.02 (dt, J = 8.3, 1.3 Hz, 2H), 7.53 (td, J = 7.4, 1.4 Hz, 1H), 7.44 – 7.36 (m, 4H), 7.36 – 7.32 (m, 4H), 7.29 (dd, J = 8.1, 6.3 Hz, 3H), 7.25 – 7.21 (m, 1H), 7.20 – 7.11 (m, 5H), 5.59 (t, J = 9.1 Hz, 1H), 5.57 – 5.50 (m, 1H), 5.41 – 5.36 (m, 1H), 5.35 – 5.27 (m, 2H), 5.07 (s, 2H), 4.97 (d, J = 11.4 Hz, 1H), 4.75 – 4.56 (m, 5H), 4.54 – 4.40 (m, 3H), 4.36 (dd, J = 12.6, 2.3 Hz, 1H), 4.25 (ddd, J = 12.6, 4.7, 1.1 Hz, 1H), 3.98 (d, J = 2.8 Hz, 1H), 3.95 – 3.89 (m, 1H), 3.85 (dt, J = 9.4, 5.9 Hz, 1H), 3.81 – 3.58 (m, 4H), 3.44 (dt, J = 9.8, 6.8 Hz, 1H), 2.93 (qm, J = 6.7 Hz, 2H), 2.87 (dd, J = 13.0, 4.7 Hz, 1H), 2.71 (td, J = 10.5, 6.5 Hz, 1H), 2.42 – 2.22 (m, 2H), 2.14 (s, 3H), 2.13 (s, 3H), 2.06 (s, 3H), 2.03 (s, 3H), 1.90 – 1.83 (m, 1H), 1.60 (s, 7H), 1.48 – 1.40 (m, 1H), 1.38 – 1.15 (m, 4H).

^{13}C NMR (151 MHz, $CDCl_3$, 50 °C): δ 171.1, 170.7, 169.9, 169.8, 166.9, 165.4, 156.5, 154.3, 138.9, 138.0, 137.1, 133.0, 130.6, 129.8, 128.6, 128.5, 128.4, 128.3, 128.1, 128.1, 127.9, 127.7, 127.7, 127.6, 127.4, 101.7, 98.6, 89.4, 80.4, 74.5, 72.4, 72.1, 70.2, 69.3, 69.1, 68.6, 66.6, 64.1, 63.1, 62.2, 53.0, 44.9, 41.1, 38.0, 29.8, 29.6, 29.1, 27.0, 23.6, 23.6, 23.3, 21.1, 20.9, 20.8.

HRMS (QToF): Calcd for $C_{65}H_{78}Cl_2N_2NaO_{22}$ [M + Na]$^+$ 1331.4321; found 1331.4380.

Crude analytical NP-HPLC (t_R = 21.3 min)

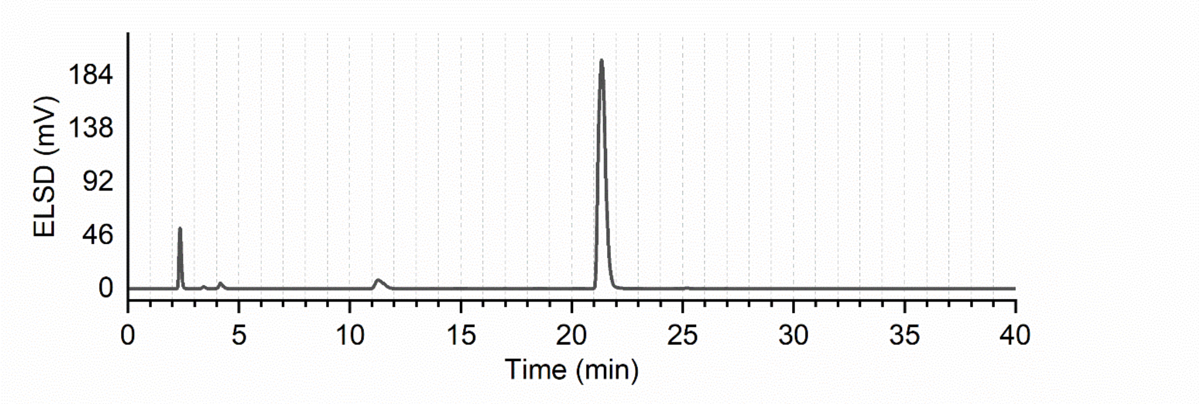

^{1}H-NMR (600 MHz, $CDCl_3$, 50 °C)

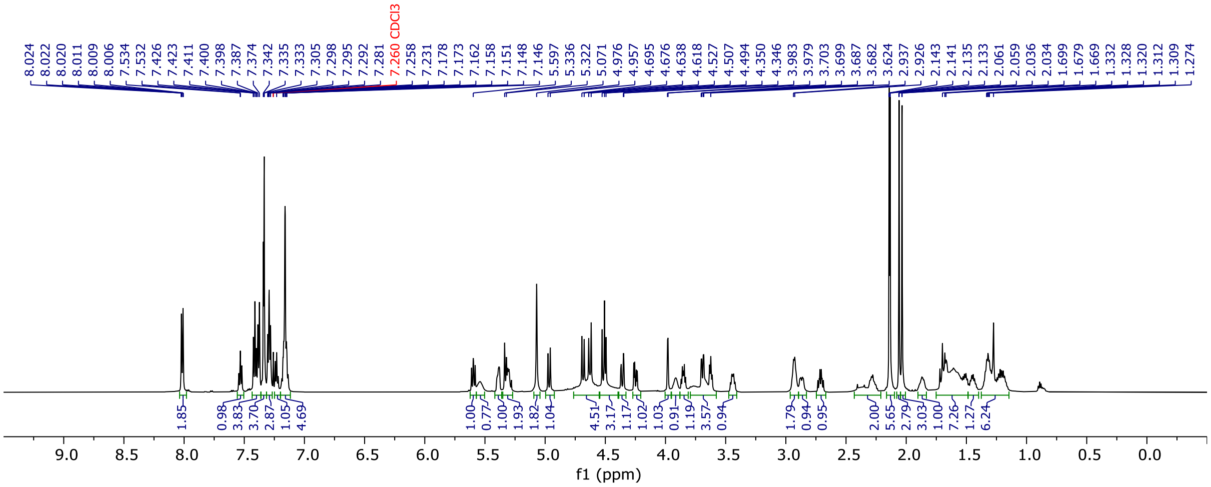

^{13}C-NMR (151 MHz, $CDCl_3$, 50 °C)

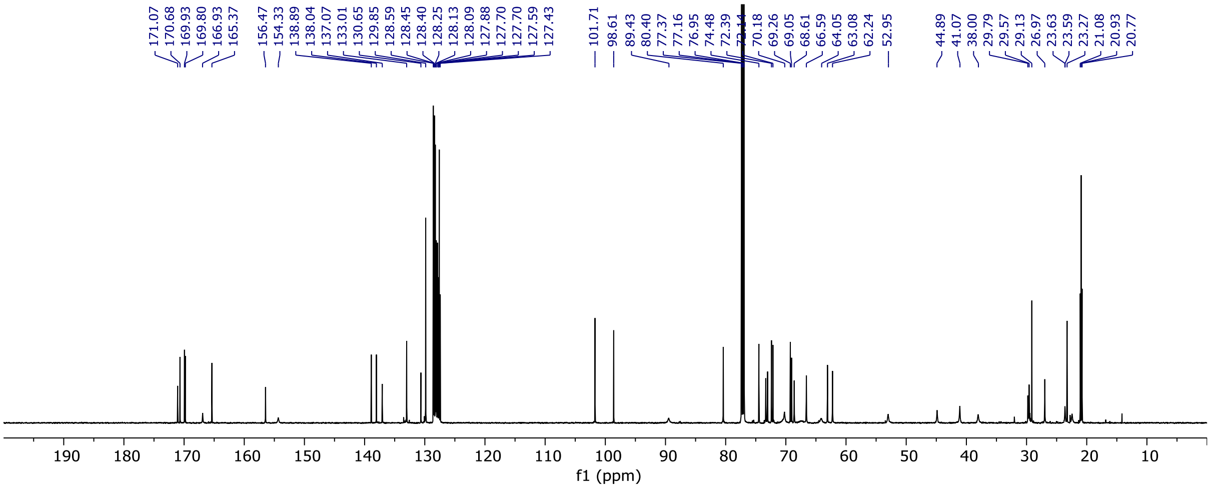

^{1}H -^{1}H COSY

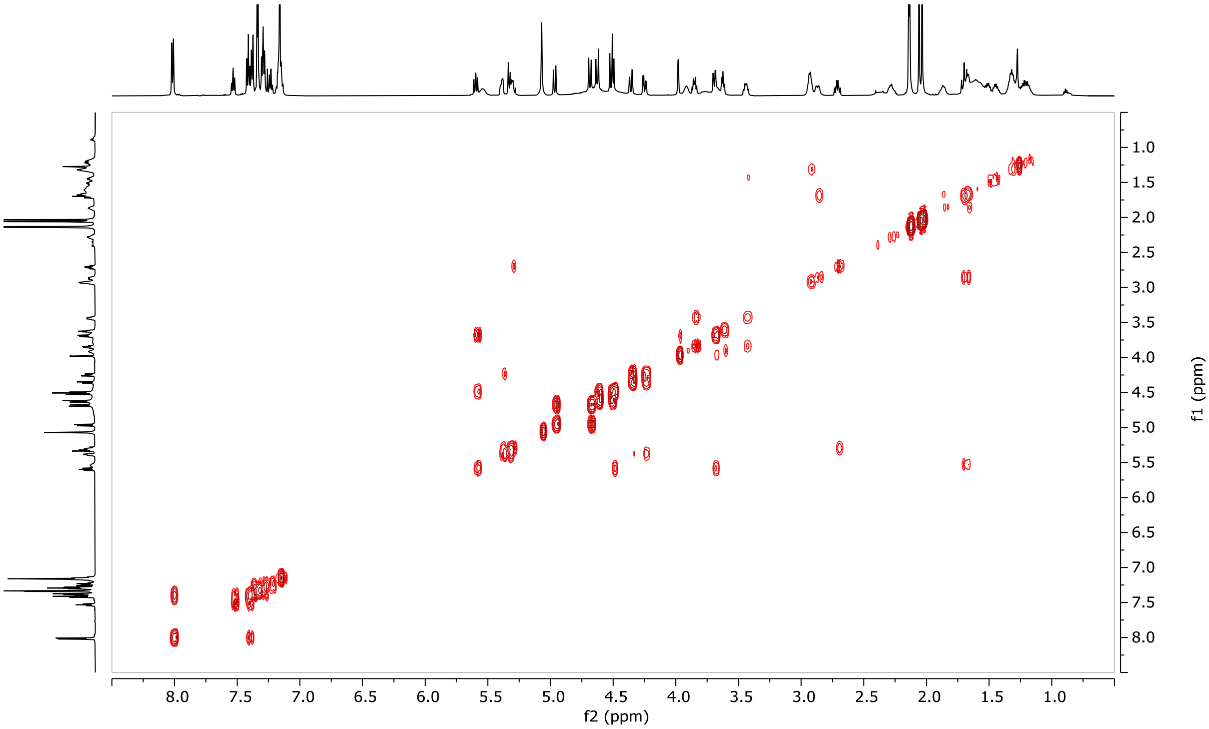

^{13}C-^{1}H HSQC

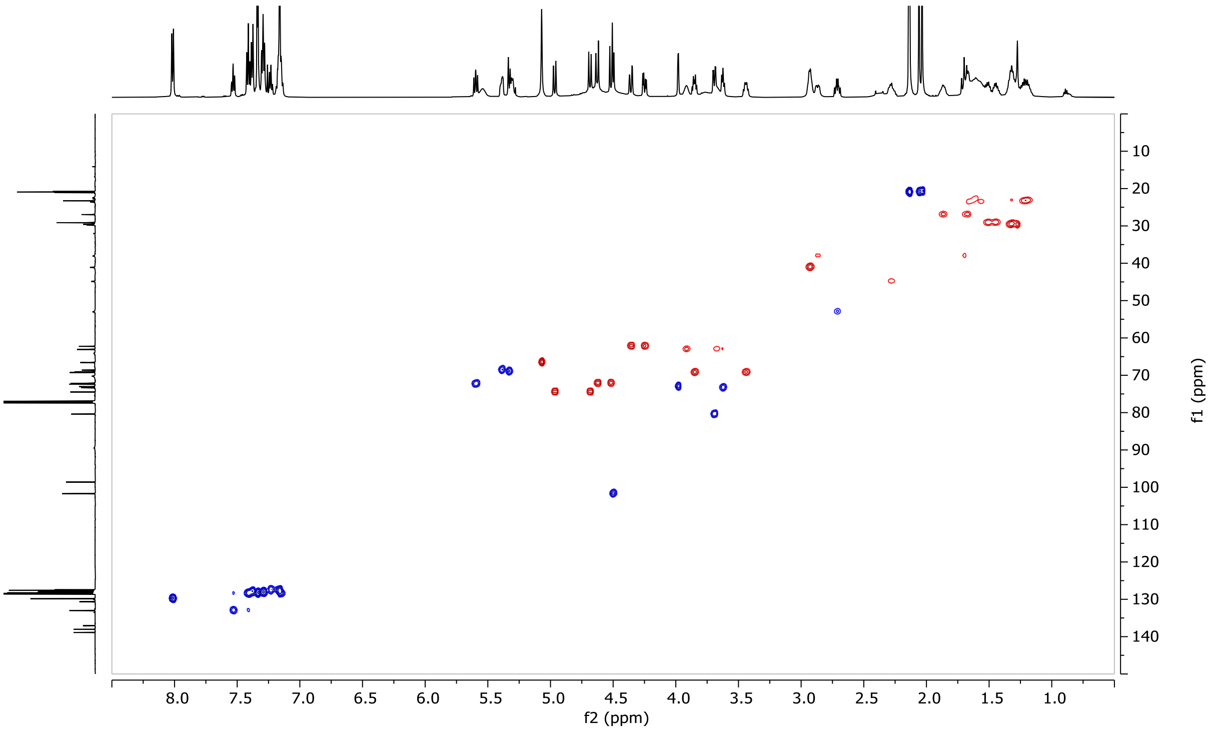

^{13}C-^{1}H HMBC

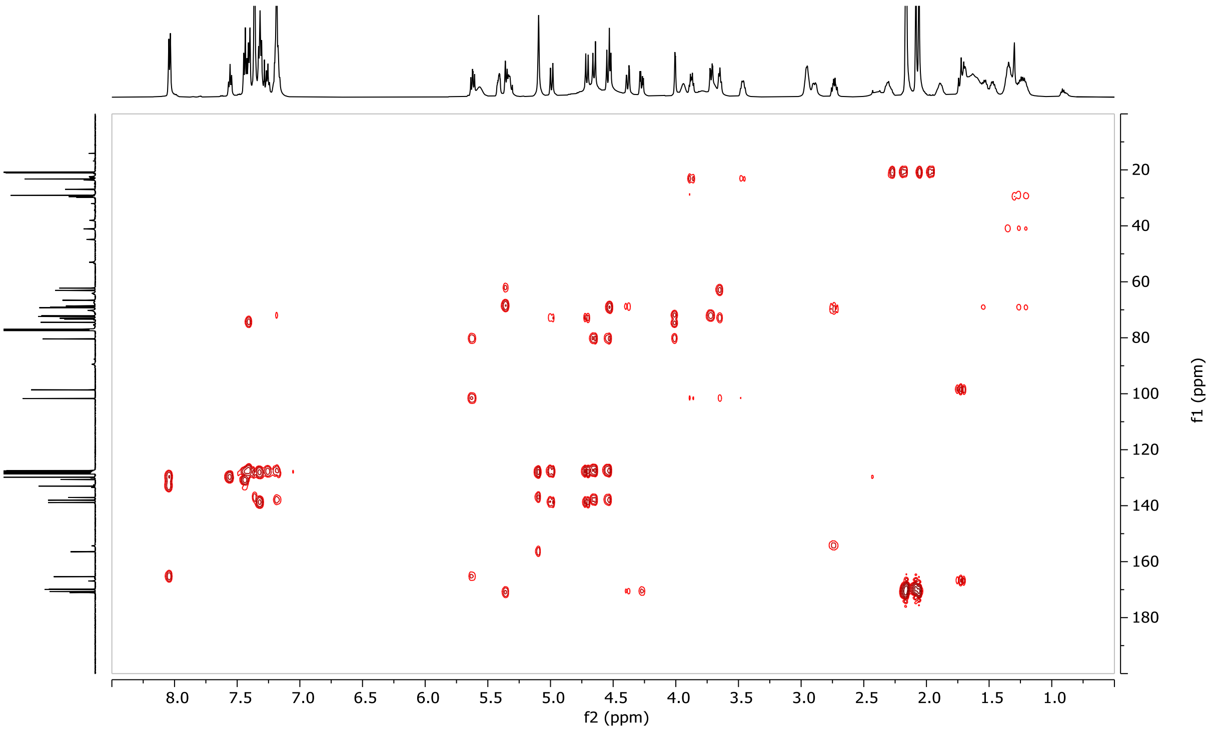

5.2 AGA of Disaccharide **5b**

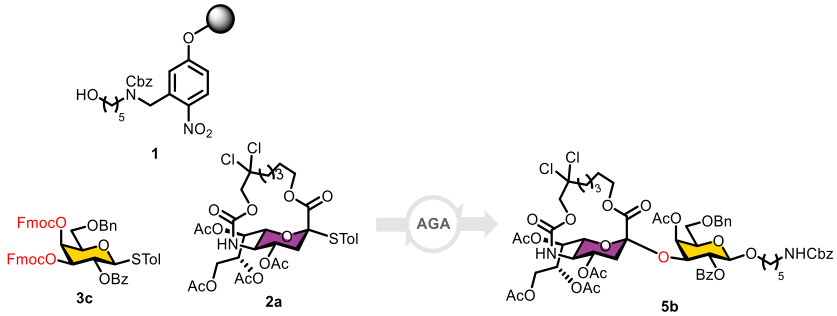

	Building blocks	Modules	Glycosylation condition
AGA		**Initiation** (40 mg resin)	
	Gal 3c	**Acidic wash**<br>**Thioglycoside glycosylation**<br>**Capping**<br>**Fmoc deprotection**	**6.5 eq.** — **t (min)**<br>T_1 = -20 °C — 5<br>T_2 = 0 °C — 20
	Sia a	**Acidic wash**<br>**Thioglycoside glycosylation x2**<br>**Capping**	**10 eq.** — **t (min)**<br>T_1 = -40 °C — 30<br>T_2 = -20 °C — 10
Post AGA		**Photocleavage**<br>**Capping** (Ac_2O/pyridine = 1/1)<br>**NP-HPLC Purification**	

Protected compound **5b** (5.8 mg, 4.60 μmol, 35%) was obtained as a colorless oil by purification using preparative NP-HPLC (**Method N1**).

^{1}H NMR (600 MHz, $CDCl_3$, 50 °C): δ 8.17 – 8.14 (m, 2H), 7.56 – 7.52 (m, 1H), 7.46 (t, J = 7.6 Hz, 2H), 7.37 – 7.27 (m, 10H), 5.62 (ddd, J = 9.8, 4.9, 2.2 Hz, 1H), 5.50 (q, J = 8.9 Hz, 1H), 5.24 (dd, J = 10.1, 7.9 Hz, 1H), 5.20 (dd, J = 9.8, 2.1 Hz, 1H), 5.18 – 5.13 (m, 1H), 5.08 (s, 3H), 5.02 – 4.96 (m, 1H), 4.71 (d, J = 7.9 Hz, 2H), 4.62 – 4.50 (m, 4H), 4.48 (d, J = 11.7 Hz, 2H), 4.30 (dd, J = 12.5, 2.2 Hz, 1H), 4.13 (dt, J = 11.2, 3.8 Hz, 1H), 4.06 (dd, J = 12.5, 5.0 Hz, 1H), 3.91 – 3.85 (m, 2H), 3.58 (dd, J = 10.0, 6.2 Hz, 1H), 3.53 – 3.45 (m, 2H), 2.91 – 2.79 (m, 2H), 2.73 – 2.63 (m, 1H), 2.43 (td, J = 10.0, 6.2 Hz, 1H), 2.36 – 2.25 (m, 2H), 2.20 (s, 3H), 2.07 (s, 3H), 2.05 (s, 3H), 2.02 – 1.97 (m, 4H), 1.70 – 1.60 (m, 2H), 1.59 – 1.45 (m, 4H), 1.45 – 1.34 (m, 5H), 1.32 – 1.20 (m, 2H), 1.20 – 1.05 (m, 2H).

^{13}C NMR (151 MHz, $CDCl_3$, 50 °C): δ 170.8, 170.6, 170.5, 170.2, 169.9, 167.8, 165.5, 156.5, 154.7, 138.3, 137.1, 133.1, 131.0, 130.3, 128.7, 128.6, 128.5, 128.2, 128.2, 127.8, 127.8, 101.8, 96.5, 96.5, 90.1, 73.7, 72.4, 71.9, 71.8, 70.1, 68.8, 68.7, 68.2, 67.3, 66.7,

64.9, 64.9, 62.6, 52.4, 44.7, 41.1, 37.4, 34.3, 29.9, 29.6, 29.2, 26.9, 23.7, 23.4, 21.5, 20.9, 20.9, 20.1.

HRMS (QToF): Calcd for $C_{60}H_{74}Cl_2N_2NaO_{23}$ $[M + Na]^+$ 1283.3957; found 1283.4000.

Crude analytical NP-HPLC (t_R = 21.1 min)

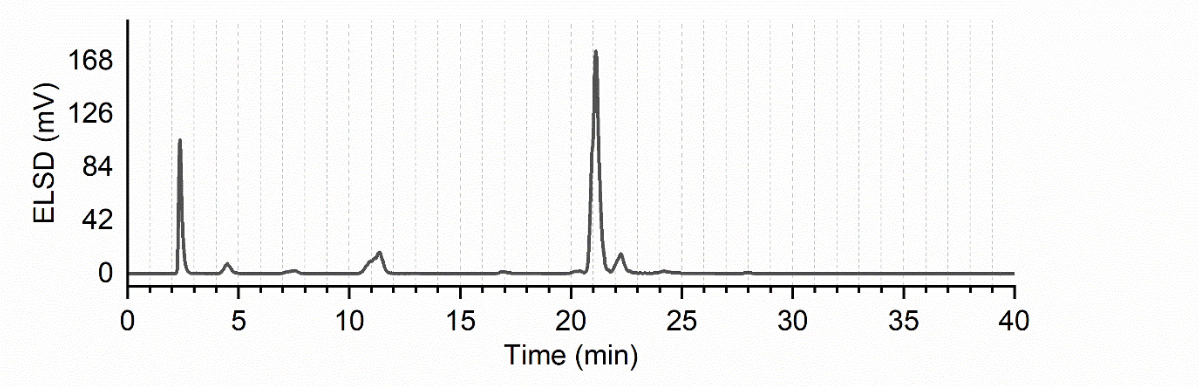

^{1}H-NMR (600 MHz, $CDCl_3$, 50 °C)

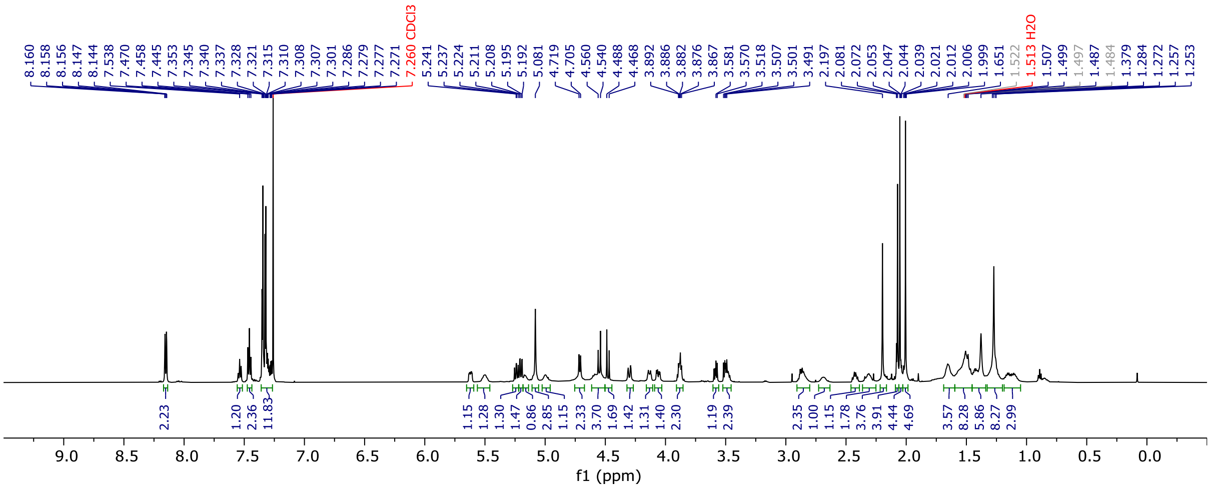

^{13}C-NMR (151 MHz, $CDCl_3$, 50 °C)

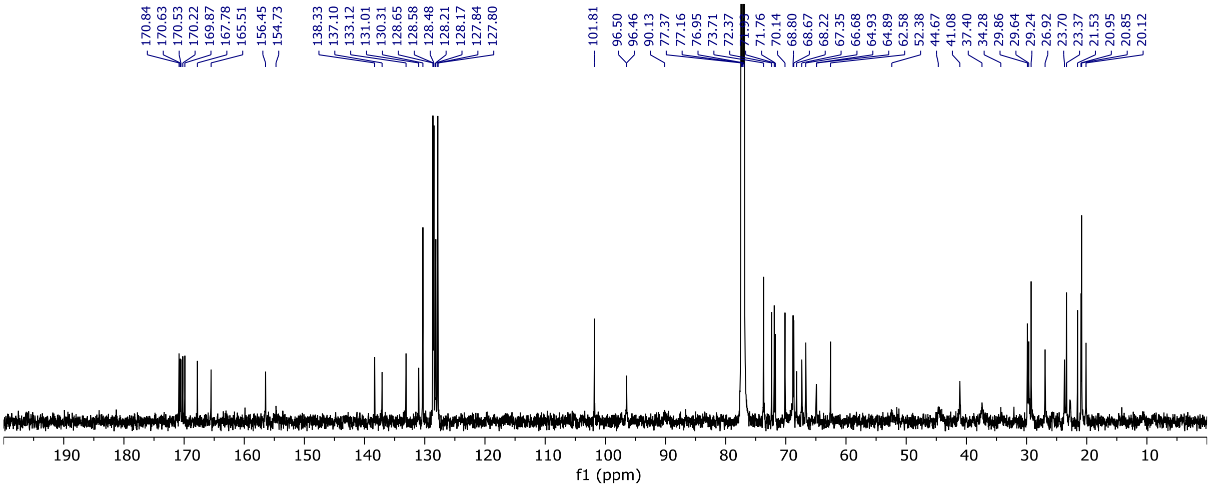

^{1}H -^{1}H COSY

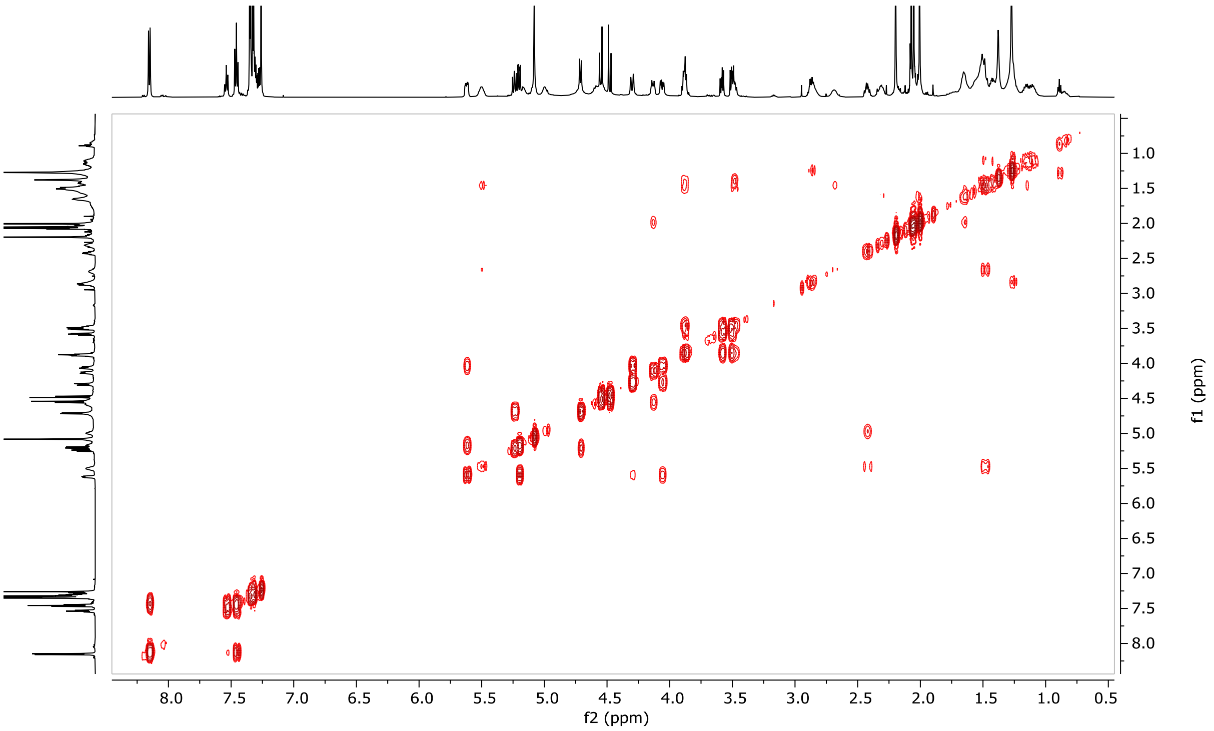

^{13}C-^{1}H HSQC

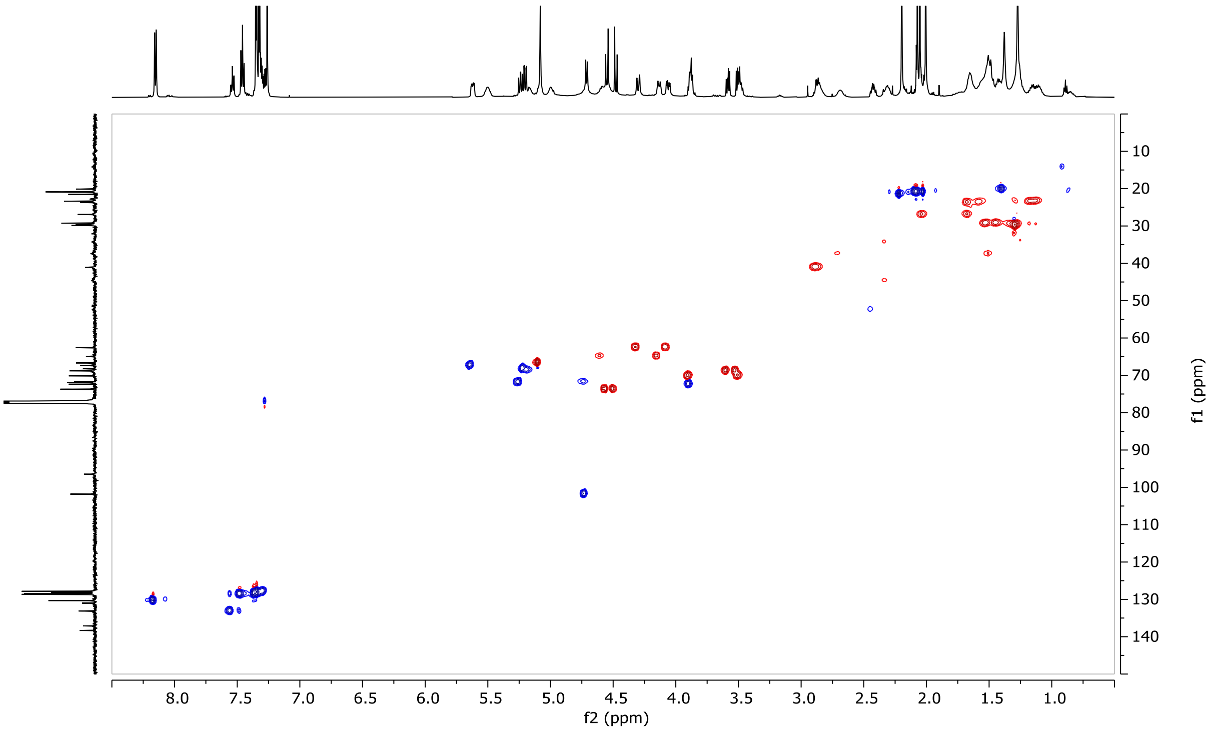

^{13}C-^{1}H HMBC

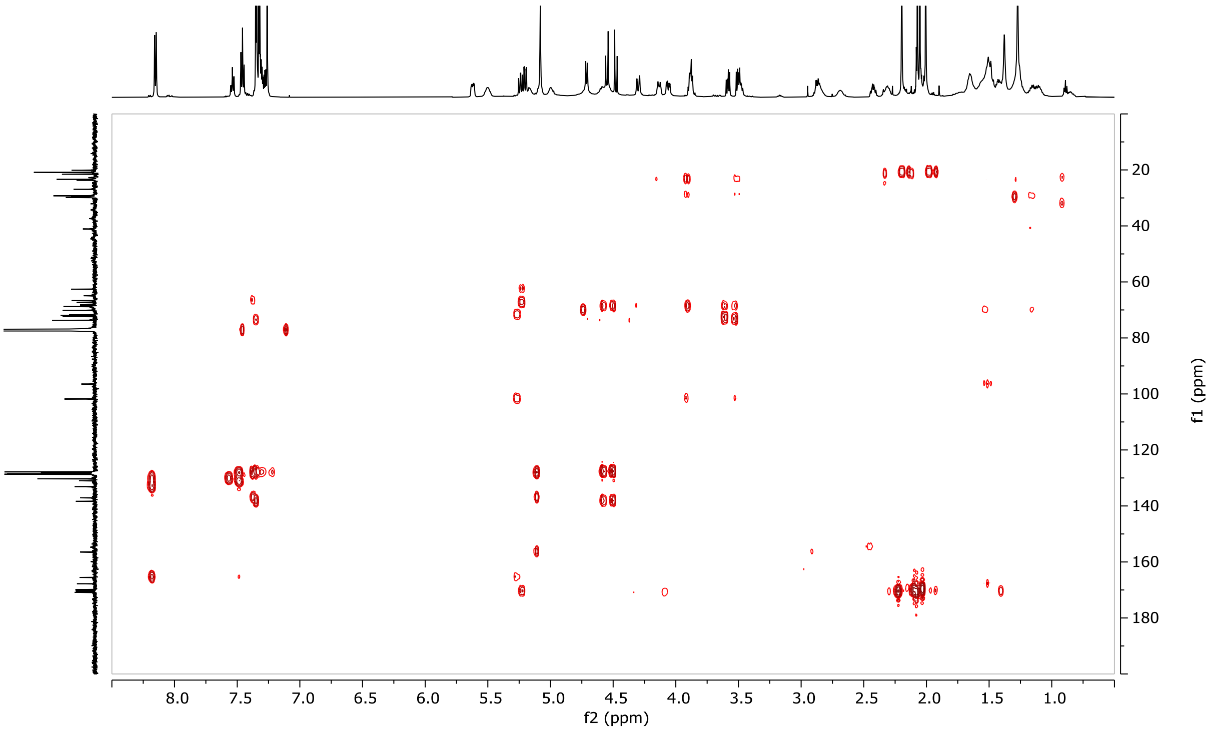

5.3 AGA of Pentasaccharide **9**

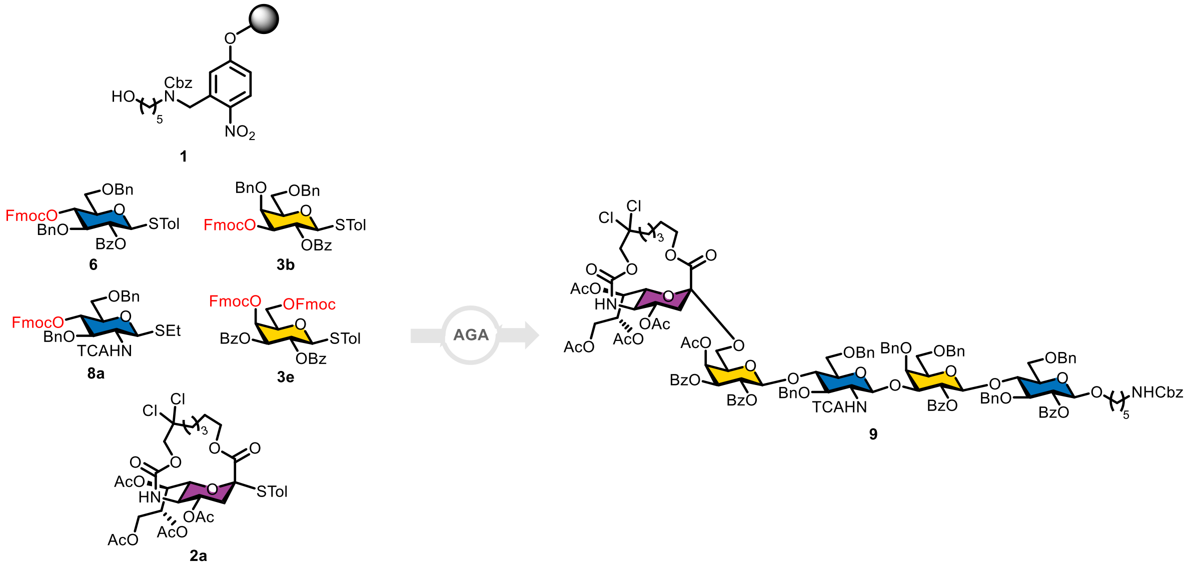

	Building blocks	Modules	Glycosylation condition
AGA		**Initiation** (40 mg resin)	
	**Glc 6**	**Acidic wash**<br>**Thioglycoside glycosylation**<br>**Capping**<br>**Fmoc deprotection**	8 eq. — t (min)<br>T_1 = -20 °C — 5<br>T_2 = 0 °C — 20
	**Gal 3b**	**Acidic wash**<br>**Thioglycoside glycosylation**<br>**Capping**<br>**Fmoc deprotection**	8 eq. — t (min)<br>T_1 = -40 °C — 5<br>T_2 = -20 °C — 20
	**GlcN 8a**	**Acidic wash**<br>**Thioglycoside glycosylation**<br>**Capping**<br>**Fmoc deprotection**	8 eq. — t (min)<br>T_1 = -20 °C — 5<br>T_2 = 0 °C — 40
	**Gal 3e**	**Acidic wash**<br>**Thioglycoside glycosylation**<br>**Capping**<br>**Fmoc deprotection**	8 eq. — t (min)<br>T_1 = -20 °C — 5<br>T_2 = 0 °C — 20
	**Sia 2a**	**Acidic wash**<br>**Thioglycoside glycosylation**<br>**Pyridine wash**] **x4**	10 eq. — t (min)<br>T_1 = -40 °C — 30<br>T_2 = -20 °C — 10
Post AGA		**Photocleavage**<br>**Capping** (Ac_2O/pyridine = 1/1)<br>**NP-HPLC Purification**	

Protected compound **9** (11.1 mg, 4.13 μmol, 31%) was obtained as a colorless oil by purification using preparative NP-HPLC (**Method N1**).

^{1}H NMR (600 MHz, $CDCl_3$, 50 °C): δ 7.95 (dd, J = 8.2, 1.4 Hz, 6H), 7.85 (dd, J = 8.3, 1.4 Hz, 2H), 7.56 – 7.52 (m, 1H), 7.52 – 7.44 (m, 4H), 7.41 (t, J = 7.7 Hz, 3H), 7.39 – 7.31 (m, 15H), 7.31 – 7.14 (m, 19H), 7.10 – 7.06 (m, 2H), 7.05 – 6.99 (m, 1H), 6.95 (dd, J = 8.2, 6.9 Hz, 2H), 6.65 (d, J = 8.2 Hz, 1H), 5.67 (d, J = 3.5 Hz, 1H), 5.58 – 5.52 (m, 2H), 5.51 – 5.45 (m, 1H), 5.43 (dd, J = 10.5, 3.5 Hz, 1H), 5.41 – 5.37 (m, 1H), 5.34 (dd, J = 9.1, 1.3 Hz, 1H), 5.21 (d, J = 6.6 Hz, 1H), 5.13 (dd, J = 9.2, 7.9 Hz, 1H), 5.06 (s, 2H), 4.98 (d, J = 11.6 Hz, 1H), 4.94 (d, J = 7.9 Hz, 1H), 4.89 (d, J = 11.3 Hz, 1H), 4.86 (d, J = 11.0 Hz, 1H), 4.75 (d, J = 7.1 Hz, 1H), 4.61 (d, J = 11.0 Hz, 1H), 4.58 – 4.52 (m, 3H), 4.48 (dd, J = 11.9, 7.6 Hz, 3H), 4.39 – 4.31 (m, 4H), 4.31 – 4.25 (m, 3H), 4.19 (d, J = 11.9 Hz, 1H), 4.05 (t, J = 7.3 Hz, 1H), 4.02 – 3.93 (m, 3H), 3.93 – 3.85 (m, 1H), 3.83 (t, J = 7.5 Hz, 1H), 3.79 – 3.61 (m, 7H), 3.52 (dd, J = 11.1, 4.1 Hz, 1H), 3.49 – 3.34 (m, 7H), 3.32 – 3.26 (m, 2H), 3.22 – 3.16 (m, 1H), 2.95 – 2.86 (m, 2H), 2.79 (d, J = 4.6 Hz, 1H), 2.75 (dd, J = 12.6, 4.6

Hz, 1H), 2.67 (td, J = 10.5, 6.5 Hz, 1H), 2.30 – 2.23 (m, 2H), 2.16 (s, 3H), 2.07 (s, 3H), 2.06 (s, 3H), 2.02 (s, 3H), 2.00 (s, 3H), 1.95 – 1.87 (m, 1H), 1.76 – 1.68 (m, 1H), 1.67 – 1.52 (m, 5H), 1.49 – 1.35 (m, 2H), 1.34 – 1.23 (m, 2H), 1.22 – 1.09 (m, 2H).

^{13}C NMR (151 MHz, $CDCl_3$, 50 °C): δ 171.5, 170.8, 169.9, 169.9, 169.7, 165.5, 165.2, 164.9, 161.7, 156.5, 139.3, 138.9, 138.5, 138.5, 138.5, 138.2, 137.1, 133.6, 133.4, 133.3, 133.0, 130.5, 130.2, 130.2, 130.1, 130.1, 129.9, 129.8, 129.8, 129.8, 129.6, 129.4, 128.7, 128.7, 128.6, 128.6, 128.5, 128.5, 128.3, 128.2, 128.2, 128.2, 128.1, 128.1, 128.1, 128.0, 128.0, 127.9, 127.9, 127.8, 127.8, 127.7, 127.6, 127.4, 127.0, 101.3, 100.9, 100.8, 99.1, 99.1, 92.4, 80.9, 80.8, 79.5, 78.1, 76.7, 76.7, 76.4, 75.7, 75.3, 75.1, 74.7, 74.0, 73.8, 73.6, 73.6, 73.6, 73.6, 72.9, 72.1, 71.8, 70.6, 69.4, 69.3, 69.2, 69.1, 68.8, 68.7, 68.2, 67.6, 66.7, 66.7, 66.6, 66.6, 62.5, 57.1, 57.1, 52.9, 44.8, 44.8, 43.3, 41.1, 38.0, 38.0, 29.6, 29.1, 27.0, 23.6, 23.5, 23.3, 21.1, 20.9, 20.9, 20.8, 20.7.

HRMS (QToF): Calcd for $C_{136}H_{146}Cl_5N_3NaO_{41}$ $[M + Na]^+$ 2674.7772; found 2674.7808.

^{1}H-NMR (600 MHz, $CDCl_3$, 50 °C)

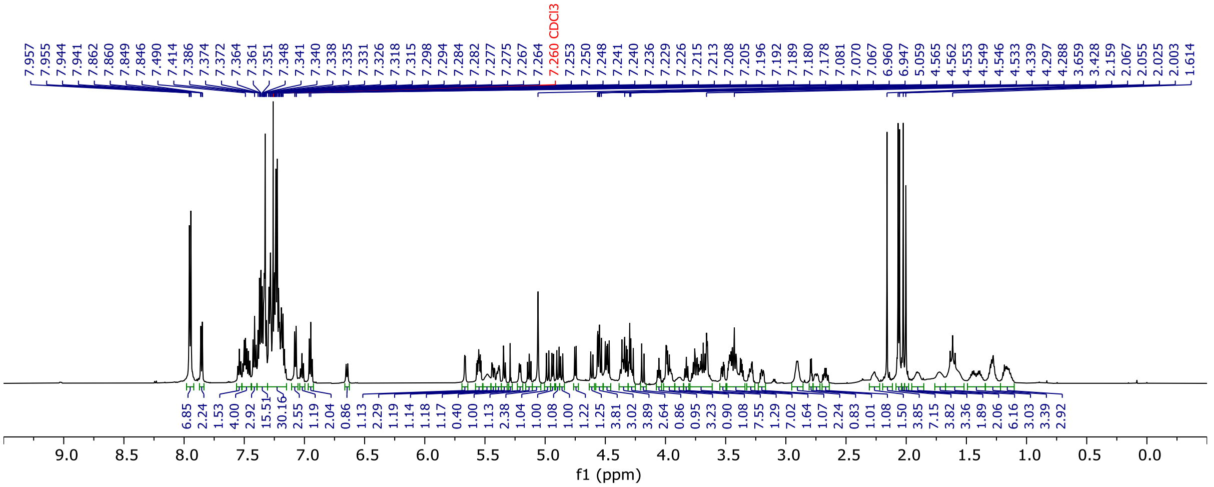

^{13}C-NMR (151 MHz, $CDCl_3$, 50 °C)

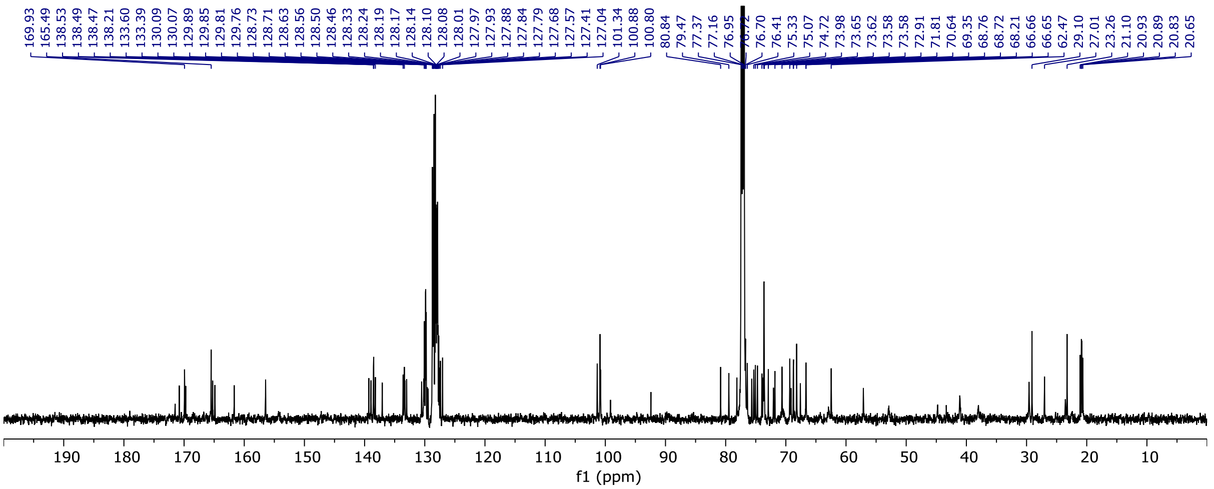

^{1}H -^{1}H COSY

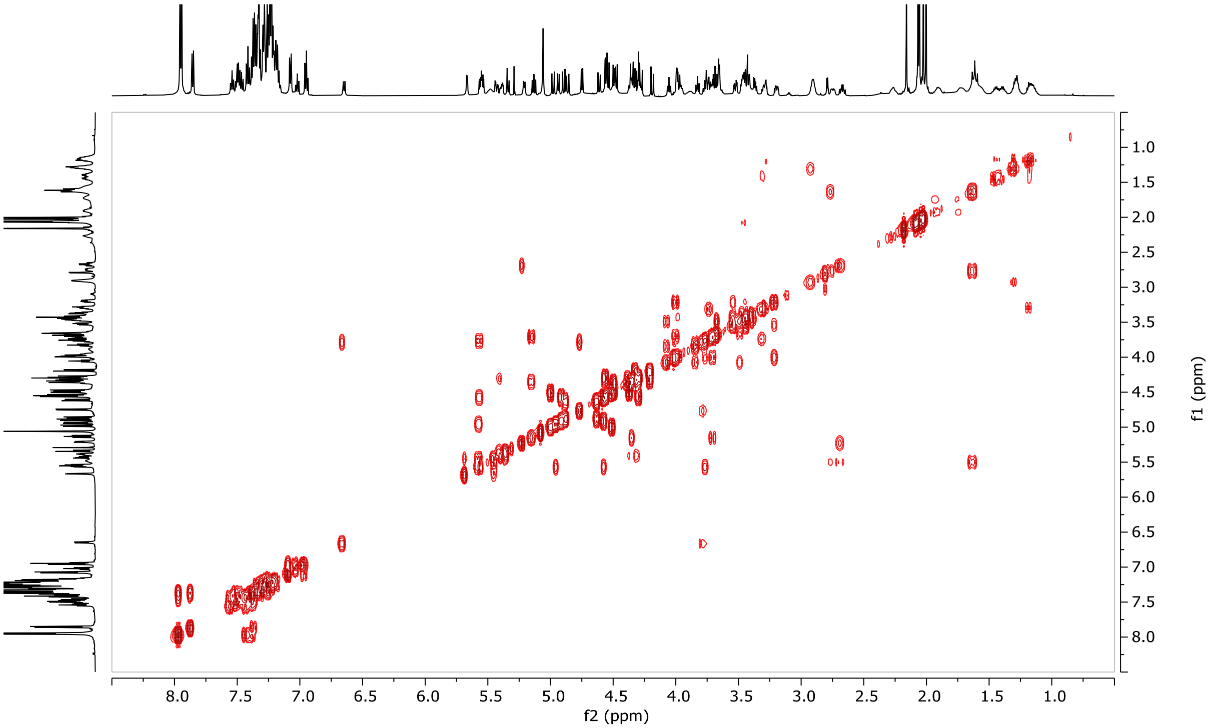

^{13}C-^{1}H HSQC

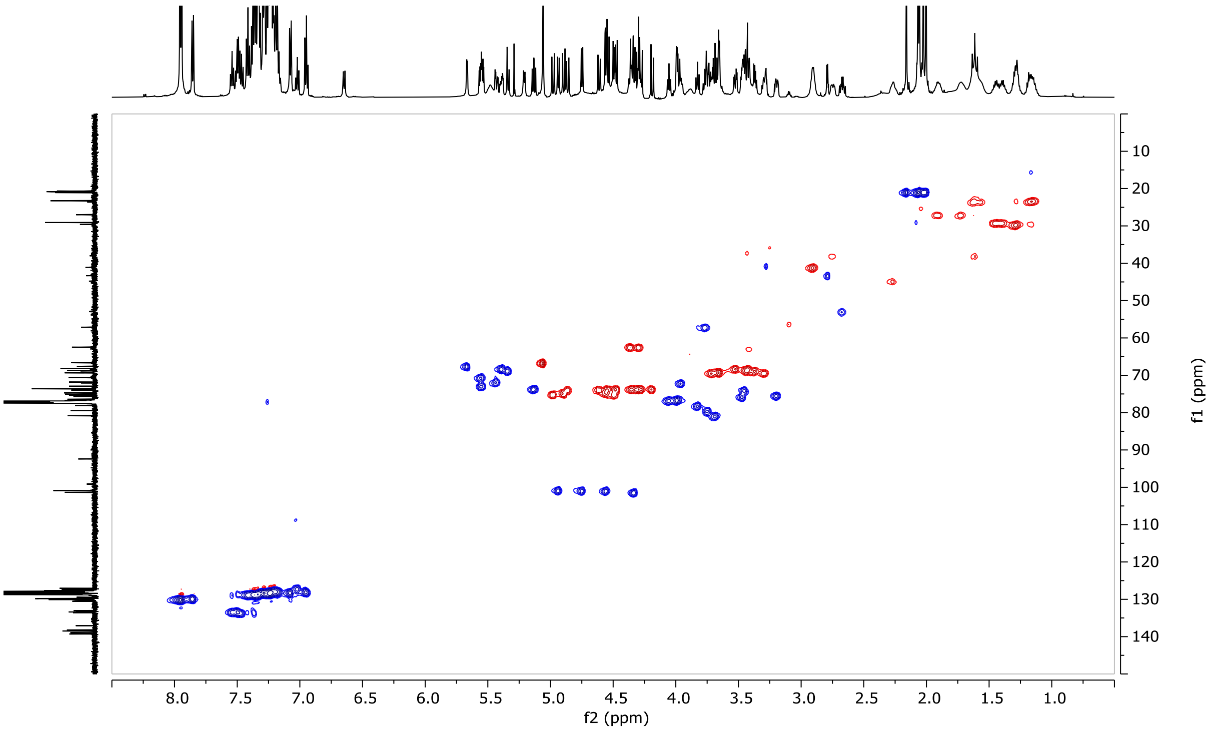

^{13}C-^{1}H HMBC

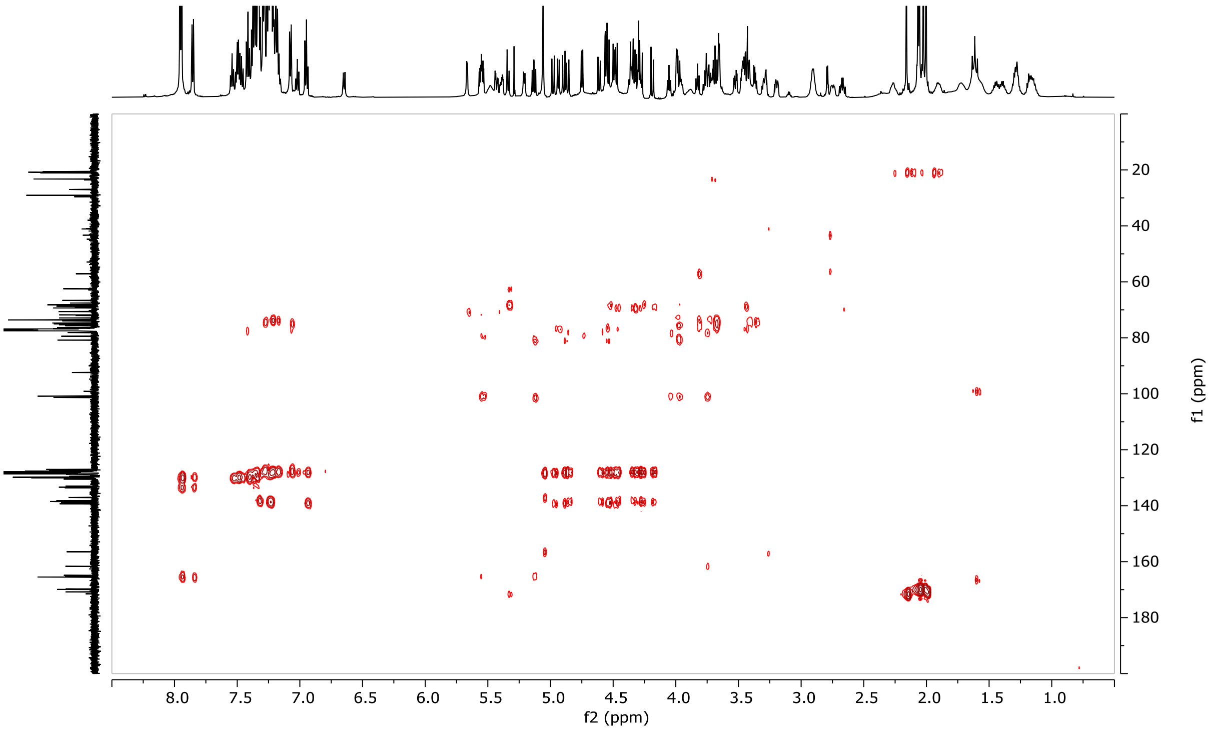

5.4 AGA of Pentasaccharide **10**

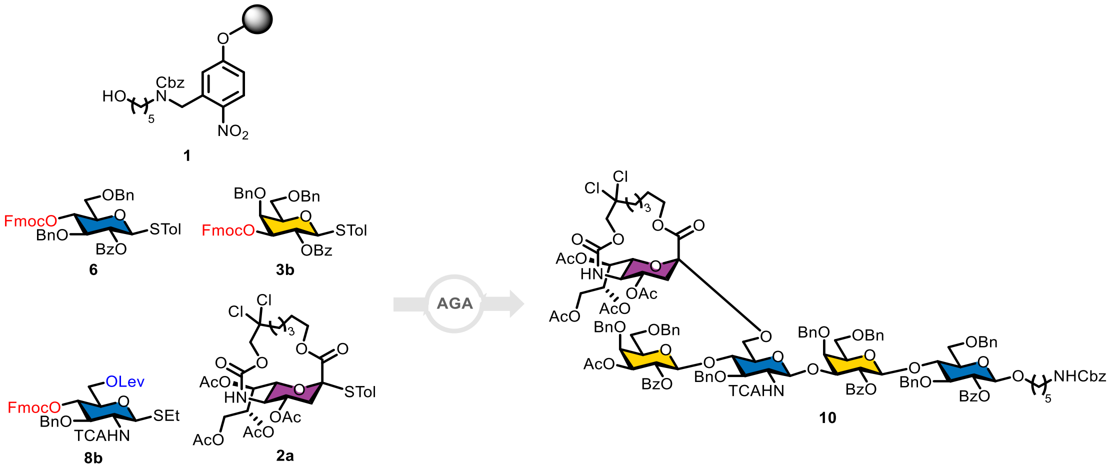

	Building blocks	Modules	Glycosylation condition
AGA		**Initiation** (40 mg resin)	
	**Glc 6**	**Acidic wash**<br>**Thioglycoside glycosylation**<br>**Capping**<br>**Fmoc deprotection**	**8 eq.** — **t (min)**<br>T_1 = -20 °C — 5<br>T_2 = 0 °C — 20
	**Gal 3b**	**Acidic wash**<br>**Thioglycoside glycosylation**<br>**Capping**<br>**Fmoc deprotection**	**8 eq.** — **t (min)**<br>T_1 = -40 °C — 5<br>T_2 = -20 °C — 20
	**GlcN 8b**	**Acidic wash**<br>**Thioglycoside glycosylation**<br>**Capping**<br>**Lev deprotection**	**8 eq.** — **t (min)**<br>T_1 = -20 °C — 5<br>T_2 = 0 °C — 40
	**Sia 2a**	**Acidic wash**<br>**Thioglycoside glycosylation** **x2**<br>**Pyridine wash**<br>**Capping**<br>**Fmoc deprotection**	**10 eq.** — **t (min)**<br>T_1 = -40 °C — 30<br>T_2 = -20 °C — 10
	**Gal 3b**	**Acidic wash**<br>**Thioglycoside glycosylation**<br>**Capping**<br>**Fmoc deprotection**	**8 eq.** — **t (min)**<br>T_1 = -40 °C — 5<br>T_2 = -20 °C — 20
**Post AGA**		**Photocleavage**<br>**Capping** (Ac_2O/pyridine = 1/1)<br>**NP-HPLC Purification**	

Protected compound **10** (7.9 mg, 2.98 µmol, 23%) was obtained as a colorless oil by purification using preparative NP-HPLC (**Method N1**).

^{1}H NMR (600 MHz, $CDCl_3$, 50 °C): δ 8.13 – 8.09 (m, 2H), 8.00 – 7.90 (m, 4H), 7.56 – 7.46 (m, 6H), 7.41 – 7.34 (m, 5H), 7.34 – 7.30 (m, 5H), 7.30 – 7.14 (m, 23H), 7.14 – 7.11 (m, 2H), 7.11 – 7.03 (m, 5H), 7.00 (t, J = 7.3 Hz, 1H), 6.92 (t, J = 7.5 Hz, 2H), 6.56 (d, J = 8.1 Hz, 1H), 5.61 – 5.48 (m, 4H), 5.38 (dd, J = 9.7, 1.7 Hz, 1H), 5.23 (dd, J = 10.5, 3.2 Hz, 1H), 5.21 – 5.16 (m, 1H), 5.13 (dd, J = 9.2, 7.9 Hz, 1H), 5.05 (s, 2H), 4.99 (d, J = 11.7 Hz, 1H), 4.90 (d, J = 11.3 Hz, 1H), 4.86 – 4.79 (m, 2H), 4.73 – 4.66 (m, 2H), 4.60 (d, J = 7.8 Hz, 1H), 4.57 – 4.51 (m, 4H), 4.49 (d, J = 11.5 Hz, 2H), 4.46 – 4.39 (m, 3H), 4.37 – 4.28 (m, 5H), 4.24 (dd, J = 12.5, 4.3 Hz, 1H), 4.18 (d, J = 12.3 Hz, 1H), 4.06 – 4.00 (m, 2H), 3.97 (t, J = 9.0 Hz, 1H), 3.94 – 3.91 (m, 2H), 3.86 – 3.79 (m, 1H), 3.76 – 3.66 (m, 6H), 3.63 (t, J = 7.5 Hz, 1H), 3.60 – 3.51 (m, 3H), 3.50 – 3.41 (m, 5H), 3.42 – 3.33 (m, 2H), 3.33 – 3.26 (m, 1H), 3.21 (ddd, J = 9.7, 4.3, 2.1 Hz, 1H), 2.95 – 2.86 (m, 2H), 2.81 (dd, J = 12.5, 5.4 Hz, 1H), 2.71 (td, J = 10.4, 6.3 Hz, 1H), 2.32 – 2.22 (m, 2H), 2.18 (s, 3H), 2.03 (s, 3H), 1.99 (s, 3H), 1.97 (s, 3H), 1.86 (s, 3H), 1.74 – 1.65 (m, 1H), 1.64 (t, J = 12.2 Hz, 1H), 1.55 (d, J = 32.3 Hz, 4H), 1.48 – 1.35 (m, 3H), 1.33 – 1.23 (m, 2H), 1.21 – 1.10 (m, 2H).

^{13}C NMR (151 MHz, $CDCl_3$, 50 °C): δ 171.2, 170.6, 170.4, 169.9, 169.9, 166.7, 165.7, 165.2, 164.9, 161.8, 156.4, 154.4, 139.3, 139.0, 138.6, 138.6, 138.4, 138.3, 138.2, 138.0, 137.1, 133.6, 133.4, 133.0, 130.5, 130.1, 130.0, 130.0, 129.8, 128.9, 128.8, 128.6, 128.5, 128.5, 128.5, 128.5, 128.3, 128.2, 128.2, 128.1, 128.1, 128.1, 127.9, 127.9, 127.8, 127.8, 127.8, 127.7, 127.7, 127.5, 127.4, 127.0, 101.4, 100.8, 100.8, 99.8, 98.6, 92.4, 89.8, 80.9, 80.8, 79.8, 79.7, 79.7, 79.6, 78.4, 76.5, 75.4, 75.4, 75.1, 75.1, 75.0, 74.8, 74.7, 74.7, 74.6, 74.5, 73.9, 73.8, 73.8, 73.6, 73.6, 73.0, 71.4, 69.4, 69.3, 68.7, 68.3, 68.1, 67.7, 66.6, 64.0, 62.3, 56.9, 52.9, 44.7, 41.1, 38.0, 38.0, 29.6, 29.1, 26.8, 23.5, 23.3, 21.3, 20.9, 20.8, 20.8.

HRMS (QToF): Calcd for $C_{136}H_{148}Cl_5N_3NaO_{40}$ $[M + Na]^+$ 2660.7979; found 2660.8057.

Crude analytical NP-HPLC (t_R = 25.3 min)

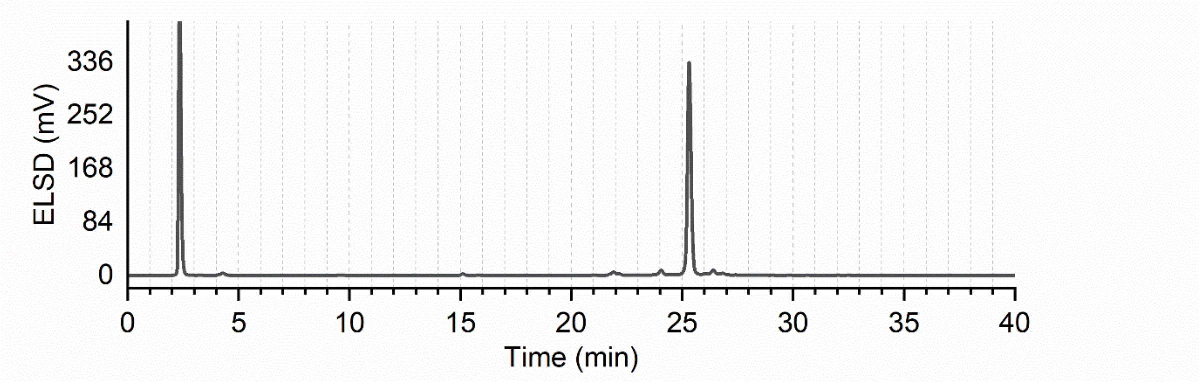

^{1}H-NMR (600 MHz, $CDCl_3$, 50 °C)

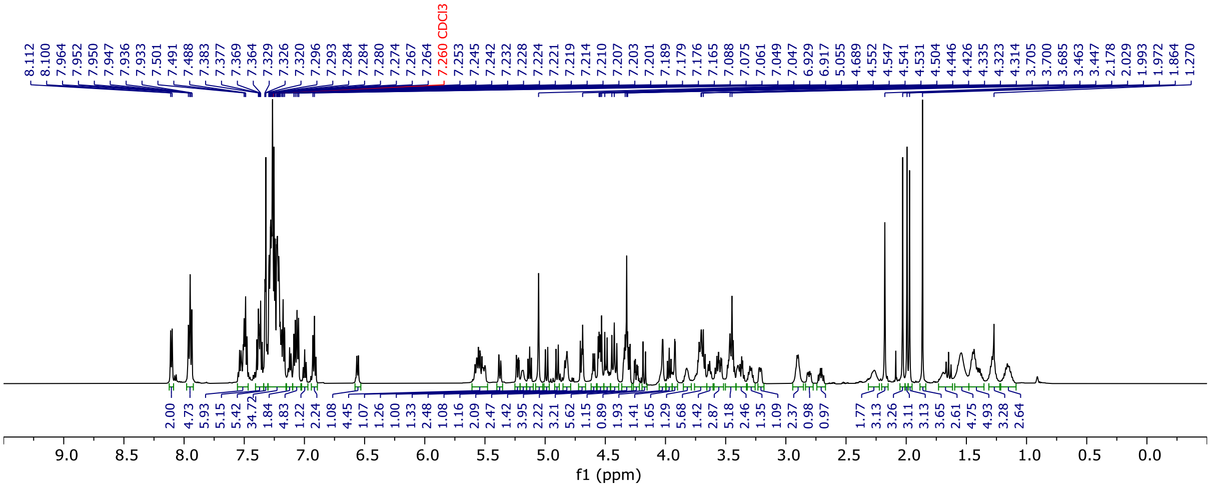

^{13}C-NMR (151 MHz, $CDCl_3$, 50 °C)

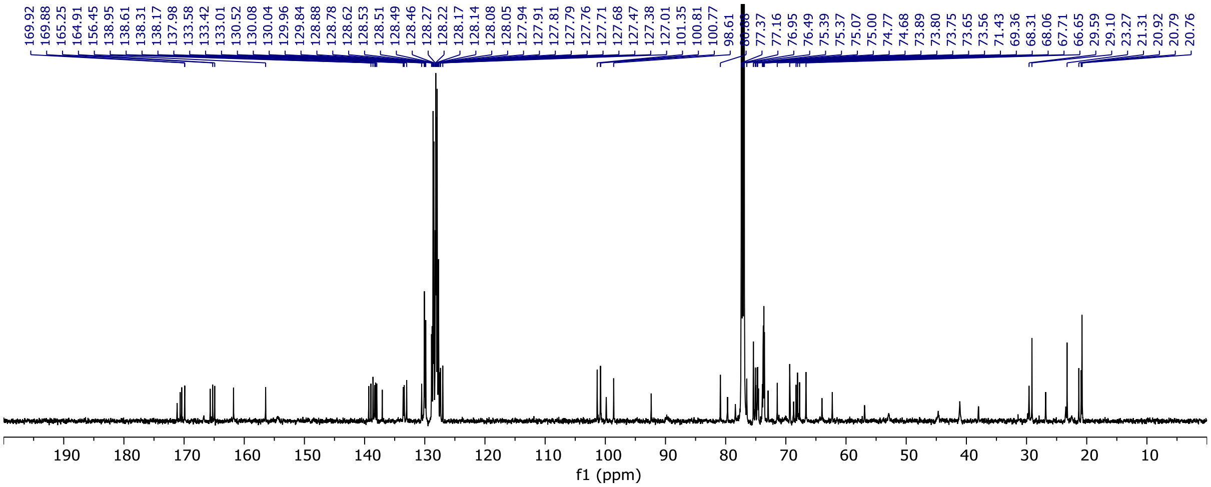

^{1}H -^{1}H COSY

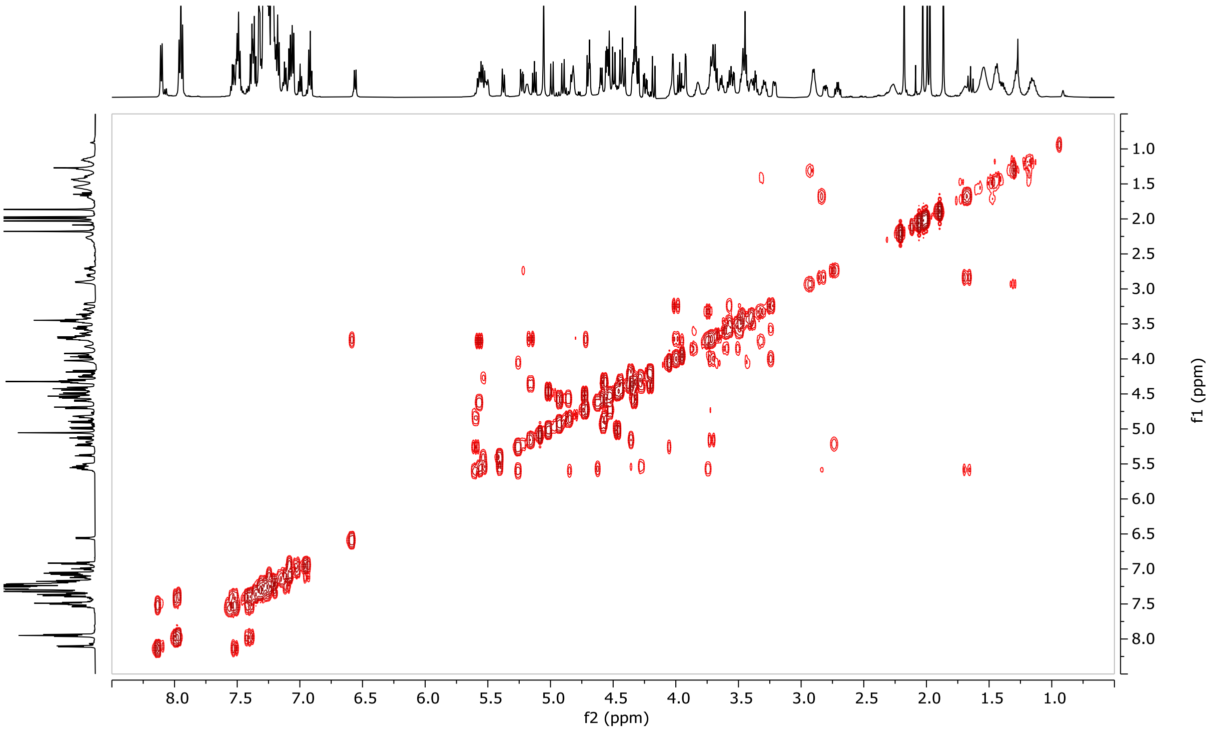

^{13}C-^{1}H HSQC

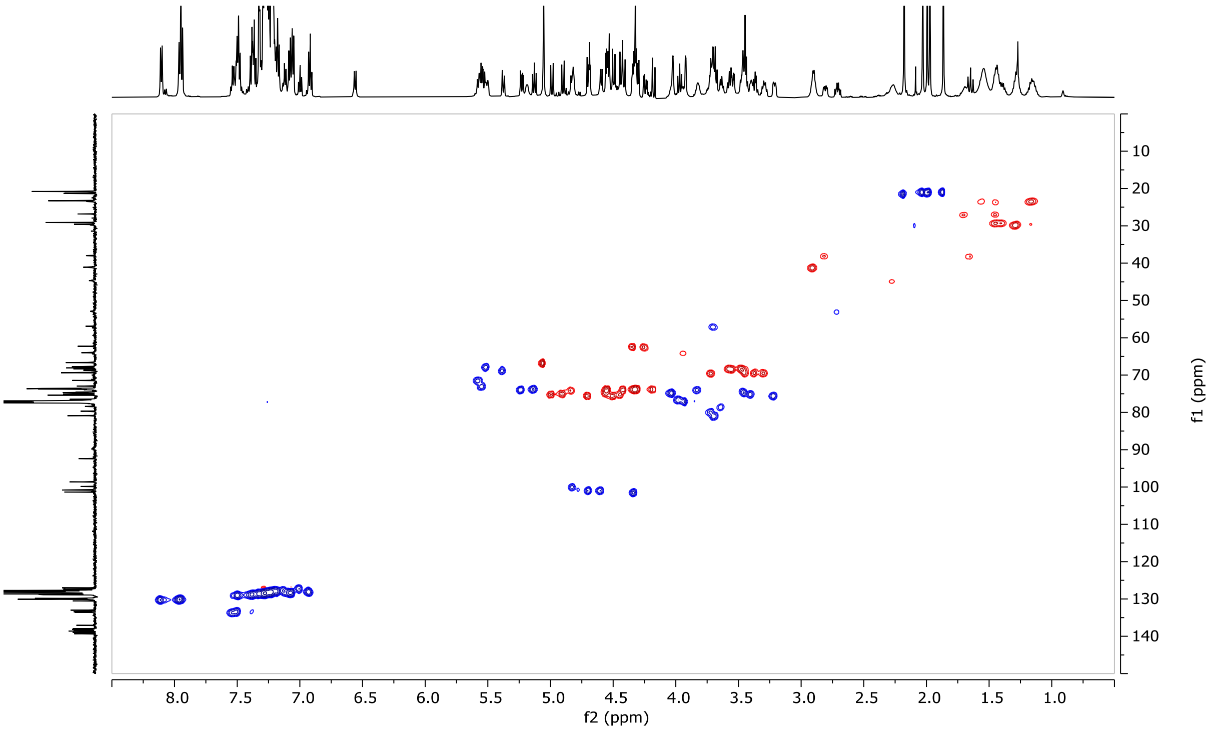

^{13}C-^{1}H HMBC

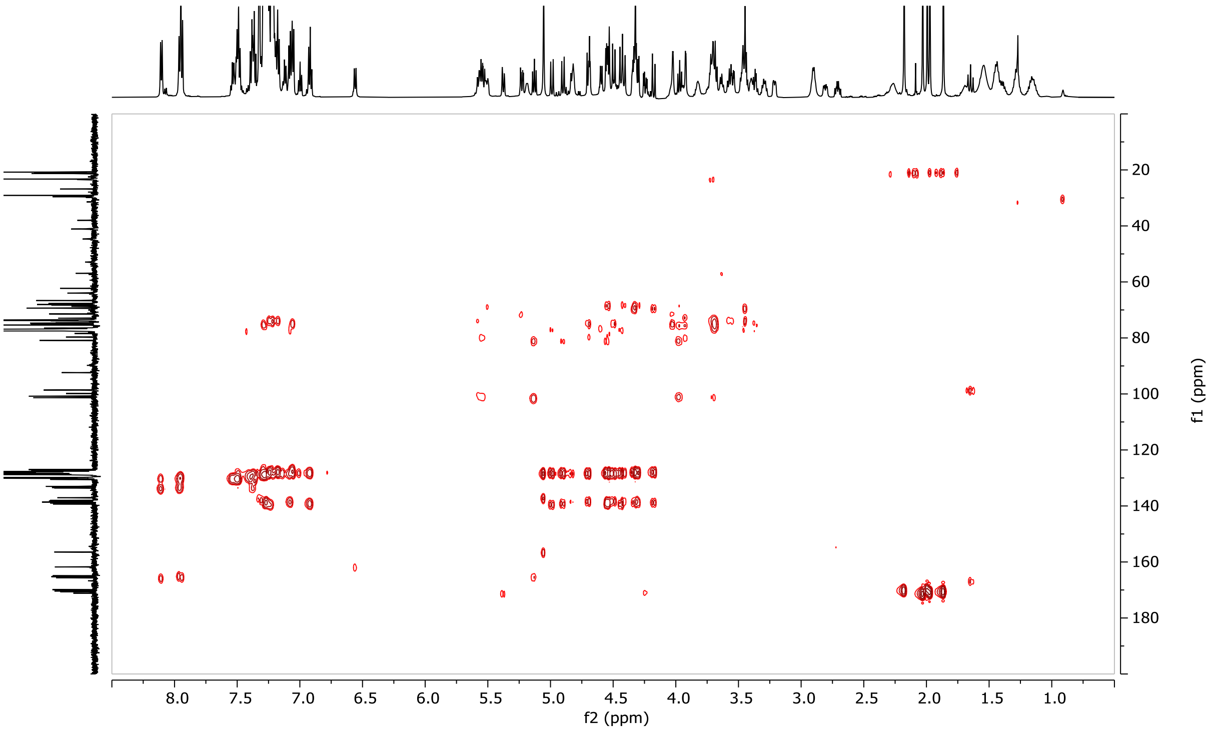

5.5 AGA of Pentasaccharide **11**

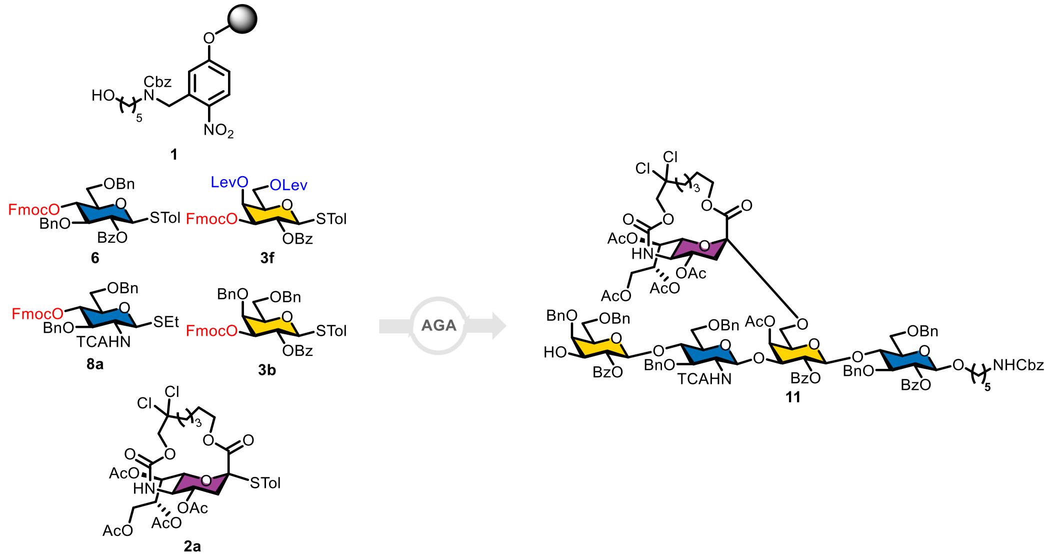

	Building blocks	Modules	Glycosylation condition
AGA		**Initiation** (40 mg resin)	
	**Glc 6**	**Acidic wash**<br>**Thioglycoside glycosylation**<br>**Capping**<br>**Fmoc deprotection**	8 eq. / t (min)<br>T_1 = -20 °C / 5<br>T_2 = 0 °C / 20
	**Gal 3f**	**Acidic wash**<br>**Thioglycoside glycosylation**<br>**Capping**<br>**Lev deprotection x2**	8 eq. / t (min)<br>T_1 = -20 °C / 5<br>T_2 = 0 °C / 20
	**Sia 2a**	[**Acidic wash**<br>**Thioglycoside glycosylation**<br>**Pyridine wash**] **x4**<br>**Basic Capping**<br>**Fmoc deprotection**	10 eq. / t (min)<br>T_1 = -40 °C / 30<br>T_2 = -20 °C / 10
	**GlcN 8a**	**Acidic wash**<br>**Thioglycoside glycosylation**<br>**Capping**<br>**Fmoc deprotection**	8 eq. / t (min)<br>T_1 = -20 °C / 5<br>T_2 = 0 °C / 40
	**Gal 3b**	**Acidic wash**<br>**Thioglycoside glycosylation**<br>**Capping**<br>**Fmoc deprotection**	8 eq. / t (min)<br>T_1 = -40 °C / 5<br>T_2 = -20 °C / 20
Post AGA		**Photocleavage**<br>**NP-HPLC Purification**	

Protected compound **11** (8.2 mg, 3.21 µmol, 24%) was obtained as a colorless oil by purification using preparative NP-HPLC (**Method N1**).

^{1}H NMR (600 MHz, $CDCl_3$, 50 °C): δ 8.03 – 7.99 (m, 2H), 7.92 (ddd, J = 9.9, 8.3, 1.4 Hz, 4H), 7.59 – 7.48 (m, 4H), 7.44 (t, J = 7.8 Hz, 3H), 7.42 – 7.18 (m, 30H), 7.19 – 7.13 (m, 4H), 7.11 – 7.07 (m, 3H), 6.57 (d, J = 8.1 Hz, 1H), 5.55 – 5.47 (m, 1H), 5.40 (d, J = 3.5 Hz, 1H), 5.36 (dd, J = 10.0, 8.0 Hz, 1H), 5.34 – 5.27 (m, 2H), 5.25 (d, J = 6.7 Hz, 1H), 5.20 – 5.12 (m, 2H), 5.06 (s, 2H), 4.86 (d, J = 11.8 Hz, 1H), 4.83 (d, J = 11.0 Hz, 1H), 4.75 – 4.65 (m, 6H), 4.63 (d, J = 2.6 Hz, 1H), 4.61 (d, J = 1.6 Hz, 1H), 4.54 (d, J = 12.2 Hz, 1H), 4.50 – 4.46 (m, 2H), 4.43 (d, J = 12.0 Hz, 1H), 4.38 (d, J = 11.7 Hz, 1H), 4.35 – 4.28 (m, 3H), 4.28 – 4.22 (m, 1H), 4.11 (dd, J = 12.5, 4.3 Hz, 1H), 4.04 (t, J = 8.6 Hz, 1H), 3.97 – 3.89 (m, 2H), 3.88 (d, J = 3.6 Hz, 1H), 3.86 – 3.79 (m, 2H), 3.77 – 3.68 (m, 4H), 3.67 – 3.62 (m, 3H), 3.58 – 3.42 (m, 8H), 3.39 – 3.31 (m, 3H), 3.31 – 3.23 (m, 2H), 2.93 – 2.86 (m, 2H), 2.73 (dd, J = 13.0, 5.5 Hz, 1H), 2.69 (dd, J = 10.4, 6.4 Hz, 1H), 2.33 – 2.23 (m, 2H), 2.09 (s, 3H), 2.03 (s, 3H), 2.03 (s, 3H), 2.00 (s, 3H), 1.99 (s, 3H), 1.91 –

1.82 (m, 1H), 1.64 (d, J = 33.9 Hz, 6H), 1.48 – 1.34 (m, 2H), 1.33 – 1.21 (m, 2H), 1.19 – 1.09 (m, 2H).

^{13}C NMR (151 MHz, $CDCl_3$, 50 °C): δ 171.2, 170.6, 169.9, 169.8, 169.6, 166.6, 166.5, 165.2, 164.9, 161.6, 156.4, 154.4, 138.9, 138.5, 138.5, 138.5, 138.3, 138.0, 137.0, 133.5, 133.5, 133.1, 130.4, 130.1, 130.0, 130.0, 130.0, 129.9, 129.9, 129.8, 128.8, 128.7, 128.7, 128.6, 128.6, 128.6, 128.6, 128.5, 128.3, 128.2, 128.2, 128.2, 128.1, 128.1, 128.0, 128.0, 127.9, 127.9, 127.9, 127.8, 127.4, 127.3, 101.3, 100.6, 100.3, 99.3, 98.5, 92.4, 80.2, 78.0, 77.3, 77.0, 76.8, 76.4, 76.3, 75.7, 75.5, 75.4, 75.1, 74.8, 74.0, 73.9, 73.7, 73.6, 73.6, 73.6, 73.6, 73.5, 73.2, 72.7, 72.7, 70.3, 69.5, 69.4, 68.9, 68.6, 68.6, 68.5, 67.9, 66.7, 64.3, 63.1, 63.1, 62.2, 62.1, 57.1, 53.2, 44.9, 41.0, 37.8, 29.8, 29.6, 29.1, 27.0, 23.8, 23.8, 23.7, 23.3, 23.2, 21.1, 21.0, 20.8, 20.8.

HRMS (QToF): Calcd for $C_{129}H_{142}Cl_5N_3NaO_{40}$ $[M + Na]^+$ 2570.7510; found 2570.7556.

Crude analytical NP-HPLC (t_R = 30.7 min)

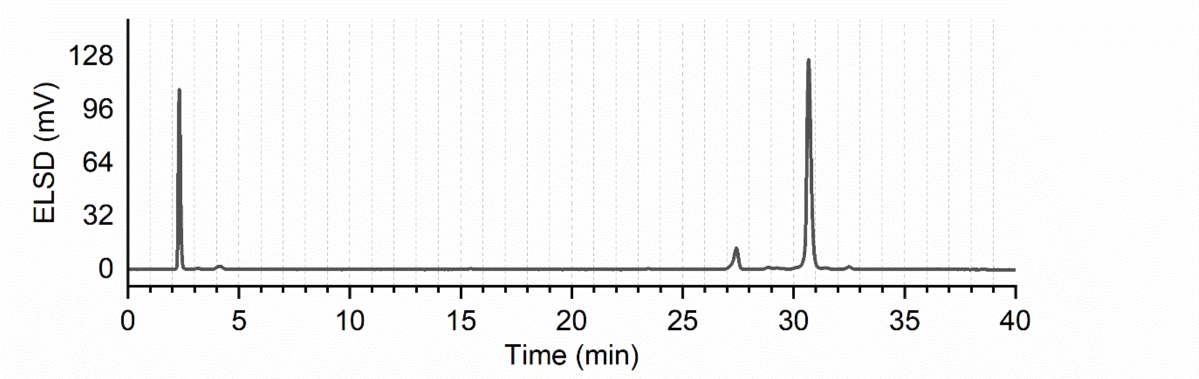

^{1}H-NMR (600 MHz, $CDCl_3$, 50 °C)

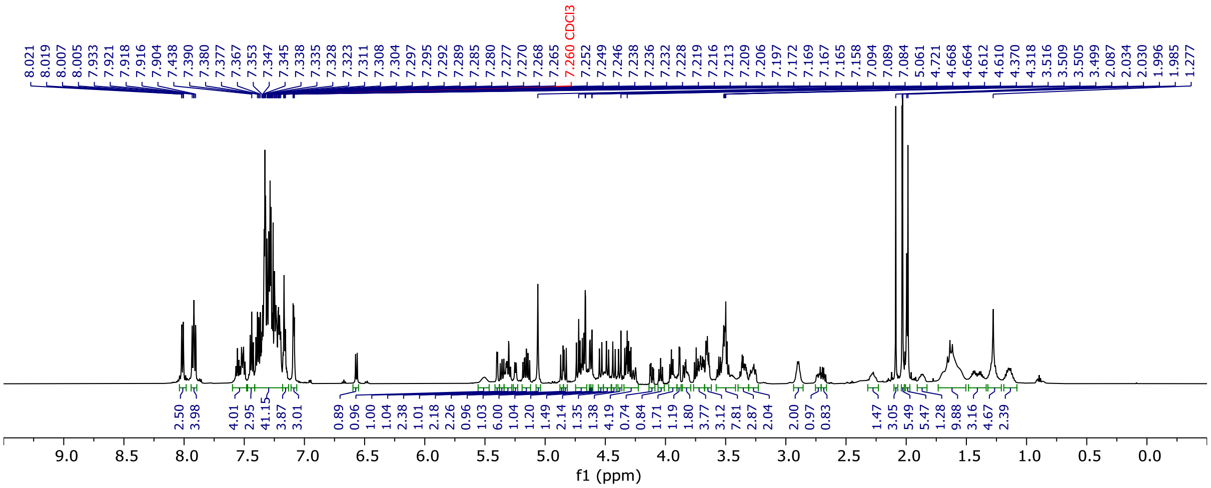

^{13}C-NMR (151 MHz, $CDCl_3$, 50 °C)

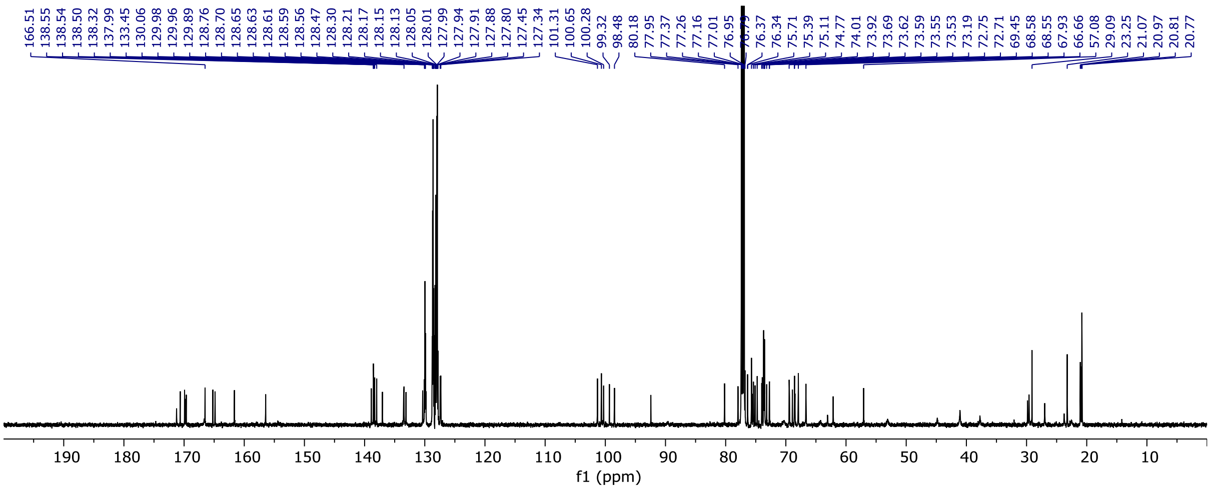

^{1}H -^{1}H COSY

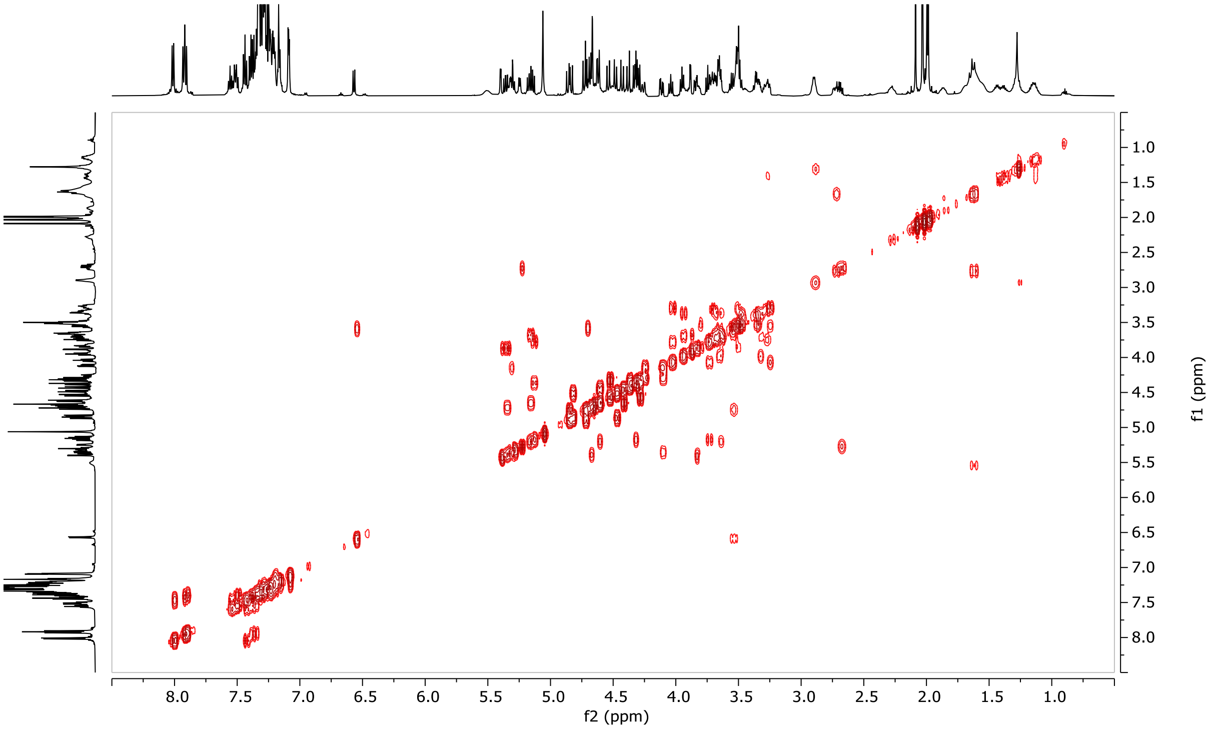

^{13}C-^{1}H HSQC

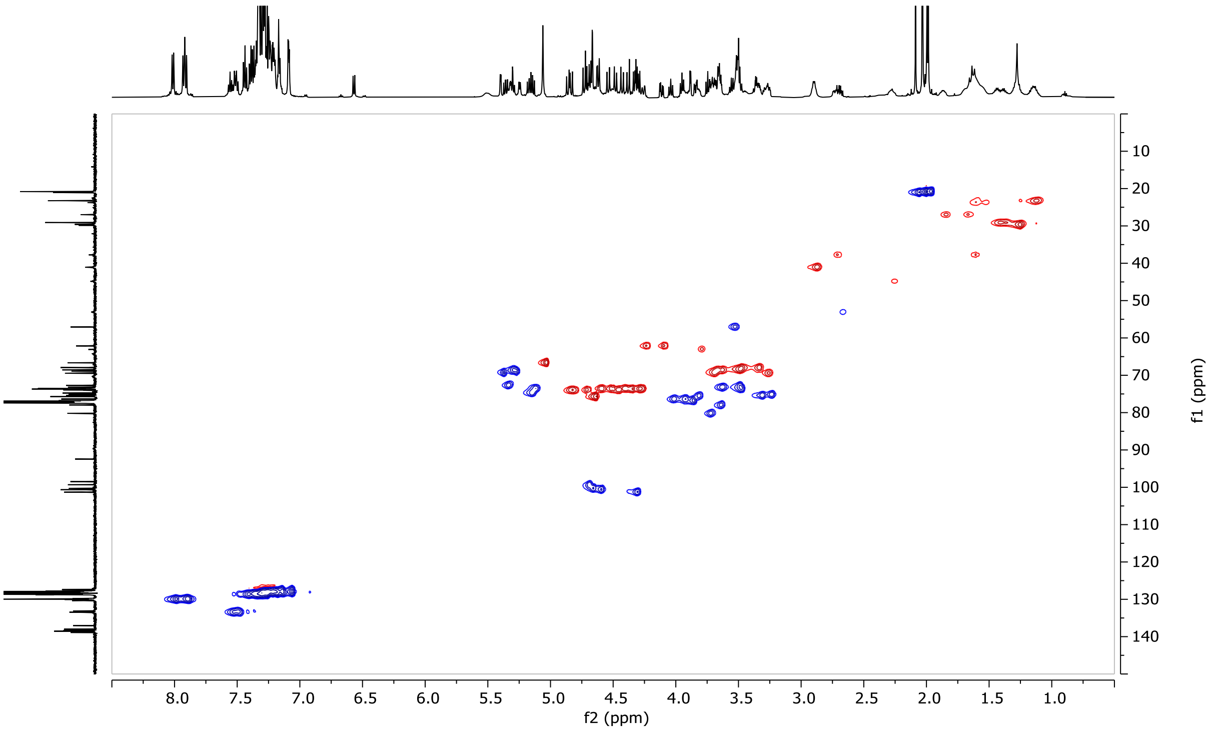

^{13}C-^{1}H HMBC

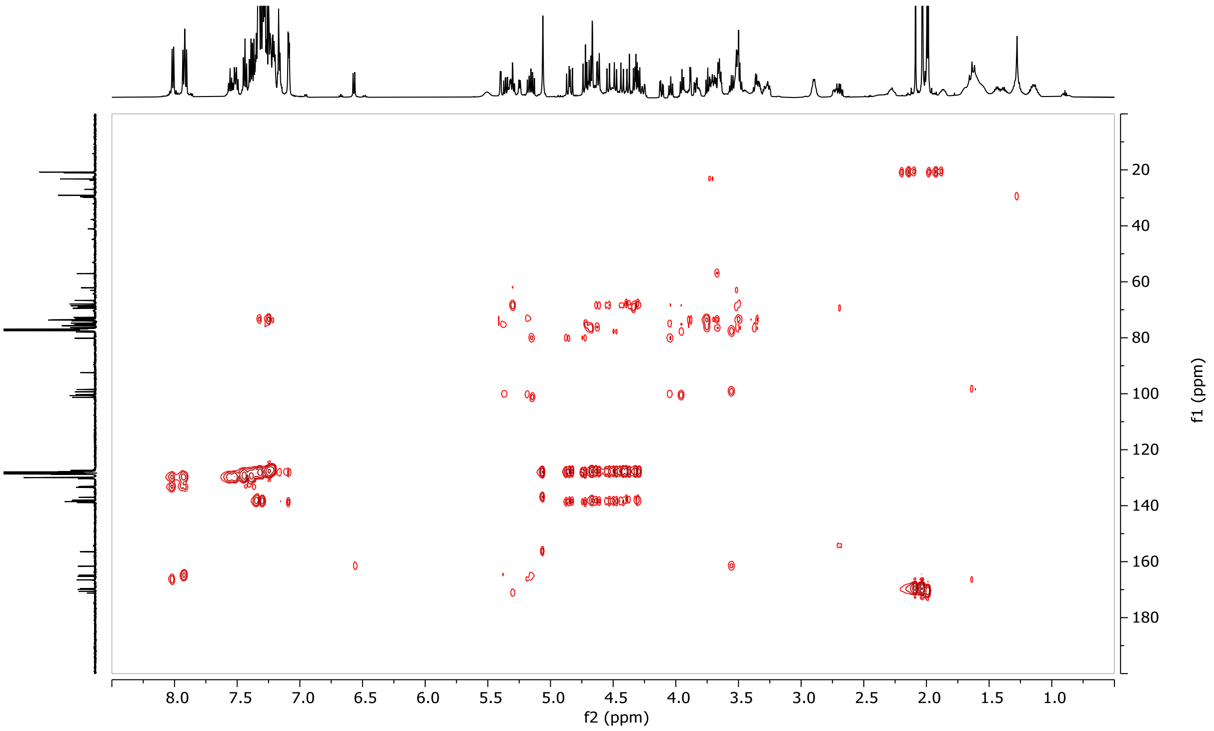

5.6 AGA of Pentasaccharide **12**

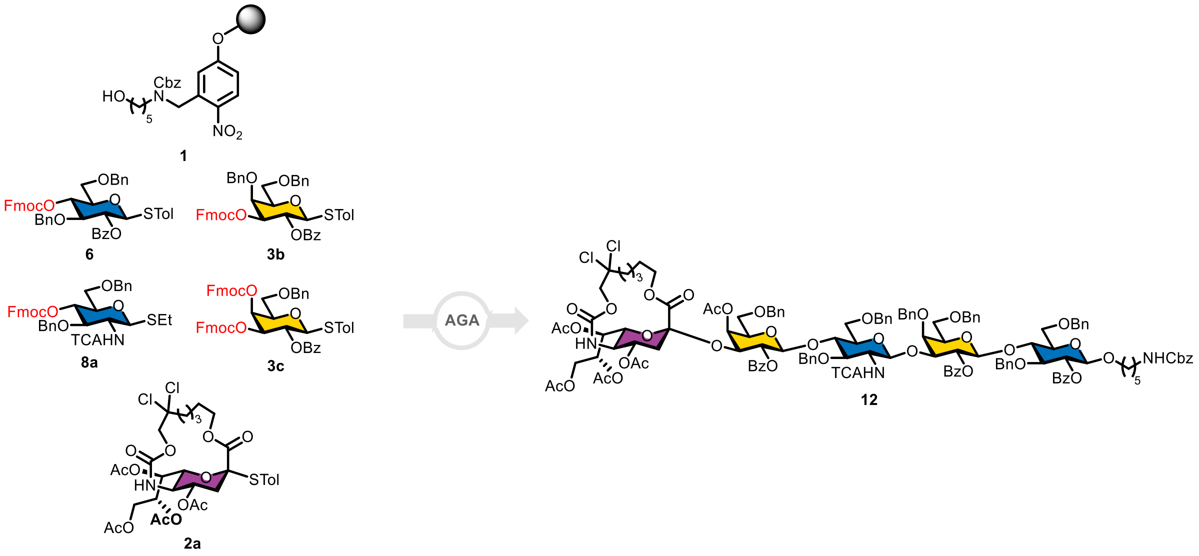

	Building blocks	Modules	Glycosylation condition
AGA		**Initiation** (40 mg resin)	
	Glc 6	Acidic wash<br>Thioglycoside glycosylation<br>Capping<br>Fmoc deprotection	8 eq. t (min)<br>T_1 = -20 °C 5<br>T_2 = 0 °C 20
	Gal 3b	Acidic wash<br>Thioglycoside glycosylation<br>Capping<br>Fmoc deprotection	8 eq. t (min)<br>T_1 = -40 °C 5<br>T_2 = -20 °C 20
	GlcN 8a	Acidic wash<br>Thioglycoside glycosylation<br>Capping<br>Fmoc deprotection	8 eq. t (min)<br>T_1 = -20 °C 5<br>T_2 = 0 °C 40
	Gal 3c	Acidic wash<br>Thioglycoside glycosylation<br>Capping<br>Fmoc deprotection	8 eq. t (min)<br>T_1 = -20 °C 5<br>T_2 = 0 °C 20
	Sia 2a	Acidic wash<br>Thioglycoside glycosylation<br>Pyridine wash<br>} x4	10 eq. t (min)<br>T_1 = -40 °C 30<br>T_2 = -20 °C 10
Post AGA		**Photocleavage**<br>**Capping** (Ac_2O/pyridine = 1/1)<br>**NP-HPLC Purification**	

Protected compound **12** (8.4 mg, 3.19 µmol, 24%) was obtained as a colorless oil by purification using preparative NP-HPLC (**Method N1**).

^{1}**H NMR** (600 MHz, $CDCl_3$, 50 °C): δ 8.24 (dt, J = 6.9, 1.5 Hz, 2H), 7.95 (dd, J = 8.3, 1.4 Hz, 2H), 7.92 (dd, J = 8.3, 1.4 Hz, 2H), 7.55 – 7.46 (m, 6H), 7.39 (dtd, J = 14.6, 7.4, 1.7 Hz, 5H), 7.33 (d, J = 5.1 Hz, 5H), 7.31 – 7.17 (m, 23H), 7.17 – 7.11 (m, 5H), 7.10 – 7.06 (m, 2H), 7.04 – 7.00 (m, 1H), 6.95 (t, J = 7.4 Hz, 2H), 6.56 (d, J = 8.1 Hz, 1H), 5.67 (ddd, J = 10.1, 4.9, 2.2 Hz, 1H), 5.57 – 5.47 (m, 2H), 5.26 – 5.19 (m, 2H), 5.19 – 5.10 (m, 2H), 5.10 – 4.99 (m, 3H), 4.96 (d, J = 11.7 Hz, 1H), 4.92 (d, J = 8.0 Hz, 1H), 4.90 (d, J = 11.2 Hz, 1H), 4.84 (d, J = 10.8 Hz, 1H), 4.73 (d, J = 7.3 Hz, 2H), 4.61 – 4.53 (m, 5H), 4.53 – 4.44 (m, 5H), 4.38 (d, J = 11.8 Hz, 1H), 4.34 – 4.22 (m, 6H), 4.20 (d, J = 11.9 Hz, 1H), 4.15 – 4.03 (m, 3H), 4.00 – 3.95 (m, 2H), 3.81 – 3.69 (m, 6H), 3.69 – 3.64 (m, 2H), 3.61 (q, J = 7.8 Hz, 1H), 3.52 (dd, J = 11.1, 4.1 Hz, 1H), 3.47 – 3.39 (m, 4H), 3.37 (dd, J = 8.2, 4.4 Hz, 1H), 3.33 – 3.25 (m, 3H), 3.19 (ddd, J = 9.6, 4.1, 2.1 Hz, 1H), 2.95 – 2.85 (m, 2H), 2.71 – 2.63 (m, 1H), 2.44 (td, J = 10.1, 6.3 Hz, 1H), 2.37 – 2.28 (m, 2H), 2.16 (s, 3H), 2.03 – 2.01 (m, 7H), 1.98 (s, 3H), 1.70 – 1.52 (m, 5H), 1.53 – 1.36 (m, 6H), 1.29 (d, J = 9.4 Hz, 2H), 1.20 – 1.09 (m, 2H).

^{13}**C NMR** (151 MHz, $CDCl_3$, 50 °C): δ 170.6, 170.3, 170.3, 170.2, 169.7, 169.6, 167.4, 165.3, 165.0, 164.7, 161.4, 156.3, 154.5, 139.1, 138.7, 138.4, 138.4, 138.3, 138.3, 138.3, 138.0, 136.9, 133.2, 133.0, 132.8, 130.5, 130.3, 130.3, 130.3, 130.2, 130.0, 129.9, 129.9, 129.8, 129.7, 129.6, 128.7, 128.6, 128.5, 128.4, 128.3, 128.3, 128.3, 128.2, 128.0, 128.0, 127.9, 127.8, 127.8, 127.7, 127.7, 127.6, 127.5, 127.4, 127.4, 127.3, 127.3, 127.1, 126.8, 101.1, 100.7, 100.5, 96.3, 96.3, 92.2, 80.6, 79.3, 76.7, 76.2, 75.7, 75.4, 75.1, 74.9, 74.5, 73.8, 73.7, 73.4, 73.4, 73.1, 72.6, 72.2, 72.1, 71.5, 69.2, 69.1, 69.1, 68.6, 68.1, 68.0, 67.8, 66.9, 66.5, 64.8, 62.3, 57.1, 52.2, 44.3, 40.9, 37.2, 29.6, 29.4, 29.4, 28.9, 23.5, 23.1, 21.3, 20.8, 20.8, 20.6, 20.5, 20.0.

HRMS (QToF): Calcd for $C_{136}H_{148}Cl_5N_3NaO_{40}$ $[M + Na]^+$ 2660.7979; found 2660.8047.

Crude analytical NP-HPLC (t_R = 28.4 min)

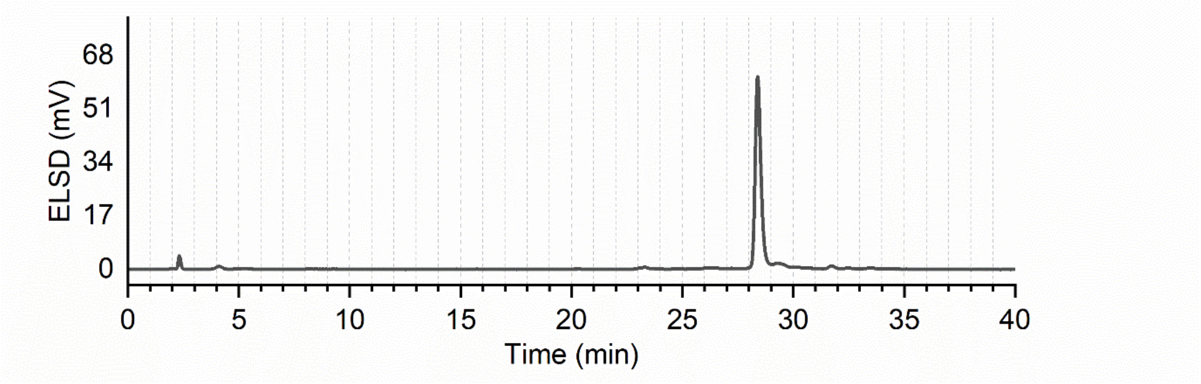

^{1}H-NMR (600 MHz, $CDCl_3$, 50 °C)

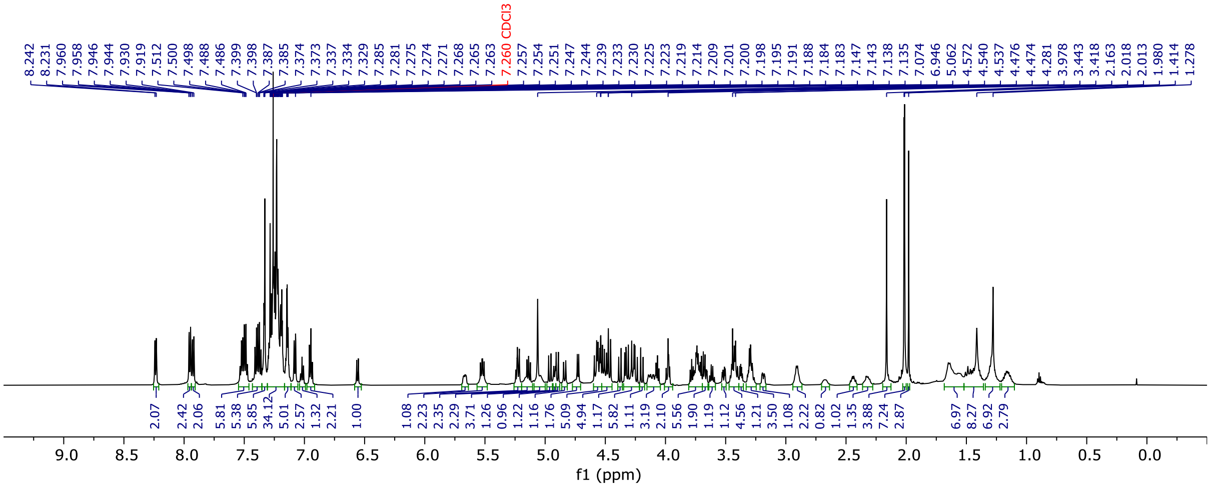

^{13}C-NMR (151 MHz, $CDCl_3$, 50 °C)

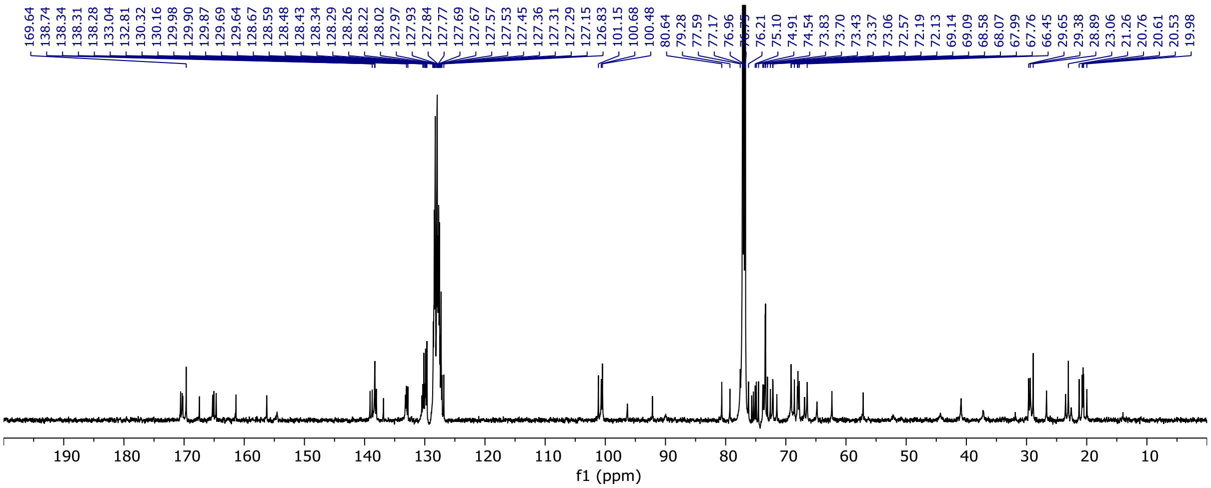

^{1}H -^{1}H COSY

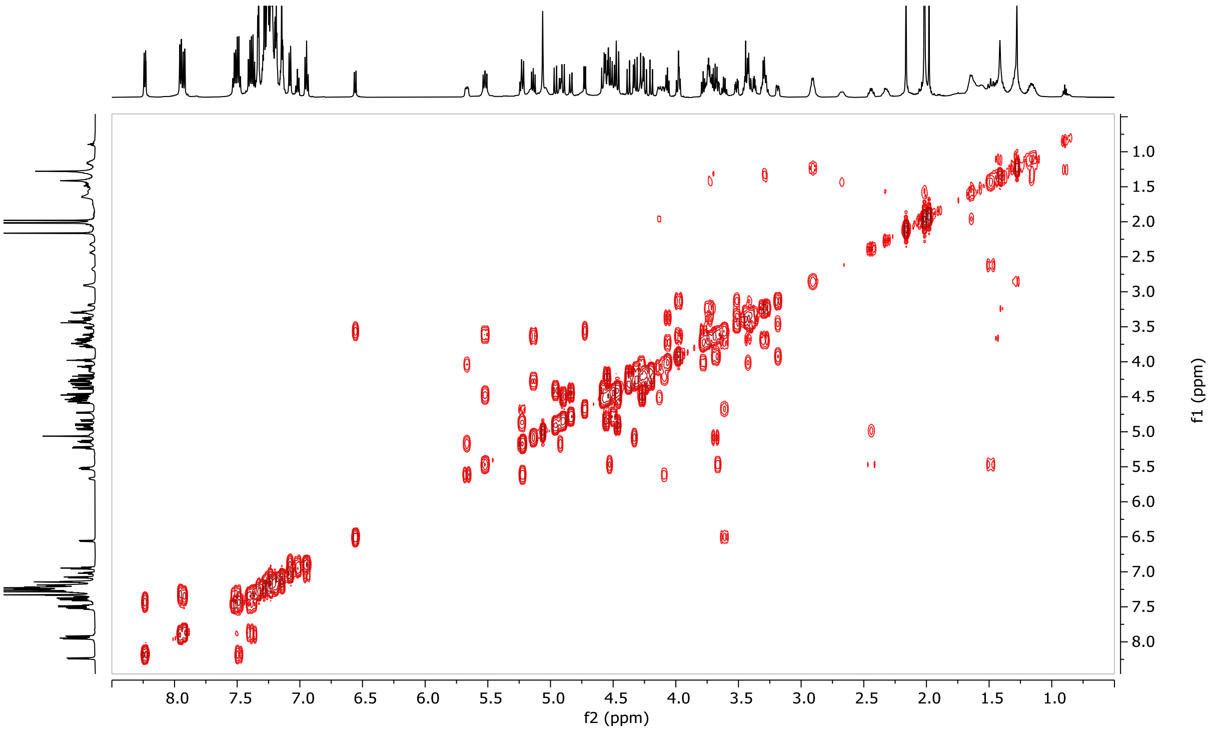

^{13}C-^{1}H HSQC

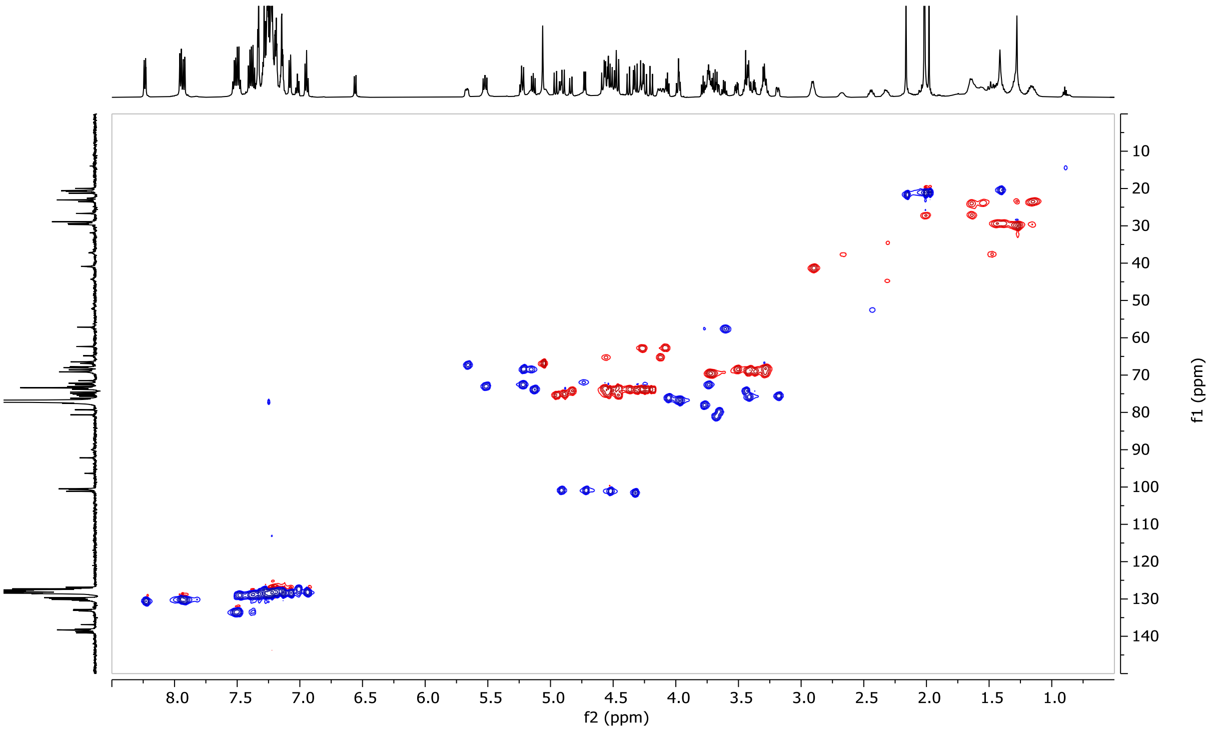

^{13}C-^{1}H HMBC

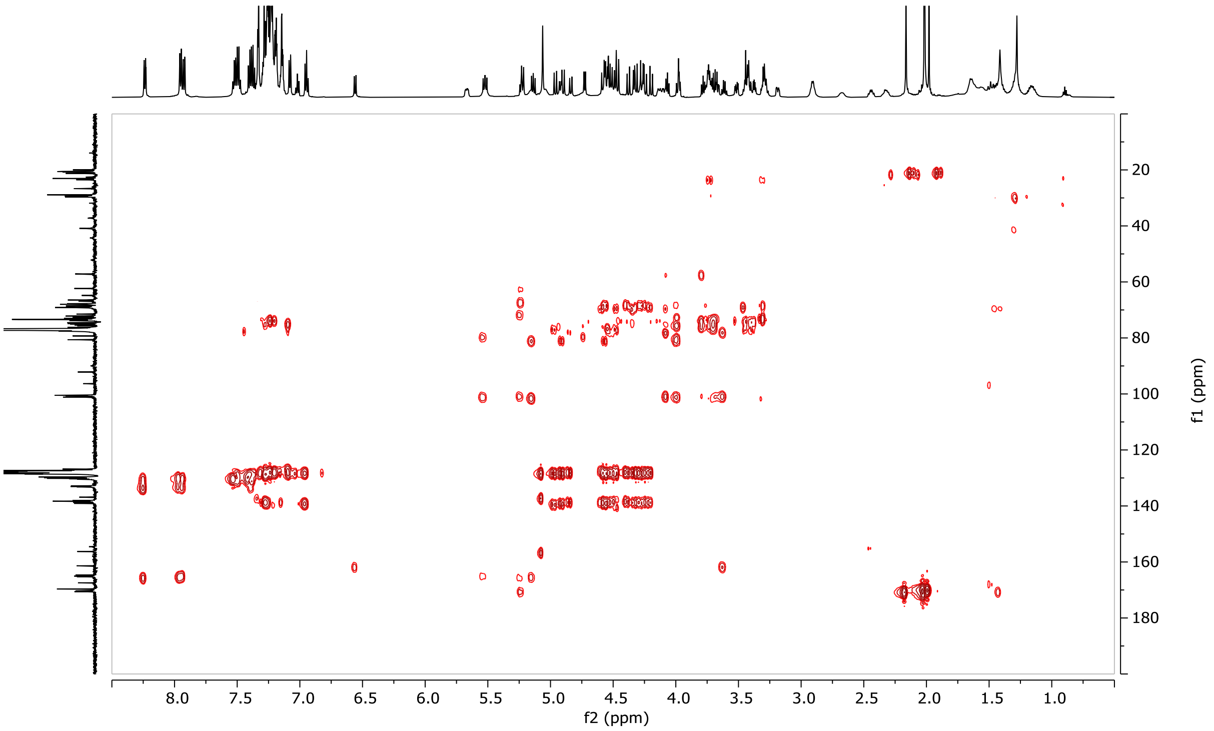

5.7 AGA of Hexasaccharide **13**

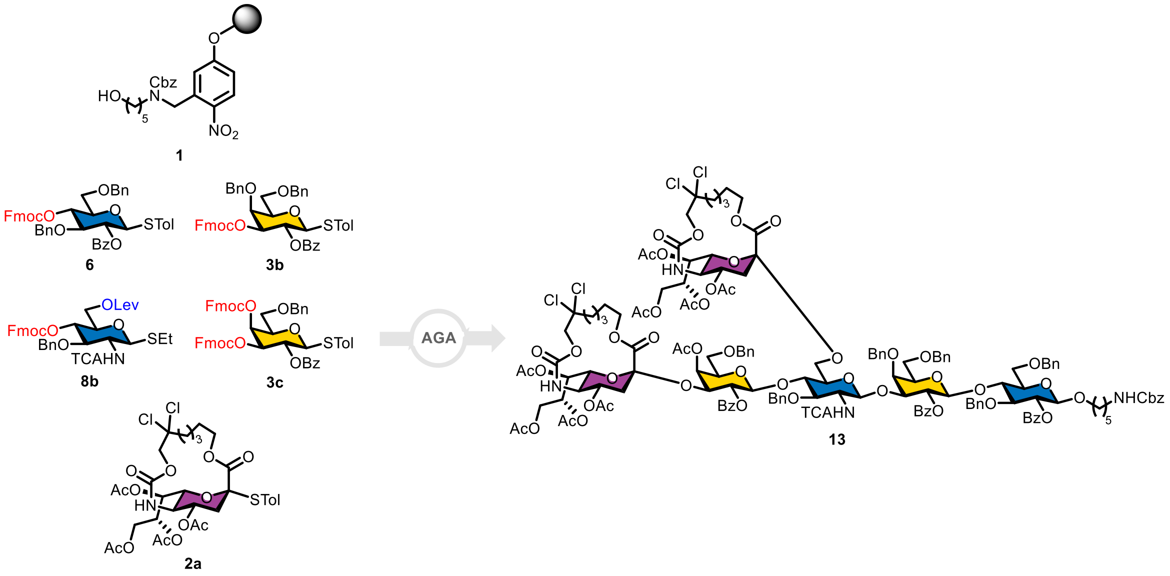

	Building blocks	Modules	Glycosylation condition
AGA		**Initiation** (40 mg resin)	
	**Glc 6**	**Acidic wash**<br>**Thioglycoside glycosylation**<br>**Capping**<br>**Fmoc deprotection**	**8 eq.** / **t (min)**<br>**T_1 = -20 °C** / **5**<br>**T_2 = 0 °C** / **20**
	**Gal 3b**	**Acidic wash**<br>**Thioglycoside glycosylation**<br>**Capping**<br>**Fmoc deprotection**	**8 eq.** / **t (min)**<br>**T_1 = -40 °C** / **5**<br>**T_2 = -20 °C** / **20**
	**GlcN 8b**	**Acidic wash**<br>**Thioglycoside glycosylation**<br>**Capping**<br>**Lev deprotection**	**8 eq.** / **t (min)**<br>**T_1 = -20 °C** / **5**<br>**T_2 = 0 °C** / **40**
	**Sia 2a**	[**Acidic wash**<br>**Thioglycoside glycosylation**<br>**Pyridine wash**] **x2**<br>**Capping**<br>**Fmoc deprotection**	**10 eq.** / **t (min)**<br>**T_1 = -40 °C** / **30**<br>**T_2 = -20 °C** / **10**
	**Gal 3c**	[**Acidic wash**<br>**Thioglycoside glycosylation**<br>**Pyridine wash**] **x2**<br>**Capping**<br>**Fmoc deprotection**	**8 eq.** / **t (min)**<br>**T_1 = -20 °C** / **5**<br>**T_2 = 0 °C** / **20**
	**Sia 2a**	[**Acidic wash**<br>**Thioglycoside glycosylation**<br>**Pyridine wash**] **x4**	**10 eq.** / **t (min)**<br>**T_1 = -40 °C** / **30**<br>**T_2 = -20 °C** / **10**
**Post AGA**		**Photocleavage**<br>**Capping** (Ac_2O/pyridine = 1/1)<br>**NP-HPLC Purification**	

Protected compound **13** (6.2 mg, 1.94 μmol, 15%) was obtained as a colorless oil by purification using preparative NP-HPLC (**Method N1**).

^{1}H NMR (600 MHz, $CDCl_3$, 50 °C): δ 8.23 – 8.16 (m, 2H), 8.06 – 8.00 (m, 2H), 7.97 – 7.92 (m, 2H), 7.55 – 7.41 (m, 9H), 7.37 (t, J = 7.8 Hz, 3H), 7.35 – 7.31 (m, 6H), 7.31 – 7.10 (m, 21H), 7.10 – 7.04 (m, 2H), 7.03 – 6.99 (m, 1H), 6.93 (dd, J = 8.2, 6.9 Hz, 2H), 6.71 (d, J = 9.1 Hz, 1H), 5.62 – 5.58 (m, 1H), 5.57 – 5.47 (m, 3H), 5.42 (ddd, J = 8.9, 4.2, 2.4 Hz, 1H), 5.39 (dd, J = 8.8, 1.6 Hz, 1H), 5.26 (dd, J = 10.0, 2.1 Hz, 1H), 5.24 – 5.19 (m, 1H), 5.18 – 5.12 (m, 2H), 5.10 (dd, J = 10.1, 7.9 Hz, 1H), 5.06 (s, 2H), 5.04 – 4.99 (m, 1H), 4.96 (d, J = 11.7 Hz, 1H), 4.94 (d, J = 11.3 Hz, 1H), 4.83 (d, J = 7.6 Hz, 1H), 4.76 – 4.68 (m, 1H), 4.65 (d, J = 11.7 Hz, 1H), 4.63 – 4.46 (m, 9H), 4.44 (d, J = 11.8 Hz, 2H), 4.39 (d, J = 11.7 Hz, 1H), 4.36 – 4.28 (m, 6H), 4.28 – 4.22 (m, 2H), 4.18 (d, J = 12.2 Hz, 1H), 4.15

– 4.09 (m, 1H), 4.07 – 3.99 (m, 2H), 3.97 (t, J = 9.1 Hz, 1H), 3.93 – 3.88 (m, 2H), 3.80 – 3.66 (m, 6H), 3.65 – 3.60 (m, 2H), 3.58 (dd, J = 11.3, 4.5 Hz, 2H), 3.51 (dd, J = 11.3, 2.1 Hz, 2H), 3.47 – 3.37 (m, 5H), 3.33 – 3.23 (m, 5H), 2.94 – 2.88 (m, 2H), 2.88 – 2.80 (m, 2H), 2.73 – 2.64 (m, 1H), 2.48 – 2.39 (m, 2H), 2.38 – 2.28 (m, 2H), 2.18 (s, 3H), 2.10 (s, 3H), 2.06 (s, 3H), 2.04 – 2.00 (m, 8H), 1.99 (s, 3H), 1.99 (s, 3H), 1.97 (s, 3H), 1.79 – 1.70 (m, 3H), 1.67 – 1.35 (m, 14H), 1.34 – 1.21 (m, 2H), 1.21 – 1.10 (m, 2H).

^{13}C NMR (151 MHz, $CDCl_3$, 50 °C): δ 171.0, 170.8, 170.7, 170.4, 170.2, 169.8, 169.8, 169.6, 167.7, 166.8, 165.9, 165.3, 164.9, 161.5, 156.5, 154.7, 139.4, 139.0, 138.7, 138.7, 138.1, 138.1, 137.1, 133.7, 133.2, 133.0, 130.8, 130.5, 130.3, 130.2, 129.9, 129.1, 128.8, 128.6, 128.5, 128.5, 128.3, 128.2, 128.2, 128.2, 128.0, 127.8, 127.8, 127.6, 127.5, 127.3, 127.0, 101.9, 101.7, 101.4, 100.8, 98.9, 96.5, 92.3, 81.0, 80.4, 77.4, 76.6, 75.5, 74.9, 74.8, 73.7, 73.6, 73.6, 72.7, 72.3, 72.1, 72.1, 71.4, 69.6, 69.4, 68.9, 68.4, 68.3, 68.2, 68.0, 67.8, 67.1, 66.7, 65.1, 64.3, 62.5, 62.2, 54.6, 52.4, 44.6, 41.1, 37.9, 37.3, 29.9, 29.7, 29.6, 29.6, 29.1, 27.0, 26.9, 23.7, 23.3, 21.5, 21.1, 21.0, 20.9, 20.8, 20.8, 20.8, 20.2.

HRMS (QToF): Calcd for $C_{154}H_{175}Cl_7N_4Na_2O_{53}$ $[M + 2Na]^{2+}$ 1609.4369; found 1609.4362.

Crude analytical NP-HPLC (t_R = 30.0 min)

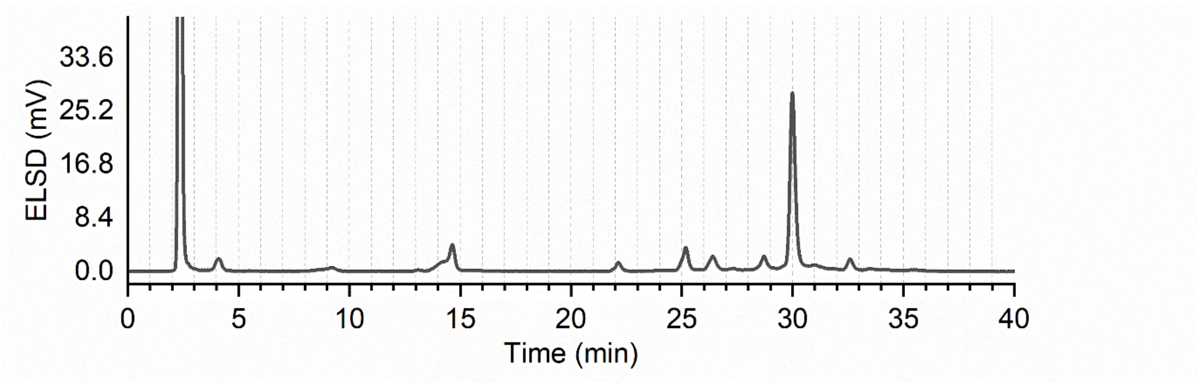

^{1}H-NMR (600 MHz, $CDCl_3$, 50 °C)

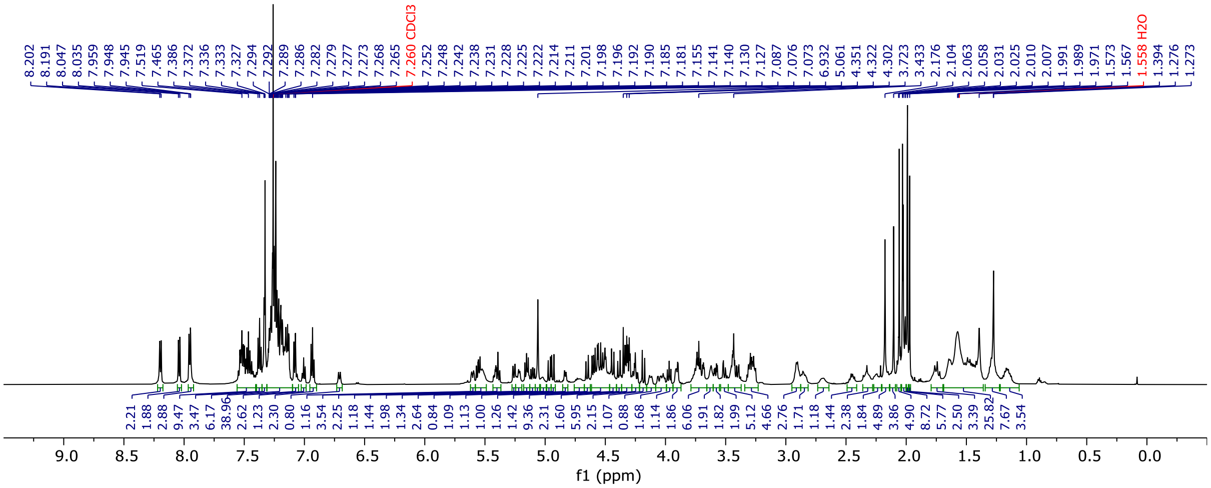

^{13}C-NMR (151 MHz, $CDCl_3$, 50 °C)

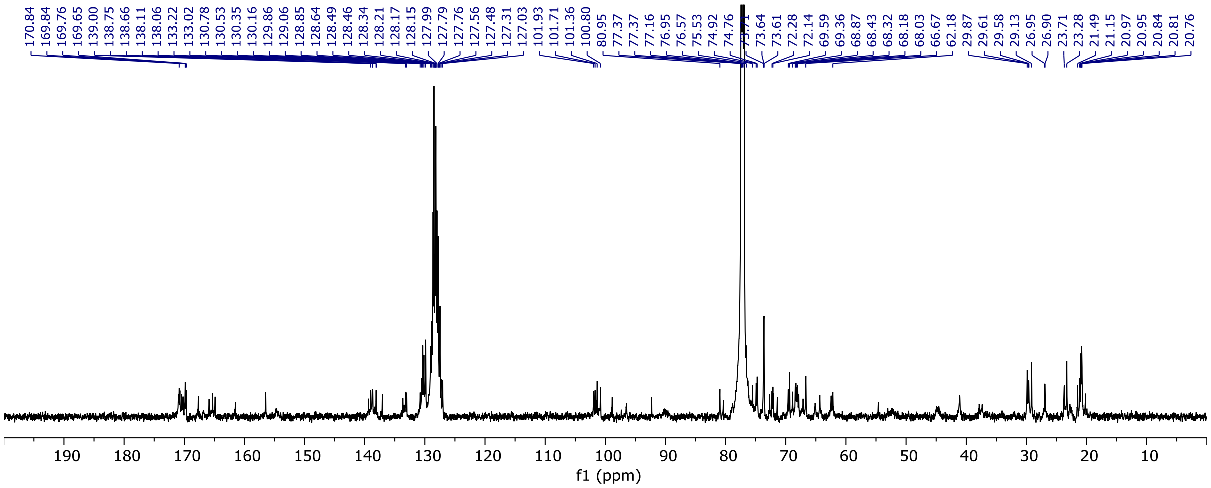

^{1}H -^{1}H COSY

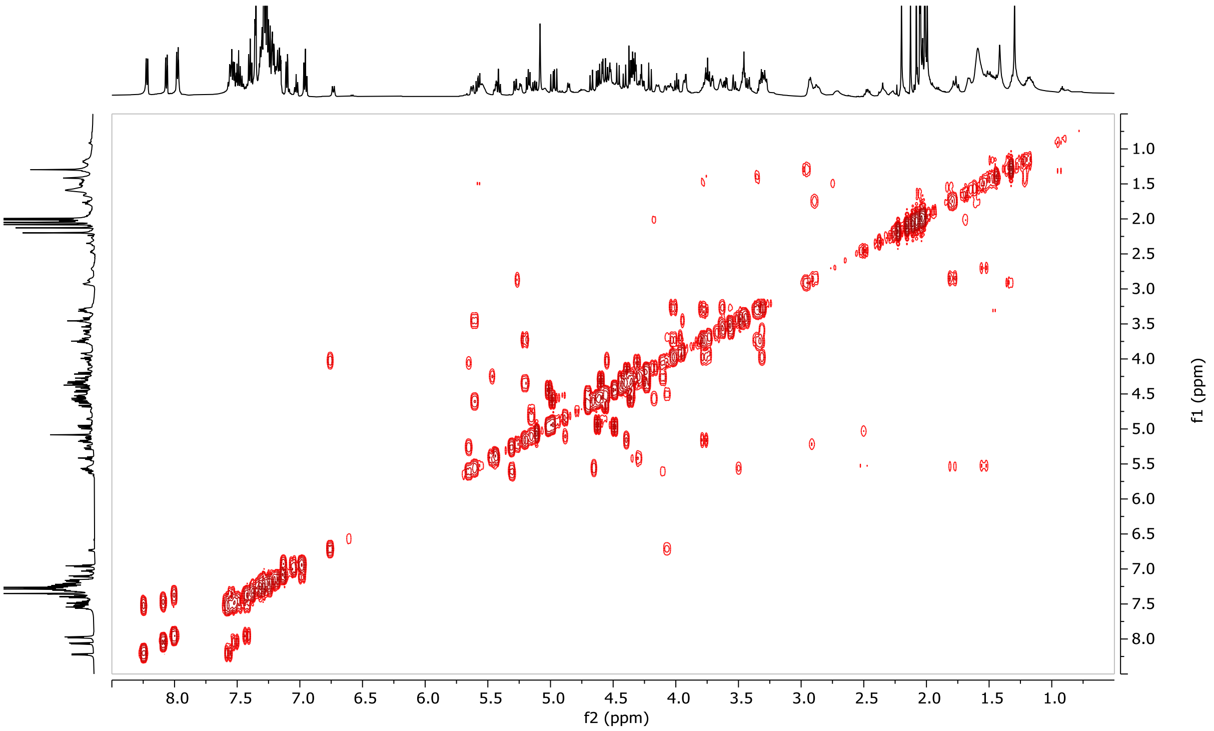

^{13}C-^{1}H HSQC

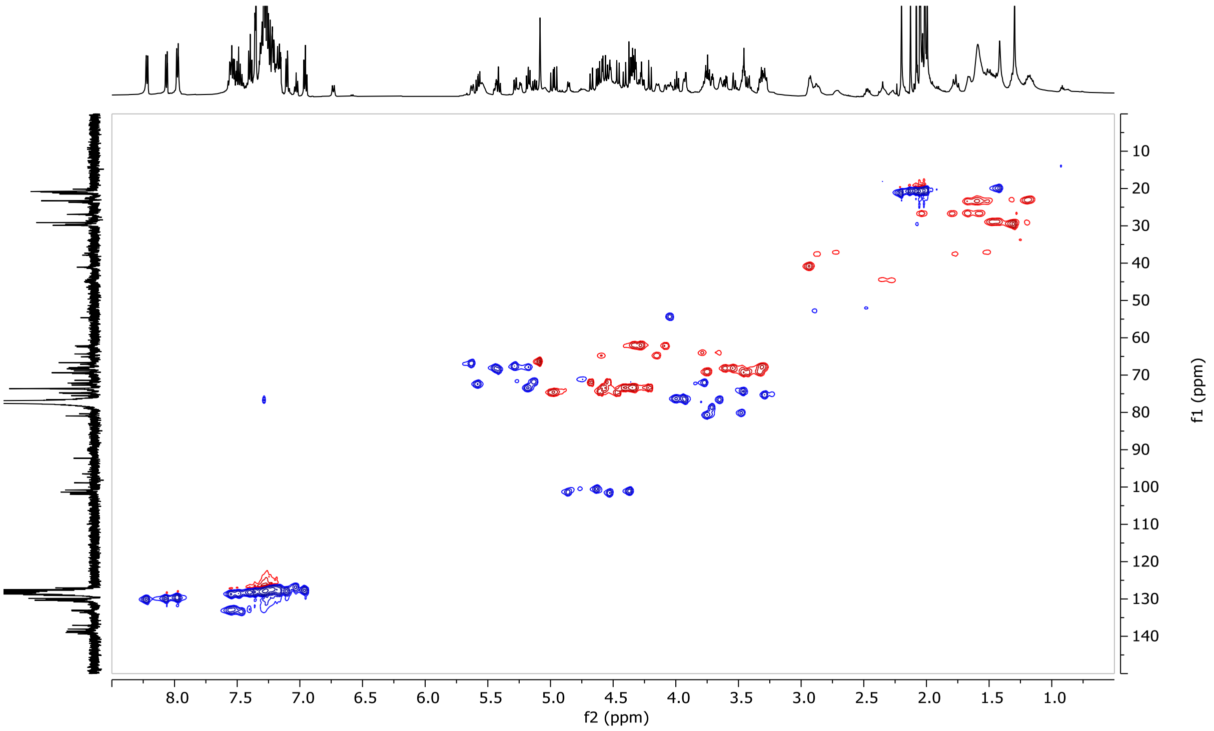

^{13}C-^{1}H HMBC

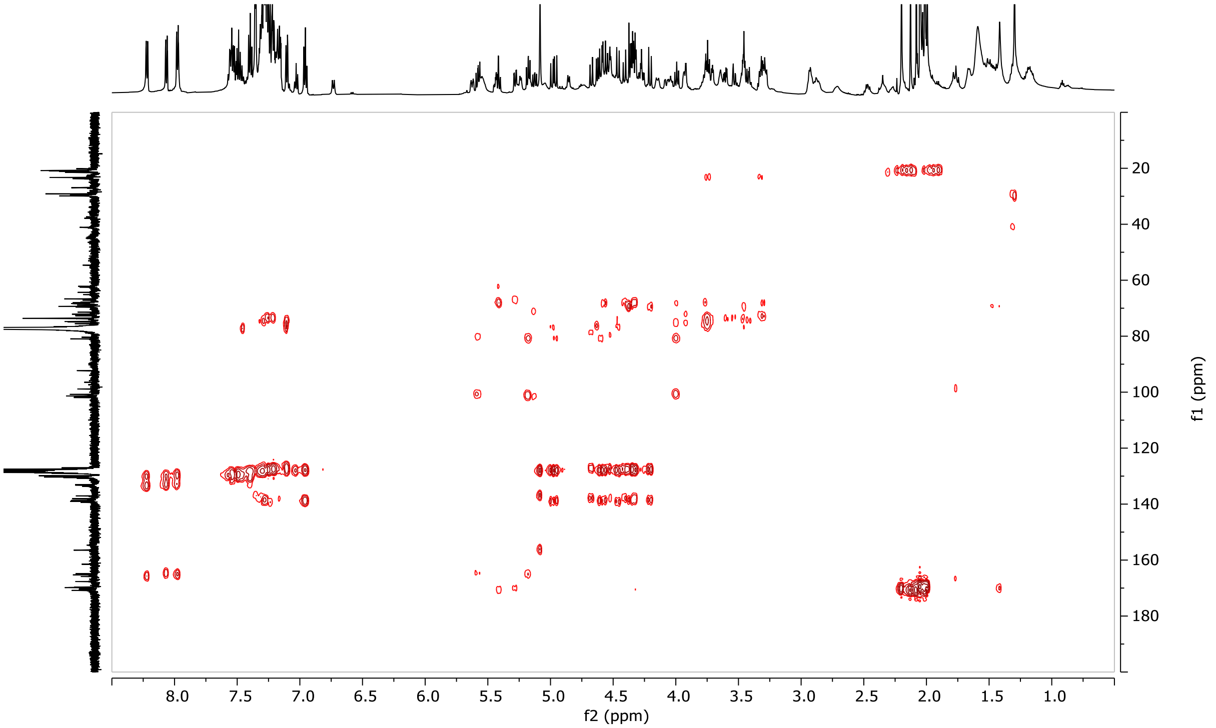

5.8 AGA of Hexasaccharide **14**

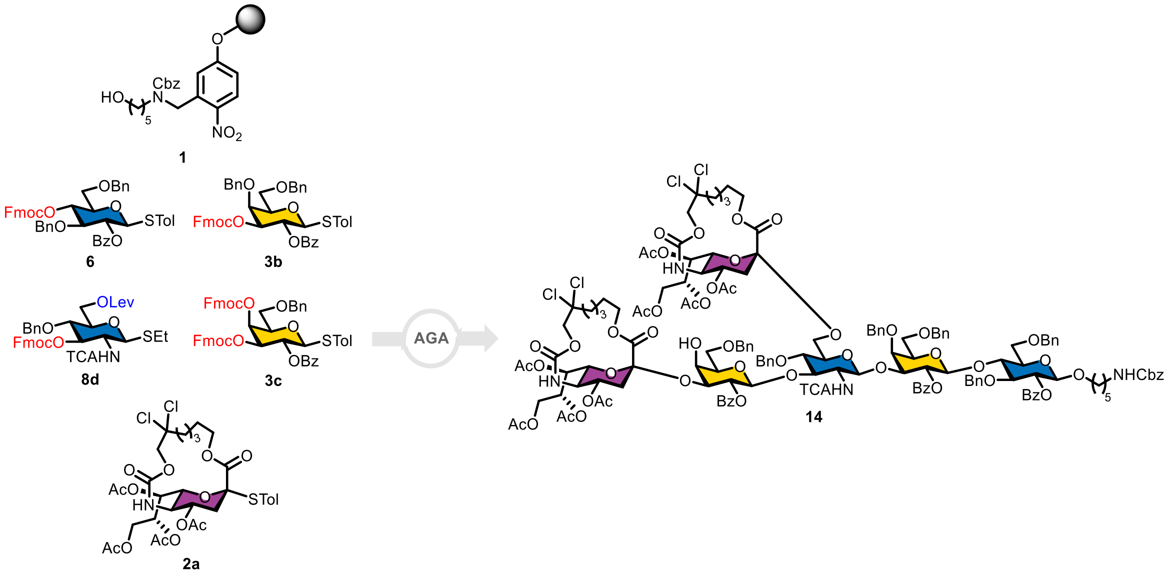

	Building blocks	Modules	Glycosylation condition
AGA		**Initiation** (40 mg resin)	
	Glc 6	Acidic wash<br>Thioglycoside glycosylation<br>Capping<br>Fmoc deprotection	8 eq.; t (min)<br>T_1 = -20 °C; 5<br>T_2 = 0 °C; 20
	Gal 3b	Acidic wash<br>Thioglycoside glycosylation<br>Capping<br>Fmoc deprotection	8 eq.; t (min)<br>T_1 = -40 °C; 5<br>T_2 = -20 °C; 20
	GlcN 8d	Acidic wash<br>Thioglycoside glycosylation<br>Capping<br>Lev deprotection	8 eq.; t (min)<br>T_1 = -20 °C; 5<br>T_2 = 0 °C; 40
	Sia 2a	Acidic wash<br>Thioglycoside glycosylation<br>Pyridine wash (x2)<br>Capping<br>Fmoc deprotection	10 eq.; t (min)<br>T_1 = -40 °C; 30<br>T_2 = -20 °C; 10
	Gal 3c	Acidic wash<br>Thioglycoside glycosylation<br>Capping<br>Fmoc deprotection	8 eq.; t (min)<br>T_1 = -20 °C; 5<br>T_2 = 0 °C; 20
	Sia 2a	Acidic wash<br>Thioglycoside glycosylation<br>Pyridine wash (x2)<br>Capping	10 eq.; t (min)<br>T_1 = -40 °C; 30<br>T_2 = -20 °C; 10
Post AGA		Photocleavage<br>NP-HPLC Purification	

Protected compound **14** (7.5 mg, 2.38 μmol, 18%) was obtained as a colorless oil by purification using preparative NP-HPLC (**Method N1**).

^{1}H NMR (700 MHz, $CDCl_3$, 50 °C): δ 8.10 – 8.07 (m, 2H), 7.96 – 7.93 (m, 2H), 7.93 – 7.88 (m, 2H), 7.54 – 7.46 (m, 3H), 7.44 – 7.41 (m, 3H), 7.41 – 7.35 (m, 6H), 7.35 – 7.31 (m, 8H), 7.30 – 7.25 (m, 6H), 7.25 – 7.20 (m, 7H), 7.20 – 7.11 (m, 6H), 7.10 – 7.04 (m, 2H), 7.03 – 6.99 (m, 1H), 6.93 (t, *J* = 7.5 Hz, 2H), 6.39 (d, *J* = 7.9 Hz, 1H), 5.58 – 5.50 (m, 1H), 5.46 (dd, *J* = 10.1, 7.8 Hz, 1H), 5.40 (dt, *J* = 9.7, 3.2 Hz, 3H), 5.35 – 5.26 (m, 2H), 5.24 – 5.16 (m, 2H), 5.12 (dd, *J* = 9.3, 7.9 Hz, 1H), 5.09 – 5.02 (m, 3H), 4.90 (d, *J* = 11.4 Hz, 2H), 4.83 (d, *J* = 7.9 Hz, 1H), 4.80 – 4.74 (m, 2H), 4.73 – 4.60 (m, 4H), 4.59 – 4.42 (m, 7H), 4.41 – 4.26 (m, 9H), 4.22 – 4.14 (m, 5H), 4.01 (dd, *J* = 13.1, 3.8 Hz, 1H), 3.98 (d, *J* = 8.5 Hz, 1H), 3.91 (d, *J* = 3.1 Hz, 1H), 3.84 (dd, *J* = 10.1, 3.0 Hz, 1H), 3.81 – 3.75 (m,

1H), 3.71 (dt, J = 9.7, 6.0 Hz, 2H), 3.66 (t, J = 8.9 Hz, 1H), 3.59 (t, J = 6.4 Hz, 2H), 3.55 – 3.43 (m, 7H), 3.43 – 3.35 (m, 4H), 3.33 – 3.26 (m, 1H), 3.25 – 3.17 (m, 1H), 3.15 (ddd, J = 9.6, 4.1, 2.1 Hz, 1H), 2.94 – 2.83 (m, 3H), 2.78 – 2.71 (m, 1H), 2.67 (td, J = 10.5, 6.5 Hz, 1H), 2.42 (td, J = 10.5, 6.5 Hz, 1H), 2.36 – 2.20 (m, 4H), 2.11 (s, 3H), 2.07 (s, 3H), 2.06 (s, 3H), 2.01 (s, 6H), 1.98 (s, 3H), 1.91 – 1.83 (m, 5H), 1.81 – 1.68 (m, 3H), 1.58 (d, J = 41.7 Hz, 15H), 1.33 – 1.23 (m, 2H), 1.21 – 1.08 (m, 2H).

^{13}C NMR (176 MHz, $CDCl_3$, 50 °C): δ 171.1, 171.0, 170.7, 170.7, 169.9, 169.8, 169.8, 168.2, 165.6, 165.2, 165.1, 161.3, 156.4, 154.4, 139.4, 139.0, 138.8, 138.7, 138.6, 138.5, 137.1, 133.5, 133.3, 133.0, 130.5, 130.4, 130.1, 130.1, 129.9, 129.8, 128.8, 128.7, 128.6, 128.5, 128.4, 128.4, 128.3, 128.2, 128.1, 128.0, 127.9, 127.9, 127.7, 127.7, 127.7, 127.6, 127.6, 127.3, 127.0, 101.3, 100.7, 100.0, 99.5, 98.5, 96.5, 92.3, 89.6, 80.8, 78.5, 76.4, 76.2, 76.1, 75.2, 75.2, 74.7, 74.7, 74.6, 74.4, 74.0, 73.7, 73.6, 73.4, 73.2, 72.6, 71.7, 70.1, 69.3, 69.3, 68.6, 68.4, 68.3, 68.2, 67.9, 67.3, 66.6, 64.3, 64.1, 63.7, 62.4, 62.4, 62.1, 62.0, 59.4, 53.0, 52.5, 44.8, 41.1, 41.1, 38.2, 37.9, 29.8, 29.6, 29.1, 29.1, 26.9, 26.8, 23.5, 23.4, 23.4, 23.3, 23.3, 23.3, 22.4, 21.2, 21.2, 20.9, 20.9, 20.9, 20.8, 20.7, 20.3.

HRMS (QToF): Calcd for $C_{152}H_{173}Cl_7N_4NaO_{52}$ $[M + 2Na]^{2+}$ 1588.4310; found 1588.4308.

Crude analytical NP-HPLC (t_R = 31.0 min)

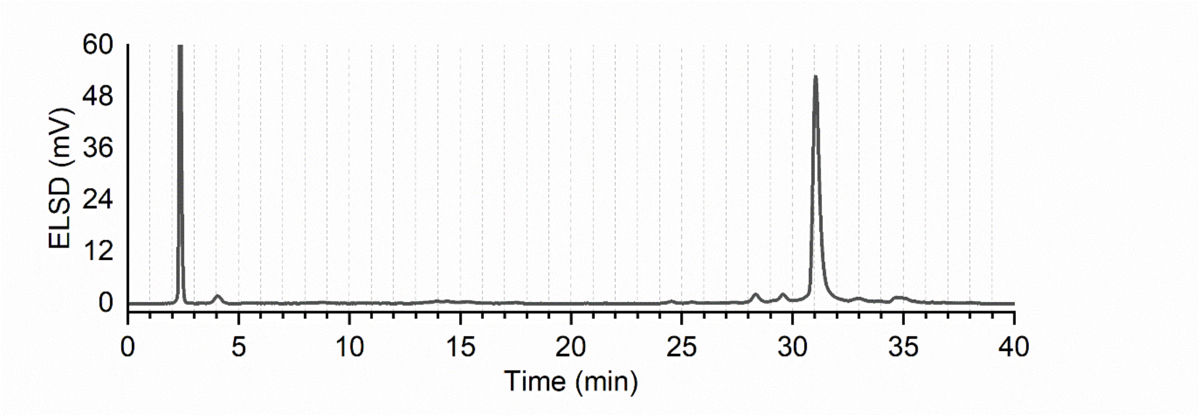

^{1}H-NMR (700 MHz, $CDCl_3$, 50 °C)

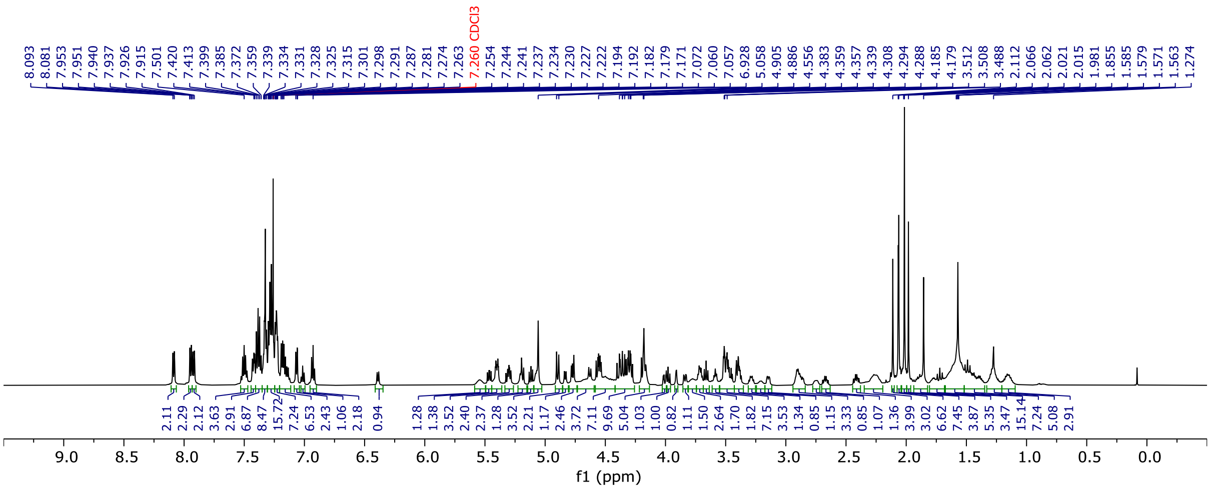

^{13}C-NMR (176 MHz, $CDCl_3$, 50 °C)

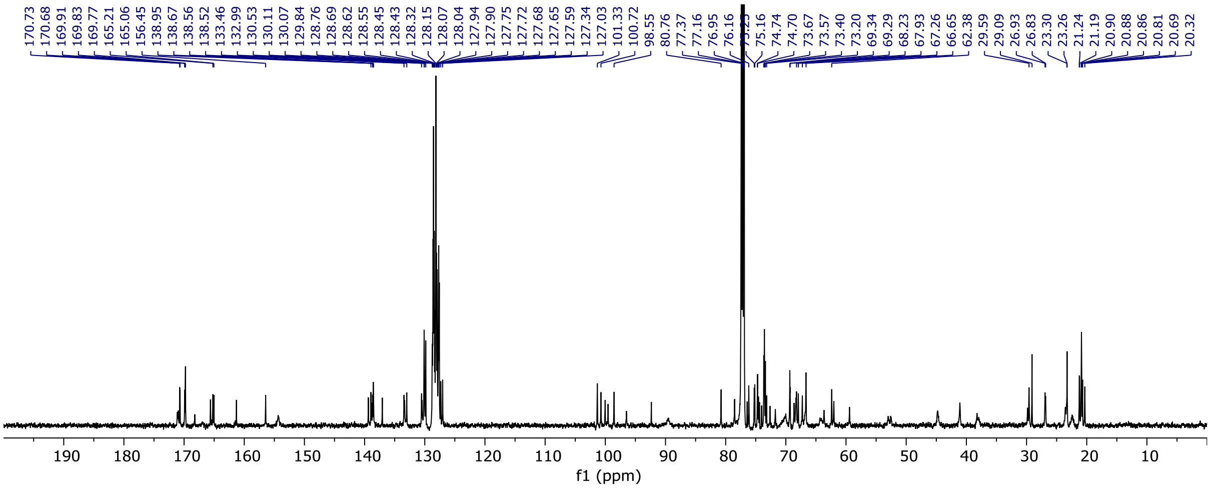

^1H -^1H COSY

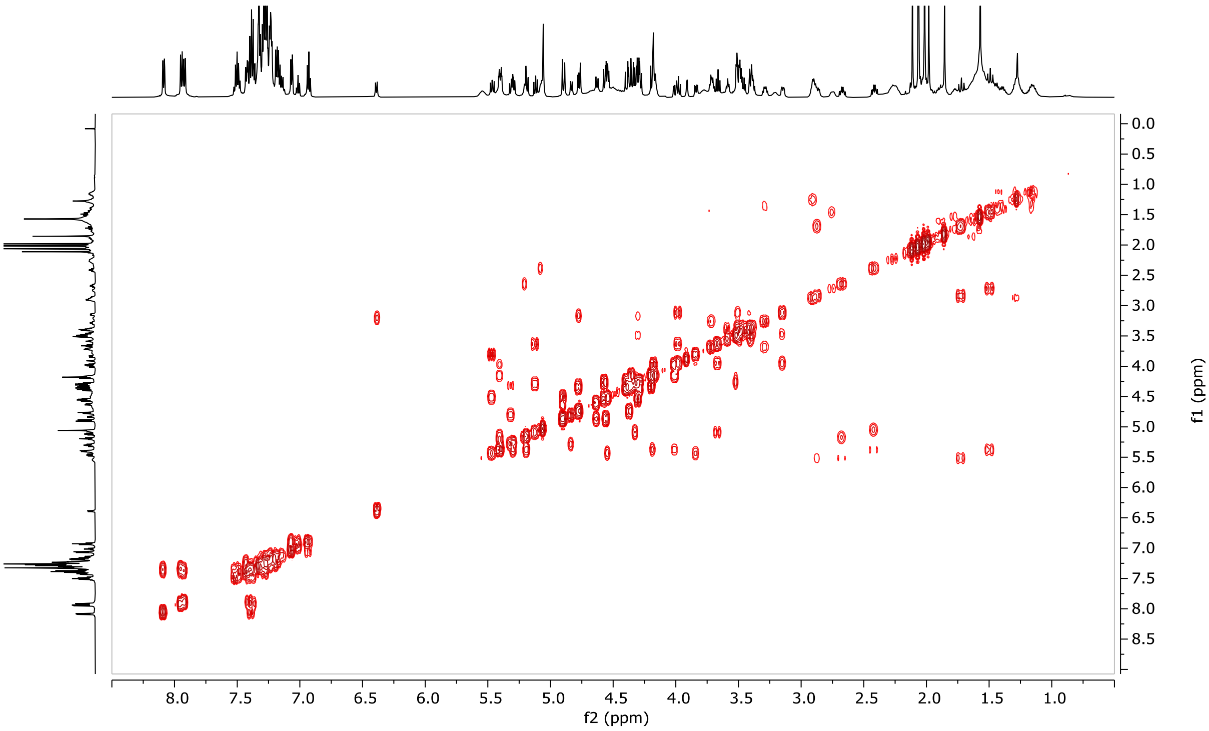

^{13}C-^1H HSQC

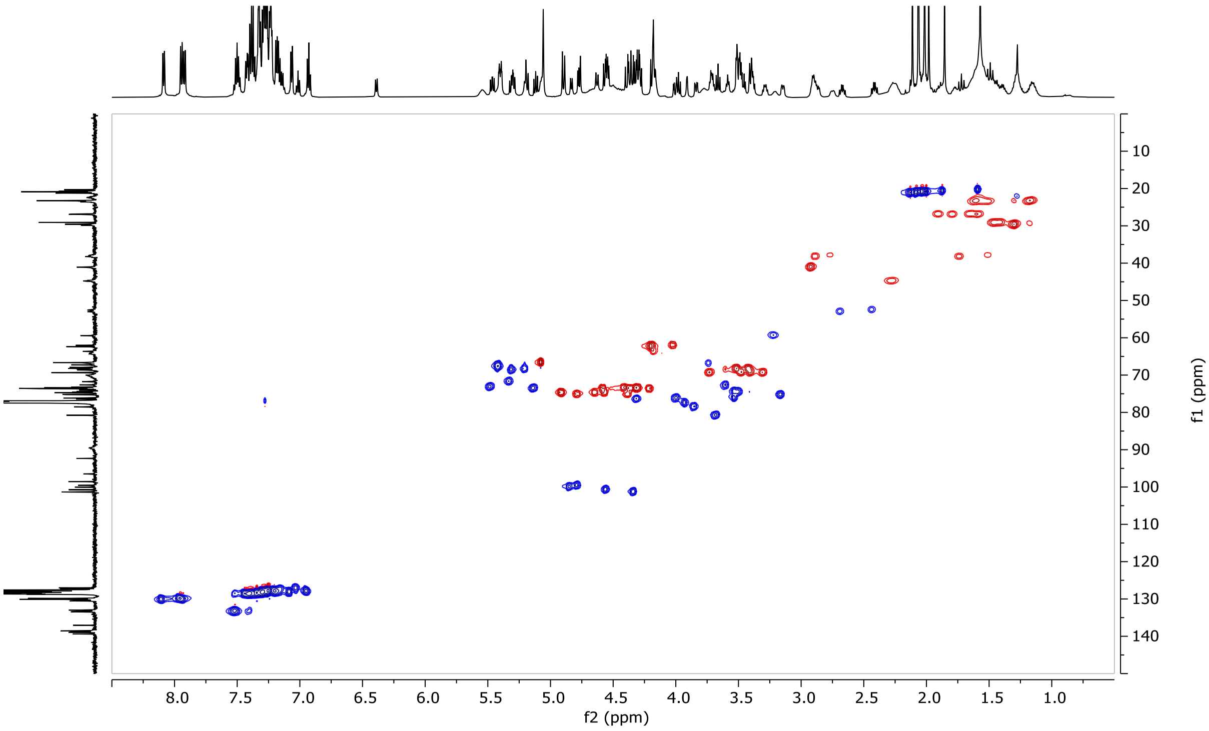

^{13}C-^{1}H HMBC

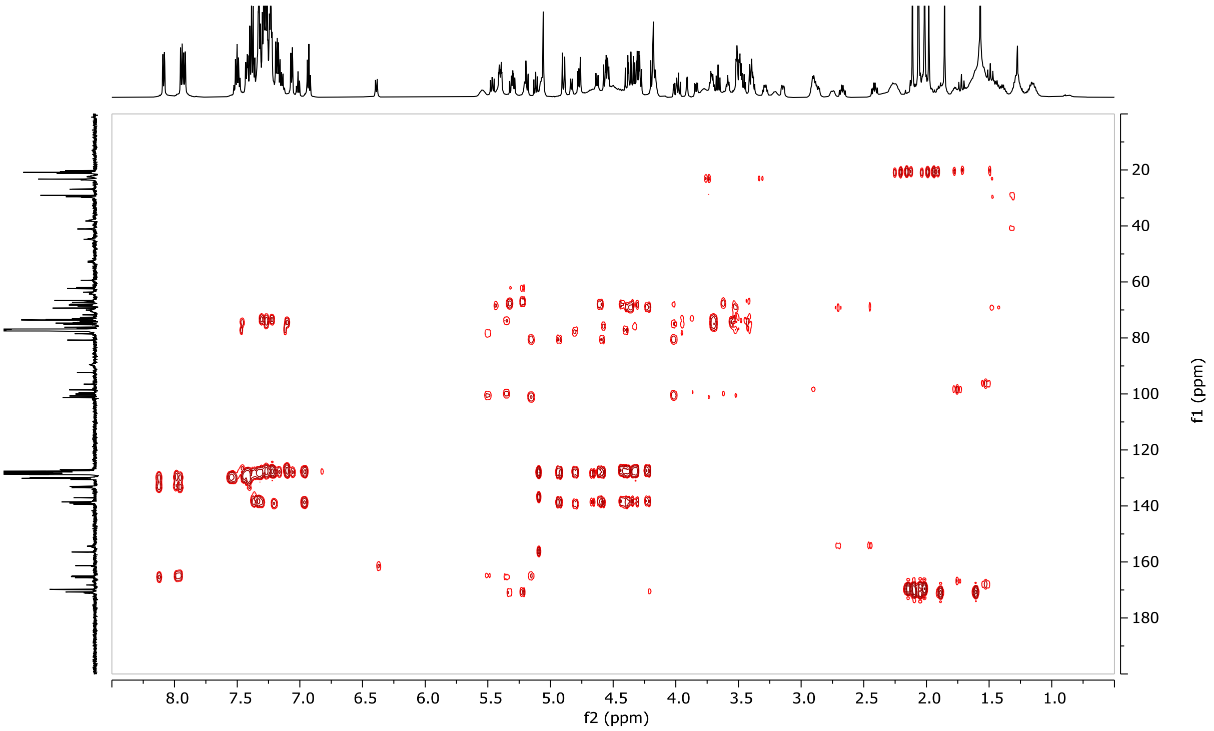

5.9 AGA of Hexasaccharide **15**

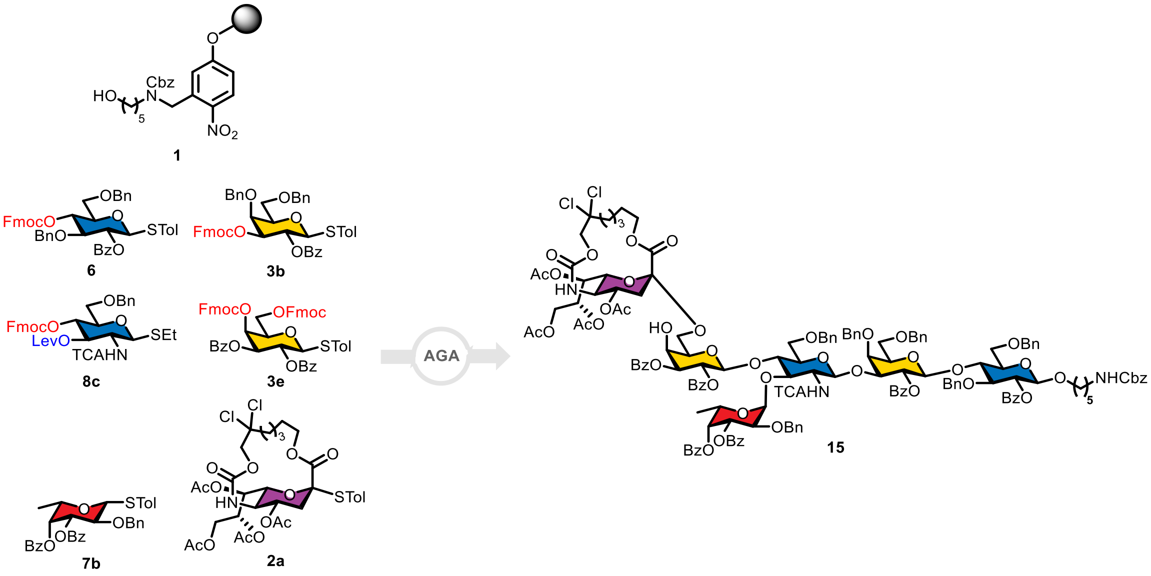

	Building blocks	Modules	Glycosylation condition
AGA		**Initiation** (40 mg resin)	
	Glc 6	Acidic wash<br>Thioglycoside glycosylation<br>Capping<br>Fmoc deprotection	8 eq. — t (min)<br>T_1 = -20 °C — 5<br>T_2 = 0 °C — 20
	Gal 3b	Acidic wash<br>Thioglycoside glycosylation<br>Capping<br>Fmoc deprotection	8 eq. — t (min)<br>T_1 = -40 °C — 5<br>T_2 = -20 °C — 20
	GlcN 8c	Acidic wash<br>Thioglycoside glycosylation<br>Capping<br>Lev deprotection	8 eq. — t (min)<br>T_1 = -20 °C — 5<br>T_2 = 0 °C — 40
	Fuc 7b	Acidic wash<br>Thioglycoside glycosylation<br>Capping<br>Fmoc deprotection	10 eq. — t (min)<br>T_1 = -40 °C — 5<br>T_2 = -20 °C — 20
	Gal 3e	Acidic wash<br>Thioglycoside glycosylation<br>Capping<br>Fmoc deprotection	10 eq. — t (min)<br>T_1 = -20 °C — 5<br>T_2 = 0 °C — 20
	Sia 2a	Acidic wash<br>Thioglycoside glycosylation x2<br>Pyridine wash	10 eq. — t (min)<br>T_1 = -40 °C — 30<br>T_2 = -20 °C — 10
Post AGA		Photocleavage<br>NP-HPLC Purification	

Protected compound **15** (8.8 mg, 2.96 µmol, 22%) was obtained as a colorless oil by purification using preparative NP-HPLC (**Method N1**).

^{1}H NMR (600 MHz, $CDCl_3$, 50 °C): δ 8.05 (d, J = 7.7 Hz, 2H), 7.96 (dt, J = 22.6, 7.7 Hz, 8H), 7.76 (d, J = 7.8 Hz, 2H), 7.66 – 7.58 (m, 1H), 7.57 – 7.44 (m, 11H), 7.43 – 7.34 (m, 19H), 7.26 (s, 8H), 7.21 – 7.03 (m, 13H), 6.98 (t, J = 7.5 Hz, 2H), 6.59 (d, J = 8.3 Hz, 1H), 5.73 (dd, J = 10.3, 8.0 Hz, 1H), 5.68 (dd, J = 10.3, 3.5 Hz, 1H), 5.65 (d, J = 3.6 Hz, 1H), 5.58 (dd, J = 10.2, 7.7 Hz, 1H), 5.41 (d, J = 3.7 Hz, 1H), 5.32 – 5.24 (m, 3H), 5.17 (t, J = 8.5 Hz, 1H), 5.09 (s, 3H), 5.06 – 5.01 (m, 2H), 4.92 (t, J = 9.8 Hz, 2H), 4.83 (d, J = 7.1 Hz, 1H), 4.71 (d, J = 12.0 Hz, 1H), 4.65 (d, J = 12.1 Hz, 1H), 4.64 – 4.57 (m, 3H), 4.57 – 4.46 (m, 6H), 4.41 – 4.27 (m, 8H), 4.23 (d, J = 11.7 Hz, 1H), 4.17 – 4.05 (m, 5H), 4.03 (t, J = 8.9 Hz, 1H), 3.89 (dd, J = 9.4, 3.9 Hz, 1H), 3.86 – 3.78 (m, 3H), 3.78 – 3.71 (m, 2H), 3.70 – 3.63 (m, 2H), 3.56 (dd, J = 11.2, 4.2 Hz, 1H), 3.52 – 3.44 (m, 4H), 3.43 – 3.36 (m,

2H), 3.32 (dt, J = 9.7, 6.5 Hz, 1H), 3.23 (ddd, J = 9.6, 4.2, 2.2 Hz, 1H), 2.97 – 2.91 (m, 3H), 2.91 – 2.84 (m, 1H), 2.77 (dd, J = 13.0, 5.3 Hz, 1H), 2.33 – 2.23 (m, 3H), 2.04 (s, 3H), 2.00 – 1.94 (m, 4H), 1.85 – 1.78 (m, 2H), 1.76 (s, 3H), 1.68 – 1.62 (m, 3H), 1.52 – 1.38 (m, 2H), 1.34 (d, J = 6.5 Hz, 3H), 1.32 – 1.26 (m, 2H), 1.26 – 1.13 (m, 2H)

^{13}C NMR (151 MHz, $CDCl_3$, 50 °C): δ 171.4, 171.1, 170.0, 170.0, 170.0, 169.8, 169.8, 167.1, 166.2, 165.9, 165.4, 165.3, 165.2, 165.2, 165.0, 161.4, 156.5, 154.3, 139.3, 138.9, 138.6, 138.4, 138.0, 137.9, 137.1, 133.4, 133.2, 133.2, 133.2, 133.0, 130.5, 130.5, 130.2, 130.2, 130.1, 130.1, 130.0, 129.9, 129.8, 129.7, 129.0, 128.9, 128.8, 128.7, 128.6, 128.5, 128.5, 128.4, 128.4, 128.4, 128.3, 128.2, 128.1, 128.1, 128.0, 128.0, 127.9, 127.8, 127.7, 127.7, 127.6, 127.5, 127.5, 127.5, 127.4, 127.3, 127.1, 101.3, 101.0, 100.6, 100.1, 99.2, 96.7, 92.3, 80.9, 78.9, 76.6, 76.4, 76.4, 75.7, 75.6, 75.4, 75.4, 75.2, 74.9, 74.7, 74.2, 74.0, 73.9, 73.7, 73.7, 73.6, 73.2, 72.7, 70.8, 70.5, 69.8, 69.3, 69.3, 68.7, 68.3, 66.7, 65.8, 65.4, 62.6, 61.4, 59.2, 52.6, 45.0, 41.1, 35.2, 29.9, 29.6, 29.6, 29.1, 27.0, 23.9, 23.3, 22.5, 21.0, 20.8, 20.4, 20.3, 16.2.

HRMS (QToF): Calcd for $C_{154}H_{162}Cl_5N_3NaO_{46}$ [M + Na]$^+$ 2986.8770; found 2986.8674.

Crude analytical NP-HPLC (t_R = 27.7 min)

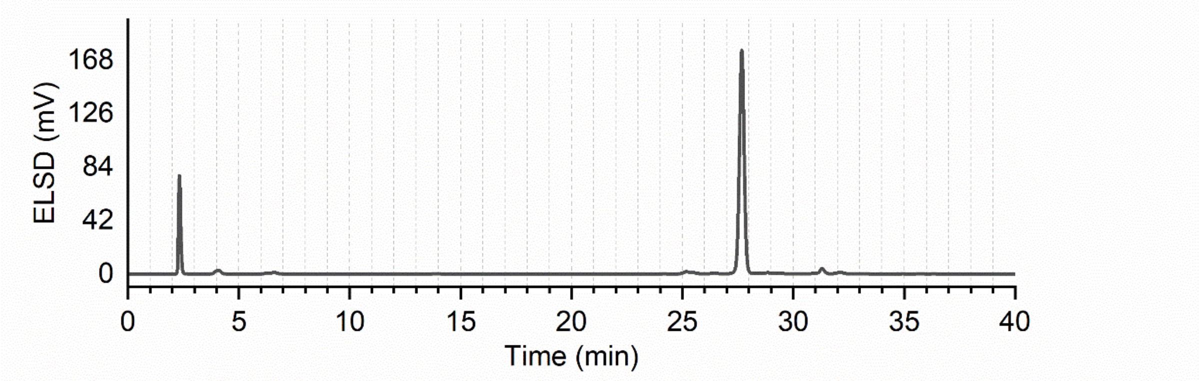

^{1}H-NMR (600 MHz, $CDCl_3$, 50 °C)

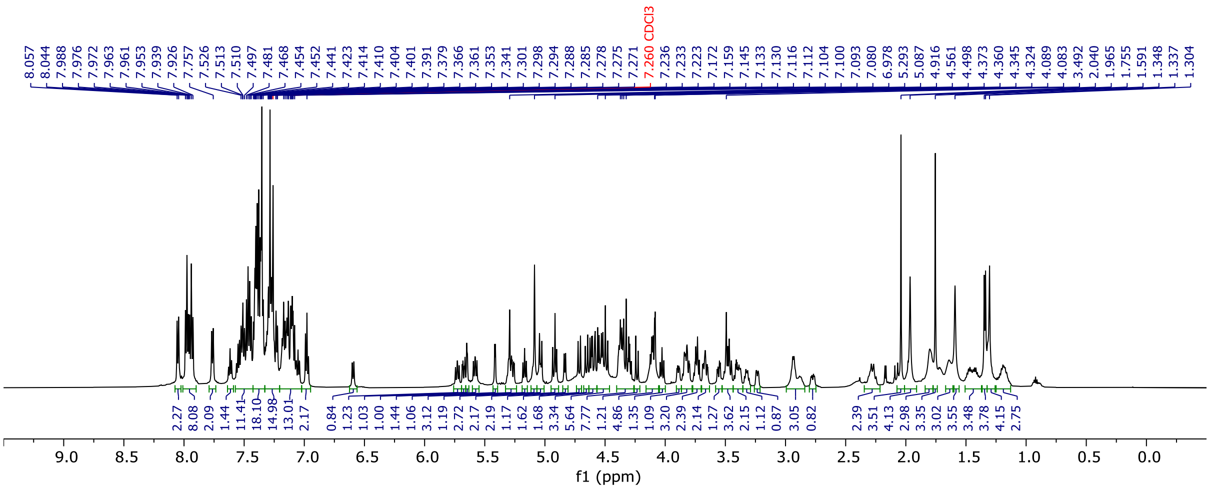

^{13}C-NMR (151 MHz, $CDCl_3$, 50 °C)

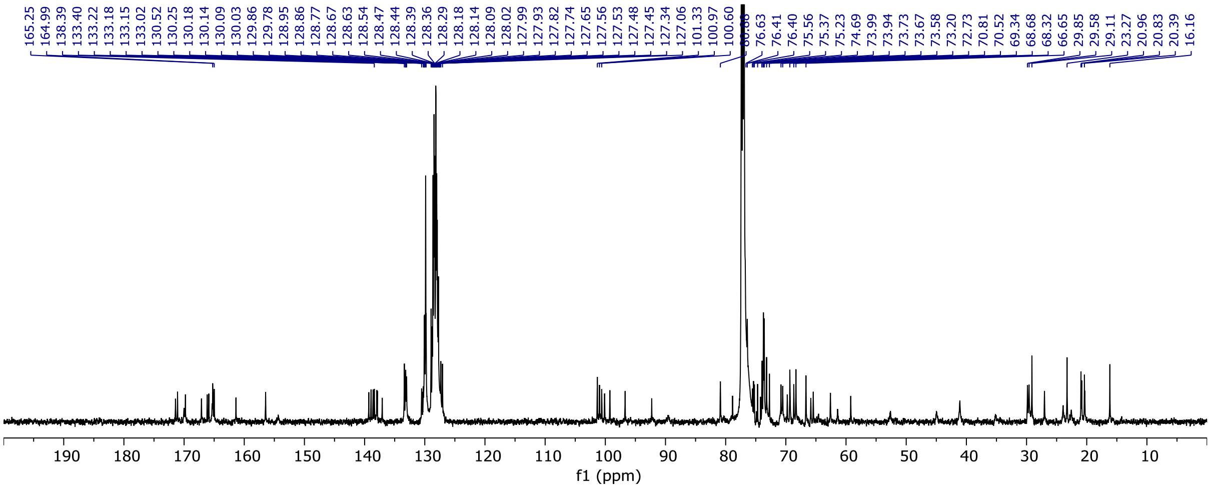

^{1}H -^{1}H COSY

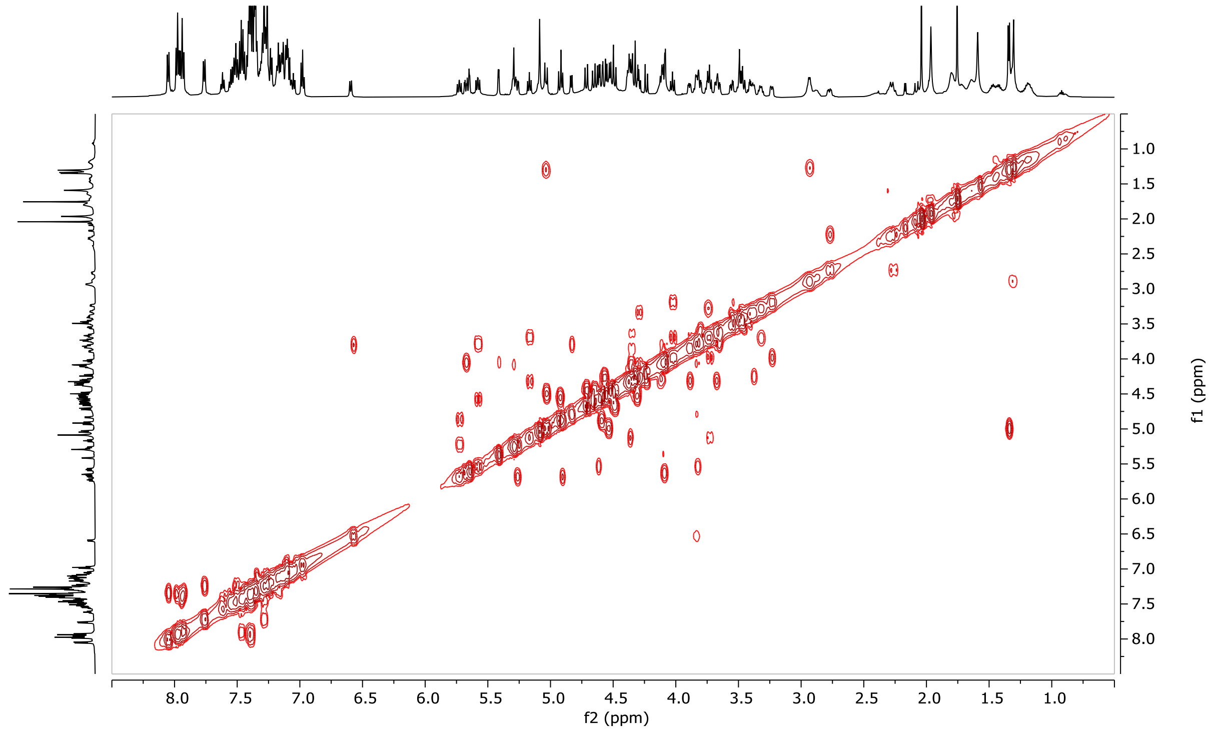

^{13}C-^{1}H HSQC

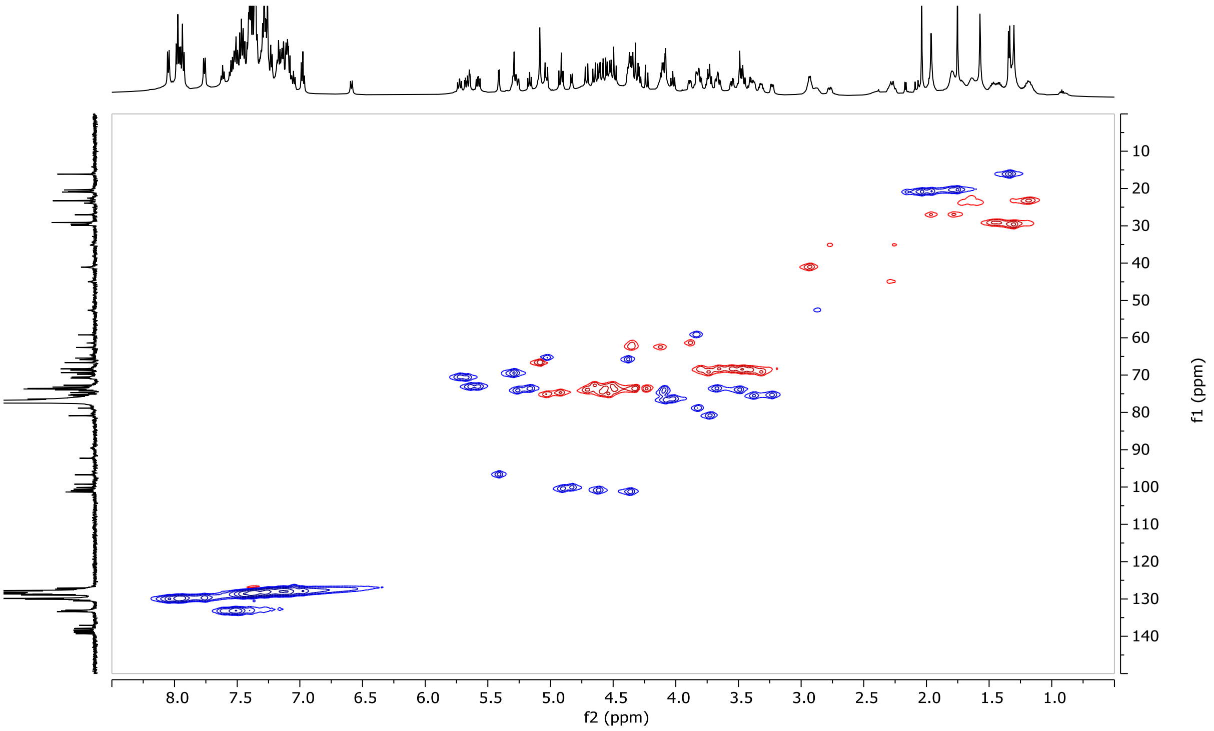

$^{13}C-^{1}H$ HMBC

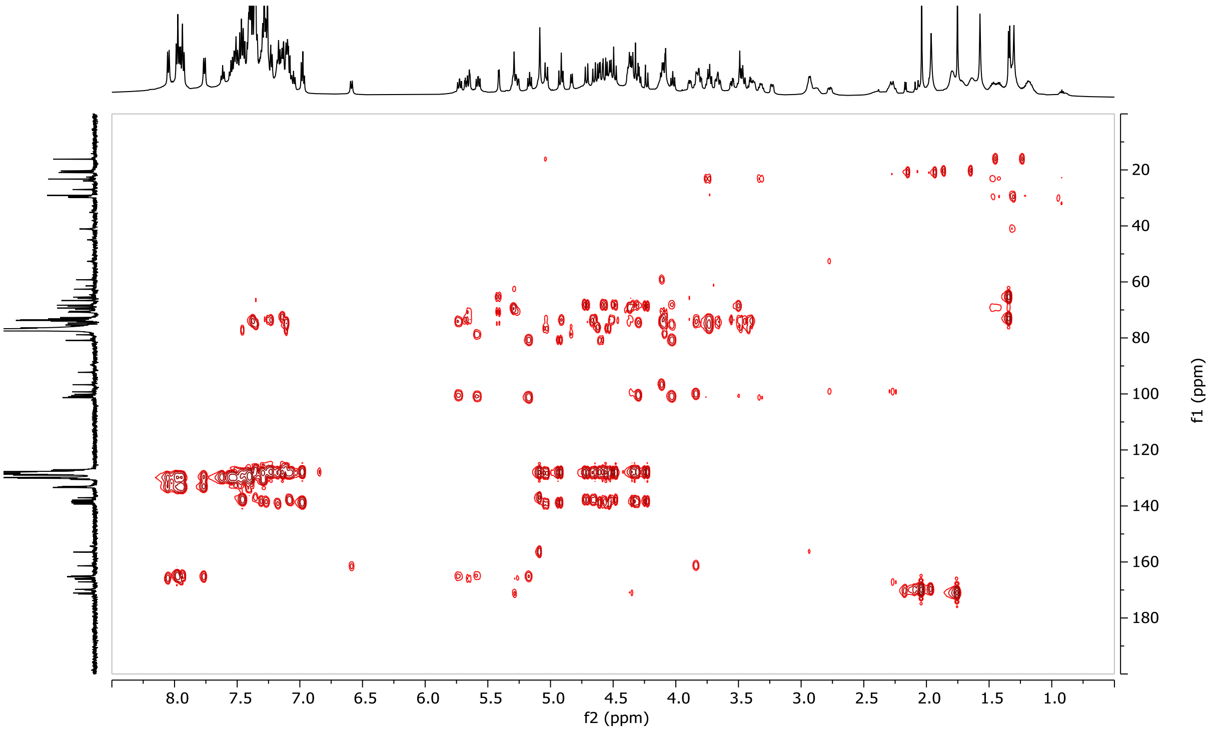

5.10 AGA of Hexasaccharide **16**

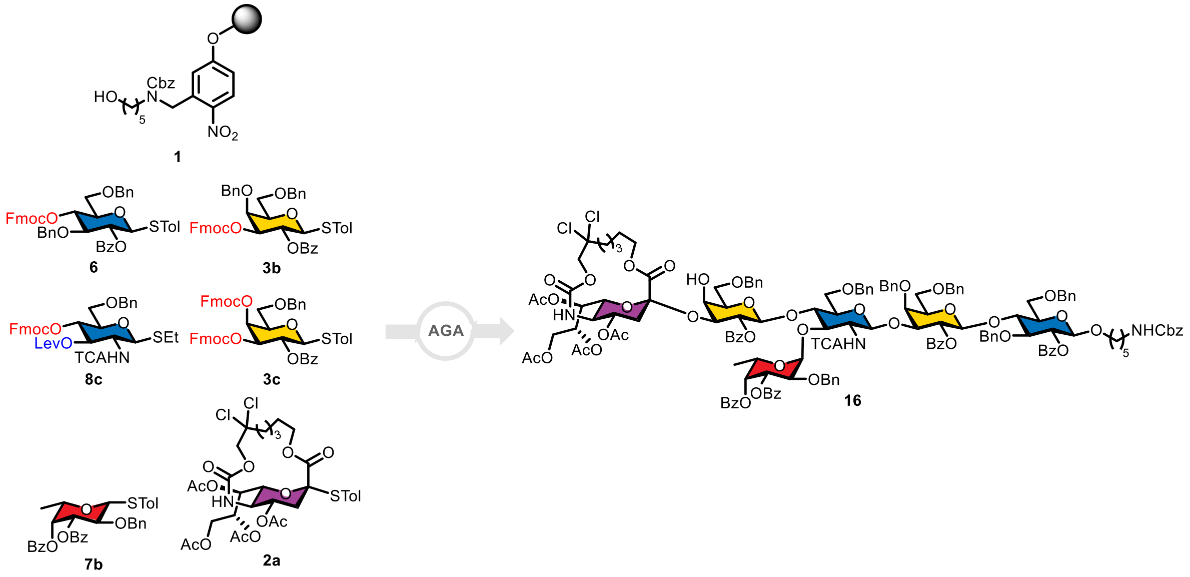

	Building blocks	Modules	Glycosylation condition
AGA		**Initiation** (40 mg resin)	
	**Glc 6**	**Acidic wash**<br>**Thioglycoside glycosylation**<br>**Capping**<br>**Fmoc deprotection**	8 eq. | t (min)<br>T_1 = -20 °C | 5<br>T_2 = 0 °C | 20
	**Gal 3b**	**Acidic wash**<br>**Thioglycoside glycosylation**<br>**Capping**<br>**Fmoc deprotection**	8 eq. | t (min)<br>T_1 = -40 °C | 5<br>T_2 = -20 °C | 20
	**GlcN 8c**	**Acidic wash**<br>**Thioglycoside glycosylation**<br>**Capping**<br>**Lev deprotection**	8 eq. | t (min)<br>T_1 = -20 °C | 5<br>T_2 = 0 °C | 40
	**Fuc 7b**	**Acidic wash**<br>**Thioglycoside glycosylation**<br>**Capping**<br>**Fmoc deprotection**	10 eq. | t (min)<br>T_1 = -40 °C | 5<br>T_2 = -20 °C | 20
	**Gal 3c**	**Acidic wash**<br>**Thioglycoside glycosylation**<br>**Capping**<br>**Fmoc deprotection**	10 eq. | t (min)<br>T_1 = -20 °C | 5<br>T_2 = 0 °C | 20
	**Sia 2a**	**Acidic wash**<br>**Thioglycoside glycosylation** **x4**<br>**Pyridine wash**	10 eq. | t (min)<br>T_1 = -40 °C | 30<br>T_2 = -20 °C | 10
**Post AGA**		**Photocleavage**<br>**NP-HPLC Purification**	

Protected compound **16** (7.9 mg, 2.65 μmol, 20%) was obtained as a colorless oil by purification using preparative NP-HPLC (**Method N1**).

^{1}H NMR (600 MHz, $CDCl_3$, 50 °C): δ 8.24 – 8.20 (m, 2H), 7.95 (dt, *J* = 8.3, 1.3 Hz, 4H), 7.91 (dd, *J* = 8.1, 1.4 Hz, 2H), 7.73 – 7.65 (m, 2H), 7.57 (tt, *J* = 7.4, 1.3 Hz, 1H), 7.54 – 7.45 (m, 6H), 7.45 – 7.36 (m, 11H), 7.35 – 7.30 (m, 10H), 7.30 – 7.17 (m, 11H), 7.17 – 7.12 (m, 1H), 7.12 – 7.00 (m, 13H), 6.98 – 6.92 (m, 2H), 6.57 (d, *J* = 8.4 Hz, 1H), 5.65 – 5.57 (m, 3H), 5.52 (dd, *J* = 10.2, 7.8 Hz, 1H), 5.49 – 5.42 (m, 1H), 5.36 (d, *J* = 3.7 Hz, 1H), 5.31 (dd, *J* = 9.7, 7.9 Hz, 1H), 5.22 (dd, *J* = 9.5, 1.8 Hz, 1H), 5.15 (dd, *J* = 9.1, 7.9 Hz, 1H), 5.10 – 5.04 (m, 3H), 4.94 (d, *J* = 11.9 Hz, 1H), 4.91 (d, *J* = 11.3 Hz, 1H), 4.87 (d, *J* = 7.9 Hz, 1H), 4.74 – 4.65 (m, 4H), 4.61 (d, *J* = 11.9 Hz, 1H), 4.59 – 4.49 (m, 8H), 4.46 (dd, *J* = 12.1, 2.4 Hz, 2H), 4.34 (d, *J* = 7.9 Hz, 1H), 4.32 – 4.25 (m, 3H), 4.23 (t, *J* = 6.8 Hz, 1H), 4.18 (d, *J* = 11.9 Hz, 1H), 4.09 (dd, *J* = 12.5, 4.8 Hz, 1H), 4.06 – 4.01 (m, 2H),

4.00 – 3.93 (m, 3H), 3.85 – 3.69 (m, 8H), 3.65 (t, J = 6.6 Hz, 1H), 3.58 (dd, J = 10.3, 2.9 Hz, 1H), 3.53 (dd, J = 11.2, 4.3 Hz, 1H), 3.50 – 3.34 (m, 6H), 3.33 – 3.27 (m, 1H), 3.22 (ddd, J = 9.6, 4.2, 2.1 Hz, 1H), 3.12 (q, J = 7.3 Hz, 1H), 2.91 (q, J = 6.9 Hz, 2H), 2.85 – 2.79 (m, 1H), 2.48 (td, J = 10.3, 6.4 Hz, 1H), 2.44 (br, 1H), 2.32 – 2.23 (m, 2H), 2.14 (s, 3H), 2.02 (s, 3H), 2.01 (s, 3H), 1.95 – 1.87 (m, 1H), 1.67 – 1.50 (m, 7H), 1.47 (s, 3H), 1.45 – 1.34 (m, 2H), 1.28 (d, J = 8.2 Hz, 2H), 1.16 (t, J = 6.1 Hz, 5H).

^{13}C NMR (151 MHz, $CDCl_3$, 50 °C): δ 170.8, 170.2, 169.8, 168.0, 166.2, 165.4, 165.3, 165.3, 164.9, 156.5, 154.5, 139.3, 139.0, 138.8, 138.6, 138.5, 138.4, 138.0, 137.1, 133.5, 133.3, 133.1, 133.1, 133.0, 132.8, 130.7, 130.5, 130.4, 130.3, 130.3, 130.2, 130.2, 130.1, 129.9, 129.9, 129.8, 128.9, 128.8, 128.6, 128.5, 128.5, 128.4, 128.3, 128.3, 128.2, 128.2, 128.1, 128.1, 128.0, 127.9, 127.9, 127.8, 127.8, 127.7, 127.7, 127.2, 127.1, 101.3, 101.0, 100.6, 100.3, 96.8, 96.7, 92.2, 80.9, 79.4, 77.4, 77.2, 77.0, 76.9, 76.7, 76.4, 76.2, 76.1, 75.4, 75.1, 75.0, 74.7, 74.1, 73.7, 73.6, 73.6, 73.5, 73.4, 73.4, 73.0, 72.9, 72.6, 71.7, 70.8, 69.3, 69.1, 68.9, 68.5, 68.5, 68.5, 68.4, 68.3, 68.3, 67.4, 66.8, 66.7, 66.7, 65.5, 62.5, 58.5, 52.5, 46.5, 44.7, 37.8, 32.1, 29.9, 29.8, 29.6, 29.5, 29.1, 26.8, 23.4, 23.3, 22.8, 21.3, 20.9, 20.8, 20.2, 16.2.

HRMS (QToF): Calcd for $C_{154}H_{164}Cl_5N_3NaO_{45}$ $[M + Na]^+$ 2972.8977; found 2972.9084.

Crude analytical NP-HPLC (t_R = 30.3 min)

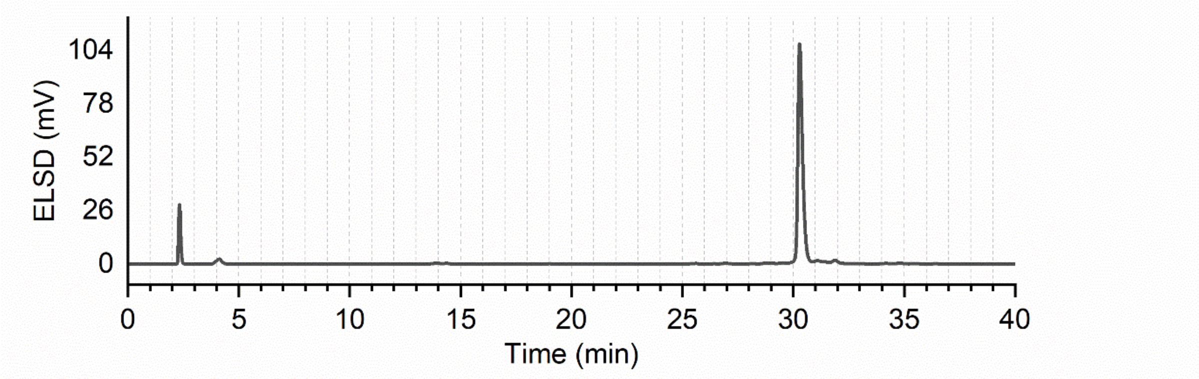

^{1}H-NMR (600 MHz, $CDCl_3$, 50 °C)

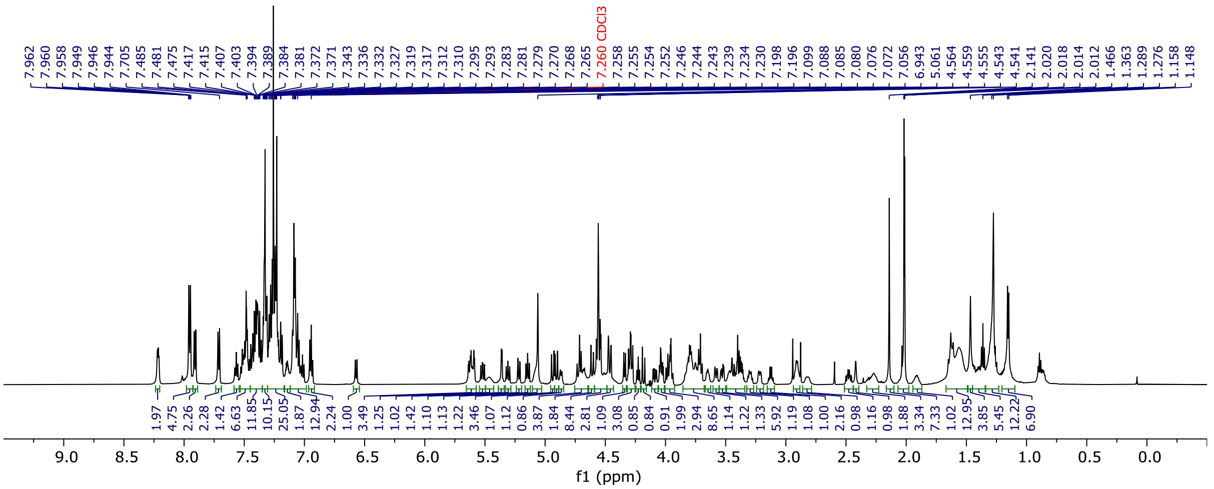

^{13}C-NMR (151 MHz, $CDCl_3$, 50 °C)

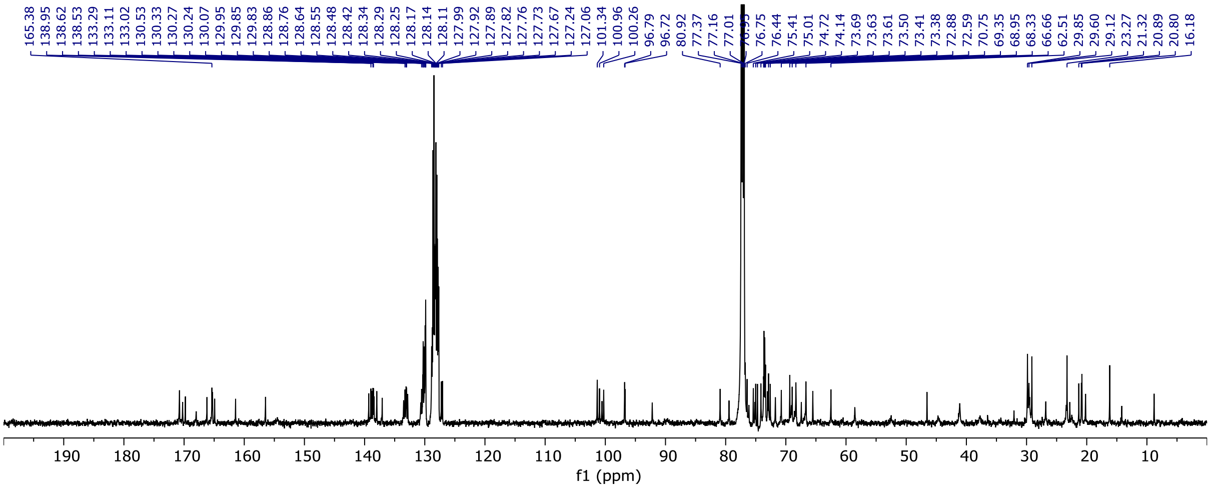

^{1}H -^{1}H COSY

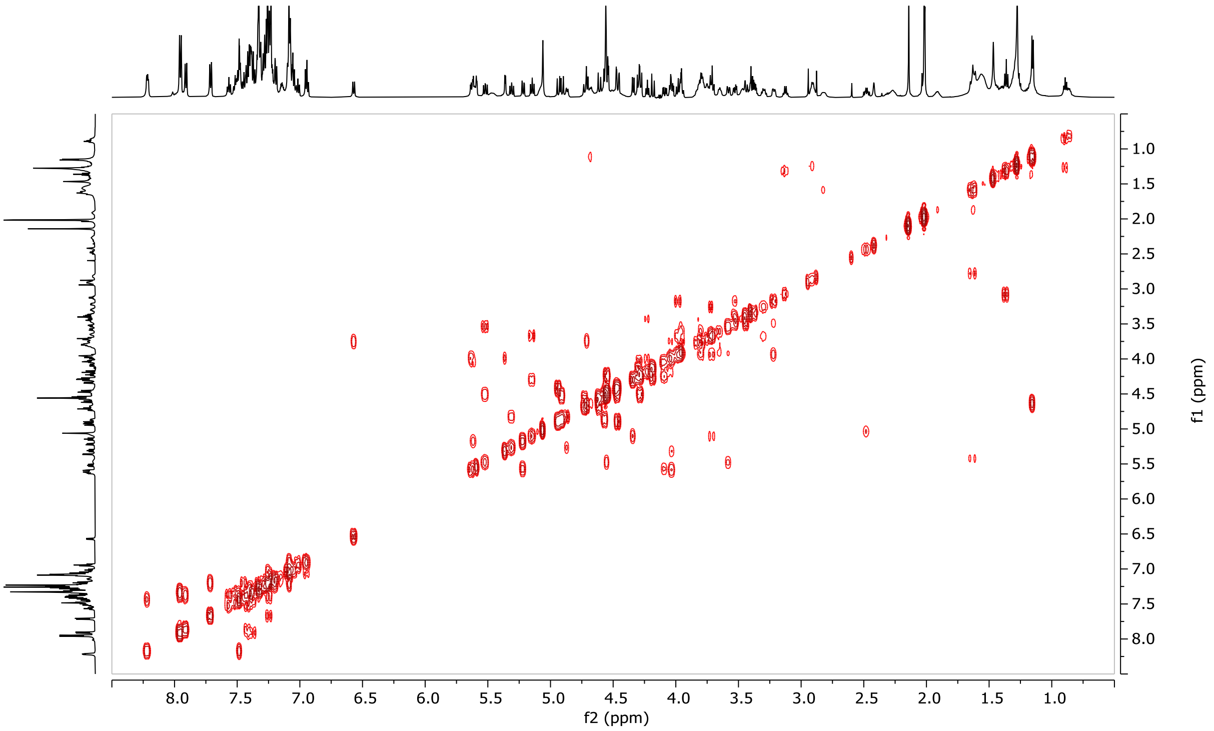

^{13}C-^{1}H HSQC

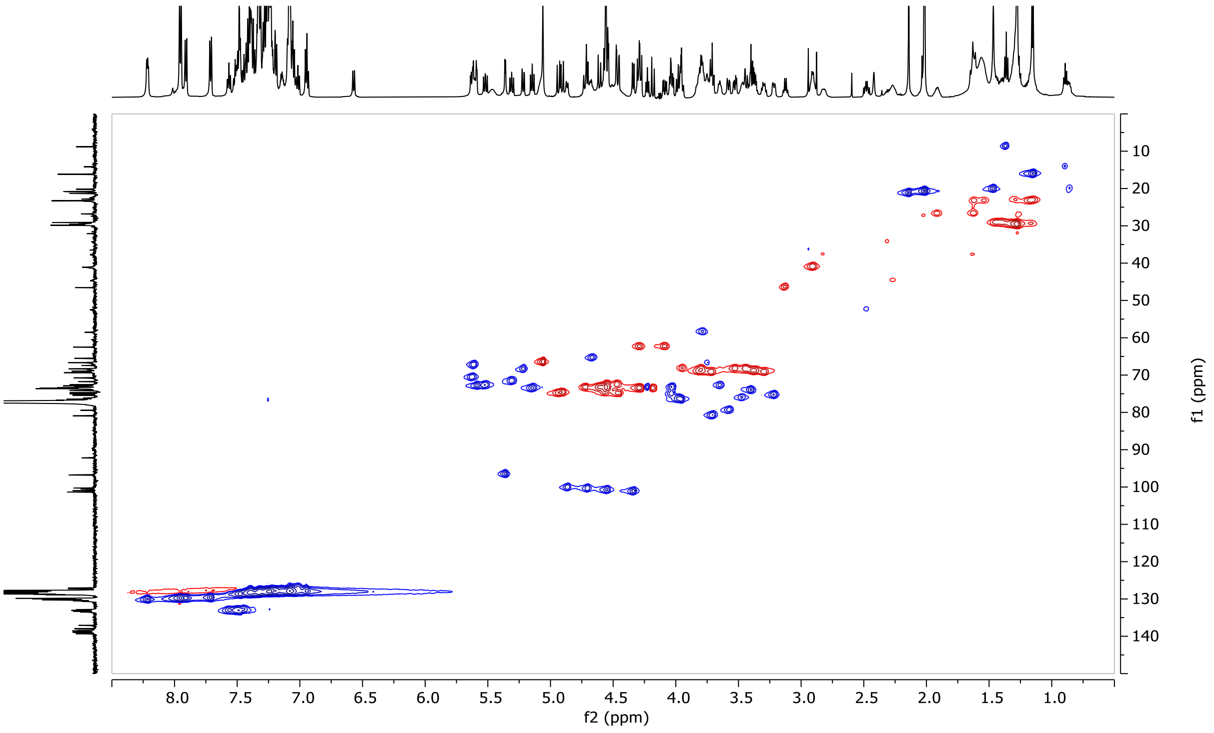

^{13}C-^{1}H HMBC

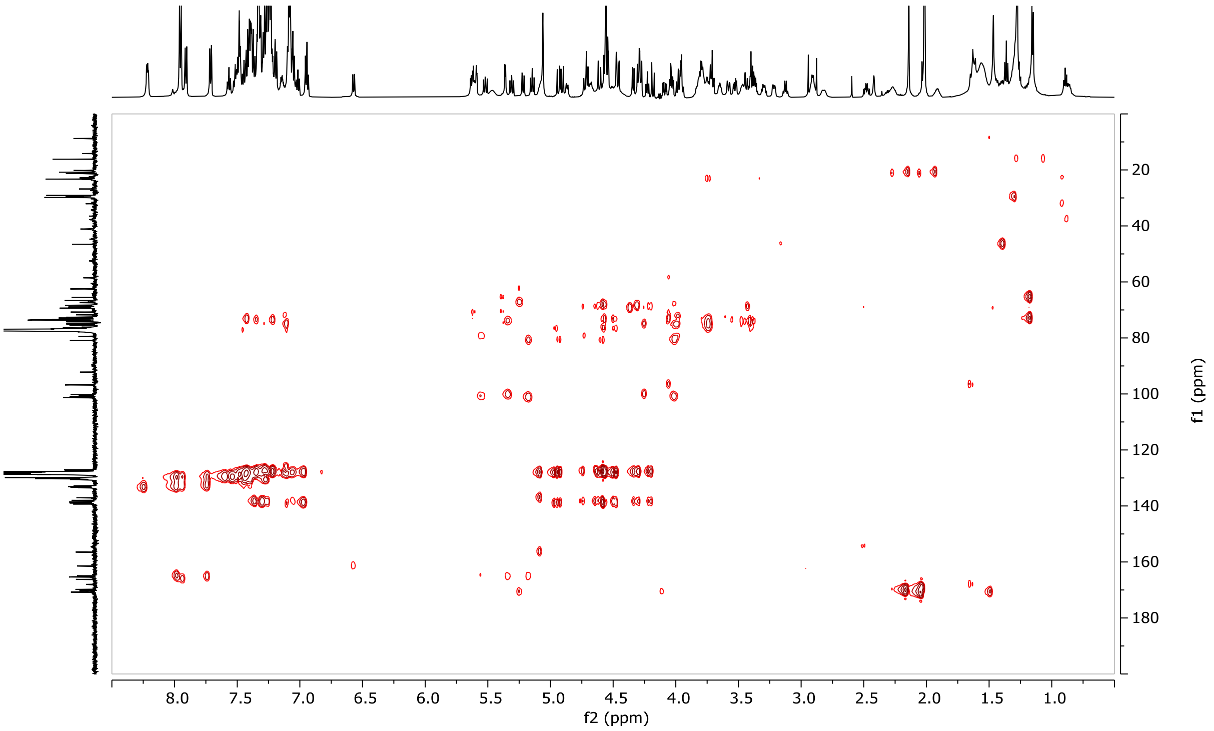

5.11 AGA of Heptasaccharide **17**

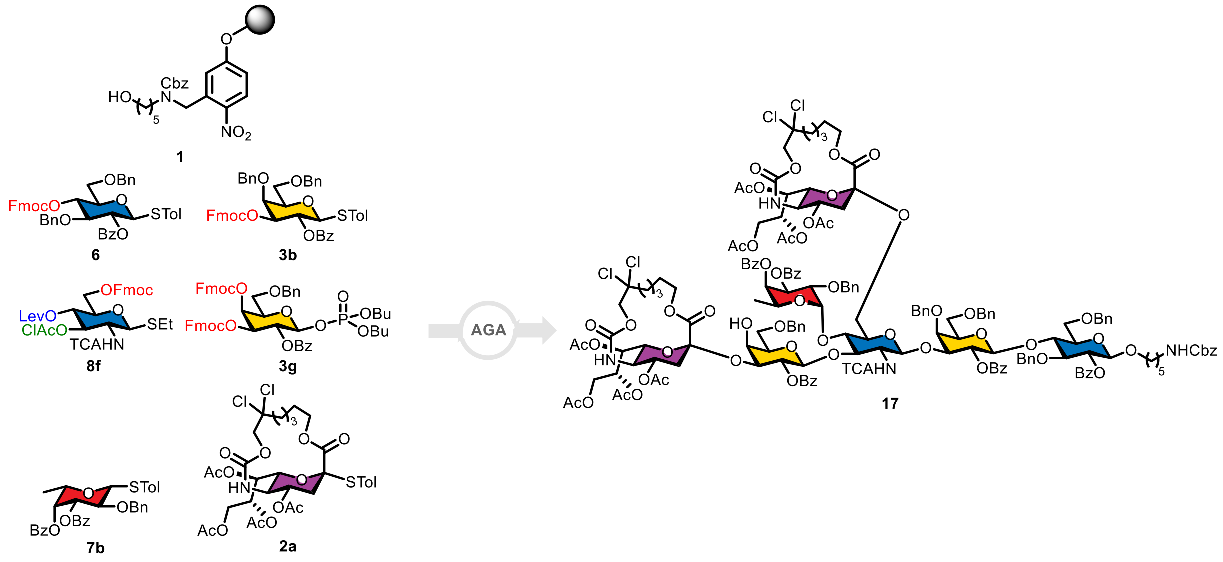

	Building blocks	Modules	Glycosylation condition
AGA		**Initiation** (40 mg resin)	
	**Glc 6**	**Acidic wash**<br>**Thioglycoside glycosylation**<br>**Capping**<br>**Fmoc deprotection**	8 eq. — t (min)<br>T_1 = -20 °C — 5<br>T_2 = 0 °C — 20
	**Gal 3b**	**Acidic wash**<br>**Thioglycoside glycosylation**<br>**Capping**<br>**Fmoc deprotection**	8 eq. — t (min)<br>T_1 = -40 °C — 5<br>T_2 = -20 °C — 20
	**GlcN 8f**	**Acidic wash**<br>**Thioglycoside glycosylation**<br>**Capping**<br>**ClAc deprotection**	8 eq. — t (min)<br>T_1 = -20 °C — 5<br>T_2 = 0 °C — 40
	**Gal 3g**	**Acidic wash**<br>**Phosphate glycosylation**<br>**Pyridine wash** (Acidic wash – Pyridine wash **x2**)<br>**Capping**<br>**Lev deprotection x2**	5 eq. — t (min)<br>T_1 = -40 °C — 10<br>T_2 = -20 °C — 30
	**Fuc 7b**	**Acidic wash**<br>**Thioglycoside glycosylation**<br>**Capping**<br>**Fmoc deprotection**	8 eq. — t (min)<br>T_1 = -40 °C — 5<br>T_2 = -20 °C — 20
	**Sia 2a**	**Acidic wash**<br>**Thioglycoside glycosylation**<br>**Pyridine wash** (Acidic wash – Pyridine wash **x6**)<br>**Capping**	10 eq. — t (min)<br>T_1 = -40 °C — 30<br>T_2 = -20 °C — 10
**Post AGA**		**Photocleavage**<br>**NP-HPLC Purification**	

Protected compound **17** (5.5 mg, 1.58 µmol, 12%) was obtained as a colorless oil by purification using preparative NP-HPLC (**Method N2**).

^{1}H NMR (700 MHz, $CDCl_3$, 50 °C): δ 8.08 – 8.05 (m, 2H), 8.01 – 7.98 (m, 2H), 7.96 (ddd, *J* = 9.6, 8.0, 1.4 Hz, 4H), 7.73 – 7.69 (m, 2H), 7.61 (tt, *J* = 7.3, 1.4 Hz, 1H), 7.52 – 7.40 (m, 9H), 7.40 – 7.31 (m, 12H), 7.31 – 7.25 (m, 4H), 7.24 – 7.18 (m, 13H), 7.18 – 7.12 (m, 6H), 7.10 – 7.06 (m, 2H), 7.04 – 7.01 (m, 1H), 6.95 (t, *J* = 7.6 Hz, 2H), 6.60 – 6.54 (m, 1H), 5.71 – 5.65 (m, 2H), 5.58 – 5.53 (m, 1H), 5.51 (dd, *J* = 10.1, 7.8 Hz, 1H), 5.44 (ddd, *J* = 9.7, 3.7, 2.3 Hz, 1H), 5.41 – 5.36 (m, 2H), 5.35 – 5.31 (m, 2H), 5.28 (dd, *J* = 9.7, 7.9 Hz, 1H), 5.24 (d, *J* = 6.6 Hz, 1H), 5.18 (dd, *J* = 9.7, 1.6 Hz, 1H), 5.13 (dd, *J* = 9.2, 7.9 Hz, 1H), 5.06 (s, 3H), 5.03 – 4.99 (m, 1H), 4.95 (d, *J* = 8.0 Hz, 1H), 4.91 (d, *J* = 11.4 Hz, 1H), 4.80 (d, *J* = 11.6 Hz, 1H), 4.77 – 4.69 (m, 3H), 4.69 – 4.55 (m, 8H), 4.54 – 4.37 (m, 5H),

4.36 – 4.28 (m, 6H), 4.26 (t, *J* = 8.4 Hz, 1H), 4.23 – 4.11 (m, 8H), 4.00 – 3.92 (m, 3H), 3.90 (dd, *J* = 9.8, 5.8 Hz, 1H), 3.88 – 3.82 (m, 2H), 3.80 – 3.65 (m, 5H), 3.65 – 3.61 (m, 1H), 3.57 – 3.49 (m, 3H), 3.48 – 3.41 (m, 3H), 3.37 (dd, *J* = 9.8, 5.8 Hz, 1H), 3.32 – 3.27 (m, 1H), 3.21 (ddd, *J* = 9.5, 4.5, 2.2 Hz, 1H), 2.96 (dd, *J* = 12.9, 5.5 Hz, 1H), 2.93 – 2.87 (m, 2H), 2.78 (td, *J* = 10.5, 6.6 Hz, 1H), 2.73 – 2.67 (m, 1H), 2.42 – 2.35 (m, 2H), 2.34 – 2.18 (m, 3H), 2.08 (s, 3H), 2.07 (s, 6H), 2.00 (s, 3H), 1.98 (s, 3H), 1.96 (s, 6H), 1.92 – 1.86 (m, 2H), 1.83 (t, *J* = 12.3 Hz, 1H), 1.75 – 1.66 (m, 2H), 1.64 – 1.49 (m, 7H), 1.48 – 1.36 (m, 5H), 1.34 – 1.22 (m, 7H), 1.22 – 1.10 (m, 2H).

^{13}C NMR (176 MHz, $CDCl_3$, 25 °C): δ 171.2, 170.9, 170.8, 170.0, 169.9, 168.5, 166.8, 166.3, 165.4, 165.2, 165.1, 164.9, 161.3, 156.3, 154.0, 139.1, 138.8, 138.6, 138.4, 137.4, 136.8, 133.7, 133.2, 133.1, 132.9, 130.2, 130.2, 130.0, 129.9, 129.8, 129.8, 129.7, 129.1, 128.6, 128.5, 128.5, 128.4, 128.3, 128.2, 128.2, 128.1, 128.0, 127.9, 127.8, 127.6, 127.4, 127.0, 101.1, 100.8, 99.6, 98.5, 96.9, 95.6, 92.5, 88.9, 80.7, 76.5, 75.0, 74.8, 74.0, 73.9, 73.6, 73.4, 73.3, 73.1, 72.3, 71.2, 70.6, 70.0, 69.5, 68.6, 68.1, 67.7, 66.8, 66.6, 66.2, 66.0, 65.5, 63.7, 63.1, 62.2, 61.5, 53.0, 52.4, 45.0, 44.7, 41.6, 40.9, 38.1, 29.8, 29.4, 28.9, 26.4, 23.2, 22.2, 22.1, 21.7, 21.3, 21.0, 20.9, 20.9, 20.1, 16.3.

HRMS (QToF): Calcd for $C_{172}H_{191}Cl_7N_4Na_2O_{58}$ [M + 2Na]$^{2+}$ 1765.4867; found 1765.4810.

Crude analytical NP-HPLC (t_R = 27.6 min)

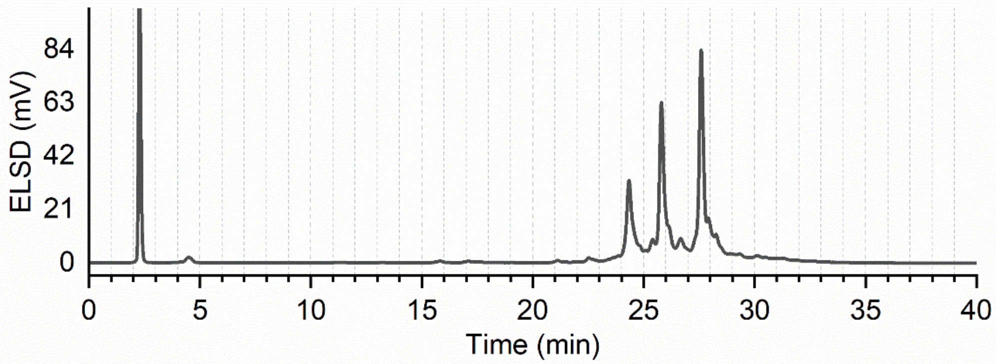

^{1}H-NMR (700 MHz, $CDCl_3$, 50 °C)

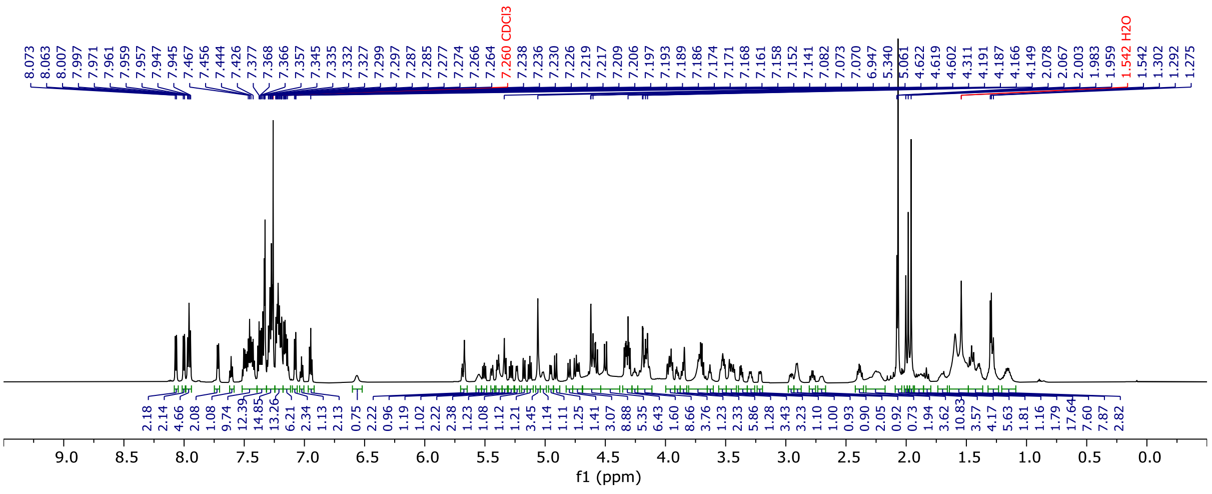

^{13}C-NMR (176 MHz, $CDCl_3$, 25 °C)

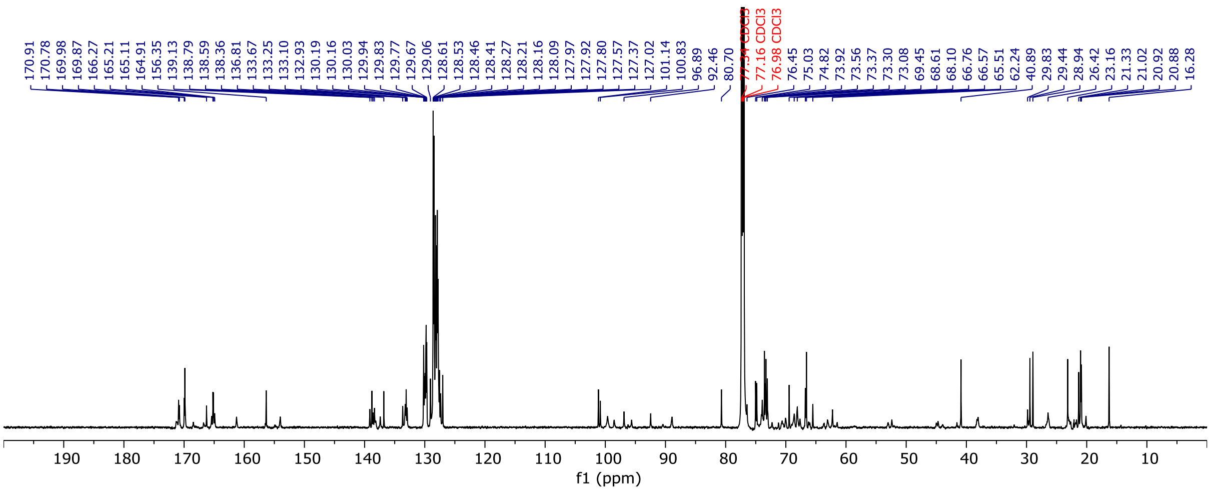

^1H -^1H COSY (25 °C)

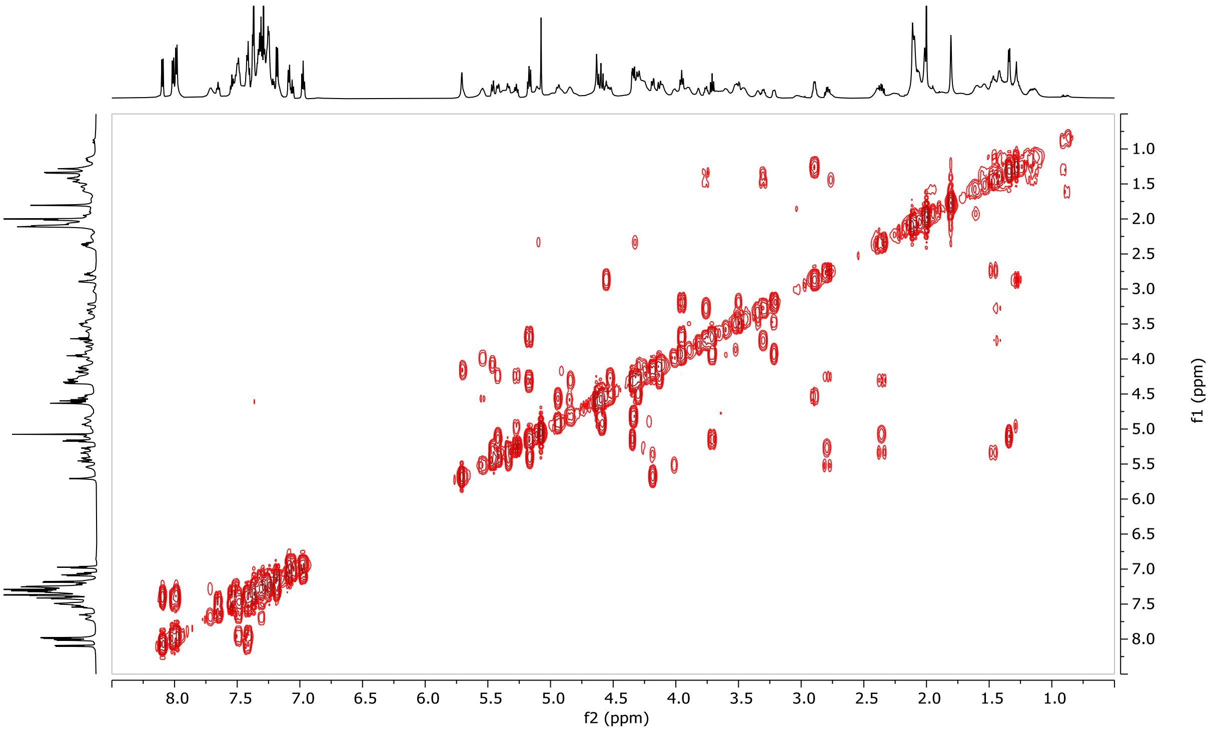

^{13}C-^1H HSQC (25 °C)

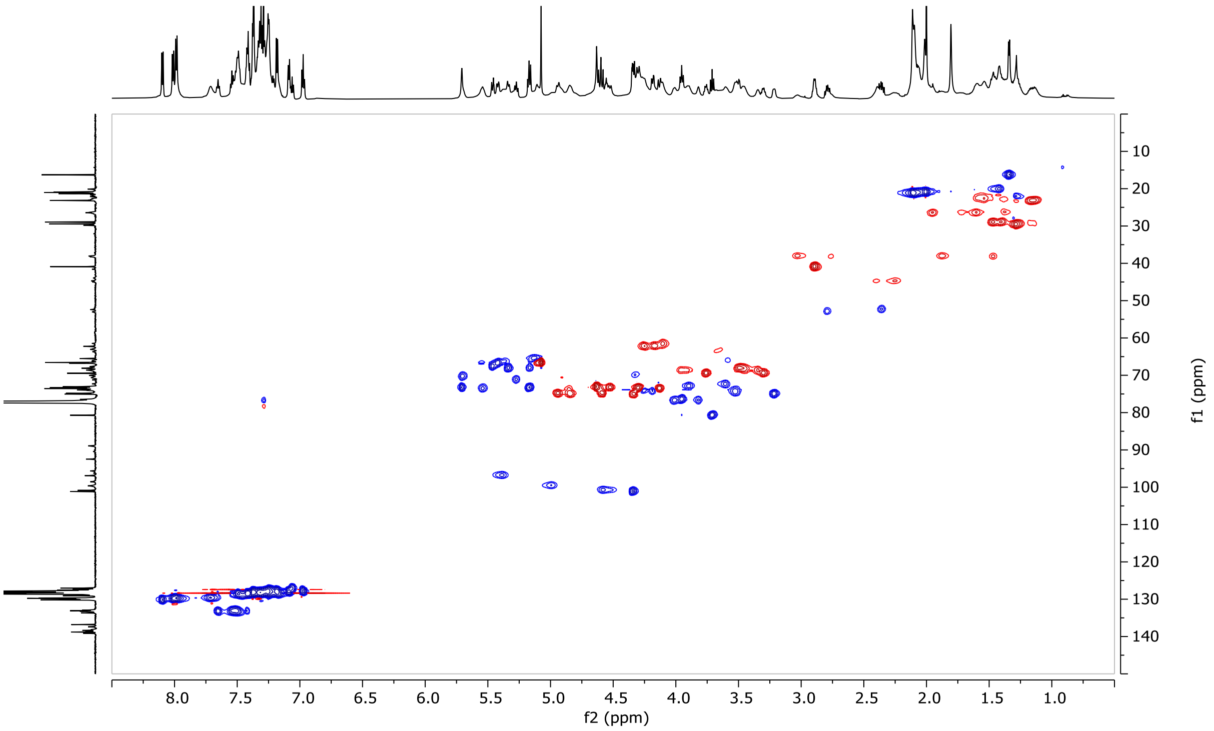

^{13}C-^{1}H HMBC (25 °C)

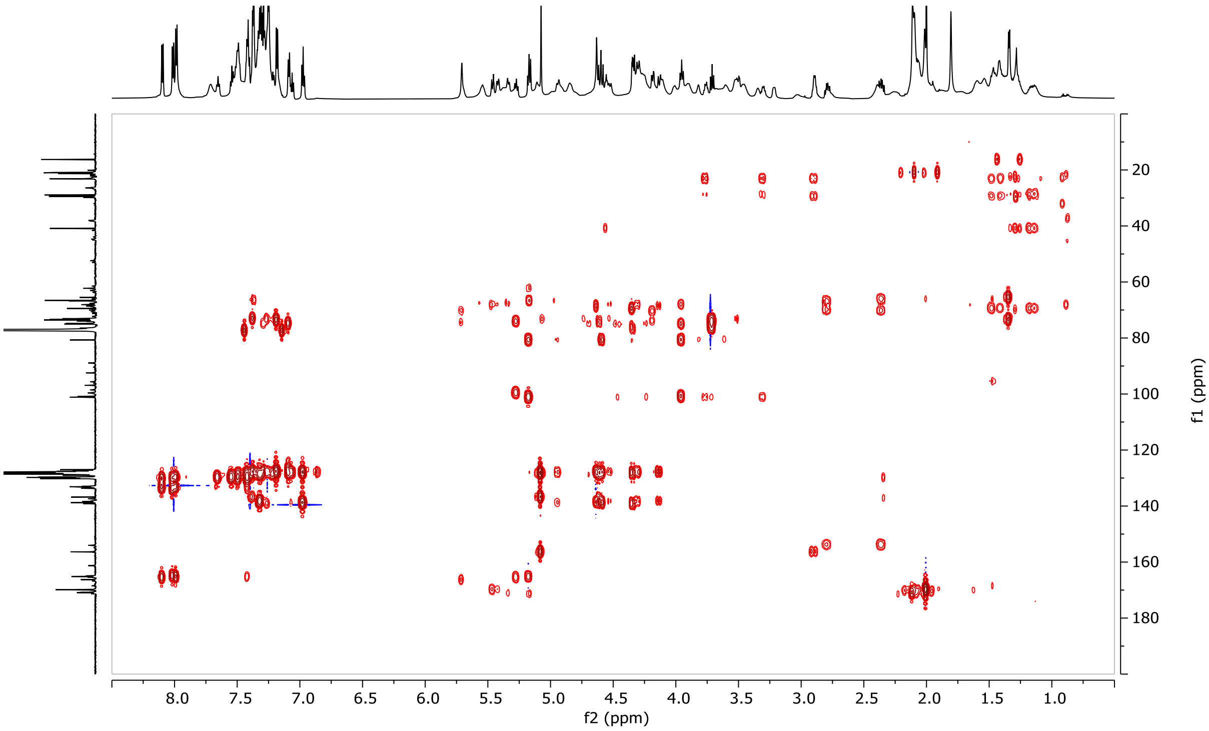

5.12 AGA of Hexasaccharide **18**

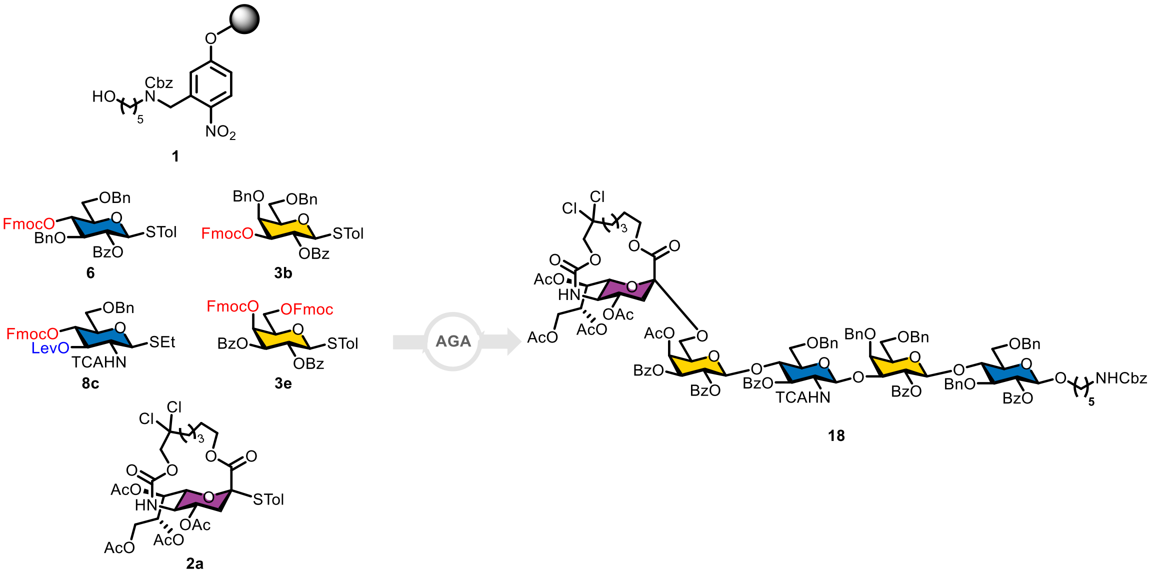

	Building blocks	Modules	Glycosylation condition
AGA		**Initiation** (40 mg resin)	
	**Glc 6**	**Acidic wash**<br>**Thioglycoside glycosylation**<br>**Capping**<br>**Fmoc deprotection**	8 eq. / t (min)<br>T_1 = -20 °C / 5<br>T_2 = 0 °C / 20
	**Gal 3b**	**Acidic wash**<br>**Thioglycoside glycosylation**<br>**Capping**<br>**Fmoc deprotection**	8 eq. / t (min)<br>T_1 = -40 °C / 5<br>T_2 = -20 °C / 20
	**GlcN 8c**	**Acidic wash**<br>**Thioglycoside glycosylation**<br>**Capping**<br>**Lev deprotection**<br>**Bz Capping**<br>**Fmoc deprotection**	8 eq. / t (min)<br>T_1 = -20 °C / 5<br>T_2 = 0 °C / 40
	**Gal 3e**	**Acidic wash**<br>**Thioglycoside glycosylation**<br>**Capping**<br>**Fmoc deprotection**	8 eq. / t (min)<br>T_1 = -20 °C / 5<br>T_2 = 0 °C / 20
	**Sia 2a**	**Acidic wash**<br>**Thioglycoside glycosylation**<br>**Pyridine wash**<br>(x2)	10 eq. / t (min)<br>T_1 = -40 °C / 30<br>T_2 = -20 °C / 10
**Post AGA**		**Photocleavage**<br>**Capping** (Ac_2O/pyridine = 1/1)<br>**NP-HPLC Purification**	

Protected compound **18** (5.4 mg, 2.02 µmol, 15%) was obtained as a colorless oil by purification using preparative NP-HPLC (**Method N1**).

^{1}H NMR (700 MHz, $CDCl_3$, 50 °C): δ 8.03 – 7.99 (m, 2H), 7.97 – 7.93 (m, 2H), 7.93 – 7.90 (m, 2H), 7.90 – 7.87 (m, 2H), 7.82 – 7.79 (m, 2H), 7.61 – 7.56 (m, 1H), 7.53 – 7.46 (m, 3H), 7.45 (t, *J* = 7.8 Hz, 3H), 7.39 – 7.28 (m, 18H), 7.28 – 7.14 (m, 5H), 7.08 – 7.05 (m, 2H), 7.03 – 6.99 (m, 1H), 6.94 (t, *J* = 7.5 Hz, 2H), 6.48 (d, *J* = 9.4 Hz, 1H), 5.55 – 5.46 (m, 3H), 5.46 – 5.40 (m, 2H), 5.37 (dd, *J* = 9.1, 1.3 Hz, 1H), 5.32 (dd, *J* = 10.4, 3.5 Hz, 1H), 5.26 (dd, *J* = 10.5, 8.7 Hz, 1H), 5.21 (d, *J* = 6.7 Hz, 1H), 5.13 (dd, *J* = 9.2, 7.9 Hz, 1H), 5.09 – 5.02 (m, 3H), 4.90 – 4.84 (m, 2H), 4.80 – 4.65 (m, 1H), 4.61 – 4.48 (m, 7H), 4.47 (d, *J* = 12.1 Hz, 2H), 4.41 – 4.30 (m, 6H), 4.28 (d, *J* = 12.2 Hz, 1H), 4.22 (d, *J* = 12.0 Hz, 1H), 4.20 – 4.13 (m, 2H), 4.03 (d, *J* = 2.9 Hz, 1H), 3.98 (t, *J* = 9.0 Hz, 1H), 3.94 – 3.87 (m, 1H), 3.85 (dd, *J* = 10.2, 2.9 Hz, 1H), 3.78 (dd, *J* = 8.6, 5.8 Hz, 1H), 3.72 (dt, *J* = 9.8, 6.0 Hz, 1H), 3.70 – 3.64 (m, 3H), 3.55 – 3.46 (m, 4H), 3.43 (dd, *J* = 11.0, 2.0 Hz, 1H), 3.38

(dd, *J* = 9.2, 5.2 Hz, 1H), 3.32 – 3.27 (m, 1H), 3.20 (ddd, *J* = 9.6, 4.1, 2.2 Hz, 1H), 3.02 (br, 1H), 2.91 (d, *J* = 8.0 Hz, 3H), 2.75 – 2.65 (m, 2H), 2.36 – 2.24 (m, 2H), 2.17 (s, 3H), 2.11 (s, 3H), 2.03 (s, 3H), 2.03 (s, 3H), 1.95 – 1.89 (m, 4H), 1.78 – 1.70 (m, 1H), 1.67 – 1.54 (m, 3H), 1.50 (s, 12H), 1.33 – 1.23 (m, 4H), 1.21 – 1.10 (m, 2H).

^{13}C NMR (176 MHz, $CDCl_3$, 50 °C): δ 171.5, 170.7, 170.0, 169.9, 169.7, 166.7, 166.3, 165.5, 165.3, 165.2, 164.7, 162.1, 156.5, 139.2, 139.0, 138.6, 138.5, 138.2, 137.1, 133.6, 133.5, 133.4, 133.2, 133.0, 130.5, 130.3, 130.0, 129.9, 129.8, 129.6, 129.4, 128.9, 128.7, 128.6, 128.5, 128.5, 128.4, 128.3, 128.1, 128.1, 128.0, 127.9, 127.8, 127.7, 127.5, 127.0, 101.7, 101.4, 100.9, 100.6, 99.0, 92.1, 81.0, 79.5, 76.5, 75.4, 75.3, 75.2, 74.8, 74.1, 73.7, 73.7, 73.6, 73.1, 72.8, 72.1, 72.0, 70.5, 69.4, 68.8, 68.7, 68.5, 68.4, 68.2, 67.3, 66.7, 62.4, 56.5, 53.0, 44.9, 41.1, 38.1, 29.9, 29.6, 29.1, 27.1, 23.6, 23.3, 21.2, 20.9, 20.9, 20.5.

HRMS (QToF): Calcd for $C_{136}H_{144}Cl_5N_3NaO_{42}$ $[M + Na]^+$ 2688.7565; found 2688.7659.

Crude analytical NP-HPLC (t_R = 28.9 min)

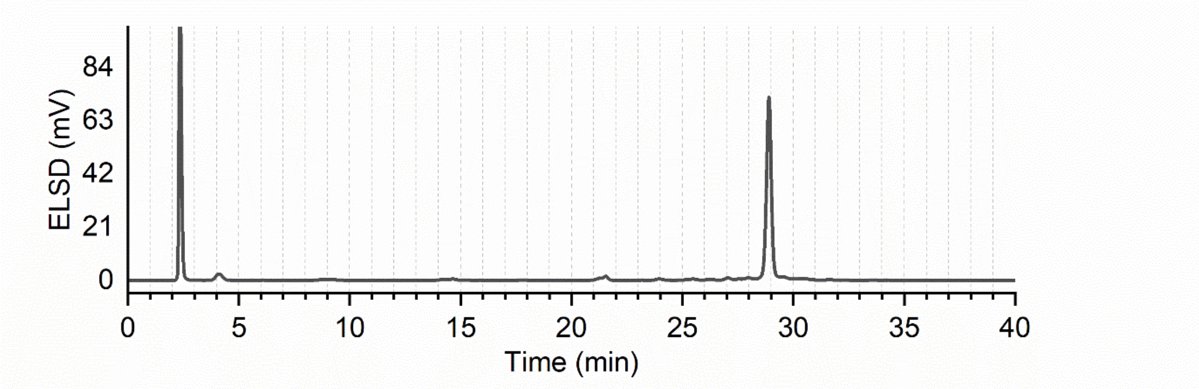

^{1}H-NMR (700 MHz, $CDCl_3$, 50 °C)

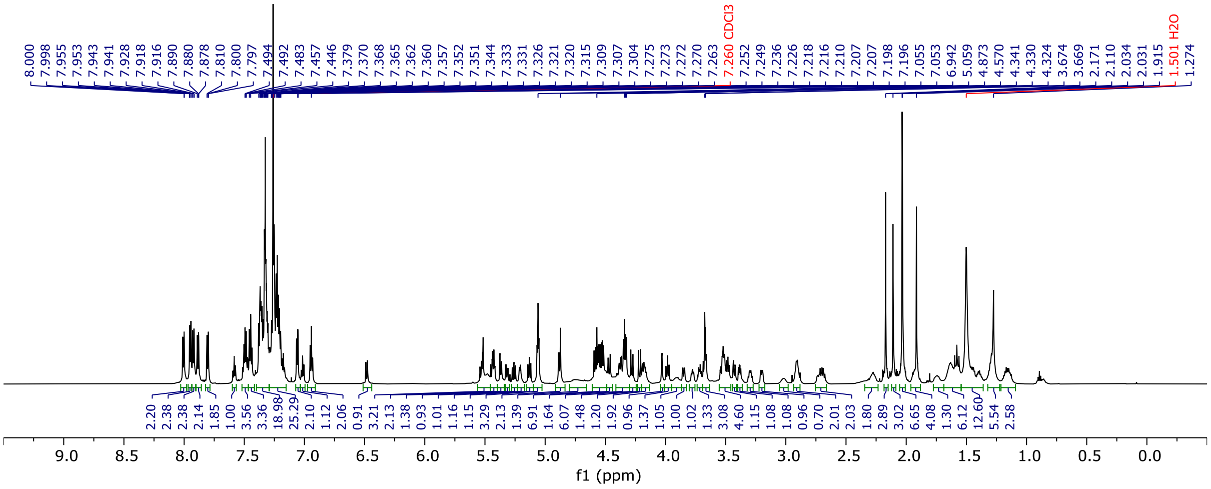

^{13}C-NMR (176 MHz, $CDCl_3$, 50 °C)

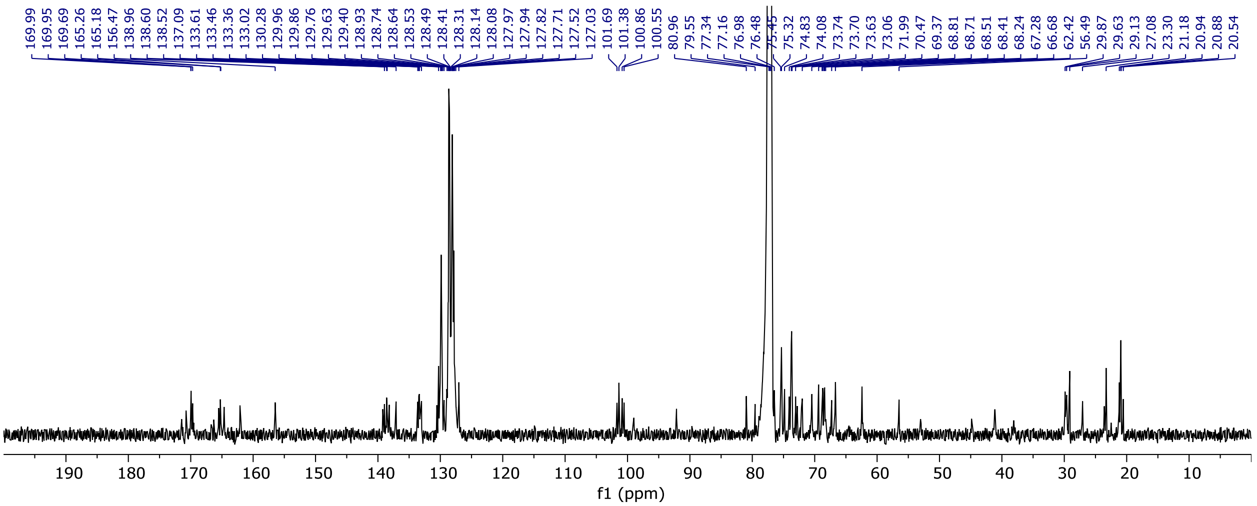

^{1}H -^{1}H COSY

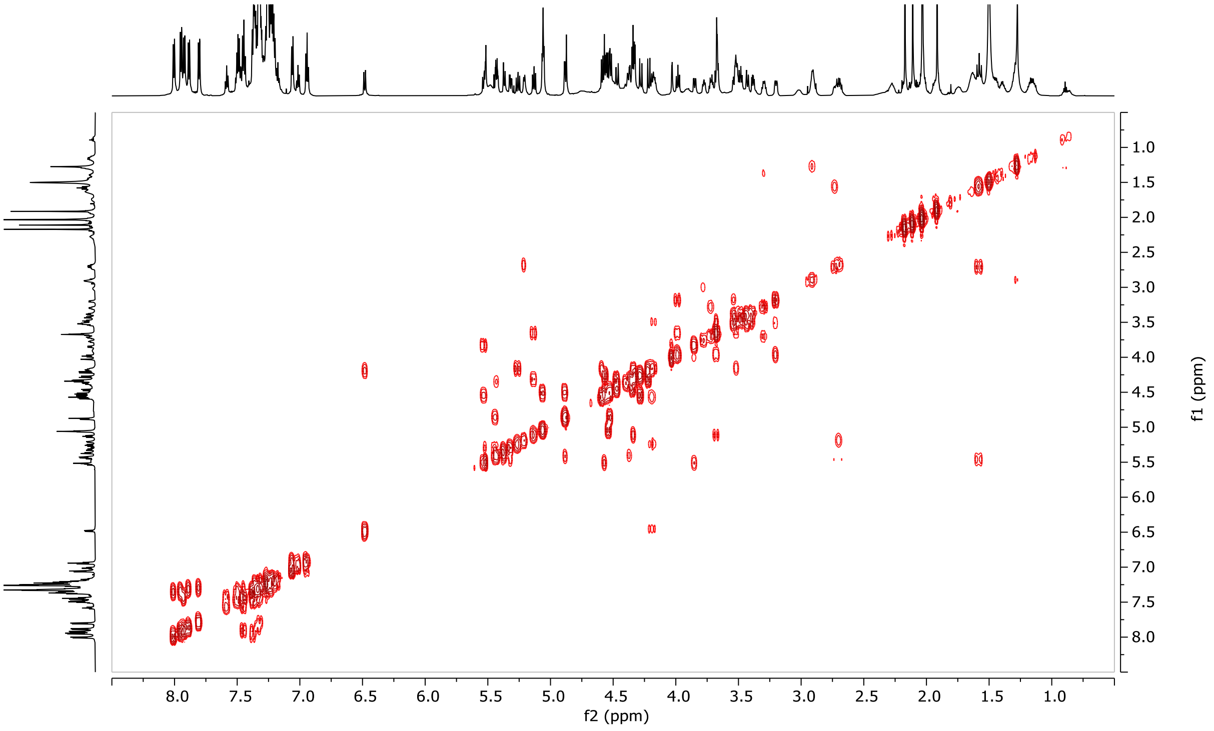

^{13}C-^{1}H HSQC

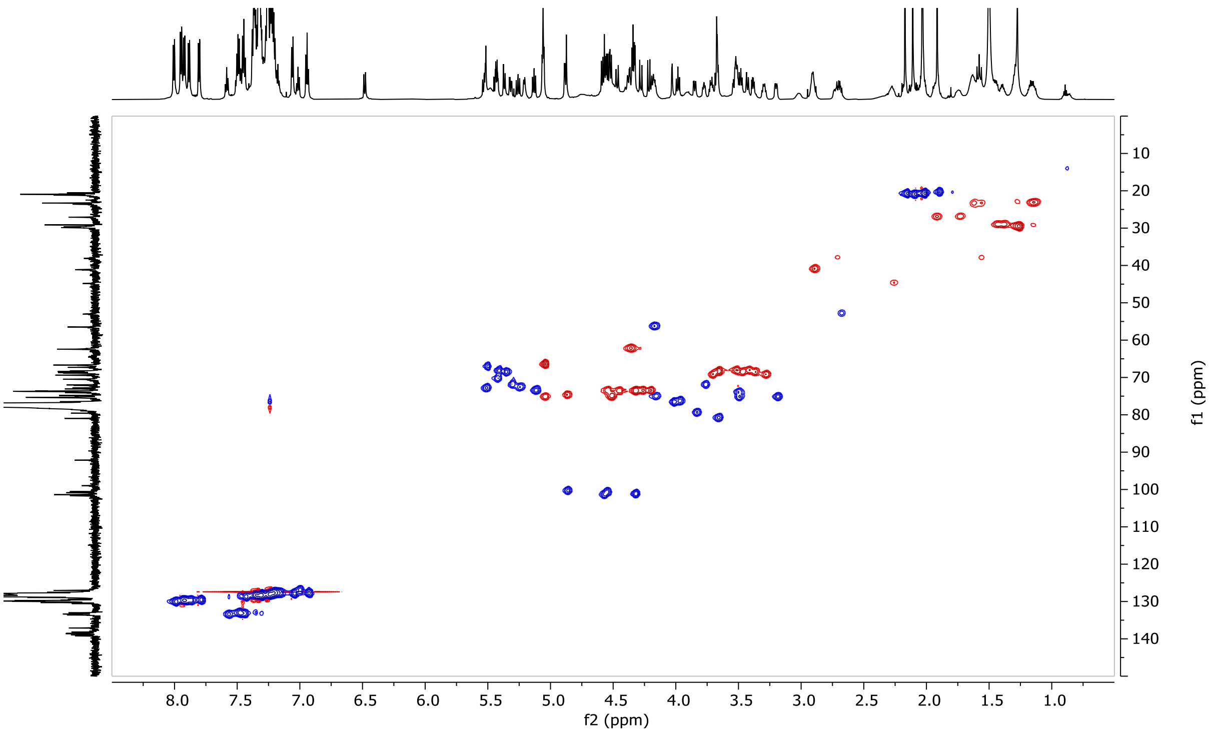

^{13}C-^{1}H HMBC

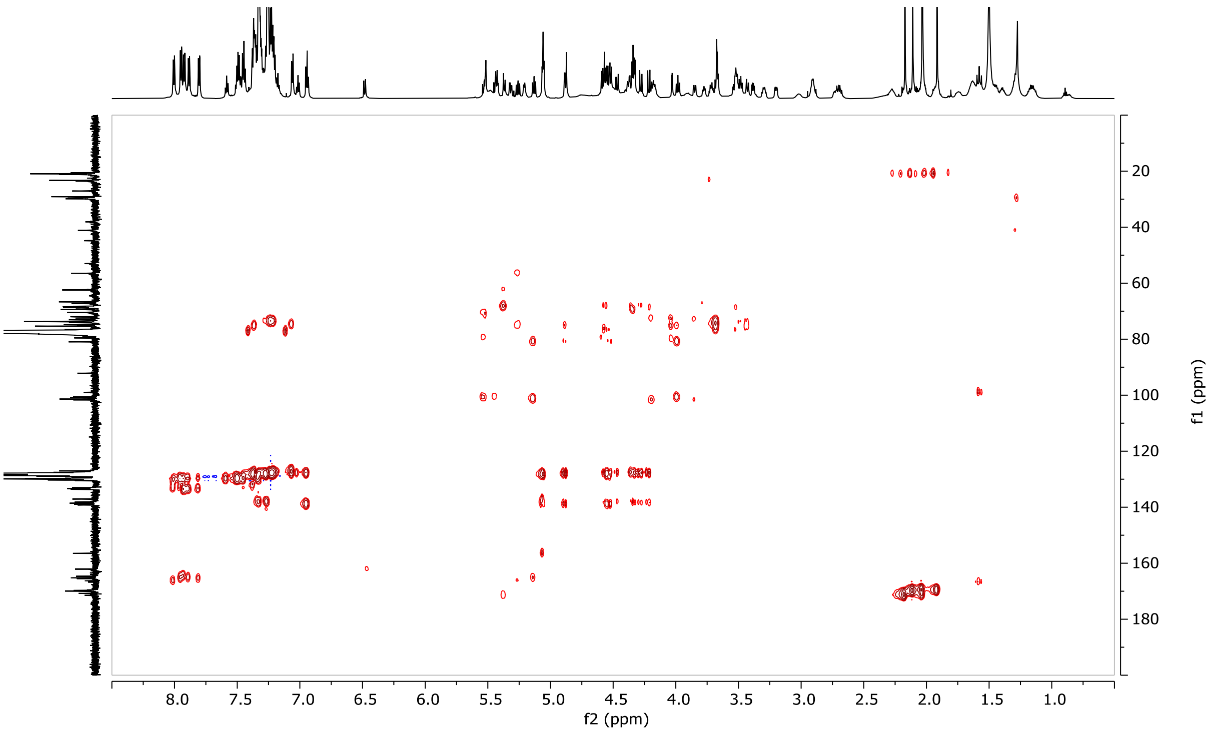

5.13 AGA of Pentasaccharide **S2**

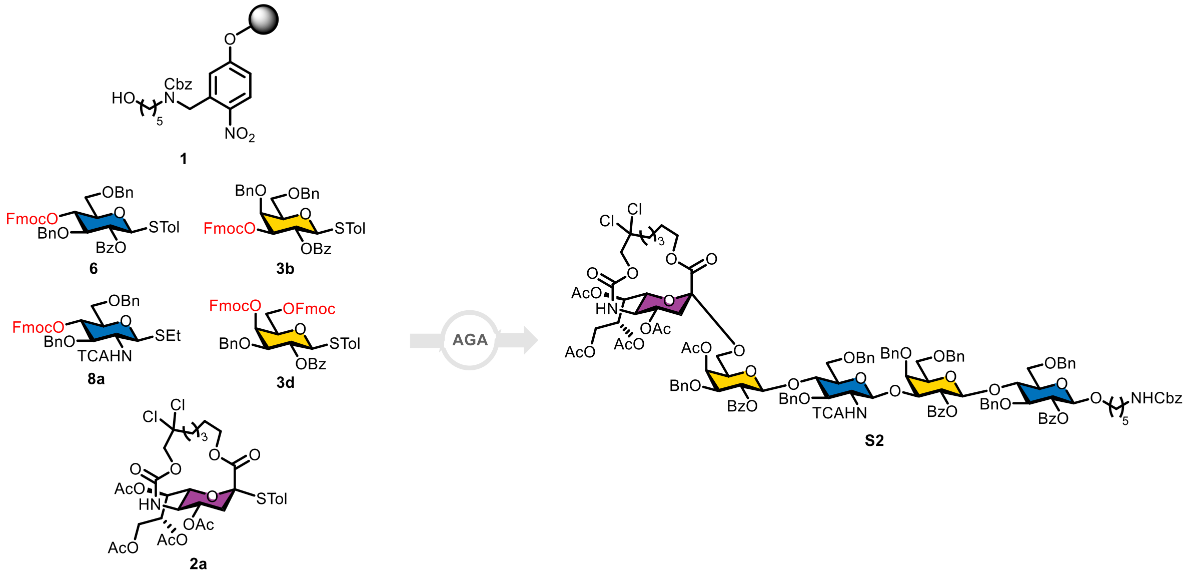

	Building blocks	Modules	Glycosylation condition
AGA		**Initiation** (40 mg resin)	
	Glc 6	Acidic wash<br>Thioglycoside glycosylation<br>Capping<br>Fmoc deprotection	8 eq. t (min)<br>T_1 = -20 °C 5<br>T_2 = 0 °C 20
	Gal 3b	Acidic wash<br>Thioglycoside glycosylation<br>Capping<br>Fmoc deprotection	8 eq. t (min)<br>T_1 = -40 °C 5<br>T_2 = -20 °C 20
	GlcN 8a	Acidic wash<br>Thioglycoside glycosylation<br>Capping<br>Fmoc deprotection	8 eq. t (min)<br>T_1 = -20 °C 5<br>T_2 = 0 °C 40
	Gal 3d	Acidic wash<br>Thioglycoside glycosylation<br>Capping<br>Fmoc deprotection	8 eq. t (min)<br>T_1 = -20 °C 5<br>T_2 = 0 °C 20
	Sia 2a	Acidic wash<br>Thioglycoside glycosylation<br>Pyridine wash (Acidic wash + Thioglycoside glycosylation x2)	10 eq. t (min)<br>T_1 = -40 °C 30<br>T_2 = -20 °C 10
Post AGA		**Photocleavage**<br>**Capping** (Ac_2O/pyridine = 1/1)<br>**NP-HPLC Purification**	

Protected compound **S2** (2.5 mg, 0.95 µmol, 7%) was obtained as a colorless oil by purification using preparative NP-HPLC (**Method N1**).

^{1}H NMR** (700 MHz, $CDCl_3$): δ 7.98 – 7.88 (m, 5H), 7.57 – 7.48 (m, 3H), 7.46 – 7.40 (m, 4H), 7.39 – 7.28 (m, 10H), 7.28 – 7.13 (m, 25H), 7.13 – 7.08 (m, 4H), 7.07 – 7.00 (m, 2H), 6.94 (t, *J* = 7.5 Hz, 2H), 6.76 (br, 1H), 5.56 – 5.43 (m, 2H), 5.38 – 5.20 (m, 4H), 5.14 (t, *J* = 8.7 Hz, 1H), 5.05 (s, 2H), 4.97 (d, *J* = 11.6 Hz, 1H), 4.89 (d, *J* = 11.1 Hz, 2H), 4.85 – 4.80 (m, 1H), 4.79 (d, *J* = 11.0 Hz, 1H), 4.69 – 4.61 (m, 4H), 4.59 – 4.48 (m, 4H), 4.45 (d, *J* = 11.6 Hz, 1H), 4.40 (t, *J* = 13.3 Hz, 2H), 4.35 – 4.18 (m, 7H), 4.13 (d, *J* = 11.8 Hz, 1H), 3.97 (dd, *J* = 18.3, 9.2 Hz, 4H), 3.82 (d, *J* = 32.8 Hz, 3H), 3.76 – 3.70 (m, 2H), 3.67 (t, *J* = 9.5 Hz, 2H), 3.60 – 3.46 (m, 6H), 3.45 – 3.35 (m, 4H), 3.34 – 3.24 (m, 3H), 3.17 (d, *J* = 9.8 Hz, 1H), 2.90 – 2.73 (m, 3H), 2.67 (td, *J* = 10.3, 6.1 Hz, 1H), 2.44 (br, 1H), 2.33 – 2.23 (m, 1H), 2.10 (s, 6H), 2.09 (s, 3H), 2.05 (s, 3H), 1.99 – 1.92 (m, 4H), 1.73 – 1.54 (m, 4H), 1.47 – 1.33 (m, 2H), 1.30 – 1.19 (m, 4H), 1.18 – 1.06 (m, 2H).

^{13}C NMR (176 MHz, $CDCl_3$): δ 171.0, 170.2, 170.2, 170.0, 169.8, 165.4, 165.2, 164.8, 161.6, 156.4, 154.1, 139.1, 138.7, 138.3, 138.2, 138.0, 137.5, 136.8, 133.6, 133.4, 133.1, 130.2, 130.0, 129.9, 129.8, 129.8, 128.8, 128.7, 128.6, 128.6, 128.5, 128.4, 128.3, 128.2, 128.2, 128.1, 127.9, 127.9, 127.9, 127.9, 127.8, 127.7, 127.7, 127.5, 127.4, 127.0, 101.2, 100.9, 100.7, 100.5, 92.2, 88.9, 80.7, 79.3, 78.2, 76.3, 76.0, 75.4, 75.0, 74.9, 74.8, 73.6, 73.5, 73.4, 72.6, 71.7, 71.1, 69.5, 68.8, 68.4, 68.1, 67.8, 66.6, 65.6, 62.1, 56.9, 53.0, 44.9, 40.9, 38.0, 29.9, 29.5, 28.9, 26.8, 23.2, 21.2, 21.0, 21.0, 20.9.

HRMS (QToF): Calcd for $C_{136}H_{148}Cl_5N_3NaO_{40}$ $[M + Na]^+$ 2660.7979; found 2660.8091.

Crude analytical NP-HPLC (t_R = 28.0 min)

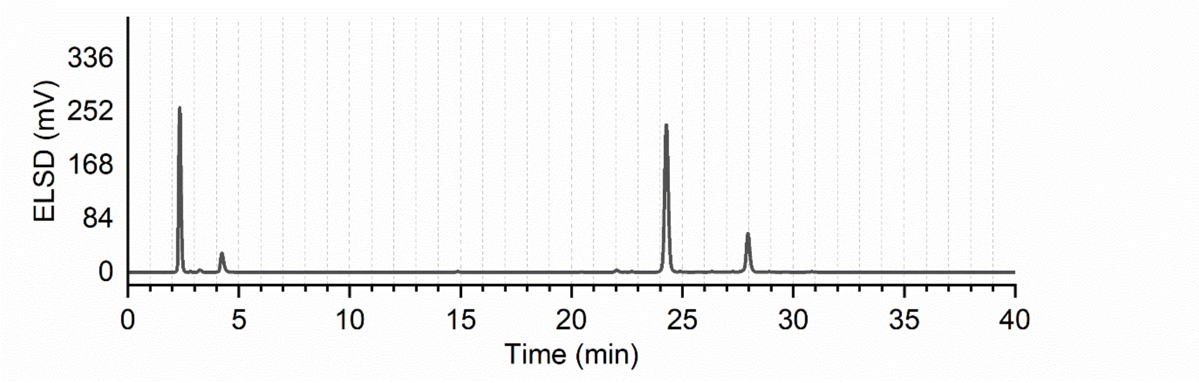

^{1}H-NMR (700 MHz, $CDCl_3$)

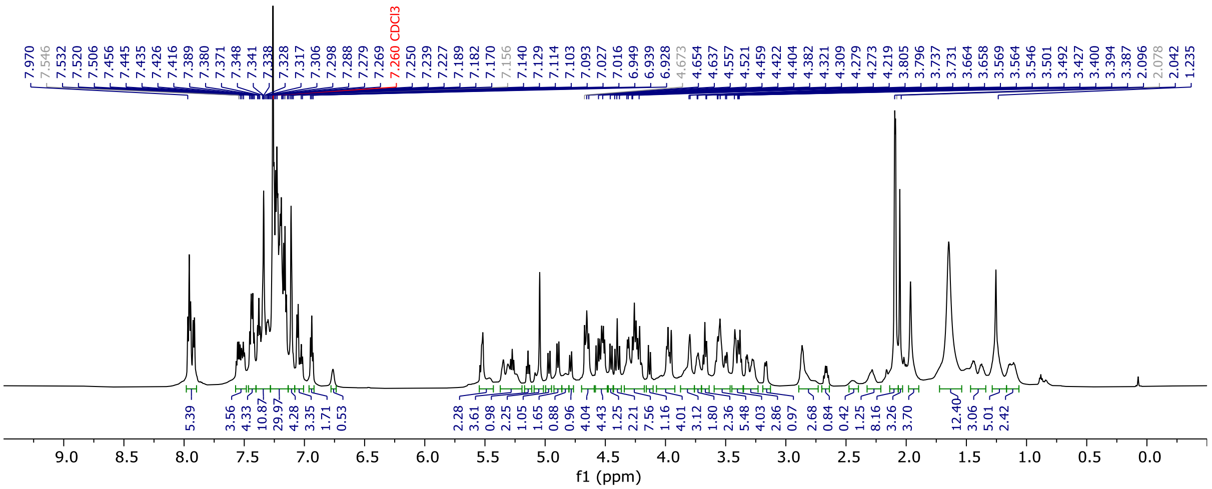

^{13}C-NMR (176 MHz, $CDCl_3$)

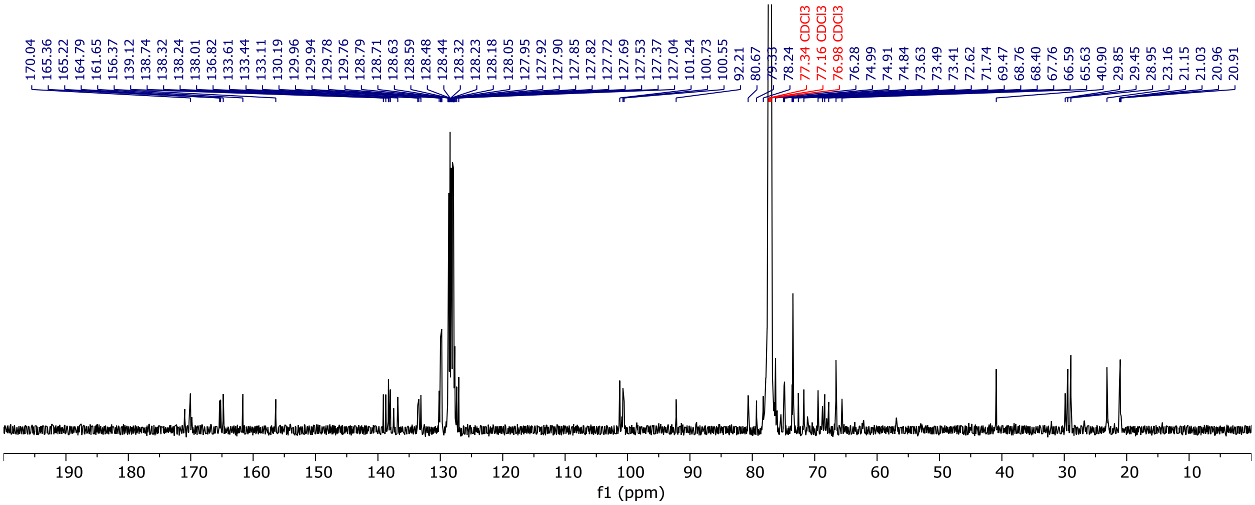

^{1}H -^{1}H COSY

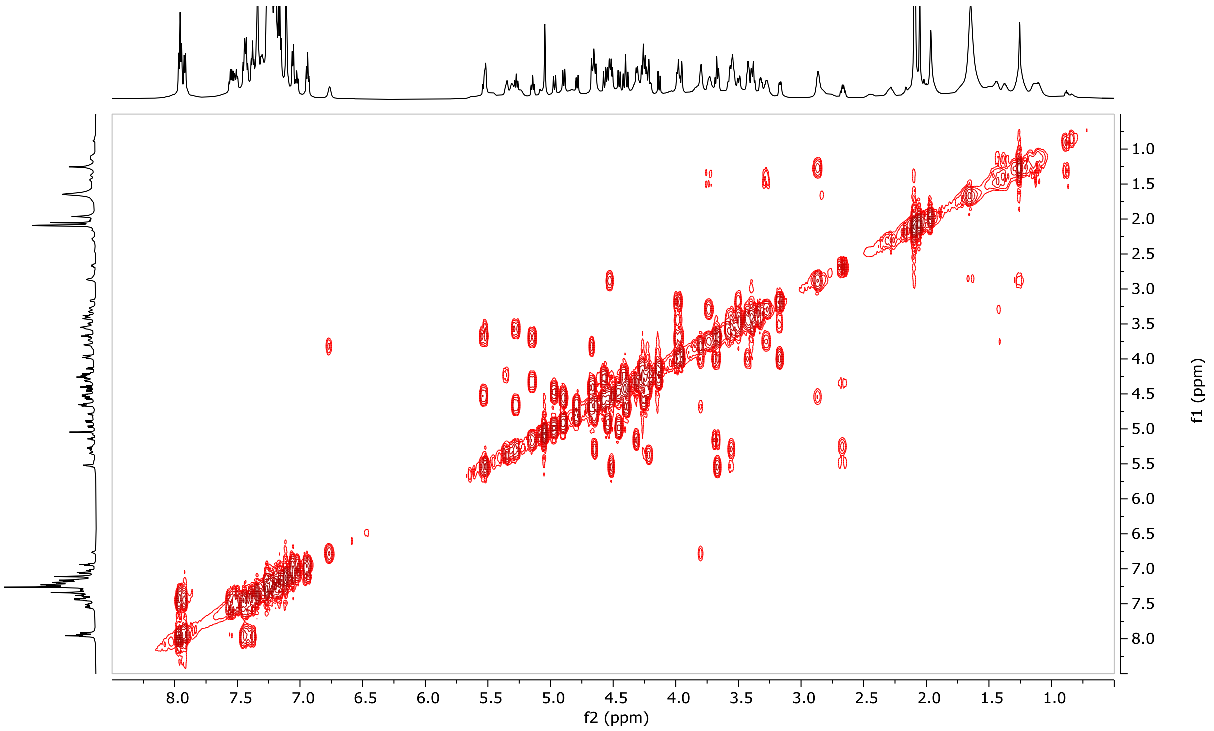

^{13}C-^{1}H HSQC

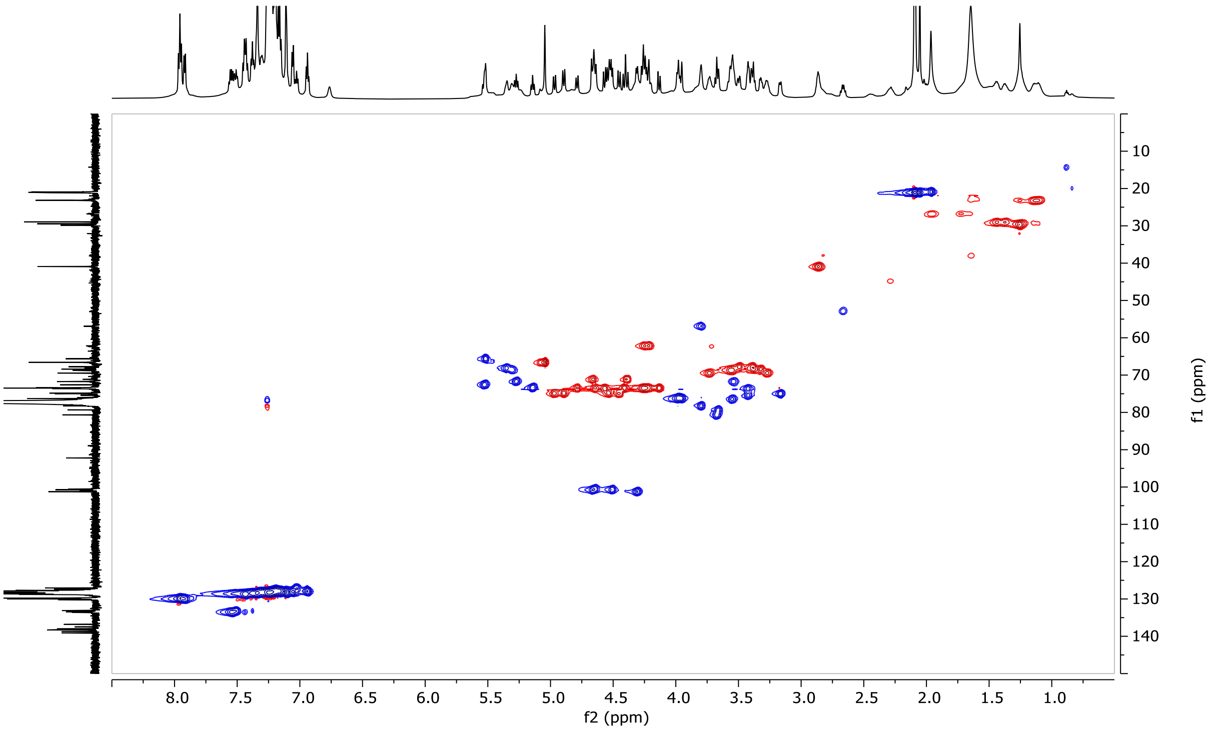

^{13}C-^{1}H HMBC

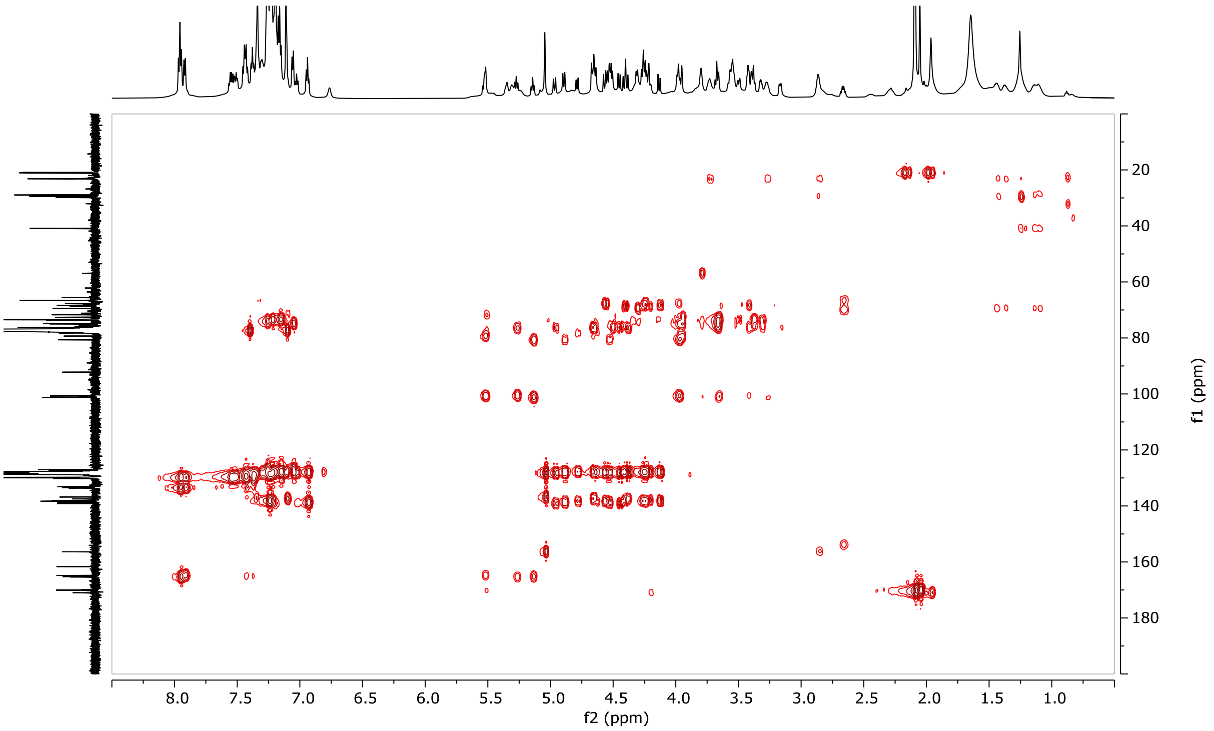

5.14 AGA of Pentasaccharide **S3**

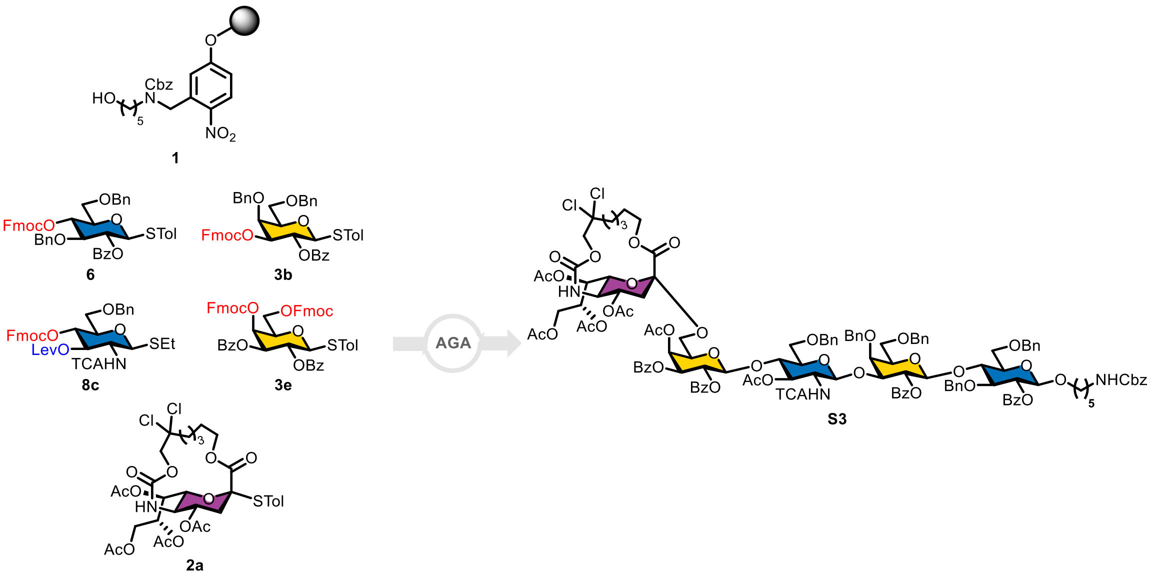

	Building blocks	Modules	Glycosylation condition
AGA		**Initiation** (40 mg resin)	
	**Glc 6**	**Acidic wash**<br>**Thioglycoside glycosylation**<br>**Capping**<br>**Fmoc deprotection**	**8 eq.** / **t (min)**<br>**T_1 = -20 °C** / **5**<br>**T_2 = 0 °C** / **20**

	**Gal 3b**	**Acidic wash**<br>**Thioglycoside glycosylation**<br>**Capping**<br>**Fmoc deprotection**		**8 eq.** | **t (min)**<br>T_1 = -40 °C | 5<br>T_2 = -20 °C | 20
	**GlcN 8c**	**Acidic wash**<br>**Thioglycoside glycosylation**<br>**Capping**<br>**Fmoc deprotection**		**8 eq.** | **t (min)**<br>T_1 = -20 °C | 5<br>T_2 = 0 °C | 40
	**Gal 3e**	**Acidic wash**<br>**Thioglycoside glycosylation**<br>**Capping**<br>**Lev deprotection**<br>**Fmoc deprotection**		**8 eq.** | **t (min)**<br>T_1 = -20 °C | 5<br>T_2 = 0 °C | 20
	**Sia 2a**	**Acidic wash**<br>**Thioglycoside glycosylation**<br>**Pyridine wash**	**x2**	**10 eq.** | **t (min)**<br>T_1 = -40 °C | 30<br>T_2 = -20 °C | 10
**Post AGA**		**Photocleavage**<br>**Capping** (Ac_2O/pyridine = 1/1)<br>**NP-HPLC Purification**		

Protected compound **S3** (4.1 mg, 1.58 µmol, 12%) was obtained as a colorless oil by purification using preparative NP-HPLC (**Method N1**).

^{1}**H NMR** (700 MHz, $CDCl_3$, 50 °C): δ 7.96 – 7.93 (m, 2H), 7.92 – 7.88 (m, 2H), 7.87 – 7.80 (m, 2H), 7.58 (td, *J* = 7.4, 1.3 Hz, 1H), 7.52 – 7.46 (m, 3H), 7.45 – 7.42 (m, 2H), 7.39 – 7.30 (m, 17H), 7.26 (s, 15H), 7.15 (tt, *J* = 6.8, 1.6 Hz, 1H), 7.08 – 7.04 (m, 2H), 7.03 – 6.99 (m, 1H), 6.95 (t, *J* = 7.4 Hz, 2H), 6.32 (d, *J* = 9.2 Hz, 1H), 5.65 (d, *J* = 3.5 Hz, 1H), 5.55 – 5.46 (m, 3H), 5.40 (dd, *J* = 10.5, 3.6 Hz, 1H), 5.37 – 5.31 (m, 2H), 5.19 (d, *J* = 6.6 Hz, 1H), 5.13 (dd, *J* = 9.2, 7.9 Hz, 1H), 5.06 (s, 3H), 5.02 (d, *J* = 11.4 Hz, 1H), 4.94 (dd, *J* = 10.6, 8.7 Hz, 1H), 4.87 (d, *J* = 11.2 Hz, 1H), 4.83 (d, *J* = 7.8 Hz, 1H), 4.58 – 4.46 (m, 7H), 4.44 (d, *J* = 12.0 Hz, 1H), 4.37 – 4.24 (m, 7H), 4.20 (d, *J* = 11.8 Hz, 1H), 4.05 – 3.90 (m, 6H), 3.82 (dd, *J* = 10.2, 3.1 Hz, 1H), 3.79 – 3.74 (m, 1H), 3.72 (dt, *J* = 10.0, 5.9 Hz, 1H), 3.67 (t, *J* = 8.7 Hz, 1H), 3.61 (dd, *J* = 11.0, 4.6 Hz, 1H), 3.58 (dd, *J* = 10.9, 2.1 Hz, 1H), 3.55 – 3.44 (m, 4H), 3.42 (dd, *J* = 11.0, 2.0 Hz, 1H), 3.40 – 3.34 (m, 2H), 3.32 – 3.26 (m, 1H), 3.20 (ddd, *J* = 9.7, 4.2, 2.1 Hz, 1H), 2.94 – 2.85 (m, 3H), 2.79 (dd, *J* = 13.0, 3.3 Hz, 1H), 2.67 (td, *J* = 10.4, 6.5 Hz, 1H), 2.41 – 2.22 (m, 2H), 2.16 (s, 3H), 2.10 (s, 3H), 2.08 (s, 3H), 2.04 (s, 6H), 2.01 (s, 3H), 1.96 – 1.89 (m, 1H), 1.73 (t, *J* = 10.9 Hz, 1H), 1.68 – 1.54 (m, 3H), 1.48 (s, 2H), 1.33 – 1.23 (m, 4H), 1.21 – 1.10 (m, 2H).

^{13}C NMR (176 MHz, $CDCl_3$, 50 °C): δ 170.9, 170.7, 170.0, 169.9, 169.8, 166.7, 165.5, 165.3, 165.2, 164.7, 162.2, 156.5, 154.4, 139.2, 139.0, 138.6, 138.5, 138.1, 137.1, 133.7, 133.5, 133.4, 133.0, 130.6, 129.9, 129.8, 129.0, 129.0, 128.8, 128.7, 128.6, 128.5, 128.3, 128.2, 128.1, 128.0, 128.0, 127.9, 127.9, 127.8, 127.5, 127.0, 101.5, 101.4, 101.1, 100.9, 99.1, 92.3, 81.0, 79.4, 76.7, 76.5, 75.7, 75.3, 74.8, 74.0, 73.7, 73.2, 72.5, 72.1, 72.0, 70.4, 69.4, 68.7, 68.2, 68.0, 67.6, 66.7, 62.4, 56.3, 52.8, 41.1, 38.3, 30.0, 29.9, 29.6, 29.1, 27.0, 23.6, 23.3, 21.1, 21.0, 20.9, 20.7.

HRMS (QToF): Calcd for $C_{131}H_{142}Cl_5N_3NaO_{42}$ $[M + Na]^+$ 2626.7408; found 2626.7512.

Crude analytical NP-HPLC (t_R = 28.5 min)

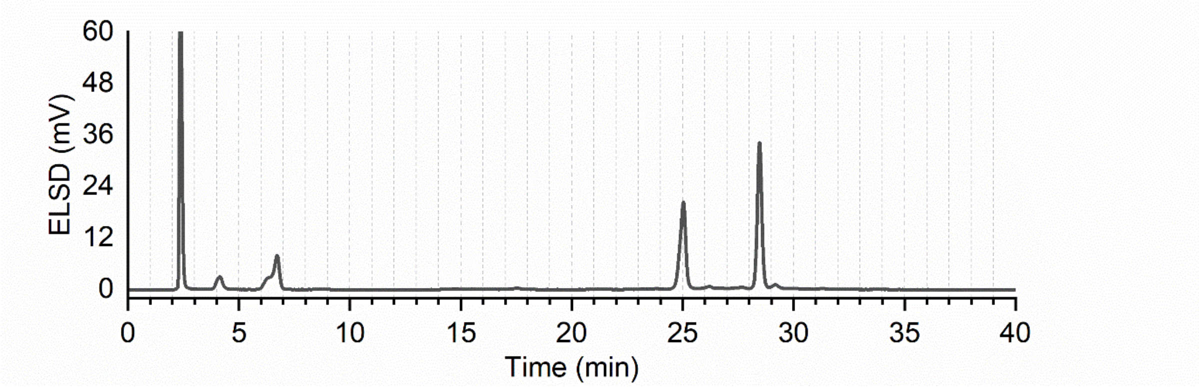

^{1}H-NMR (700 MHz, $CDCl_3$, 50 °C)

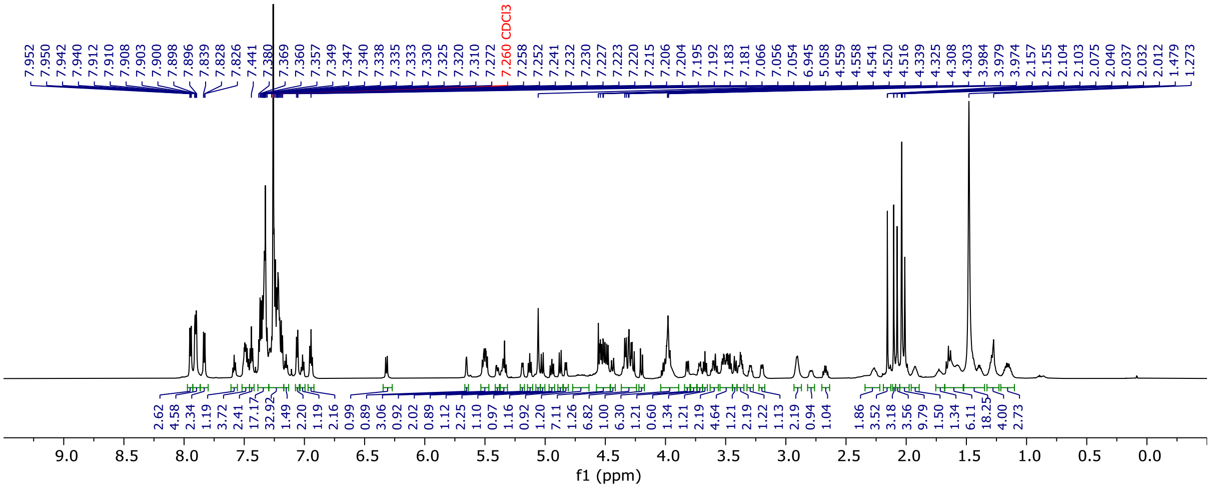

^{13}C-NMR (176 MHz, $CDCl_3$, 50 °C)

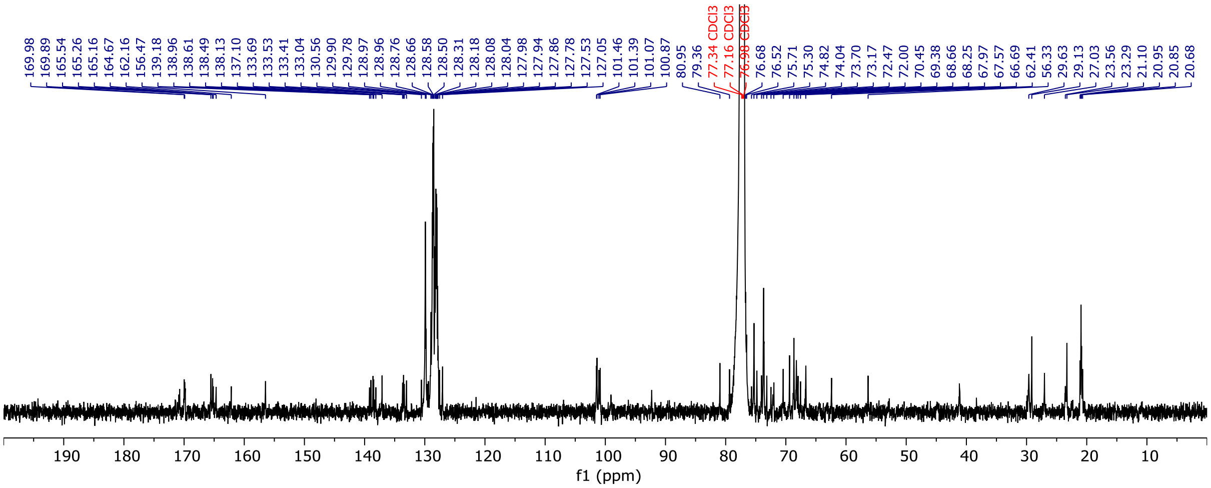

^{1}H -^{1}H COSY

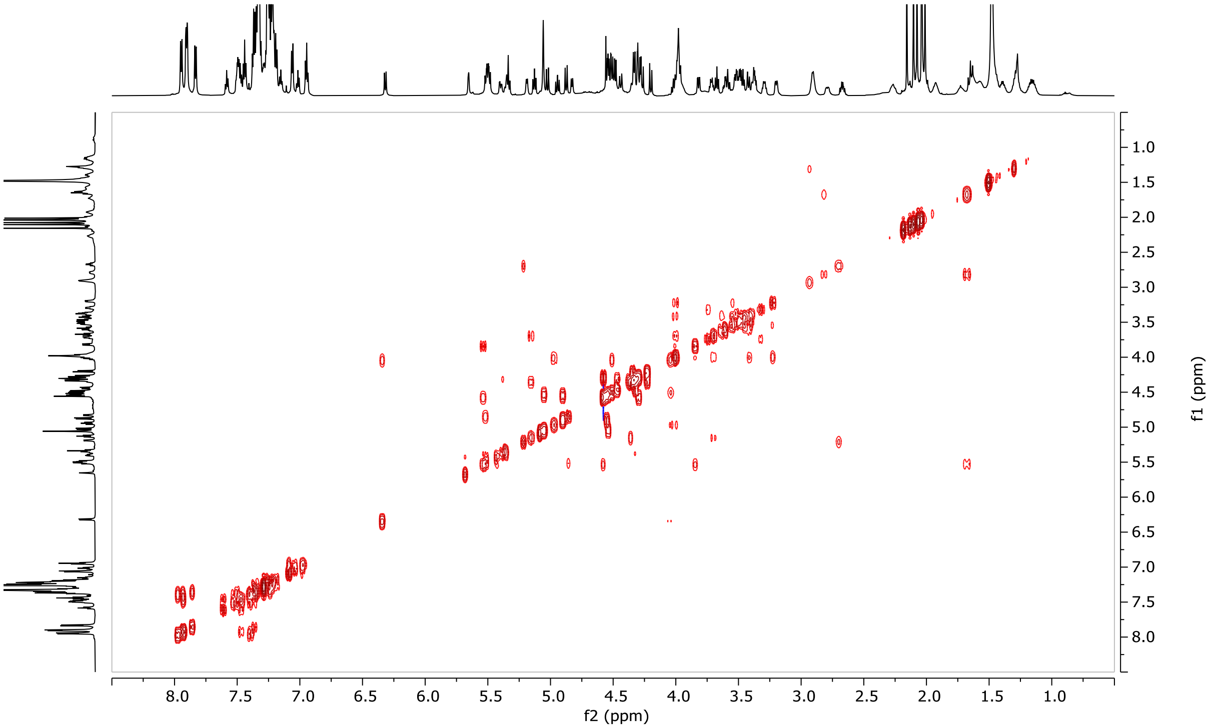

^{13}C-^{1}H HSQC

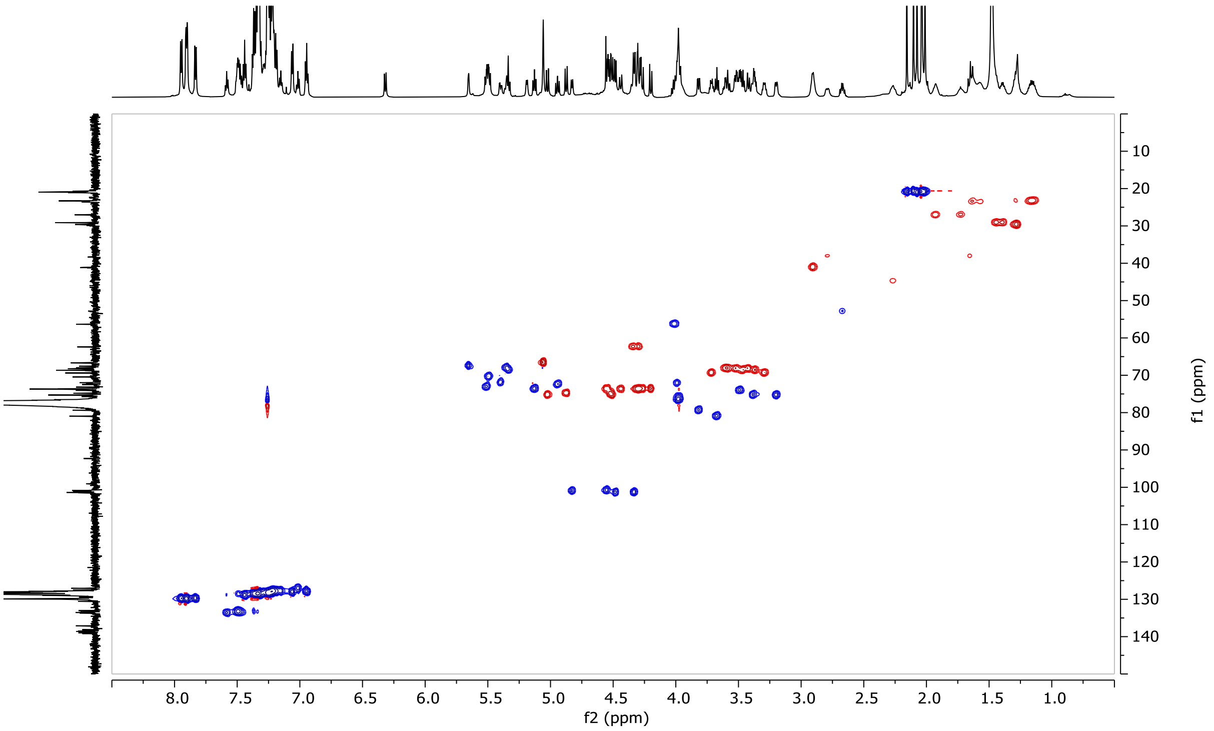

^{13}C-^{1}H HMBC

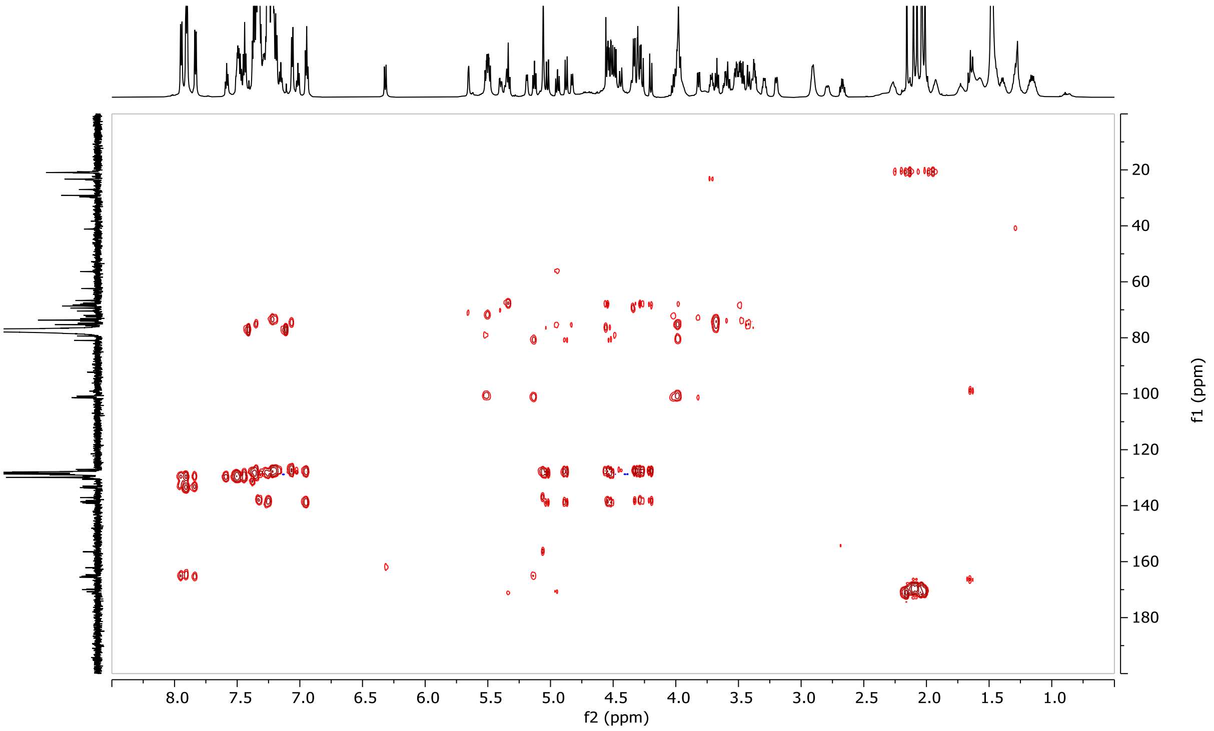

6 Global Deprotection of Sialylated Glycans

6.1 Disaccharide 19

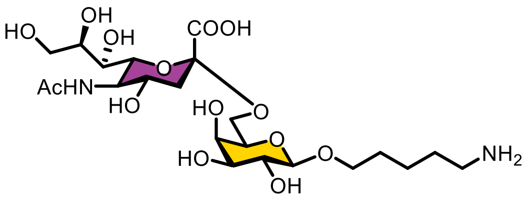

19

Unprotected disaccharide **19** (5.5 mg, 1.45 µmol, 64%) was obtained as a colorless oil after global deprotection (**Method C**), followed by purification using preparative RP-HPLC (**Method R2**).

^{1}H NMR (600 MHz, D_2O)**:** δ 4.39 (d, *J* = 7.9 Hz, 1H), 3.96 – 3.91 (m, 3H), 3.91 – 3.86 (m, 2H), 3.83 (t, *J* = 10.1 Hz, 1H), 3.77 (ddd, *J* = 7.6, 4.7, 1.1 Hz, 1H), 3.74 – 3.67 (m, 3H), 3.67 – 3.61 (m, 3H), 3.59 (dd, *J* = 8.8, 1.8 Hz, 1H), 3.49 (dd, *J* = 9.9, 7.9 Hz, 1H), 3.02 (t, *J* = 7.5 Hz, 2H), 2.74 (dd, *J* = 12.5, 4.7 Hz, 1H), 2.04 (s, 3H), 1.74 – 1.66 (m, 5H), 1.50 – 1.44 (m, 2H).

^{13}C NMR (151 MHz, D_2O)**:** δ 175.1, 173.3, 102.9, 100.5, 73.4, 72.7, 72.6, 71.8, 70.7, 70.3, 68.6, 68.2, 68.2, 63.4, 62.6, 51.8, 40.2, 39.4, 28.2, 26.4, 22.0.

HRMS (QToF)**:** Calcd for $C_{22}H_{39}N_2O_{14}$ $[M - H]^-$ 555.2401; found 555.2405.

Crude analytical RP-HPLC (t_R = 32.9 min)

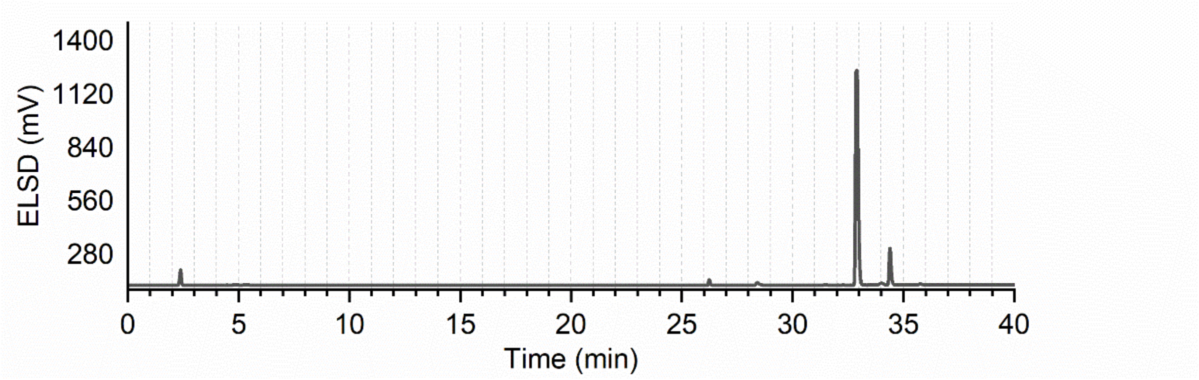

Purified analytical RP-HPLC

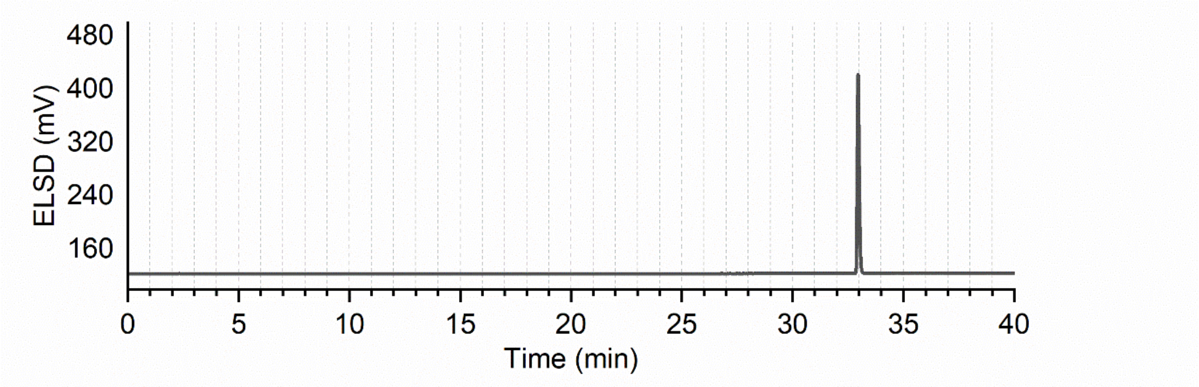

^{1}H-NMR (600 MHz, D_2O)

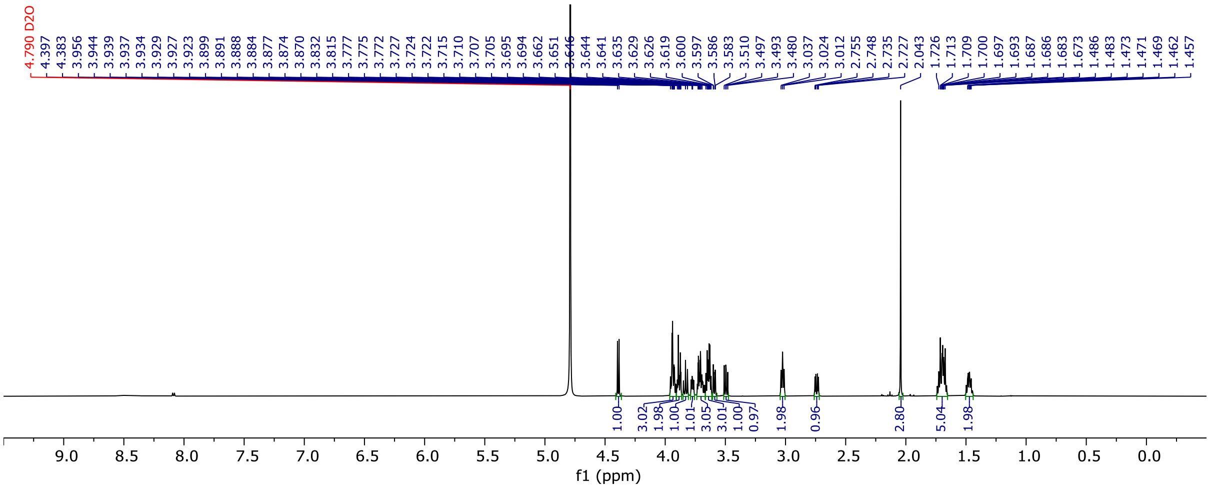

^{13}C-NMR (151 MHz, D_2O)

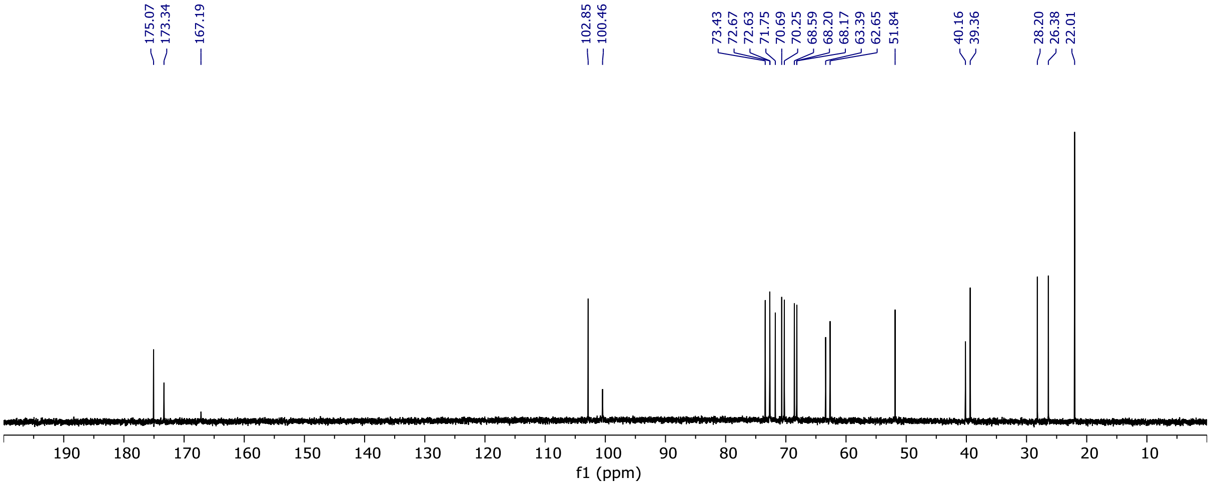

^{1}H -^{1}H COSY

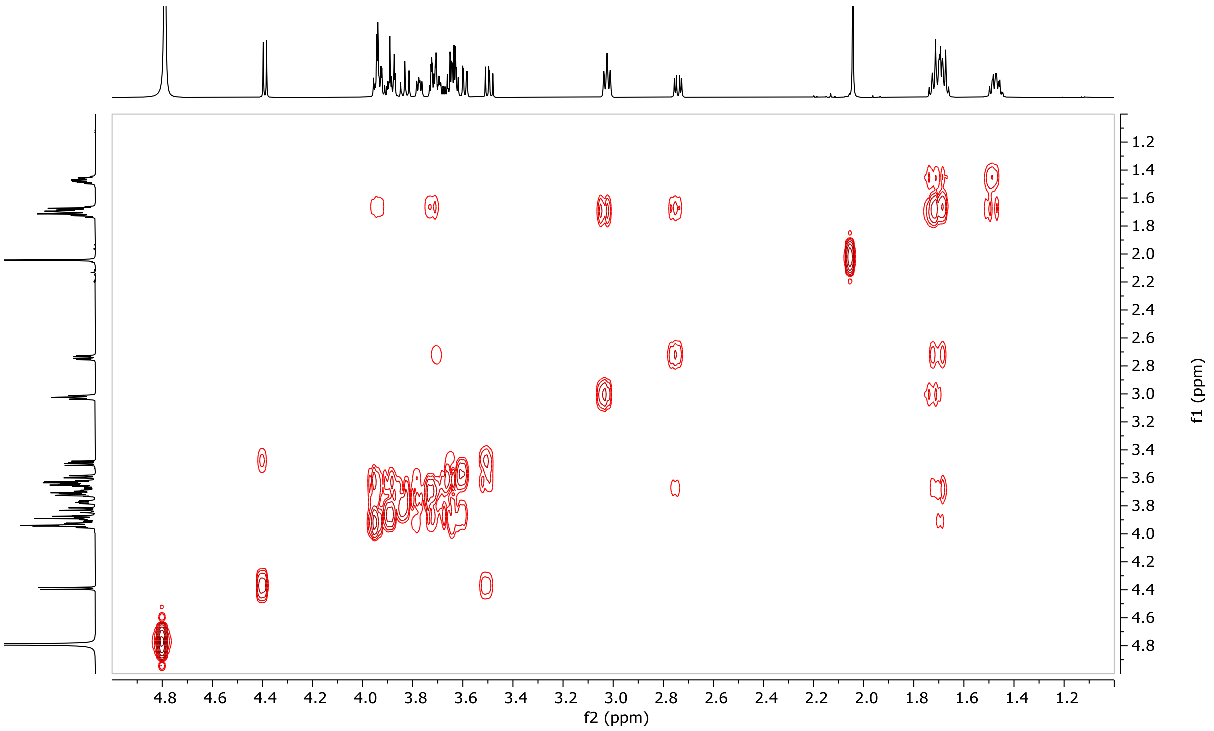

^{13}C-^{1}H HSQC

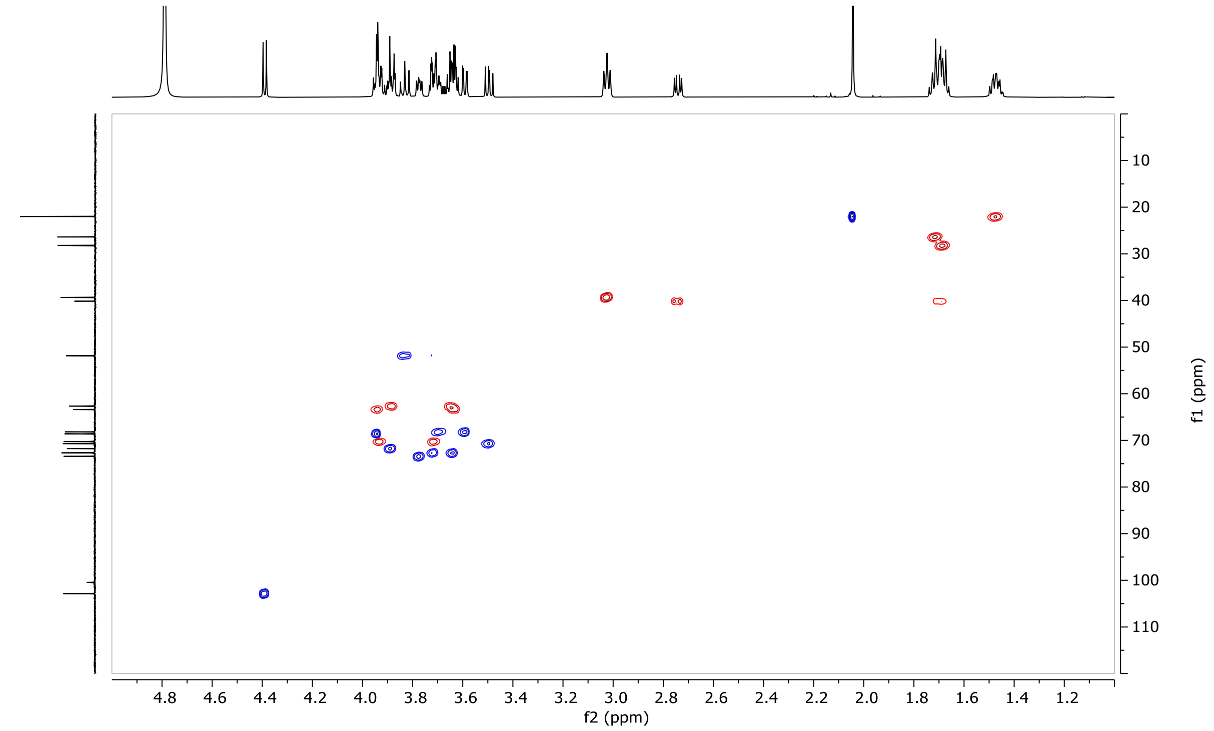

^{13}C-^{1}H HMBC

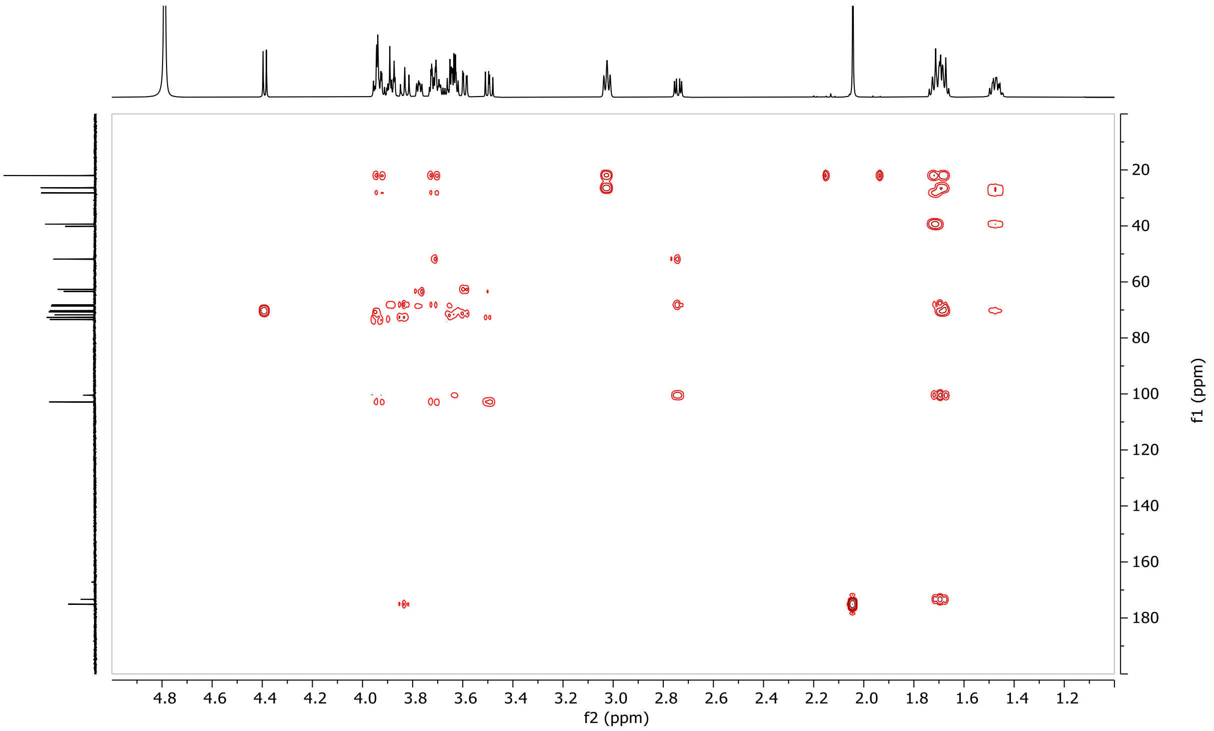

Expansion of ^{13}C-^{1}H HMBC

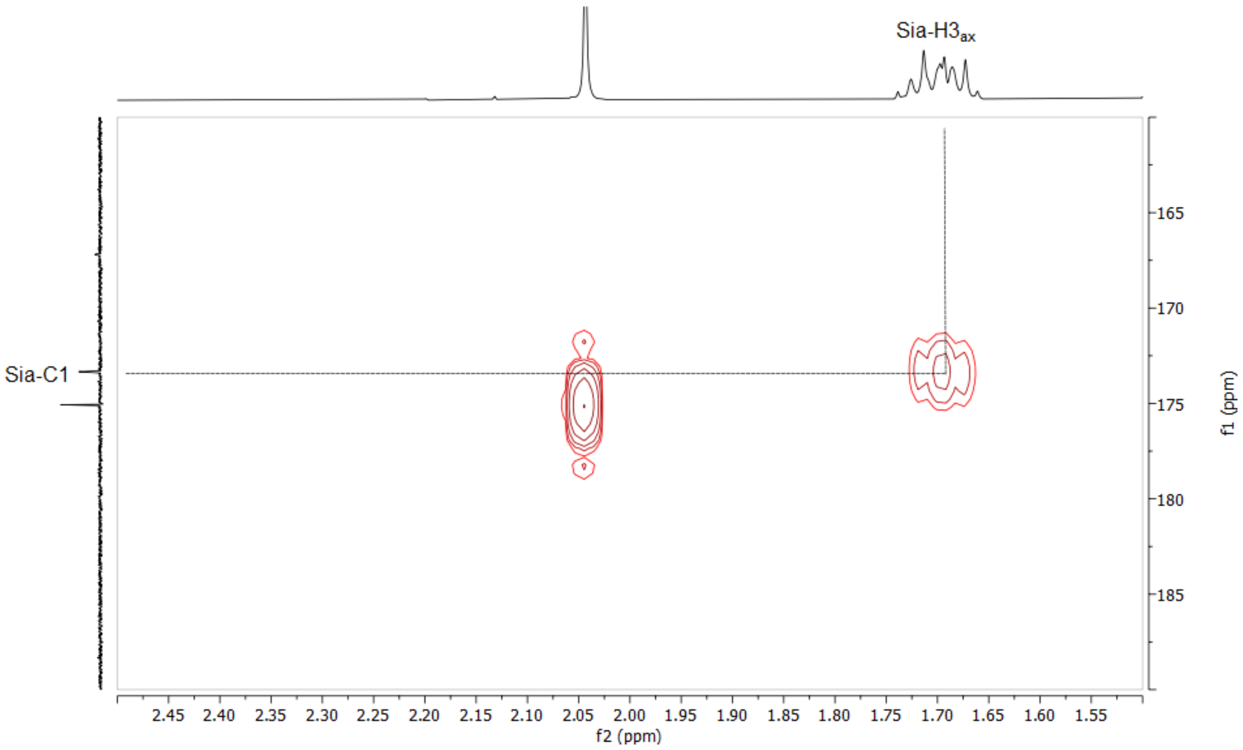

6.2 Sialyllacto-*N*-neotetraose c (LSTc) **20**

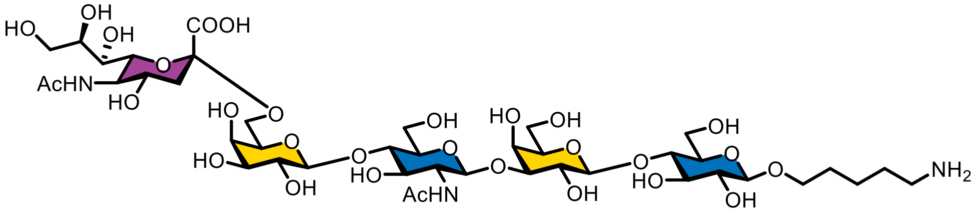

20

Unprotected pentasaccharide **20** (1.6 mg, 1.45 µmol, 64%) was obtained as a colorless oil after global deprotection (**Method C**), followed by purification using preparative RP-HPLC (**Method R1**).

^{1}H NMR (600 MHz, D_2O)**:** δ 4.73 (d, *J* = 7.5 Hz, 1H), 4.49 (d, *J* = 8.0 Hz, 1H), 4.46 (d, *J* = 8.0 Hz, 1H), 4.44 (d, *J* = 7.9 Hz, 1H), 4.16 (d, *J* = 3.3 Hz, 1H), 4.02 – 3.95 (m, 3H), 3.95 – 3.91 (m, 2H), 3.91 – 3.84 (m, 3H), 3.84 – 3.76 (m, 6H), 3.76 – 3.70 (m, 4H), 3.70 – 3.67 (m, 2H), 3.67 – 3.62 (m, 5H), 3.62 – 3.57 (m, 3H), 3.57 – 3.51 (m, 3H), 3.30 (t, *J* = 8.5 Hz, 1H), 3.01 (t, *J* = 7.5 Hz, 2H), 2.67 (dd, *J* = 12.4, 4.7 Hz, 1H), 2.05 (s, 3H), 2.03 (s, 3H), 1.70 (ddd, *J* = 21.5, 16.8, 9.5 Hz, 5H), 1.46 (p, *J* = 7.8 Hz, 2H).

^{13}C NMR (151 MHz, D_2O)**:** δ 174.9, 173.5, 103.4, 102.9, 102.5, 102.0, 100.1, 82.0, 80.5, 78.4, 74.9, 74.8, 74.4, 74.2, 73.7, 72.8, 72.5, 72.4, 72.2, 71.7, 70.7, 70.0, 69.9, 68.4, 68.3, 68.3, 68.2, 63.3, 62.6, 60.9, 60.1, 60.0, 54.9, 51.8, 40.1, 39.3, 28.1, 26.4, 22.3, 22.0, 22.0.

HRMS (QToF)**:** Calcd for $C_{42}H_{72}N_3O_{29}$ $[M - H]^-$ 1082.4257; found 1082.4159.

Crude analytical RP-HPLC (t_R = 29.8 min)

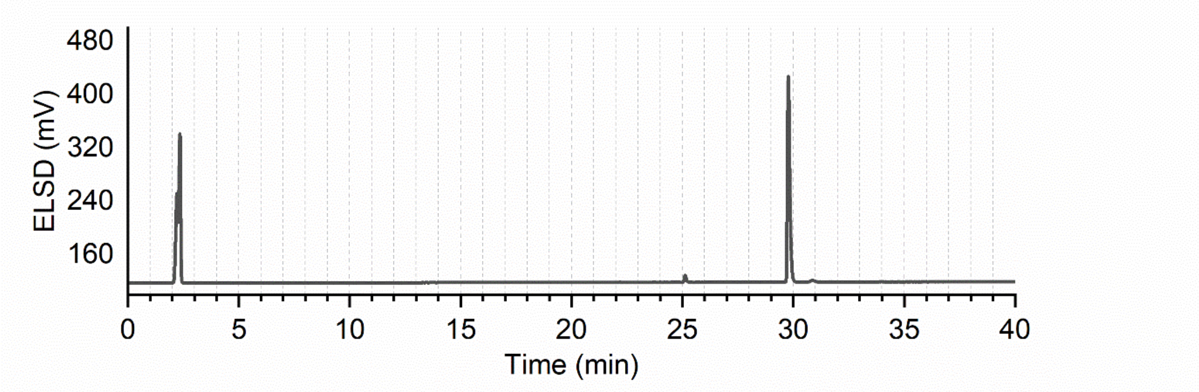

Purified analytical RP-HPLC

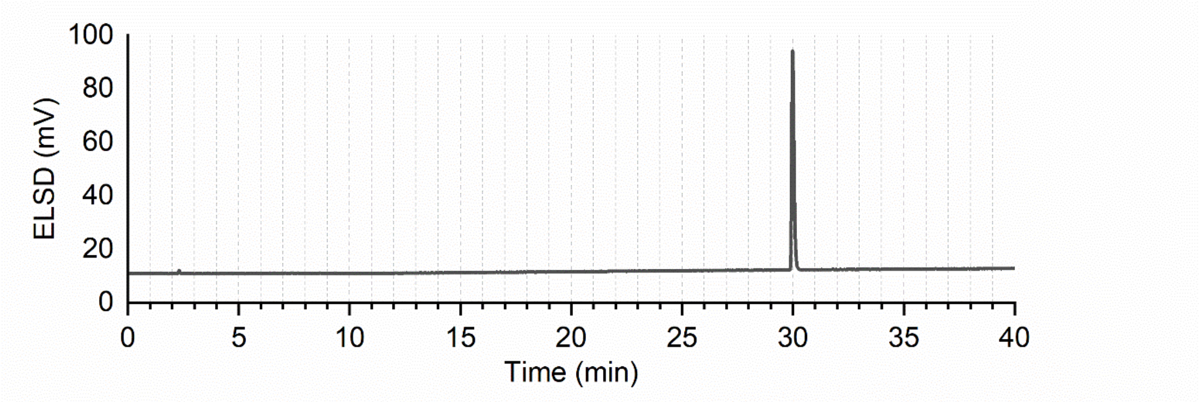

^{1}H-NMR (600 MHz, D_2O)

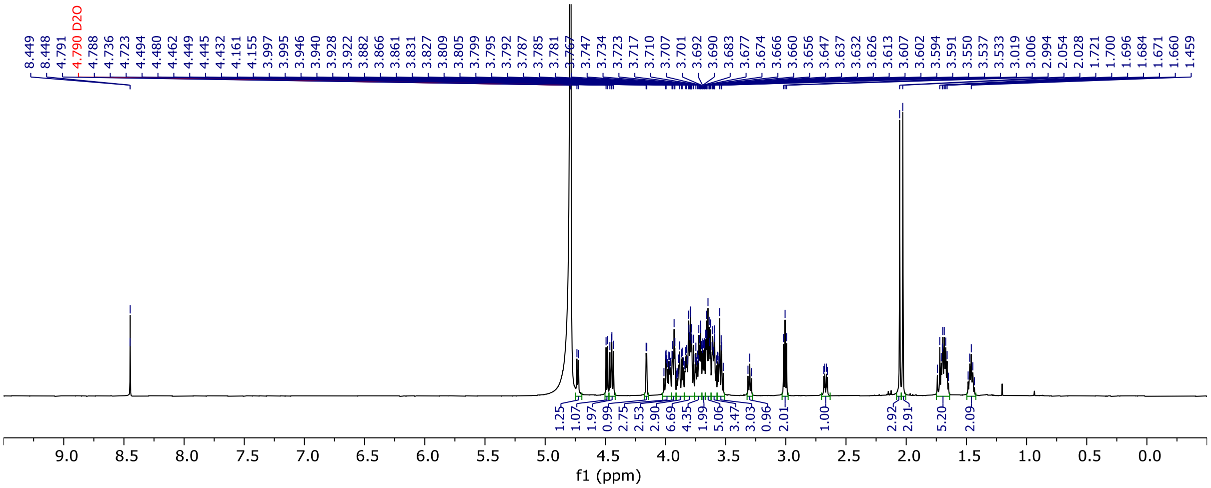

^{13}C-NMR (151 MHz, D_2O)

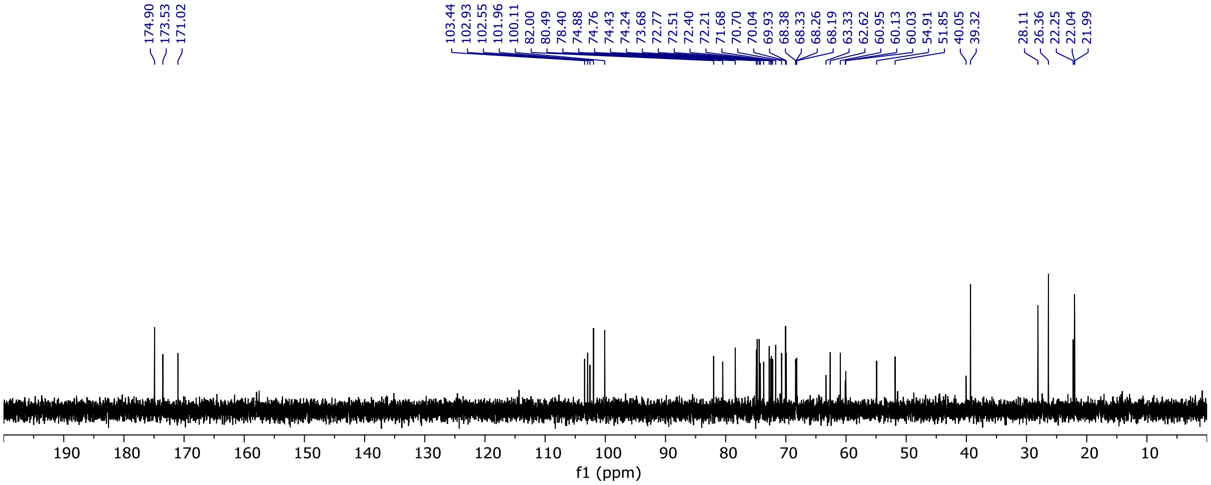

^{1}H -^{1}H COSY

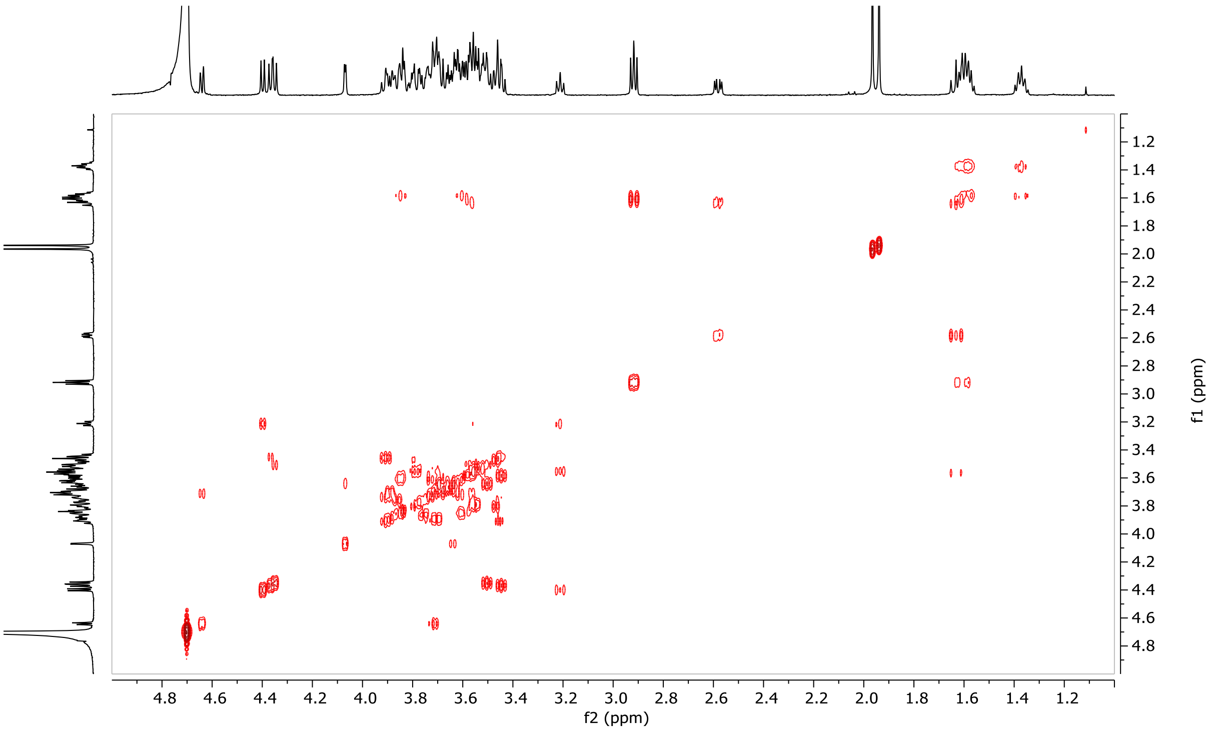

^{13}C-^{1}H HSQC

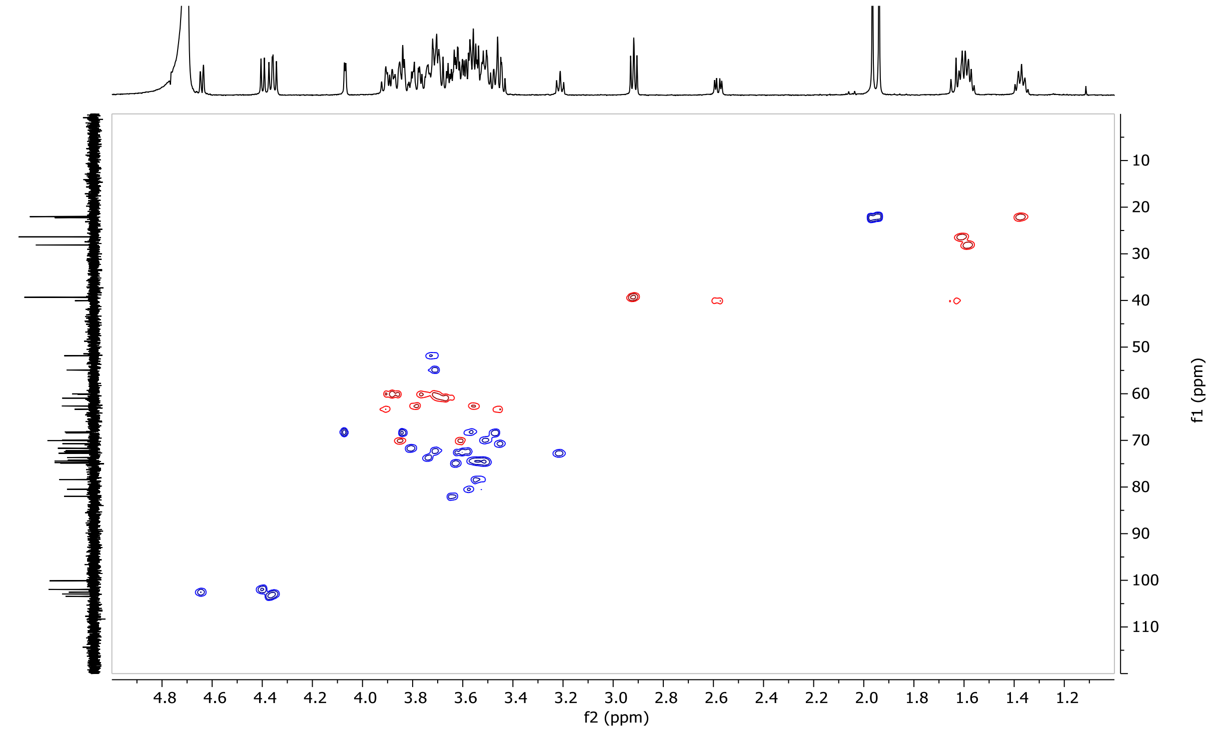

^{13}C-^{1}H HMBC

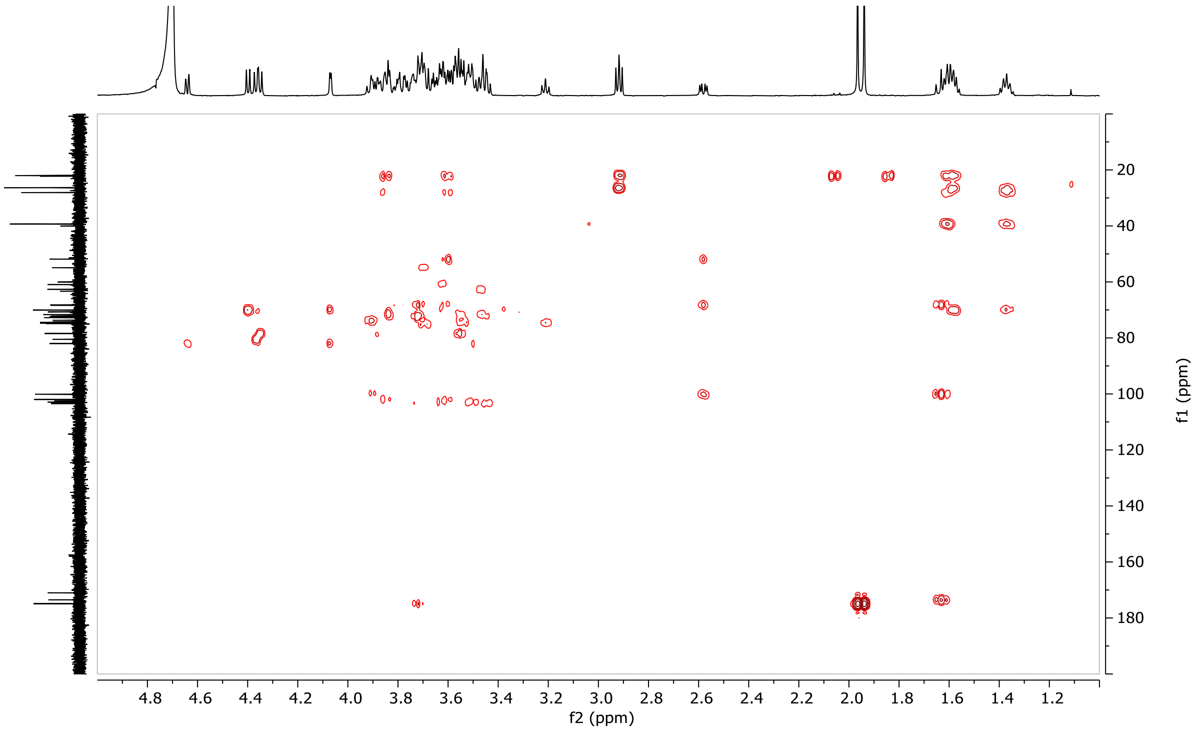

Expansion of ^{13}C-^{1}H HMBC

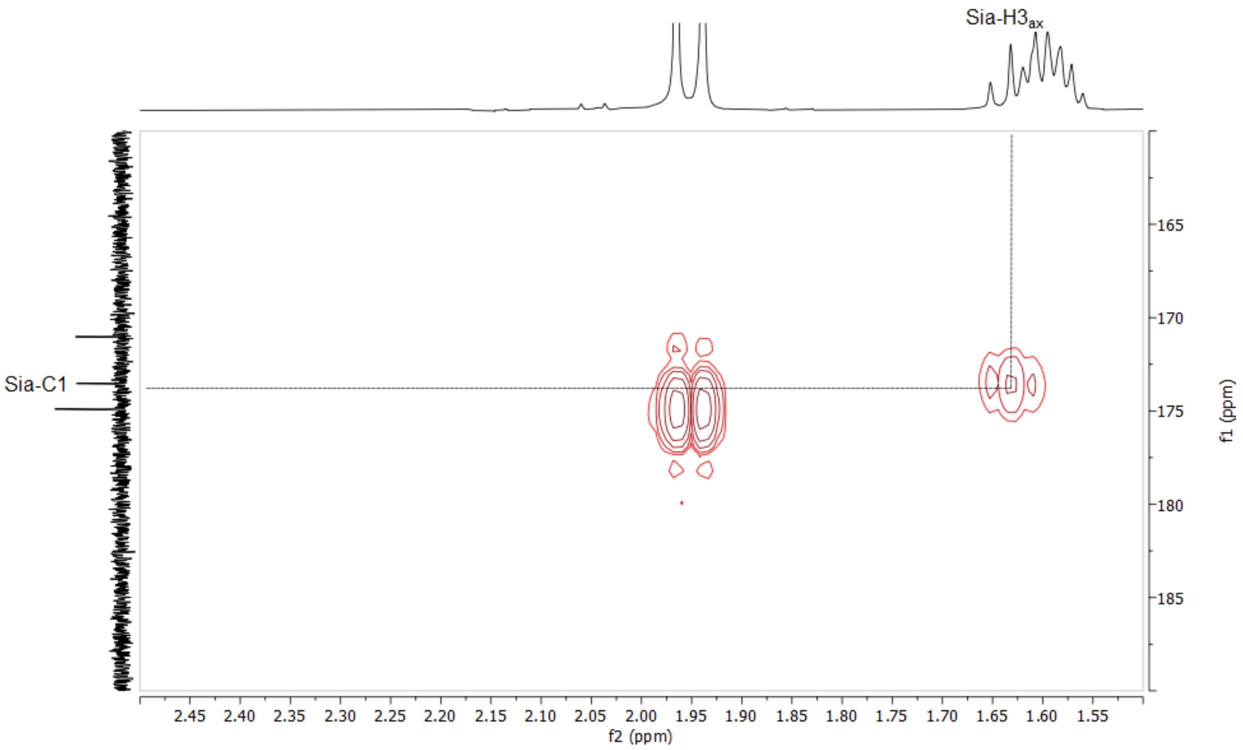

6.3 Sialyllacto-*N*-neotetraose **21**

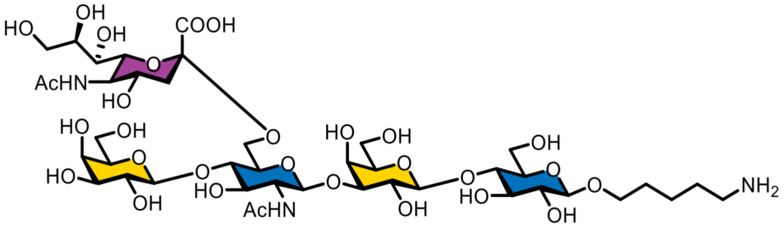

21

Unprotected pentasaccharide **21** (3.0 mg, 2.80 µmol, 62%) was obtained as a colorless oil after global deprotection (**Method C**), followed by purification using preparative RP-HPLC (**Method R1**).

^{1}H NMR (600 MHz, D_2O)**:** δ 4.69 (d, *J* = 8.4 Hz, 1H), 4.59 (d, *J* = 7.8 Hz, 1H), 4.50 (d, *J* = 8.0 Hz, 1H), 4.44 (d, *J* = 7.9 Hz, 1H), 4.18 (d, *J* = 3.0 Hz, 1H), 4.07 (dd, *J* = 10.9, 4.8 Hz, 1H), 3.99 (dd, *J* = 12.3, 2.0 Hz, 1H), 3.97 – 3.89 (m, 4H), 3.89 – 3.83 (m, 2H), 3.83 – 3.73 (m, 9H), 3.73 – 3.62 (m, 10H), 3.62 – 3.57 (m, 3H), 3.52 (dd, *J* = 9.9, 7.8 Hz, 1H), 3.31 (td, *J* = 8.0, 1.6 Hz, 1H), 3.02 (t, *J* = 7.6 Hz, 2H), 2.80 (dd, *J* = 12.5, 4.8 Hz, 1H), 2.04 (s, 3H), 2.04 (s, 3H), 1.74 – 1.65 (m, 5H), 1.50 – 1.44 (m, 2H).

^{13}C NMR (151 MHz, D_2O)**:** δ 175.0, 174.8, 173.4, 102.9, 102.8, 102.3, 102.0, 100.1, 82.3, 78.5, 77.0, 75.4, 75.0, 74.8, 74.4, 73.0, 72.8, 72.4, 72.2, 71.7, 71.2, 70.0, 69.9, 68.7, 68.4, 68.3, 68.1, 62.6, 62.1, 61.1, 61.1, 60.1, 55.3, 51.9, 40.3, 39.3, 28.1, 26.4, 22.2, 22.1, 22.0.

HRMS (QToF)**:** Calcd for $C_{42}H_{72}N_3O_{29}$ [M - H]$^-$ 1082.4251; found 1082.4391.

Crude analytical RP-HPLC (t_R = 34.6 min)

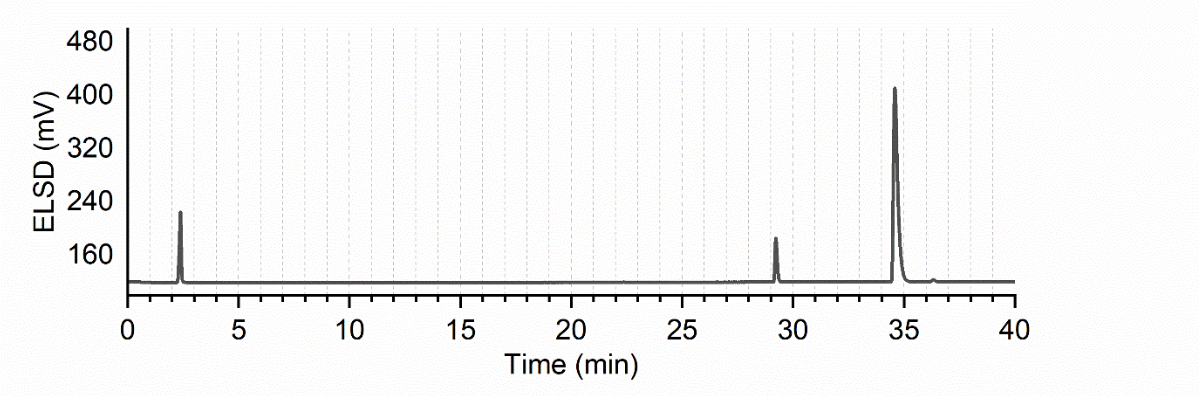

Purified analytical RP-HPLC

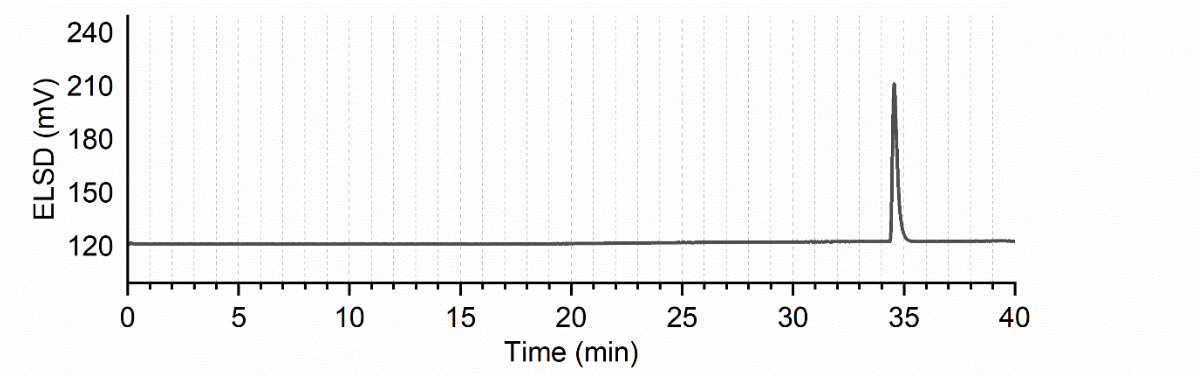

^{1}H-NMR (600 MHz, D_2O)

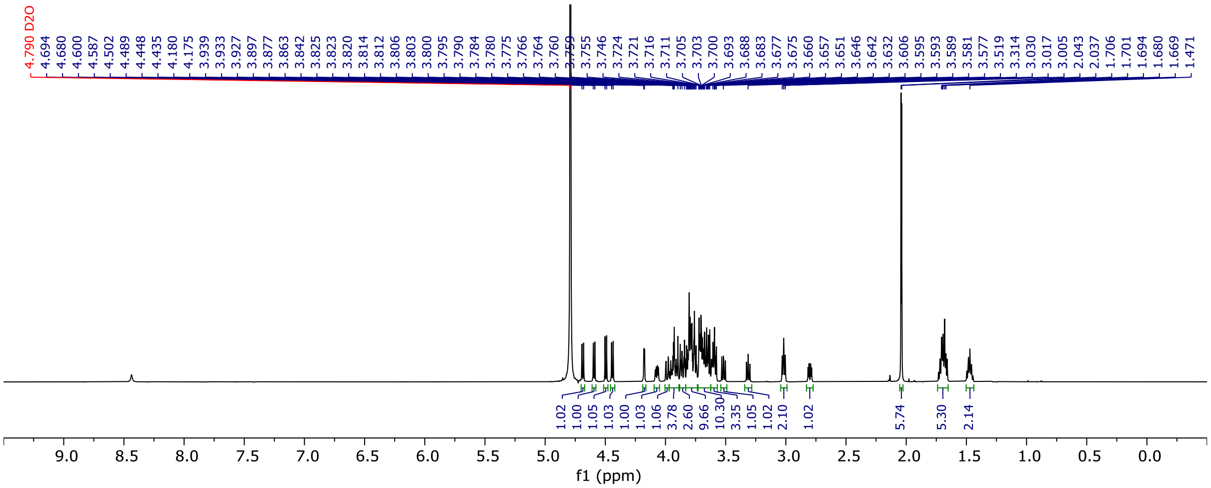

^{13}C-NMR (151 MHz, D_2O)

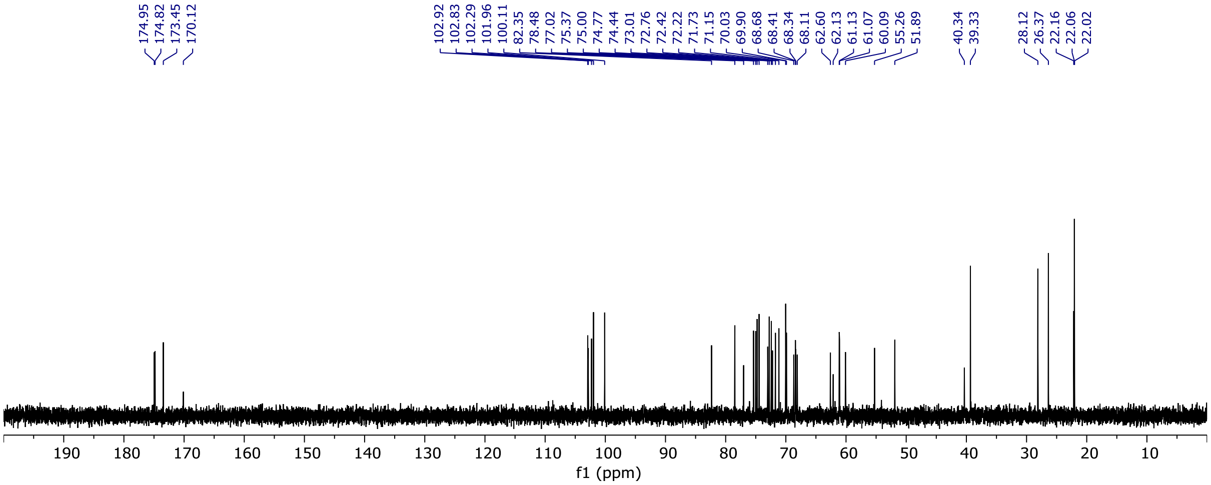

^1H -^1H COSY

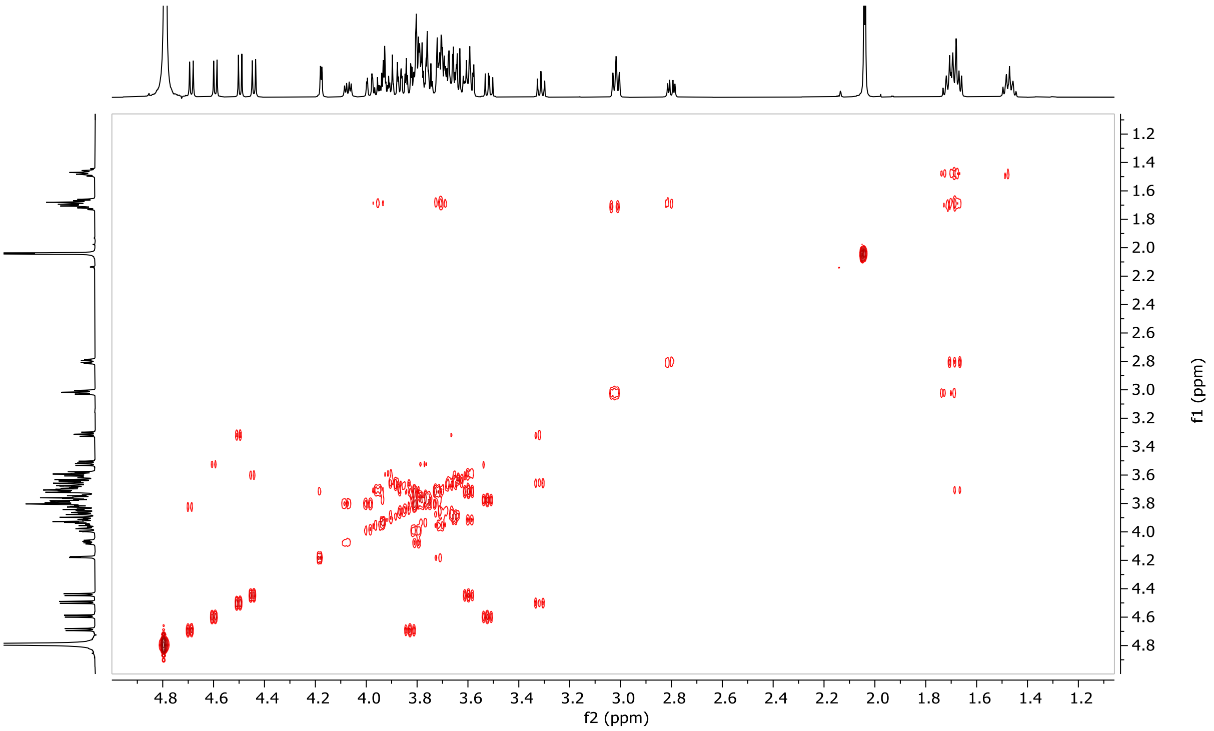

^{13}C-^1H HSQC

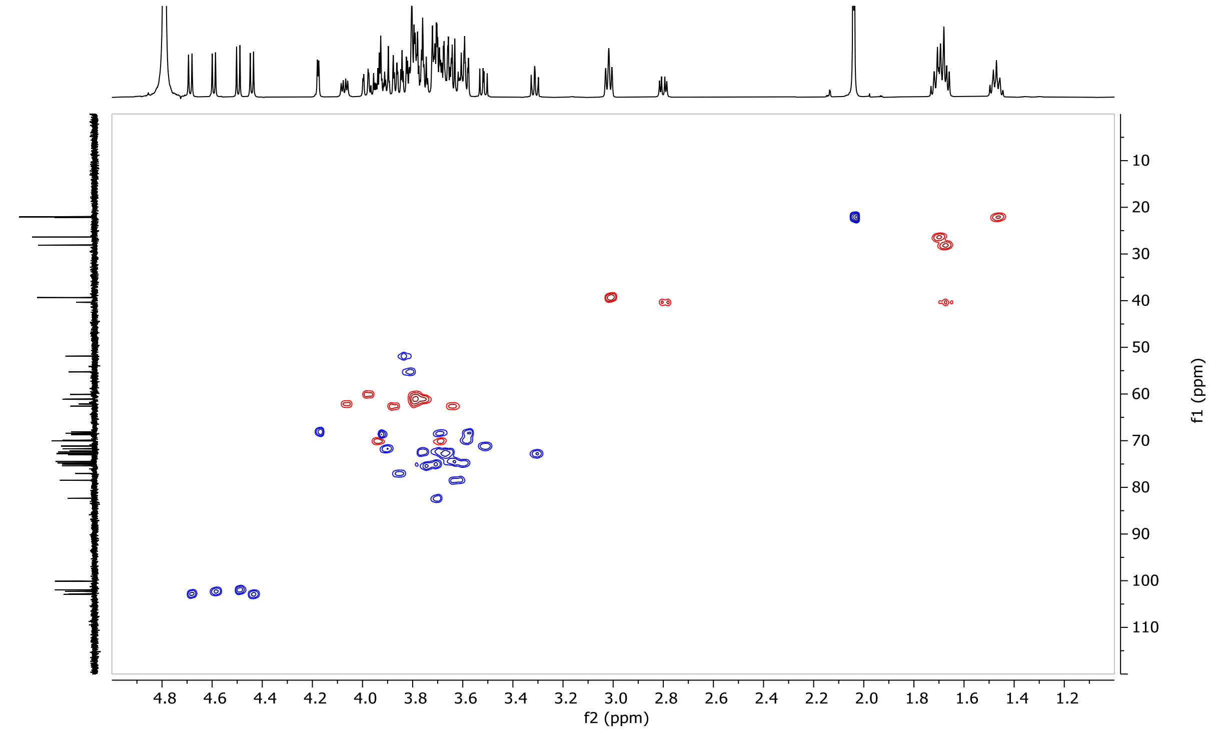

^{13}C-^{1}H HMBC

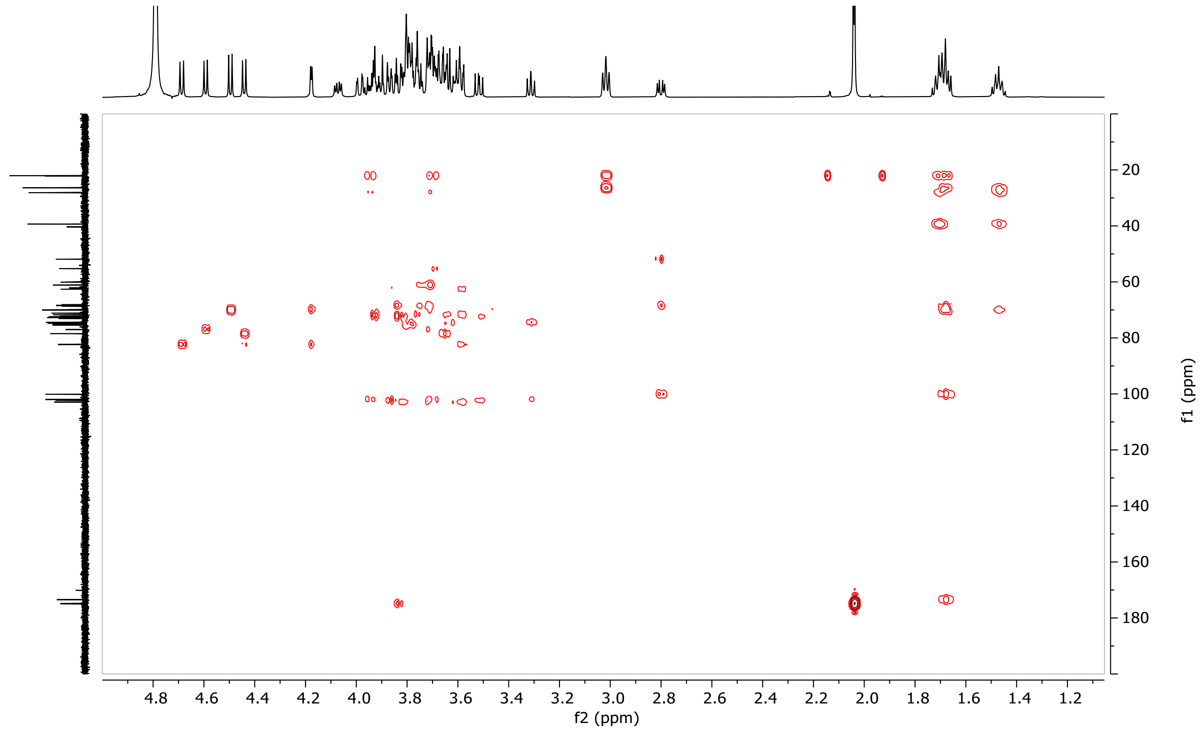

Expansion of ^{13}C-^{1}H HMBC

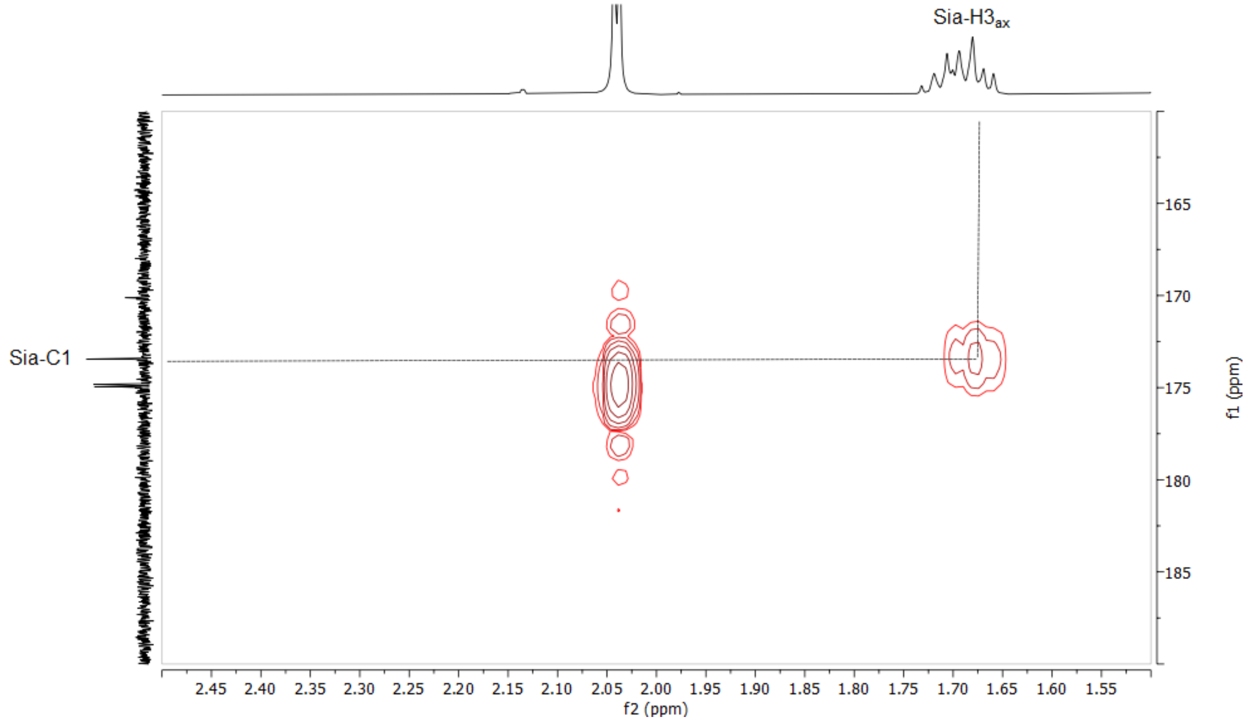

6.4 Sialyllacto-*N*-neotetraose **22**

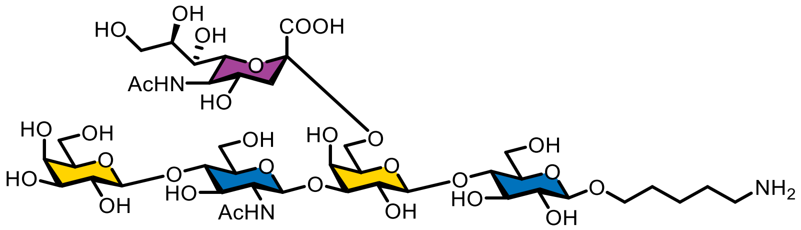

22

Unprotected pentasaccharide **22** (2.9 mg, 2.65 µmol, 56%) was obtained as a colorless oil after global deprotection (**Method C**), followed by purification using preparative RP-HPLC (**Method R1**).

^{1}H NMR (600 MHz, D_2O)**:** δ 4.69 (d, *J* = 8.4 Hz, 1H), 4.50 (d, *J* = 8.0 Hz, 1H), 4.48 (d, *J* = 7.8 Hz, 1H), 4.42 (d, *J* = 7.9 Hz, 1H), 4.19 (d, *J* = 3.3 Hz, 1H), 4.00 – 3.93 (m, 5H), 3.92 – 3.87 (m, 2H), 3.87 – 3.82 (m, 2H), 3.82 – 3.75 (m, 5H), 3.75 – 3.69 (m, 6H), 3.69 – 3.63 (m, 4H), 3.63 – 3.52 (m, 7H), 3.33 (dd, *J* = 9.3, 8.1 Hz, 1H), 3.02 (t, *J* = 7.5 Hz, 2H), 2.71 (dd, *J* = 12.4, 4.7 Hz, 1H), 2.04 (s, 6H), 1.77 – 1.66 (m, 5H), 1.51 – 1.45 (m, 2H).

^{13}C NMR (151 MHz, D_2O)**:** δ 174.9, 174.8, 173.5, 103.2, 102.9, 102.8, 101.8, 100.3, 82.2, 79.7, 78.2, 75.3, 74.7, 74.6, 74.5, 73.3, 72.7, 72.5, 72.5, 72.2, 71.7, 70.9, 70.0, 69.6, 68.5, 68.4, 68.1, 63.5, 62.6, 61.0, 60.3, 59.9, 55.2, 51.8, 40.1, 39.3, 28.1, 26.4, 22.2, 22.1, 22.0.

HRMS (QToF)**:** Calcd for $C_{42}H_{72}N_3O_{29}$ [M - H]$^-$ 1082.4251; found 1082.4218.

Crude analytical RP-HPLC (t_R = 29.6 min)

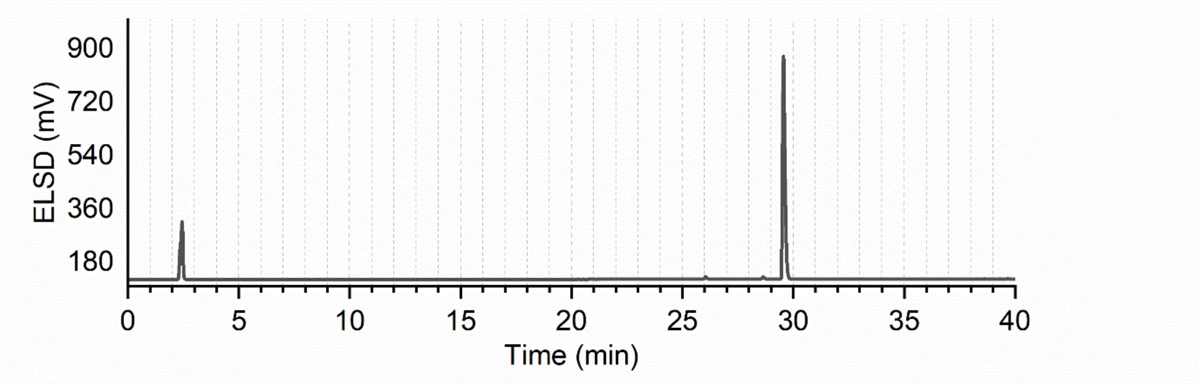

Purified analytical RP-HPLC

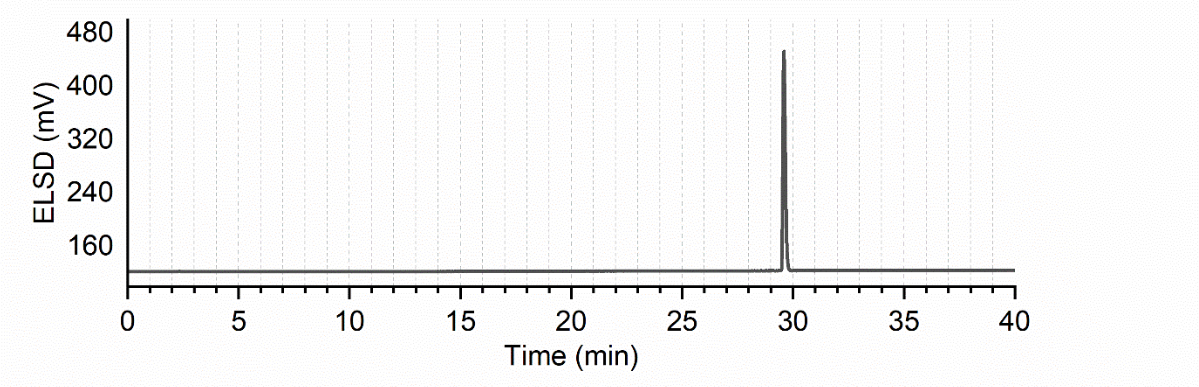

^{1}H-NMR (600 MHz, D_2O)

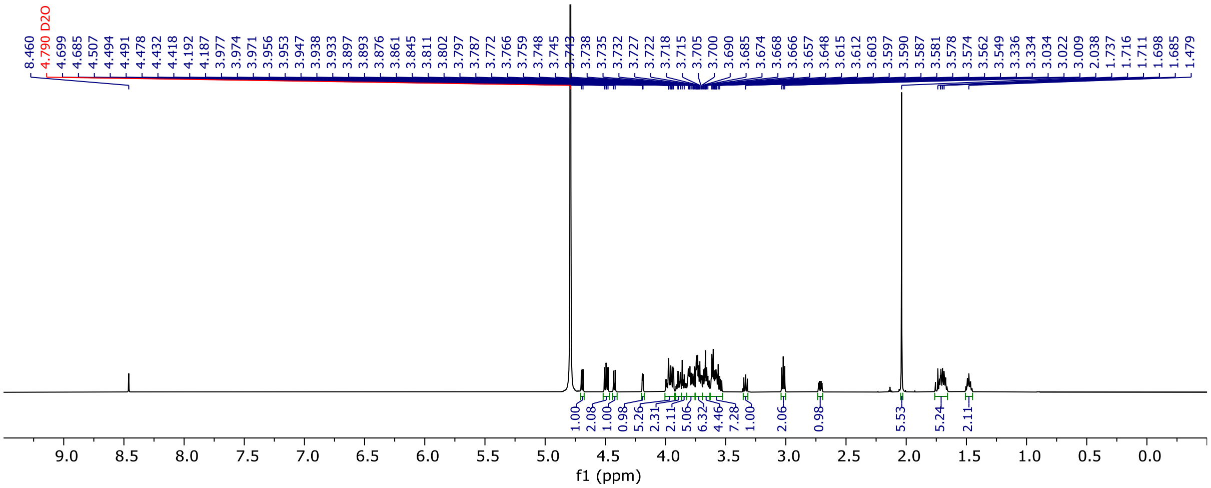

^{13}C-NMR (151 MHz, D_2O)

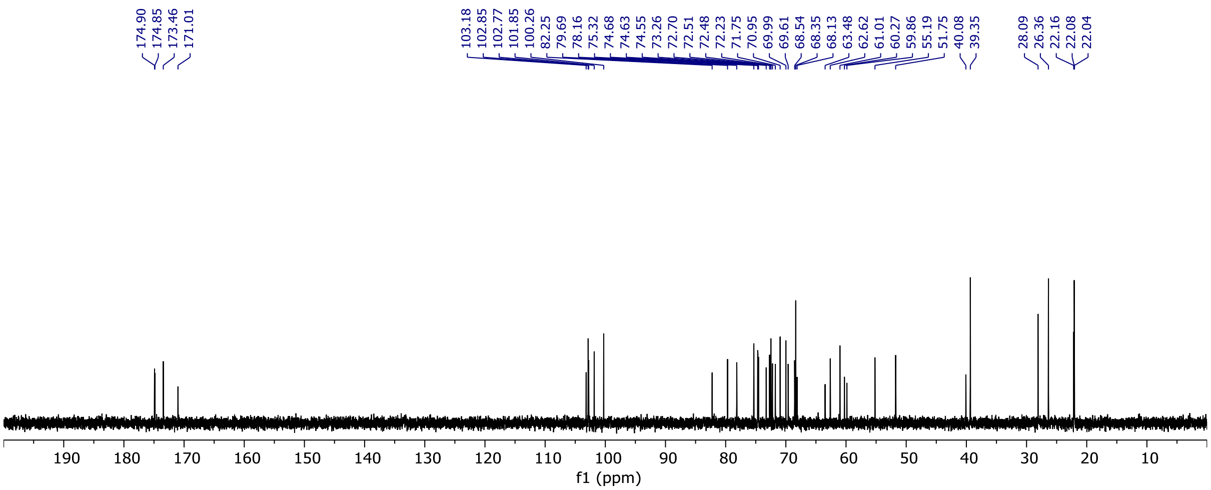

^{1}H -^{1}H COSY

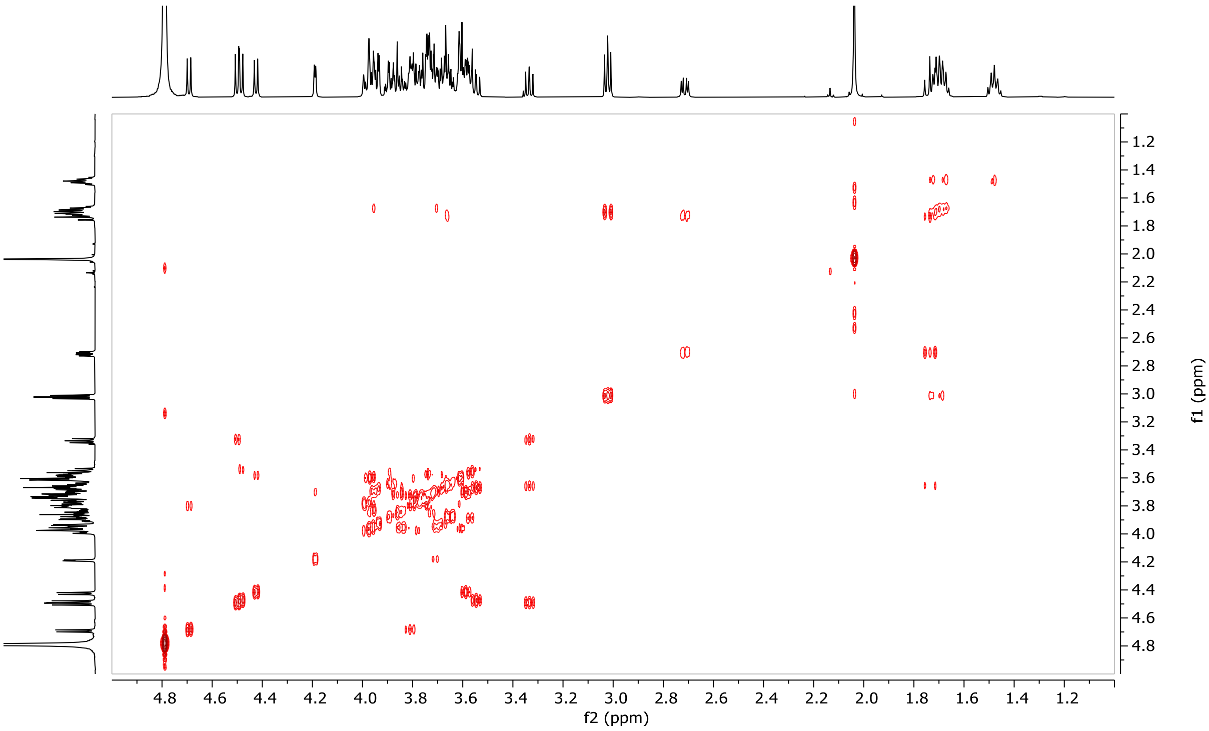

^{13}C-^{1}H HSQC

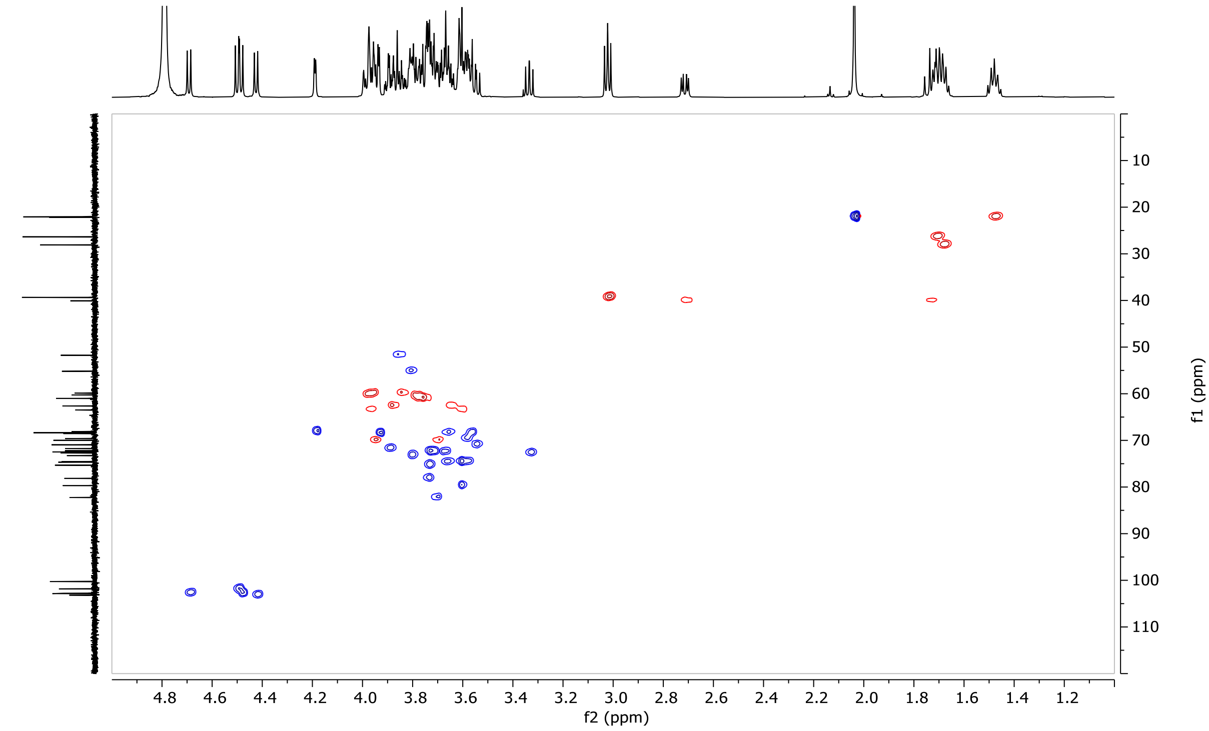

^{13}C-^{1}H HMBC

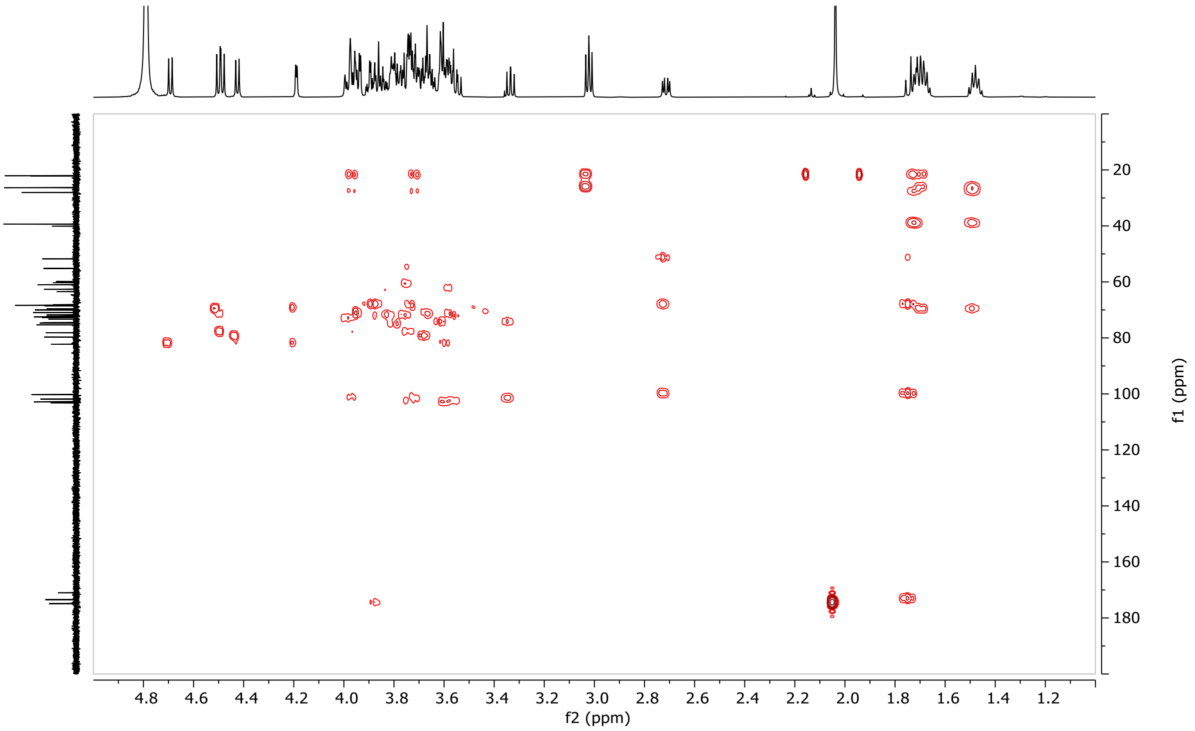

Expansion of ^{13}C-^{1}H HMBC

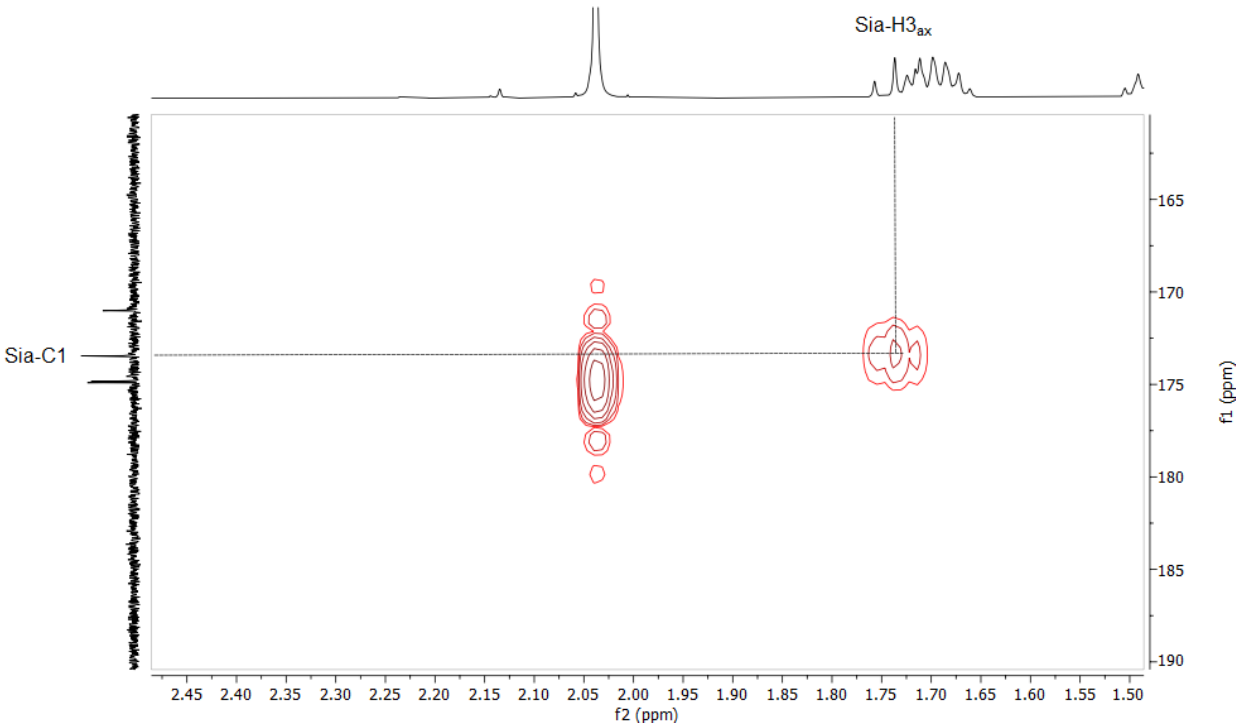

6.5 <u>Disaccharide **23**</u>

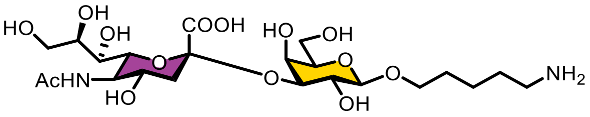

23

Unprotected disaccharide **23** (1.0 mg, 1.74 µmol, 31%) was obtained as a colorless oil after global deprotection (**Method C**), followed by purification using preparative RP-HPLC (**Method R2**).

^{1}H NMR (600 MHz, D_2O)**:** δ 4.48 (d, J = 8.0 Hz, 1H), 4.09 (dd, J = 9.8, 3.2 Hz, 1H), 3.98 – 3.92 (m, 2H), 3.91 – 3.82 (m, 3H), 3.79 – 3.73 (m, 2H), 3.73 – 3.66 (m, 3H), 3.66 – 3.59 (m, 3H), 3.55 (dd, J = 9.8, 8.0 Hz, 1H), 3.02 (t, J = 7.5 Hz, 2H), 2.77 (dd, J = 12.4, 4.6 Hz, 1H), 2.04 (s, 3H), 1.81 (t, J = 12.1 Hz, 1H), 1.75 – 1.63 (m, 4H), 1.51 – 1.43 (m, 2H).

^{13}C NMR (151 MHz, D_2O)**:** δ 175.1, 173.3, 102.9, 100.5, 73.4, 72.7, 72.6, 71.8, 70.7, 70.3, 68.6, 68.2, 68.2, 63.4, 62.6, 51.8, 40.2, 39.4, 28.2, 26.4, 22.0.

HRMS (QToF)**:** Calcd for $C_{22}H_{39}N_2O_{14}$ [M - H]$^-$ 555.2401; found 555.2401

Crude analytical RP-HPLC (t_R = 24.7 min)

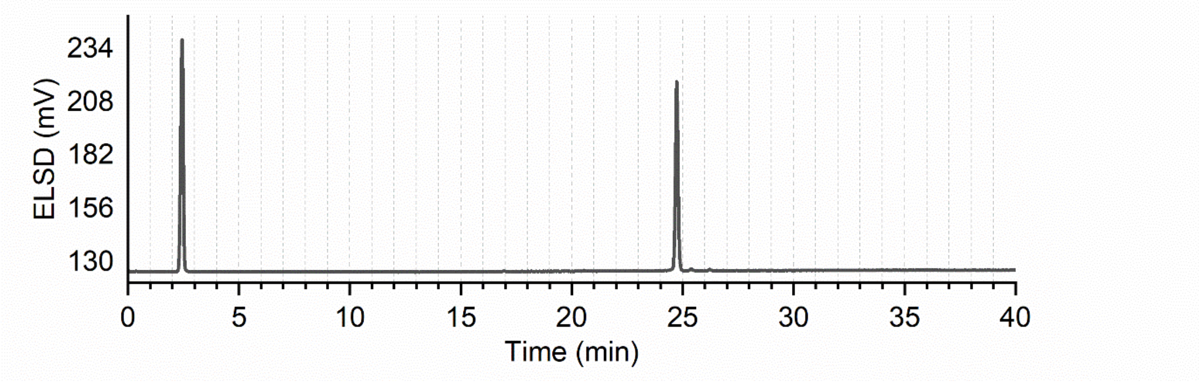

Purified analytical RP-HPLC

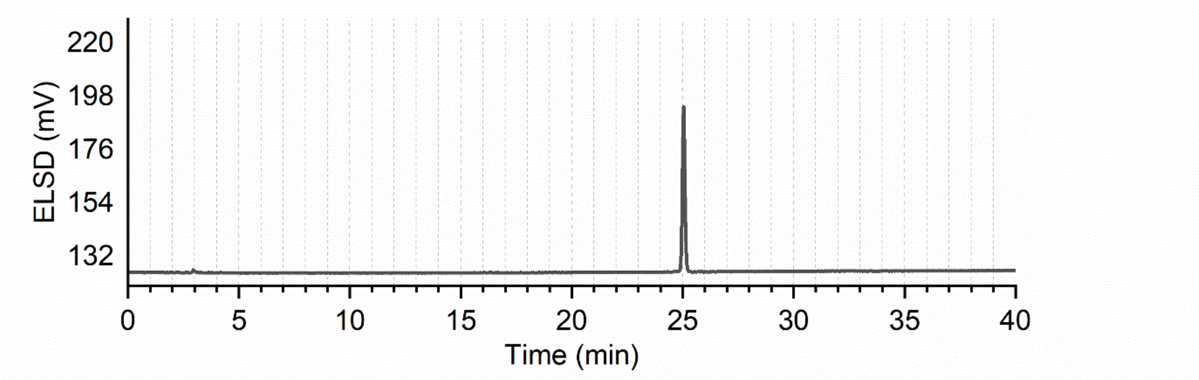

^{1}H-NMR (600 MHz, D_2O)

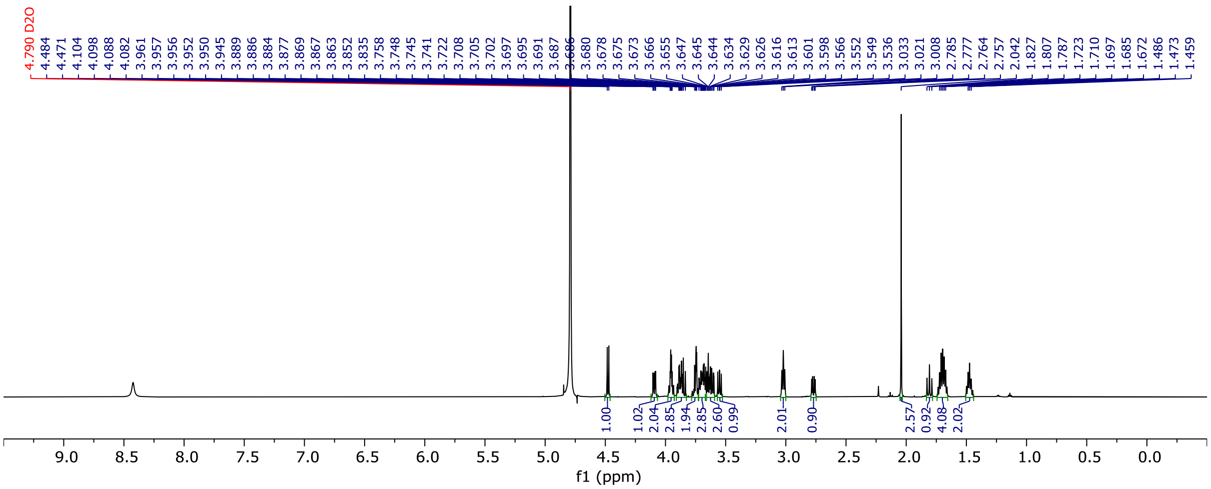

^{13}C-NMR (151 MHz, D_2O)

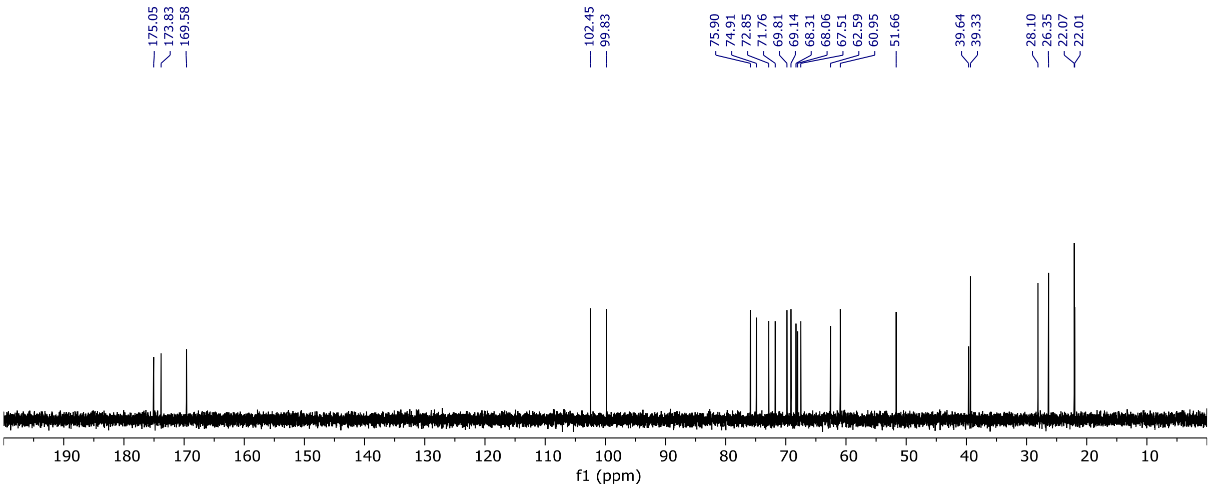

^{1}H -^{1}H COSY

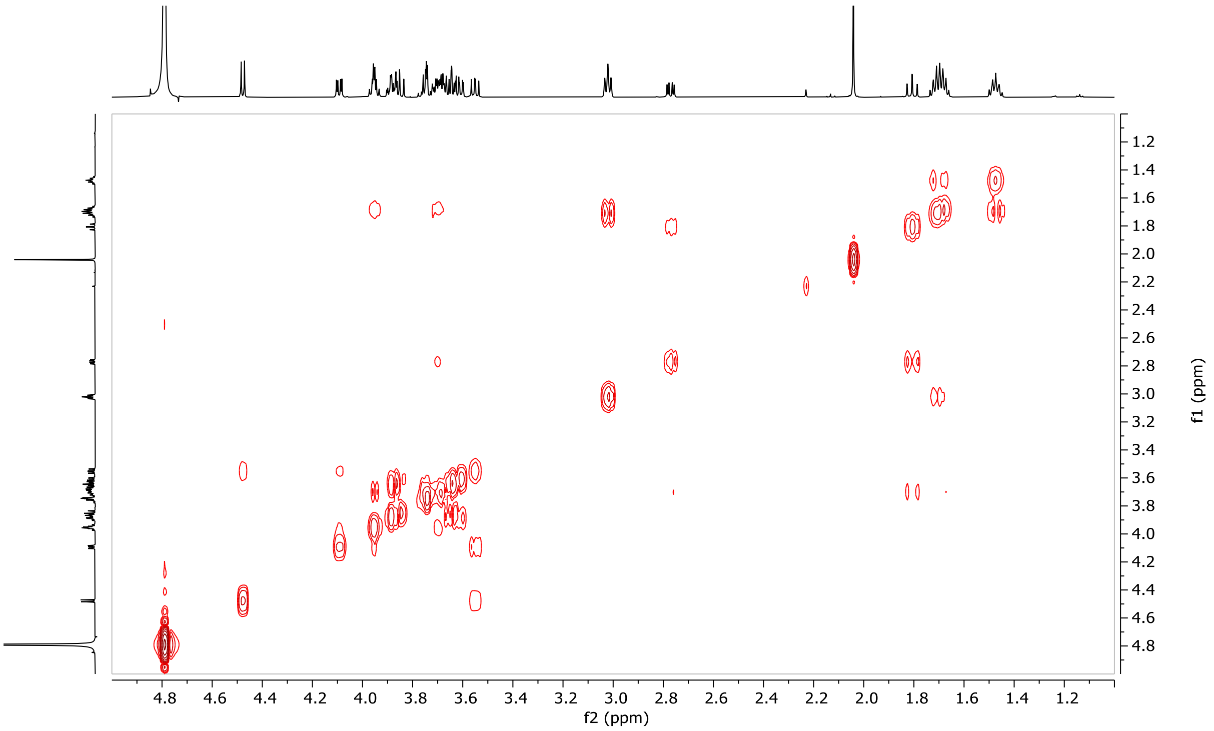

^{13}C-^{1}H HSQC

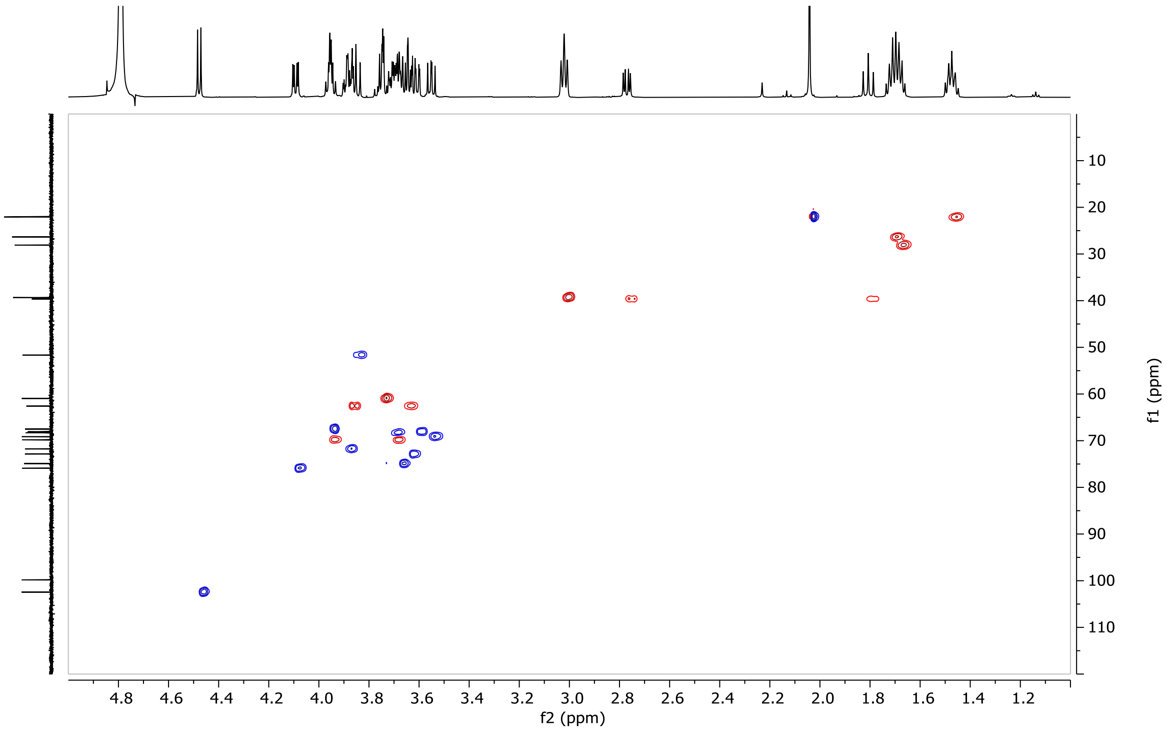

^{13}C-^{1}H HMBC

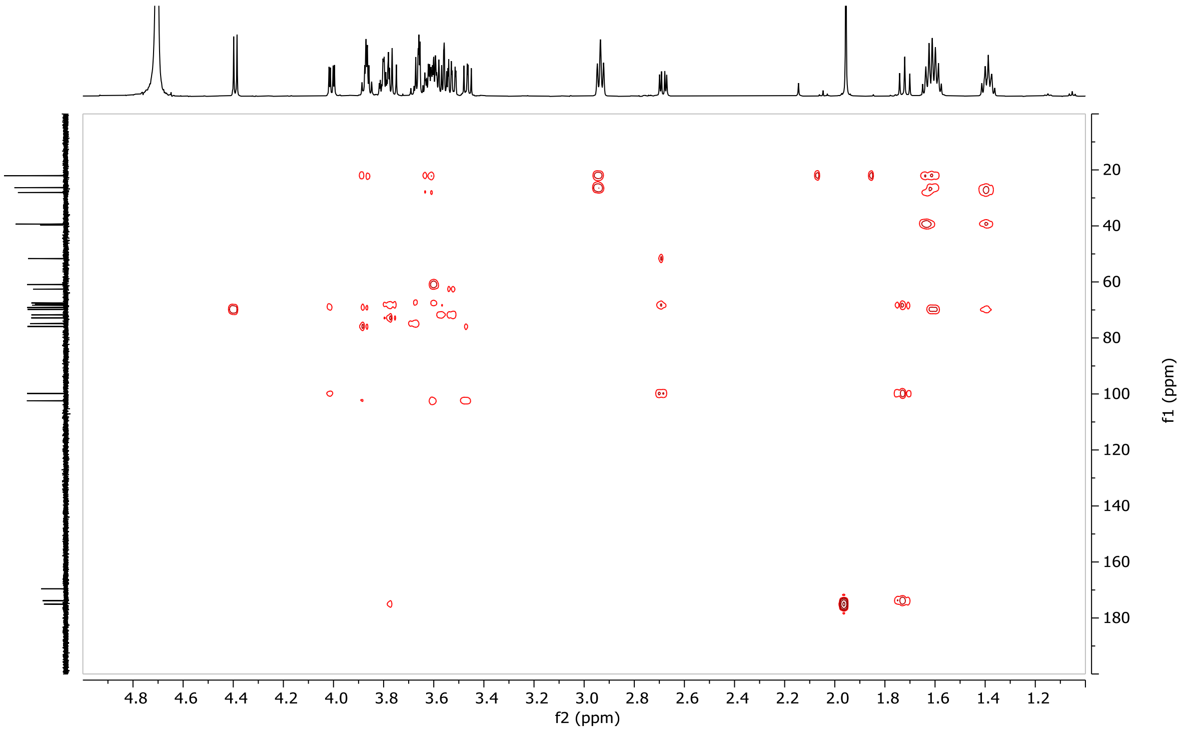

Expansion of ^{13}C-^{1}H HMBC

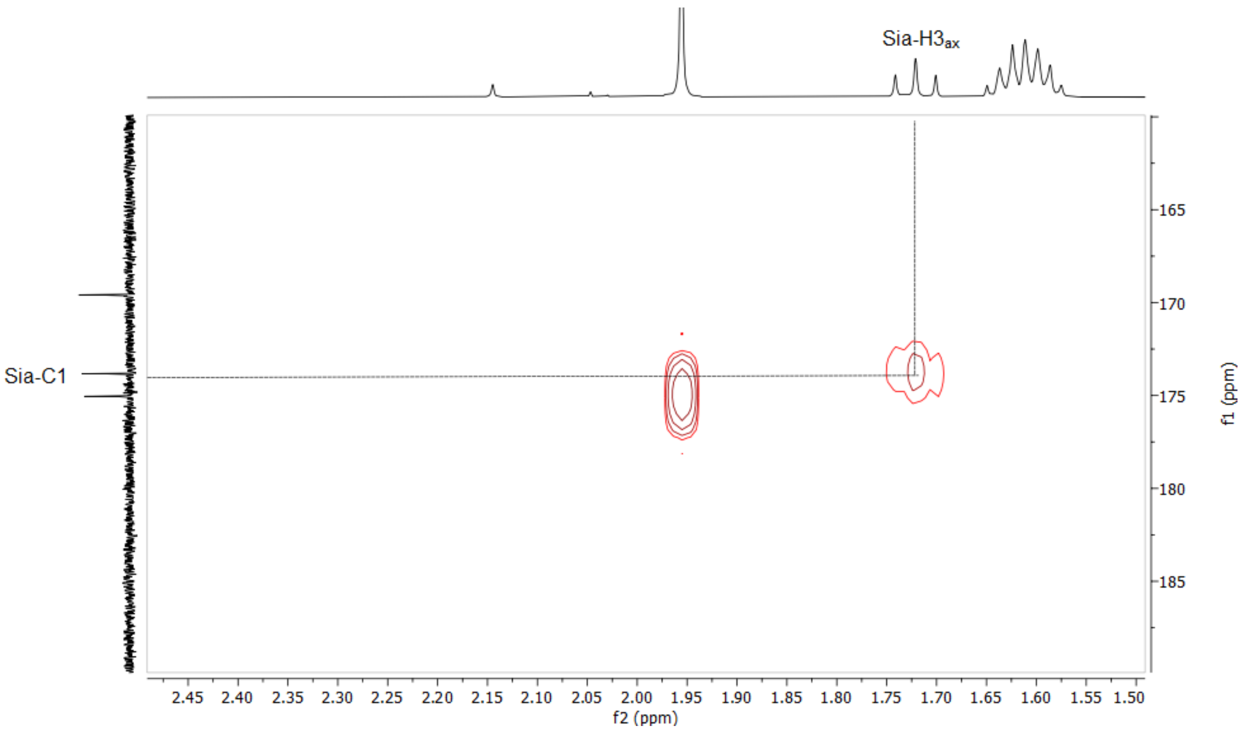

6.6 Sialyllacto-N-neotetraose **24**

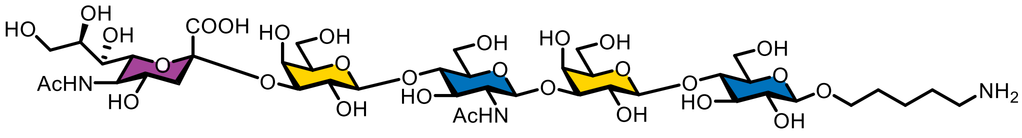

24

Unprotected pentasaccharide **24** (1.7 mg, 1.52 µmol, 40%) was obtained as a colorless oil after global deprotection (**Method C**), followed by purification using preparative RP-HPLC (**Method R3**).

^{1}H NMR (600 MHz, D_2O)**:** δ 4.71 (d, J = 8.4 Hz, 1H), 4.57 (d, J = 7.8 Hz, 1H), 4.49 (d, J = 8.0 Hz, 1H), 4.44 (d, J = 7.9 Hz, 1H), 4.16 (d, J = 3.3 Hz, 1H), 4.12 (dd, J = 9.9, 3.2 Hz, 1H), 4.00 – 3.92 (m, 4H), 3.92 – 3.82 (m, 4H), 3.82 – 3.67 (m, 13H), 3.67 – 3.63 (m, 4H), 3.63 – 3.56 (m, 5H), 3.31 (d, J = 8.3 Hz, 1H), 3.01 (t, J = 7.6 Hz, 2H), 2.77 (dd, J = 12.4, 4.6 Hz, 1H), 2.04 (s, 6H), 1.81 (t, J = 12.1 Hz, 1H), 1.75 – 1.64 (m, 4H), 1.51 – 1.43 (m, 2H).

^{13}C NMR (151 MHz, D_2O)**:** δ 175.0, 174.9, 173.8, 169.1, 102.9, 102.8, 102.5, 102.0, 99.8, 82.0, 78.4, 78.0, 75.5, 75.2, 74.9, 74.8, 74.6, 74.5, 74.4, 72.9, 72.8, 72.1, 71.7, 70.1, 69.9, 69.4, 68.3, 68.3, 68.1, 67.5, 62.6, 61.0, 61.0, 60.1, 59.8, 59.8, 55.2, 51.7, 39.6, 39.3, 28.1, 26.4, 22.2, 22.1, 22.0.

HRMS (QToF)**:** Calcd for $C_{42}H_{72}N_3O_{29}$ [M - H]$^-$ 1082.4251; found 1082.4242.

Crude analytical RP-HPLC (t_R = 32.9 min)

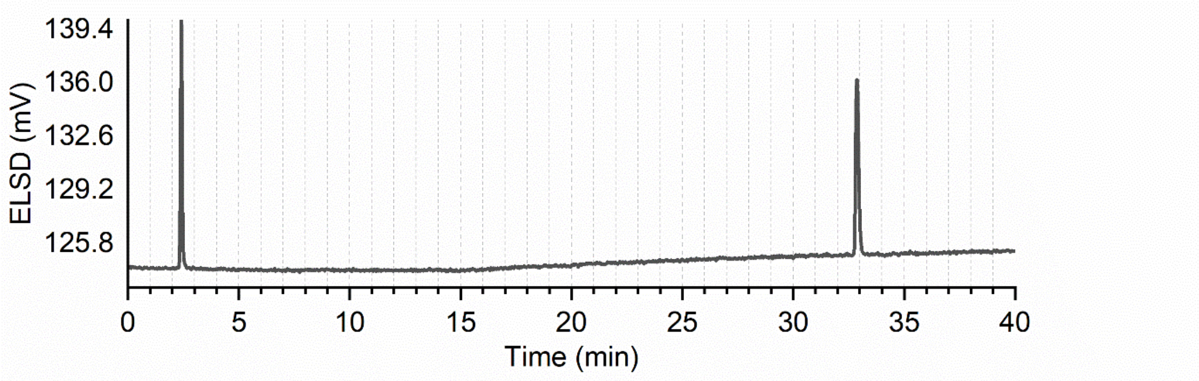

Purified analytical RP-HPLC

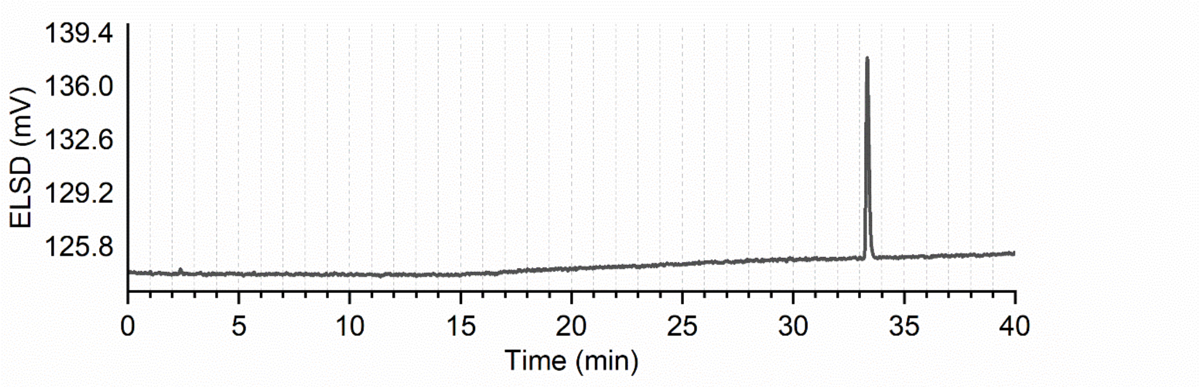

^{1}H-NMR (600 MHz, D_2O)

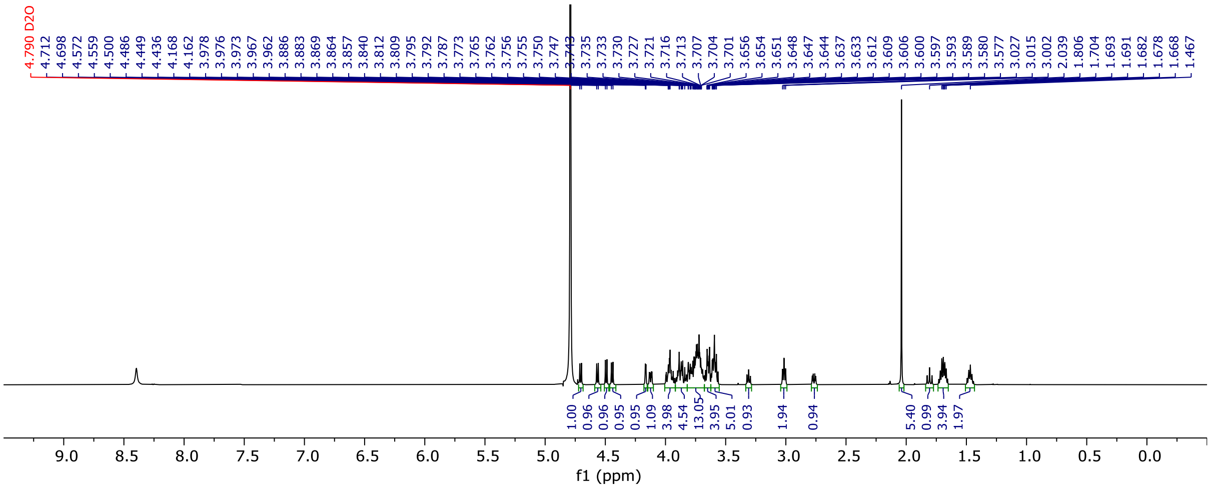

^{13}C-NMR (151 MHz, D_2O)

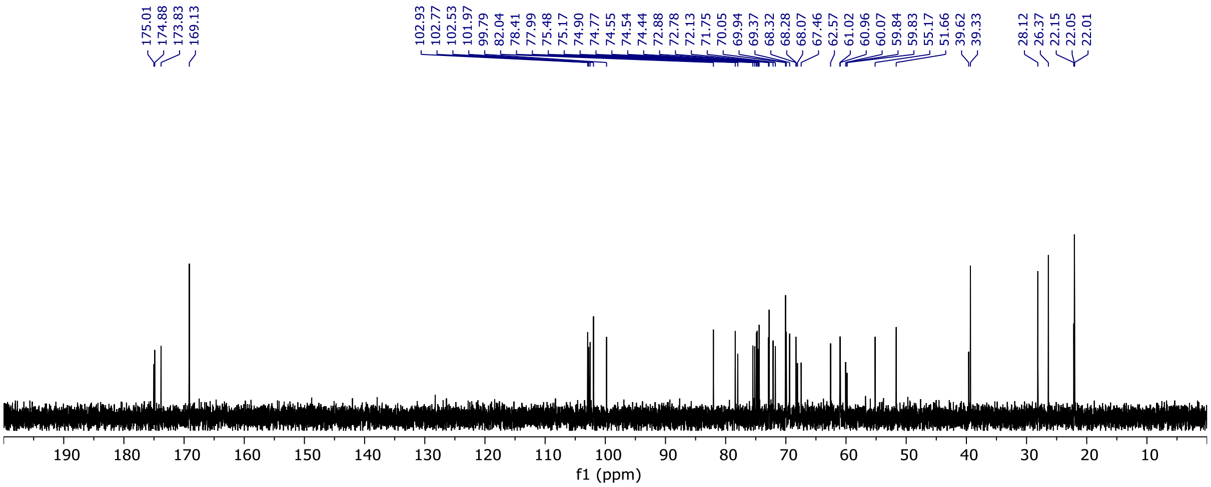

^{1}H -^{1}H COSY

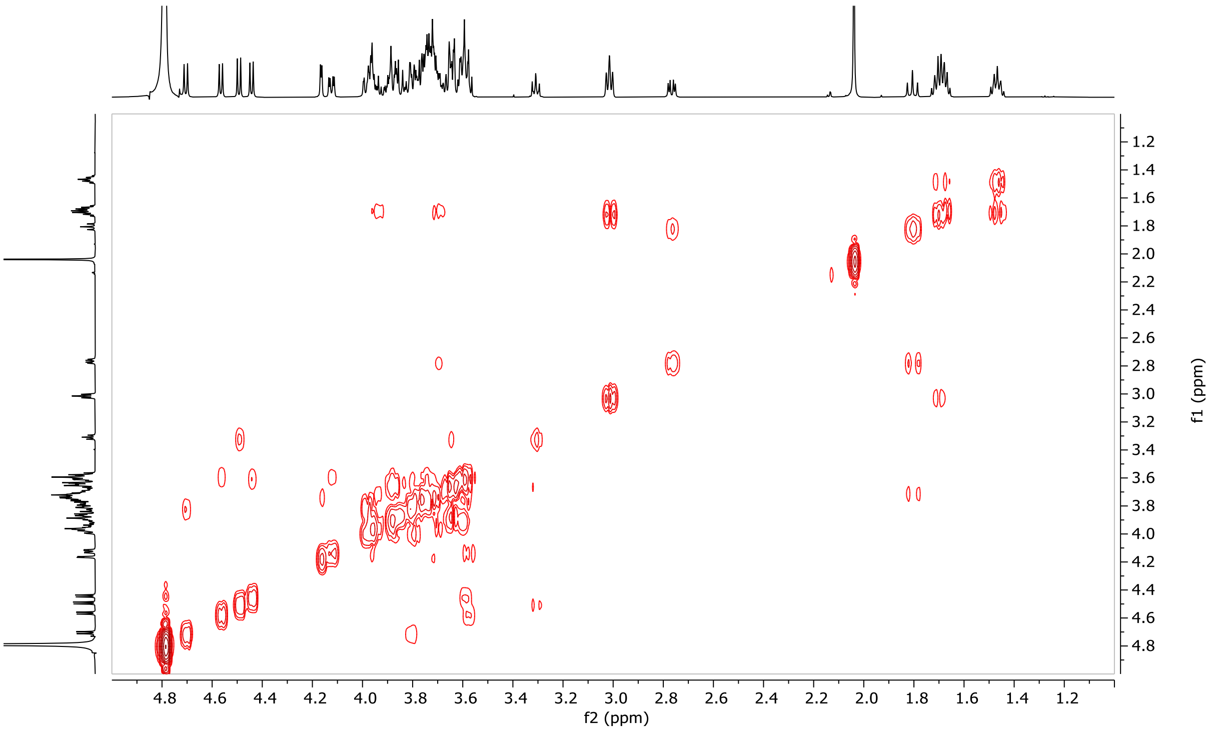

^{13}C-^{1}H HSQC

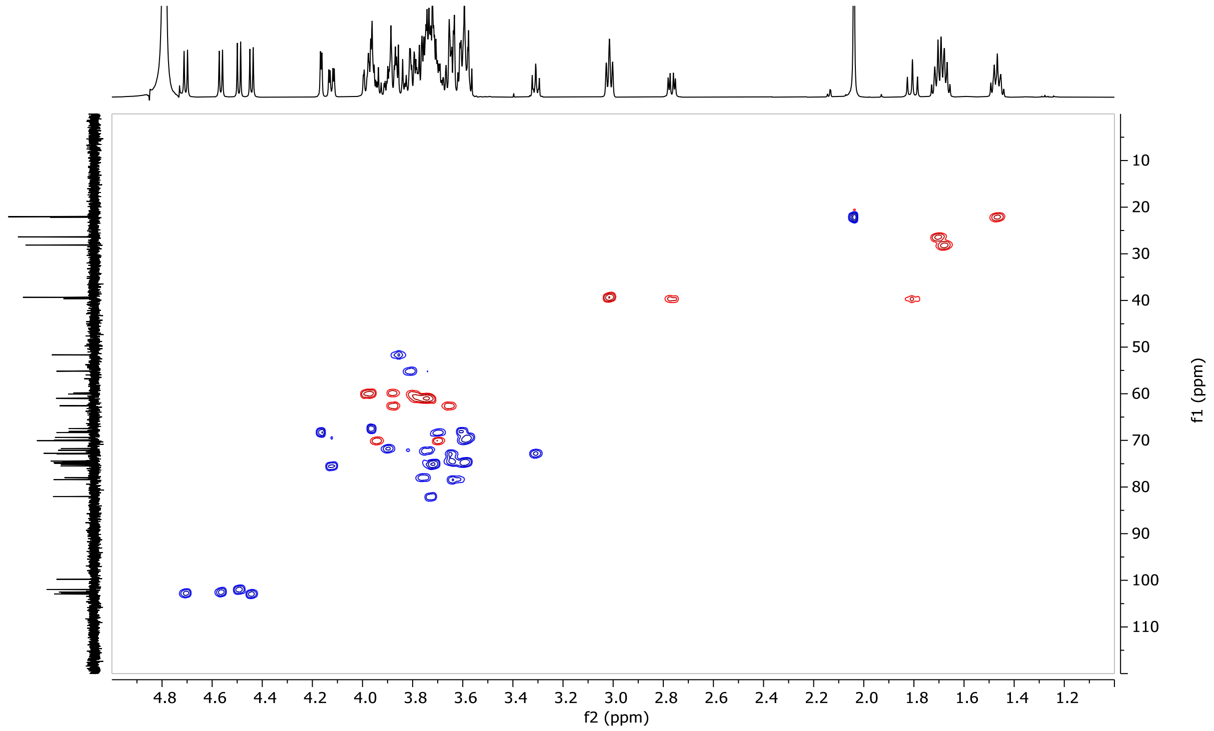

^{13}C-^{1}H HMBC

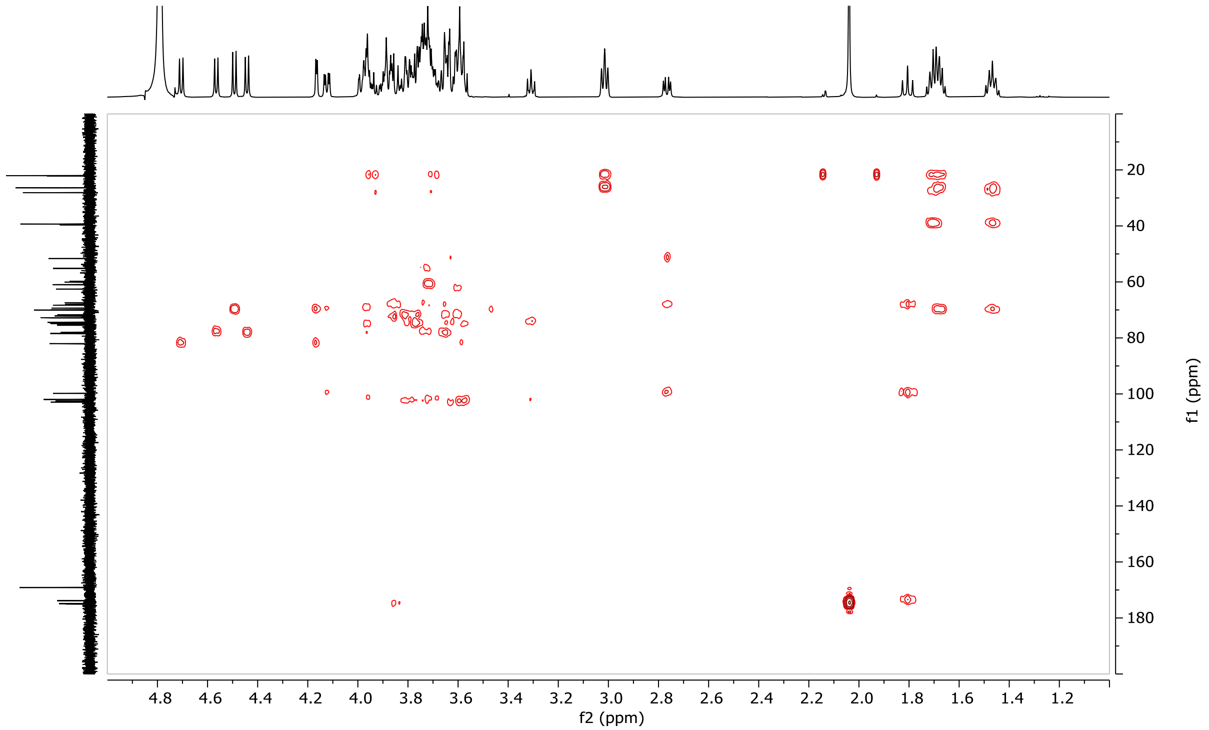

Expansion of ^{13}C-^{1}H HMBC

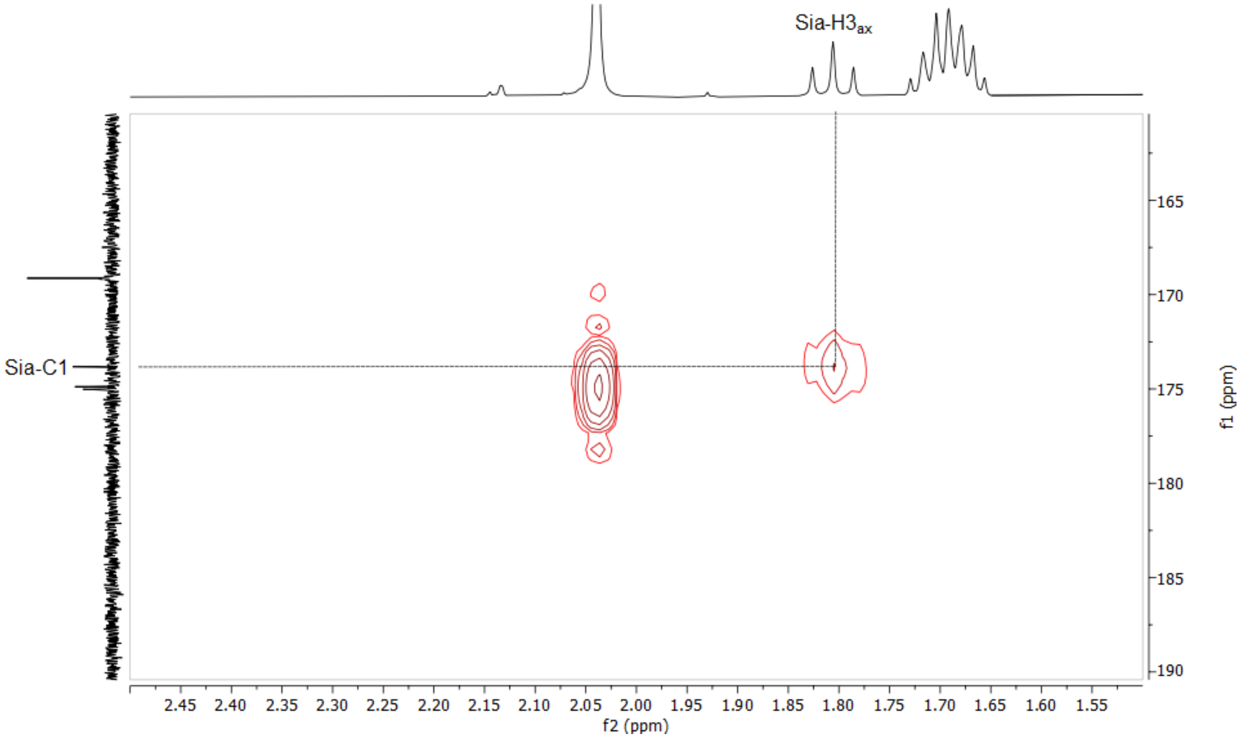

6.7 Disialyllacto-*N*-neotetraose (DSLNnT) **25**

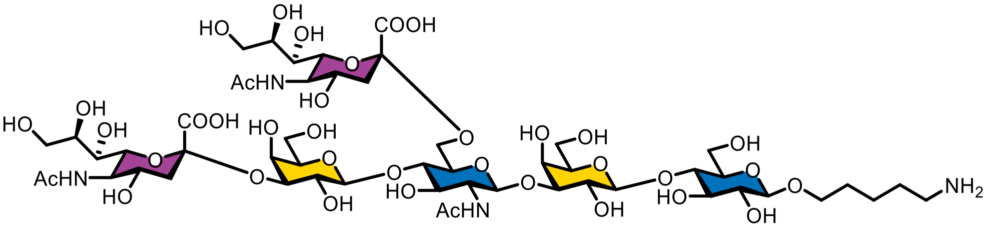

25

Unprotected hexasaccharide **25** (1.1 mg, 0.80 µmol, 28%) was obtained as a colorless oil after global deprotection (**Method C**), followed by purification using preparative RP-HPLC (**Method R4**).

^{1}H NMR (600 MHz, D_2O)**:** δ 4.68 (d, J = 8.4 Hz, 1H), 4.61 (d, J = 7.8 Hz, 1H), 4.50 (d, J = 8.0 Hz, 1H), 4.45 (d, J = 8.0 Hz, 1H), 4.23 – 4.19 (m, 1H), 4.16 (dd, J = 9.8, 3.2 Hz, 1H), 4.01 – 3.97 (m, 2H), 3.97 – 3.89 (m, 7H), 3.88 – 3.76 (m, 8H), 3.75 – 3.63 (m, 14H), 3.63 – 3.53 (m, 6H), 3.31 (d, J = 8.0 Hz, 1H), 3.04 – 2.99 (m, 2H), 2.77 (dd, J = 12.3, 4.7 Hz, 1H), 2.73 (dd, J = 12.2, 4.3 Hz, 1H), 2.04 (s, 3H), 2.04 (s, 3H), 2.03 (s, 3H), 1.86 (d, J = 12.6 Hz, 1H), 1.74 – 1.65 (m, 5H), 1.51 – 1.43 (m, 2H).

^{13}C NMR (151 MHz, D_2O)**:** δ 175.0, 174.9, 174.8, 174.2, 173.5, 171.0, 102.9, 102.7, 102.0, 101.9, 100.3, 100.3, 100.2, 82.4, 78.5, 78.5, 77.8, 75.3, 75.0, 74.9, 74.8, 74.5, 73.0, 72.8, 72.8, 72.5, 72.3, 71.7, 71.7, 71.7, 70.0, 69.9, 69.7, 68.6, 68.4, 68.2, 68.1, 67.6, 62.8, 62.8, 62.7, 62.6, 62.6, 62.6, 62.6, 61.2, 61.1, 60.1, 55.0, 51.9, 51.7, 39.9, 39.3, 39.1, 39.1, 28.1, 26.4, 22.2, 22.1, 22.0.

HRMS (QToF)**:** Calcd for $C_{53}H_{89}N_4O_{37}$ $[M - H]^-$ 1373.5206; found 1373.5194.

Crude analytical RP-HPLC (t_R = 32.7 min)

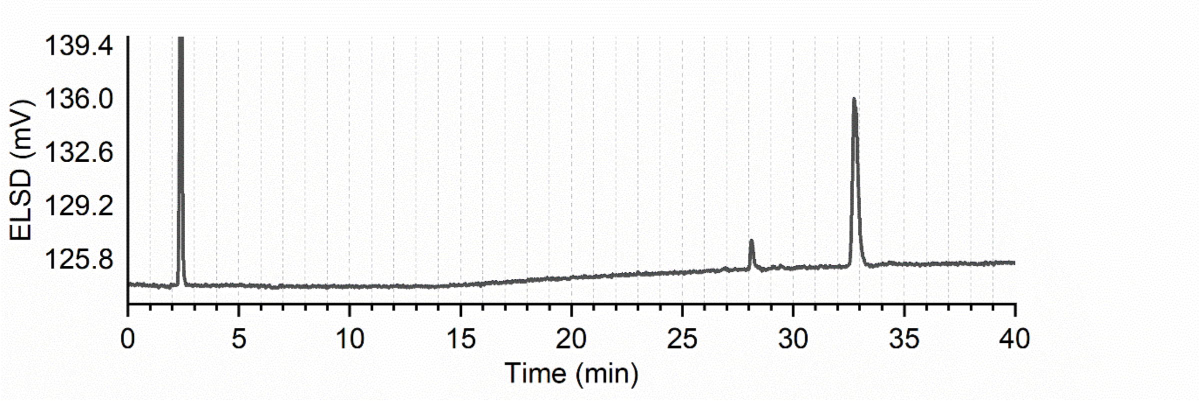

Purified analytical RP-HPLC

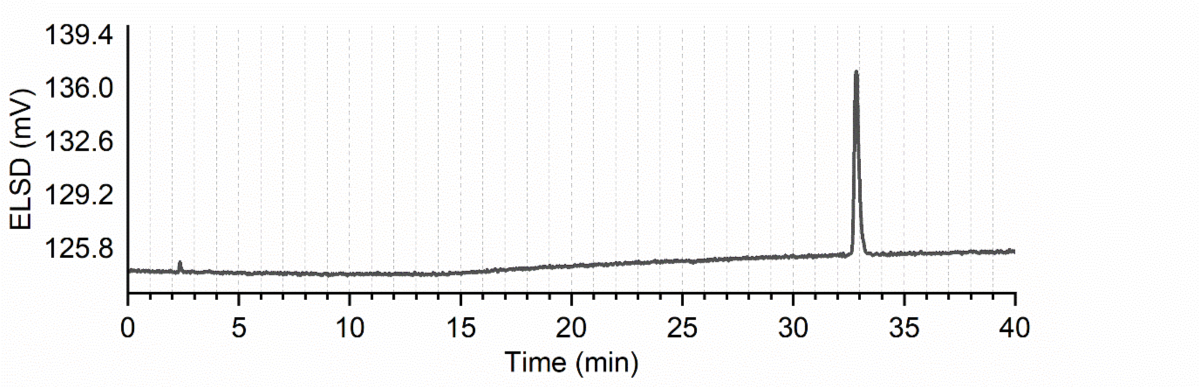

^{1}H-NMR (600 MHz, D_2O)

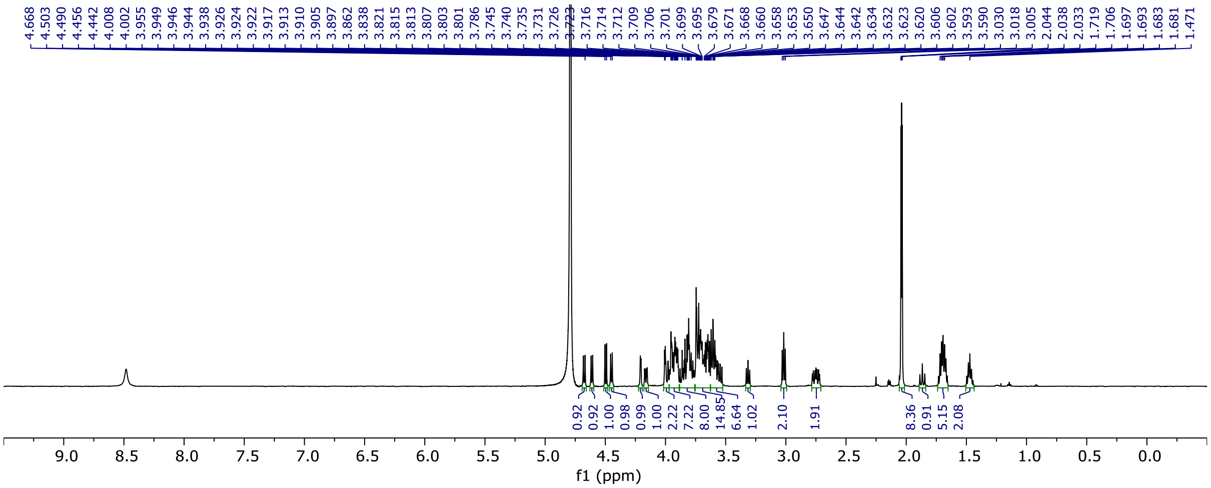

^{13}C-NMR (151 MHz, D_2O)

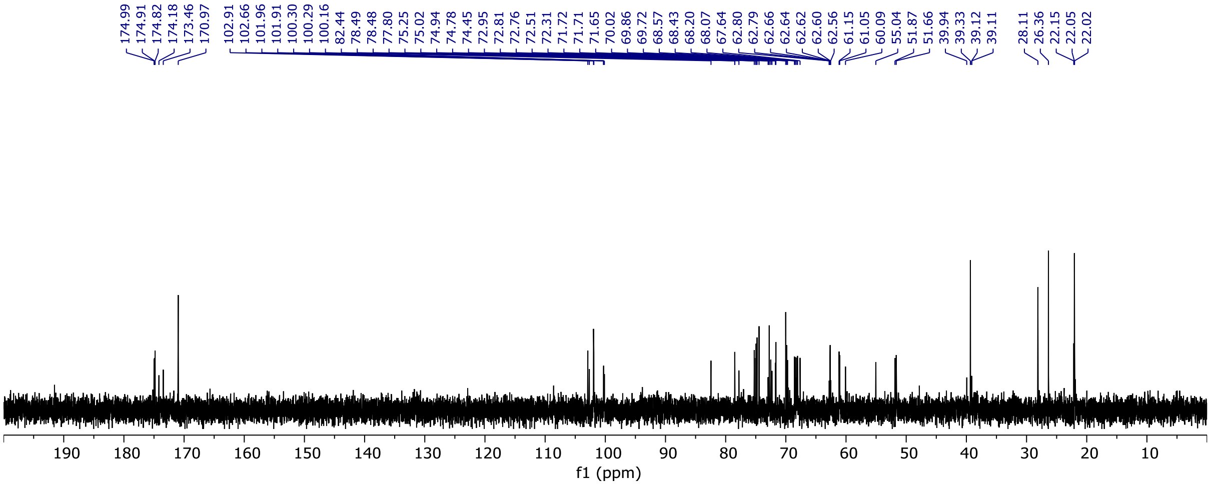

^{1}H -^{1}H COSY

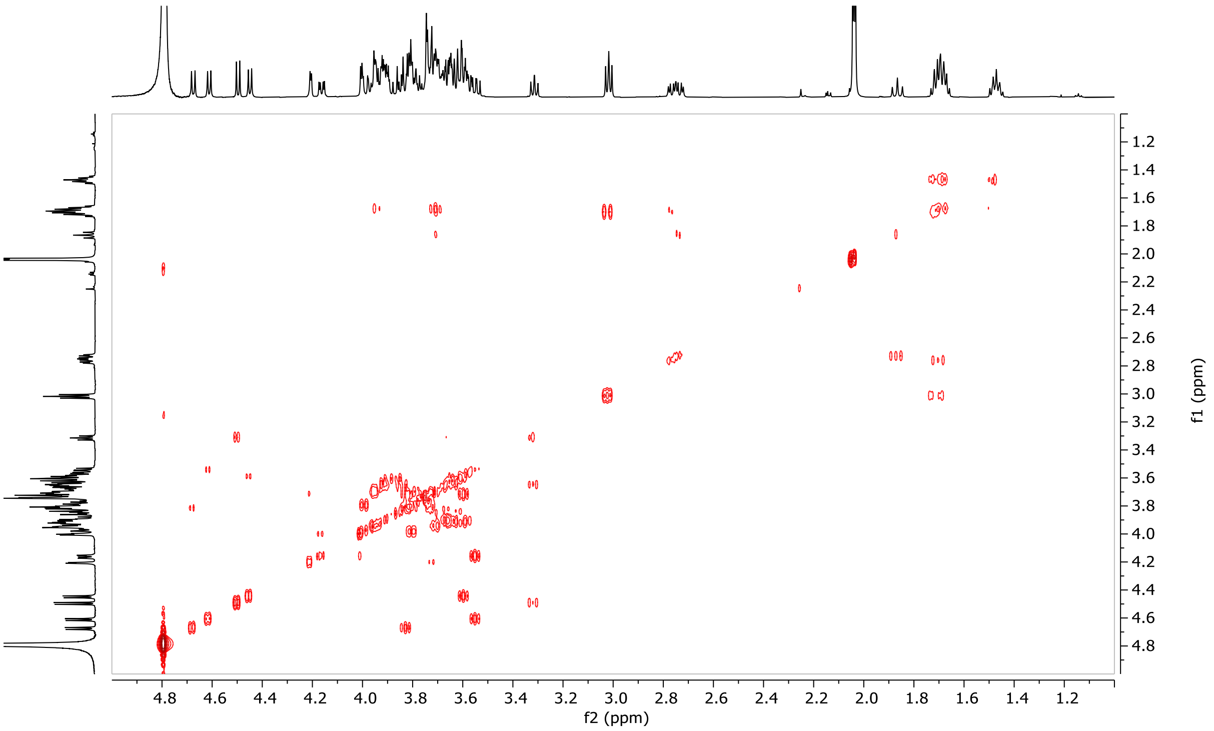

^{13}C-^{1}H HSQC

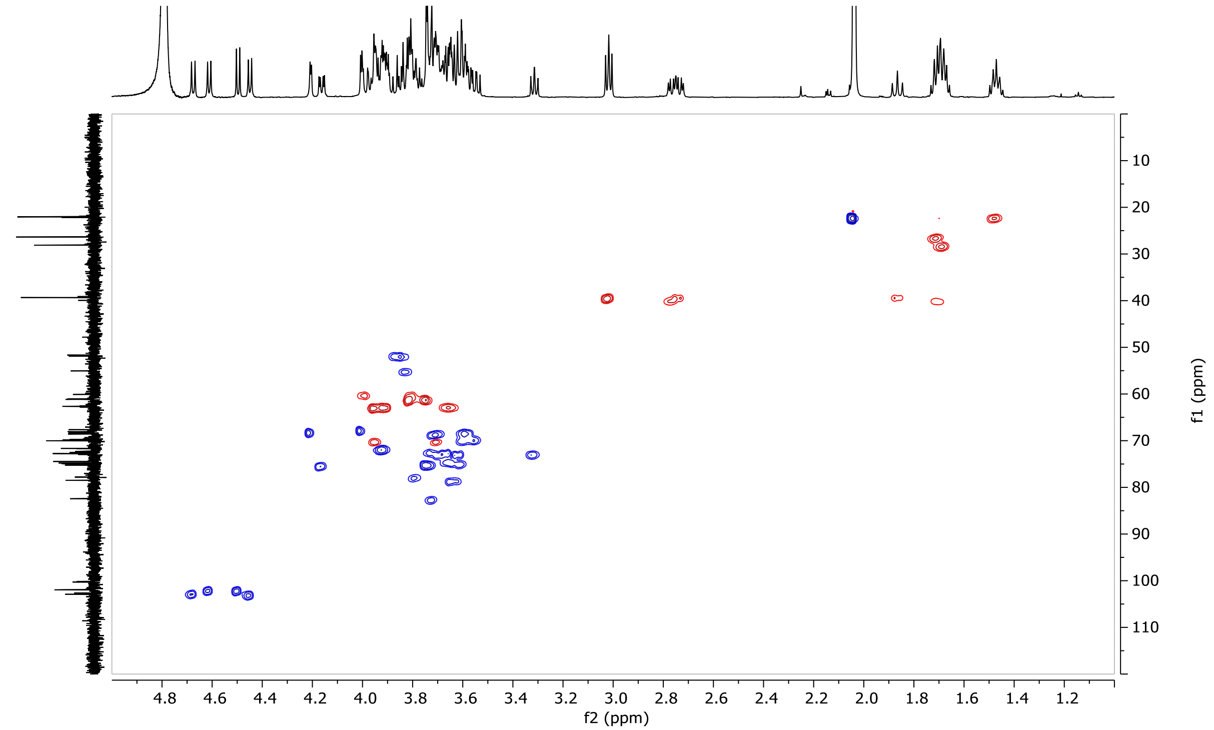

^{13}C-^{1}H HMBC

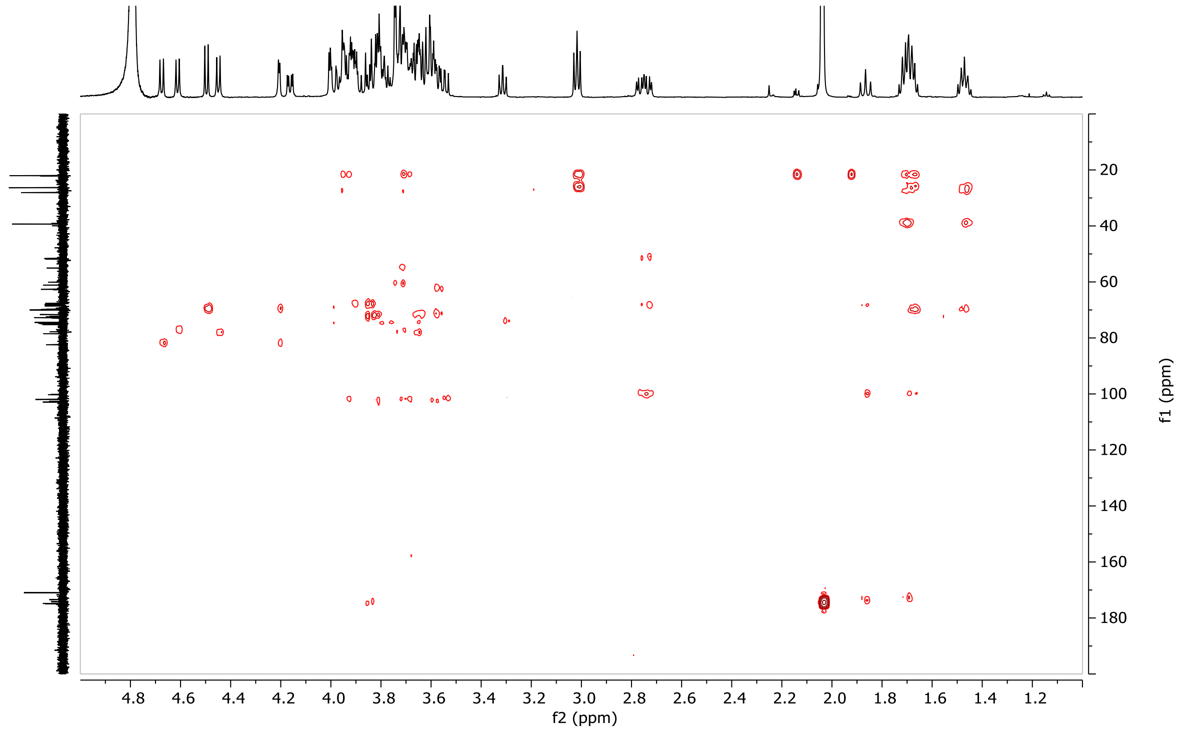

Expansion of ^{13}C-^{1}H HMBC

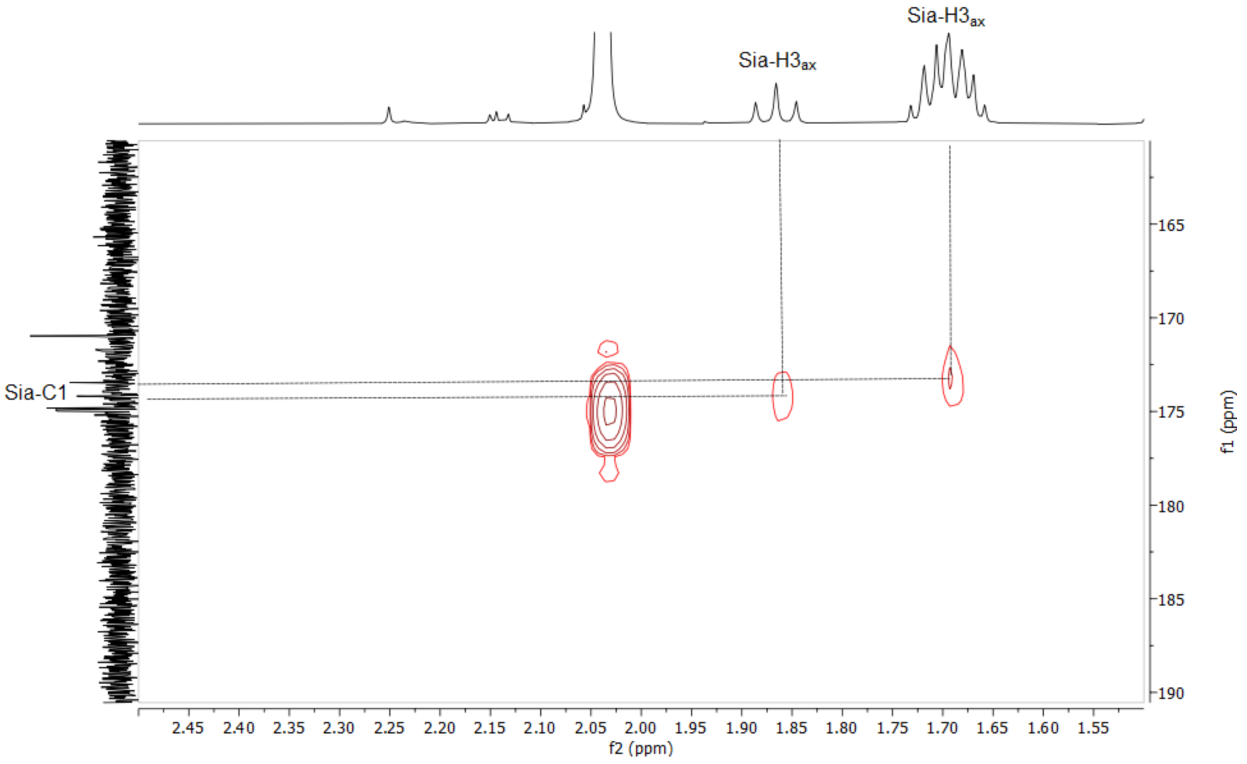

6.8 Disialyllacto-*N*-tetraose (DSLNT) **26**

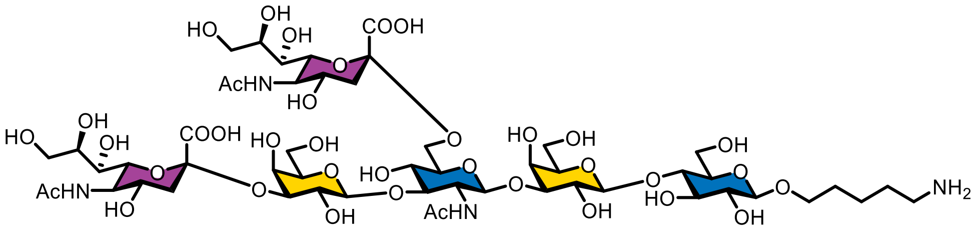

26

Unprotected hexasaccharide **26** (1.6 mg, 1.19 μmol, 31%) was obtained as a colorless oil after global deprotection (**Method C**), followed by purification using preparative RP-HPLC (**Method R4**).

^{1}H NMR (600 MHz, D_2O)**:** δ 4.72 (d, J = 8.5 Hz, 1H), 4.51 (d, J = 7.3 Hz, 1H), 4.49 (d, J = 8.1 Hz, 1H), 4.45 (d, J = 7.9 Hz, 1H), 4.18 (d, J = 3.2 Hz, 1H), 4.09 (dd, J = 9.9, 3.2 Hz, 1H), 4.01 – 3.93 (m, 4H), 3.93 – 3.83 (m, 7H), 3.82 – 3.75 (m, 6H), 3.75 – 3.65 (m, 10H), 3.65 – 3.58 (m, 8H), 3.55 (td, J = 9.0, 6.4 Hz, 2H), 3.31 (q, J = 8.0 Hz, 1H), 3.02 (t, J = 7.5 Hz, 2H), 2.77 (dd, J = 11.9, 4.5 Hz, 1H), 2.74 (dd, J = 12.1, 4.6 Hz, 1H), 2.04 (s, 3H), 2.04 (s, 3H), 2.03 (s, 3H), 1.79 (t, J = 12.1 Hz, 1H), 1.74 – 1.66 (m, 5H), 1.51 – 1.44 (m, 2H).

^{13}C NMR (151 MHz, D_2O)**:** δ 175.0, 175.0, 173.4, 168.6, 103.4, 102.9, 102.5, 102.0, 100.2, 99.6, 82.2, 81.8, 78.5, 77.0, 76.9, 75.6, 75.1, 75.0, 74.8, 74.5, 73.7, 72.8, 72.5, 71.8, 71.7, 70.0, 69.9, 69.1, 68.4, 68.4, 68.3, 68.2, 68.1, 68.1, 67.3, 62.9, 62.6, 62.5, 61.1, 61.0, 60.1, 54.6, 51.8, 51.6, 40.0, 39.8, 39.3, 28.1, 26.4, 22.3, 22.1, 22.0.

HRMS (QToF)**:** Calcd for $C_{53}H_{89}N_4O_{37}$ $[M - H]^-$ 1373.5206; found 1373.5205.

Crude analytical RP-HPLC (t_R = 29.2 min)

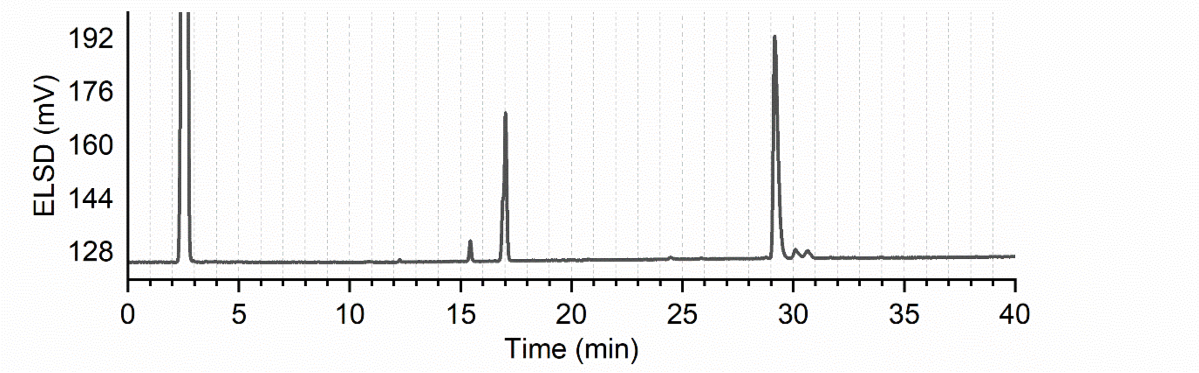

Purified analytical RP-HPLC

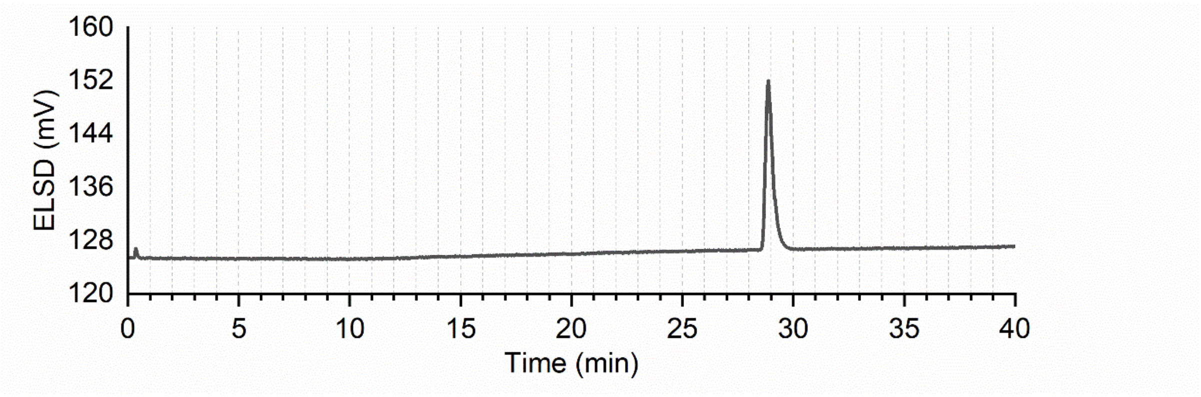

^{1}H-NMR (600 MHz, D_2O)

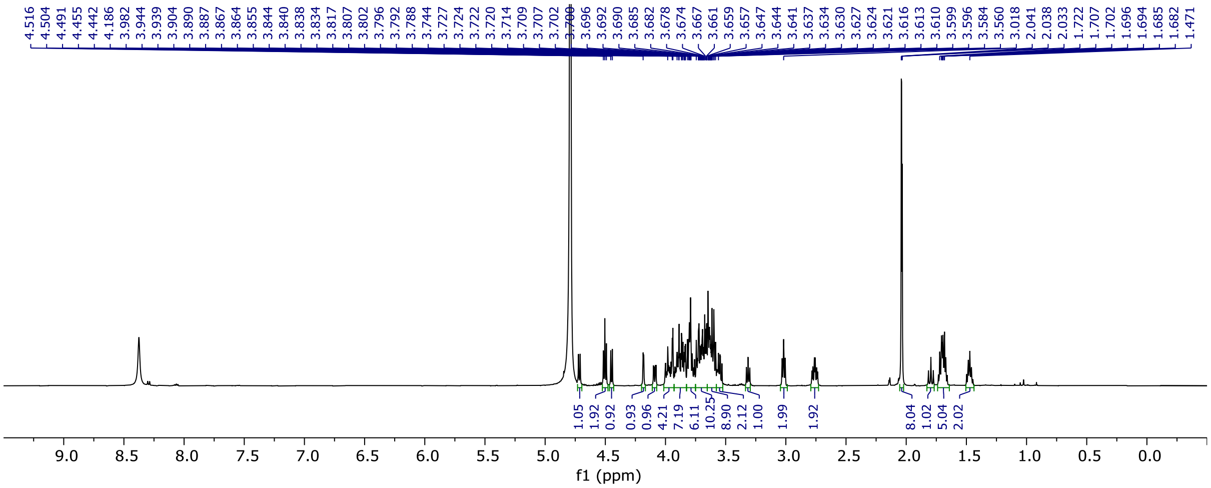

^{13}C-NMR (151 MHz, D_2O)

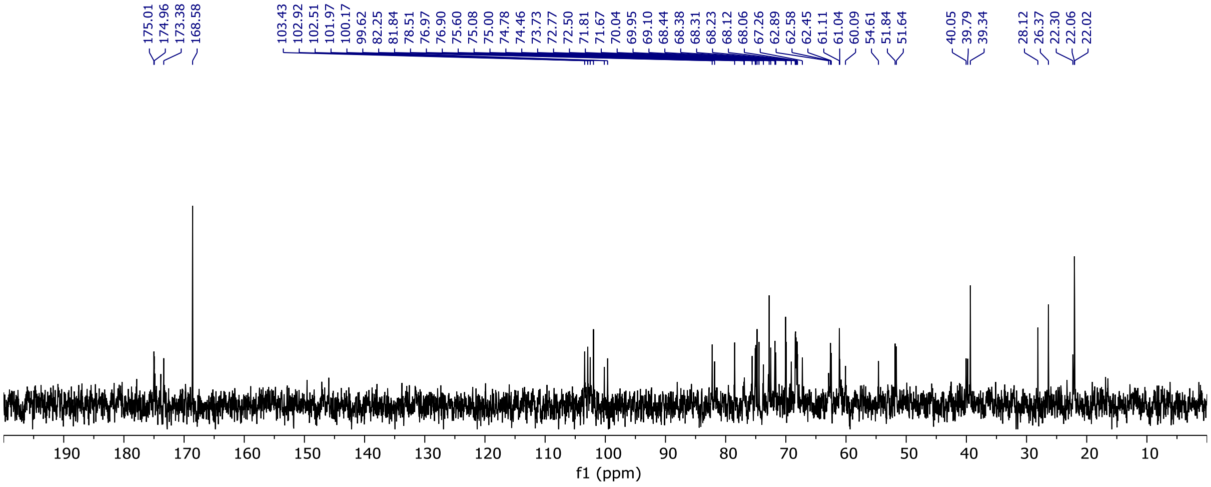

^{1}H -^{1}H COSY

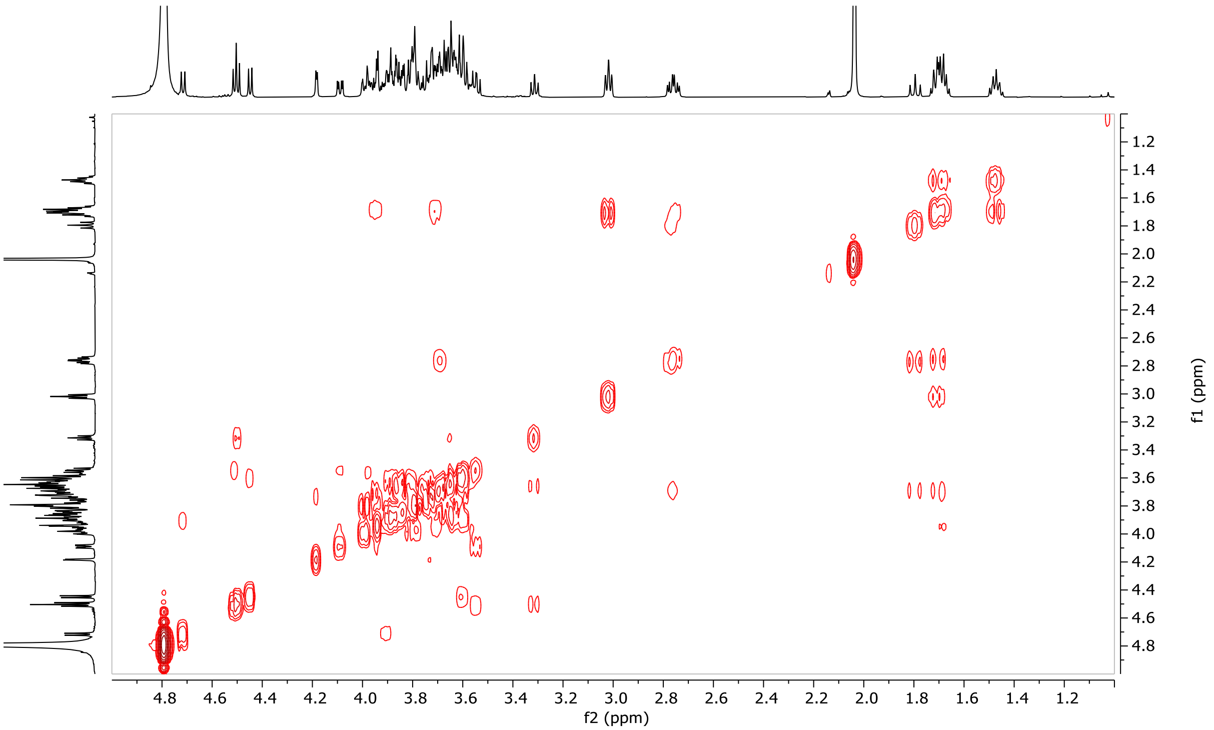

^{13}C-^{1}H HSQC

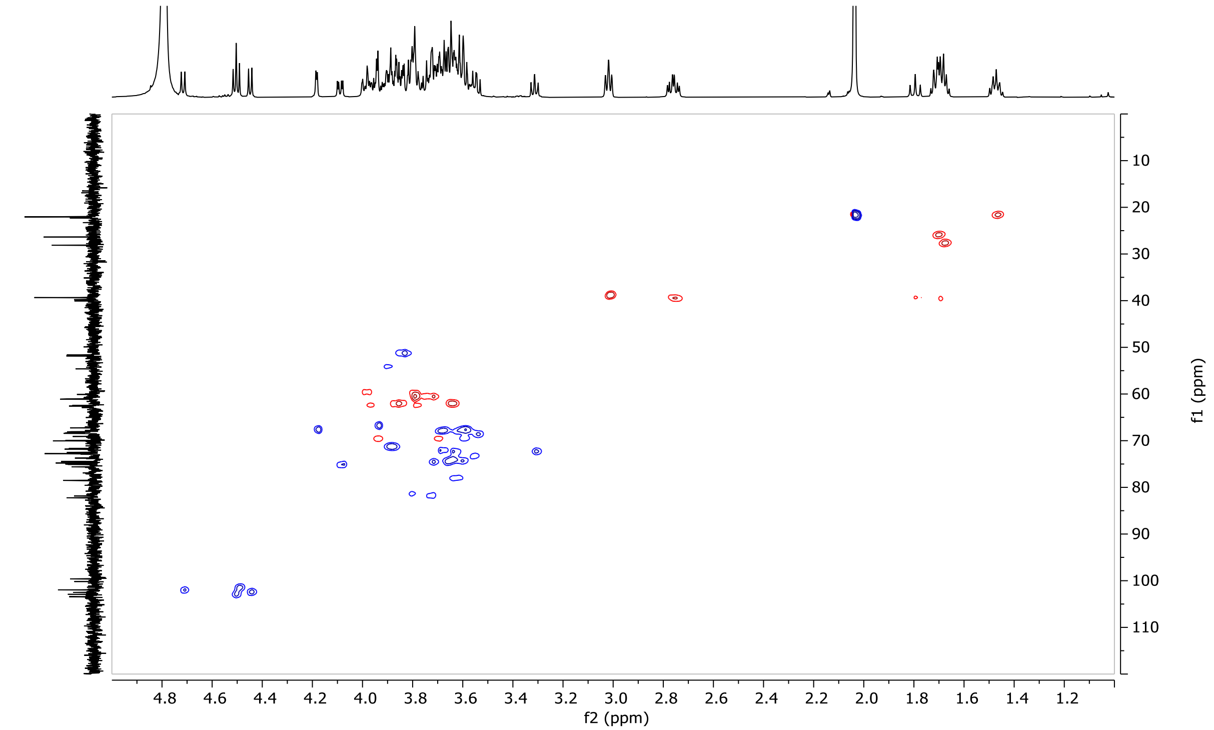

^{13}C-^{1}H HMBC

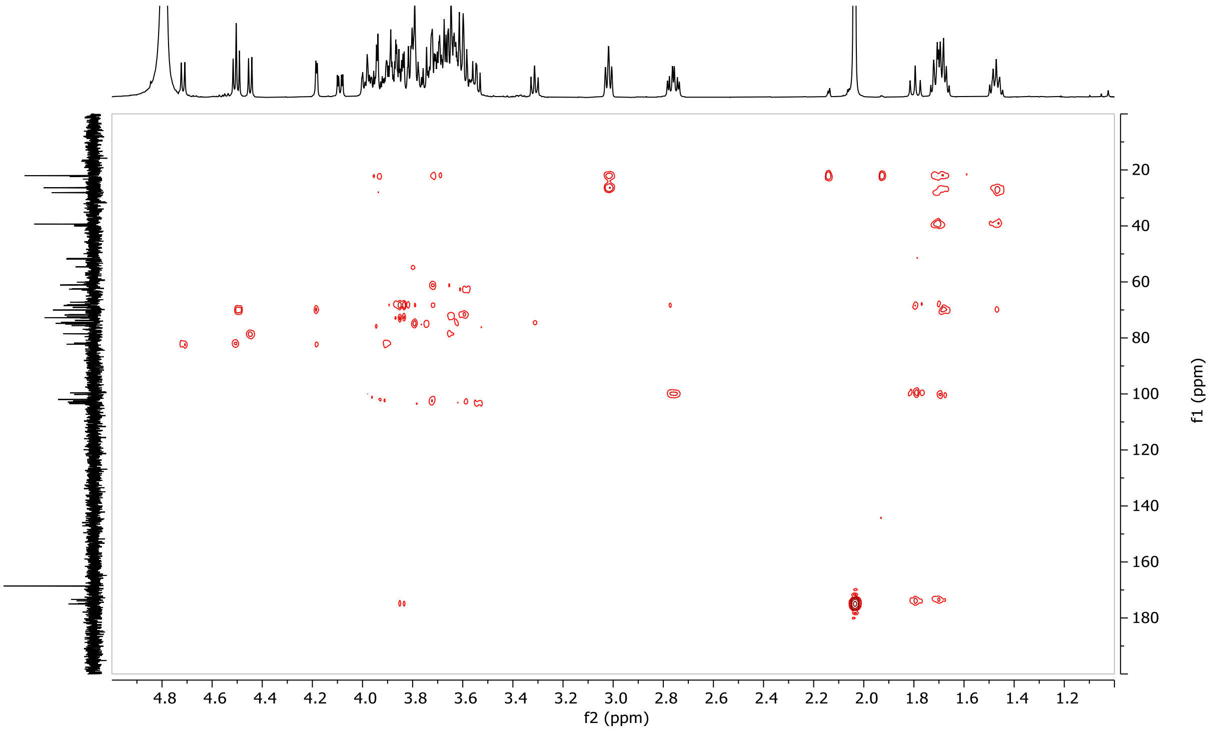

Expansion of ^{13}C-^{1}H HMBC

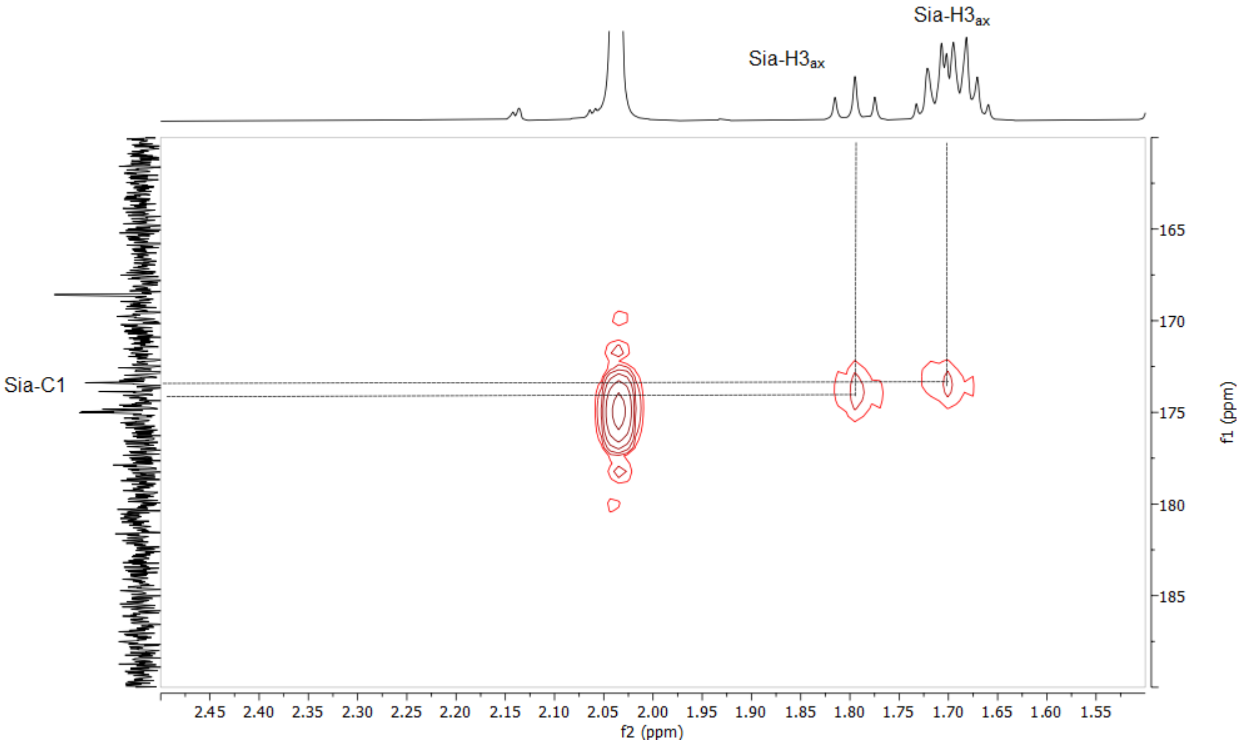

6.9 Sialyllacto-*N*-fucopentaose III (Sialyl LNFP III) **27**

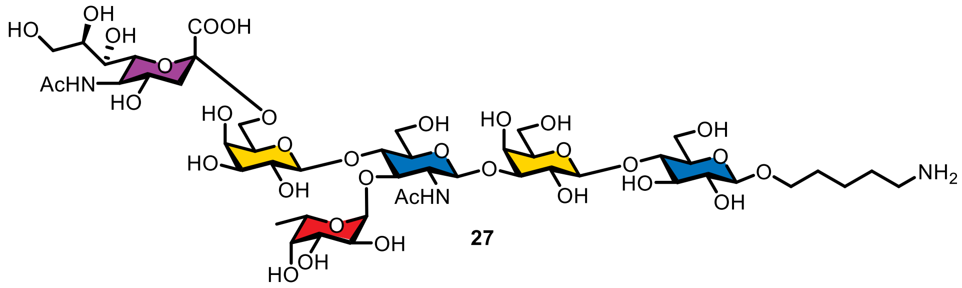

Unprotected hexasaccharide **27** (2.9 mg, 2.65 µmol, 56%) was obtained as a colorless oil after global deprotection (**Method C**), followed by purification using preparative RP-HPLC (**Method R1**).

^{1}H NMR (600 MHz, D_2O)**:** δ 5.11 (d, J = 4.0 Hz, 1H), 4.86 – 4.80 (m, 1H), 4.72 (d, J = 8.3 Hz, 1H), 4.50 (d, J = 8.0 Hz, 1H), 4.47 (d, J = 7.7 Hz, 1H), 4.44 (d, J = 7.5 Hz, 1H), 4.17 (d, J = 3.2 Hz, 1H), 4.01 – 3.92 (m, 6H), 3.92 – 3.85 (m, 6H), 3.84 (s, 1H), 3.83 – 3.75 (m, 4H), 3.75 – 3.63 (m, 11H), 3.63 – 3.57 (m, 5H), 3.50 (t, J = 8.8 Hz, 1H), 3.31 (t, J = 8.0 Hz, 1H), 3.02 (t, J = 7.3 Hz, 2H), 2.75 (dd, J = 12.7, 4.7 Hz, 1H), 2.05 (s, 3H), 2.03 (s, 3H), 1.75 – 1.64 (m, 5H), 1.51 – 1.43 (m, 2H), 1.18 (d, 3H).

^{13}C NMR (151 MHz, D_2O)**:** δ 175.1, 174.7, 173.5, 171.0, 103.0, 102.5, 102.0, 101.8, 100.7, 98.7, 82.1, 78.5, 75.2, 75.2, 74.9, 74.8, 74.5, 73.4, 72.8, 72.3, 72.1, 71.8, 71.0, 70.1, 70.0, 69.2, 68.3, 68.2, 67.8, 66.6, 62.9, 62.7, 61.0, 60.1, 59.7, 56.1, 51.9, 40.2, 39.4, 28.1, 26.4, 22.3, 22.1, 22.0, 15.4.

HRMS (QToF)**:** Calcd for $C_{42}H_{72}N_3O_{29}$ $[M - H]^-$ 1228.4831; found 1228.4785.

Crude analytical RP-HPLC (t_R = 27.0 min)

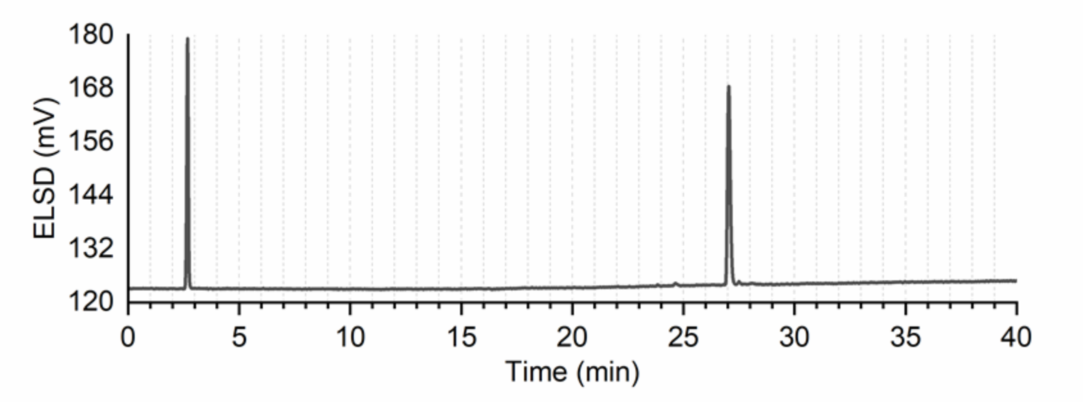

Purified analytical RP-HPLC

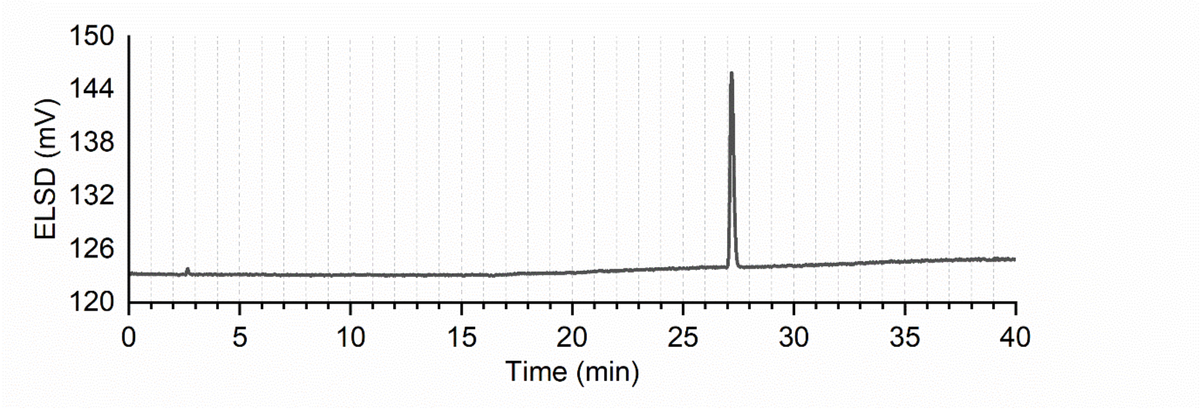

^{1}H-NMR (600 MHz, D_2O)

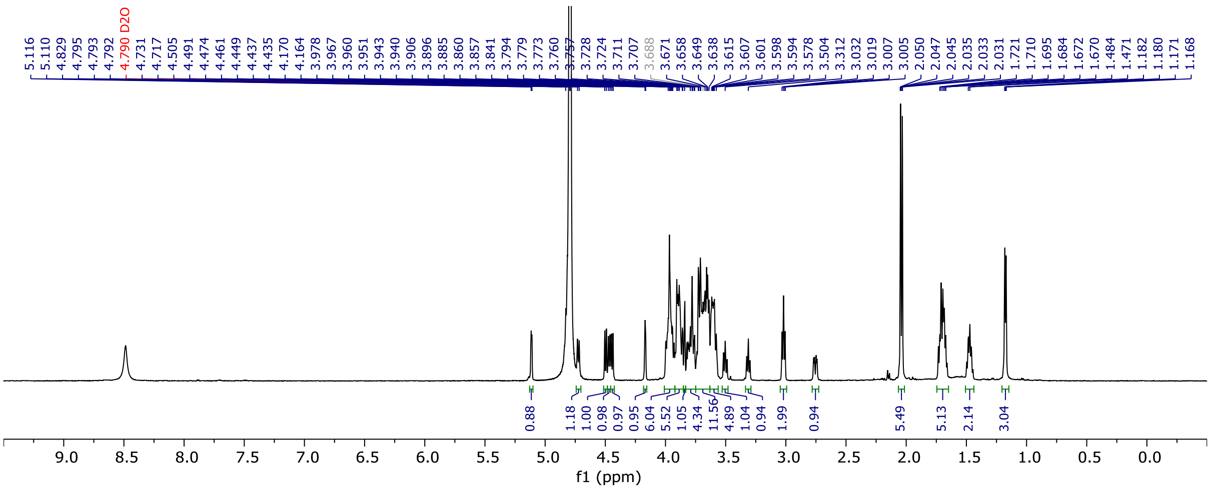

^{13}C-NMR (151 MHz, D_2O)

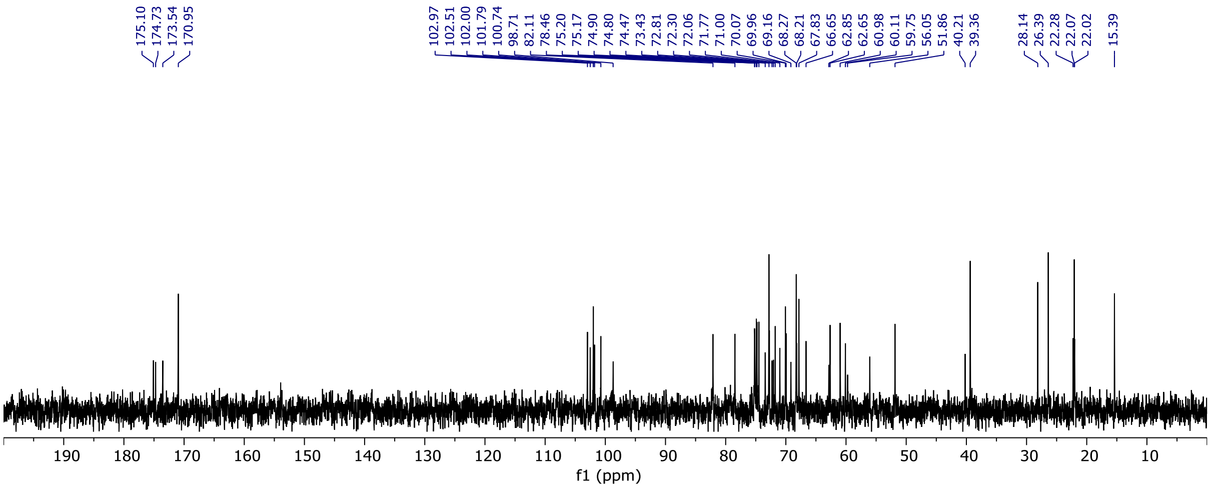

^{1}H -^{1}H COSY

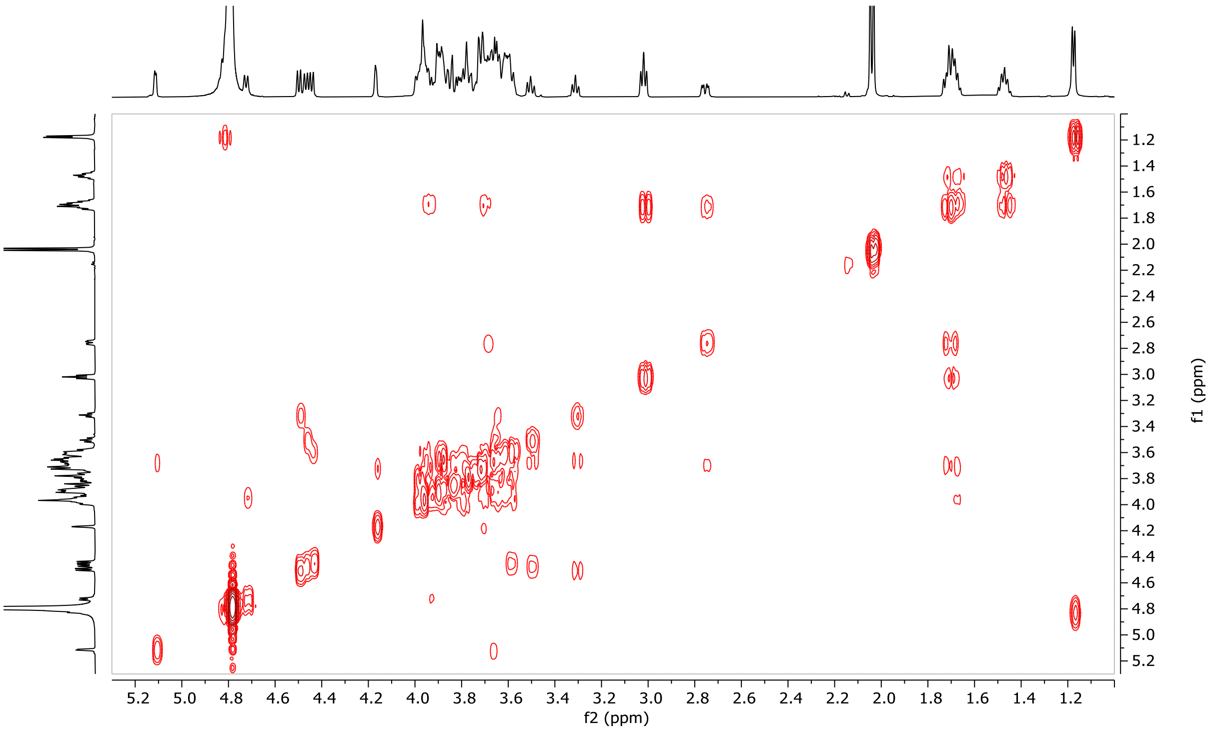

^{13}C-^{1}H HSQC

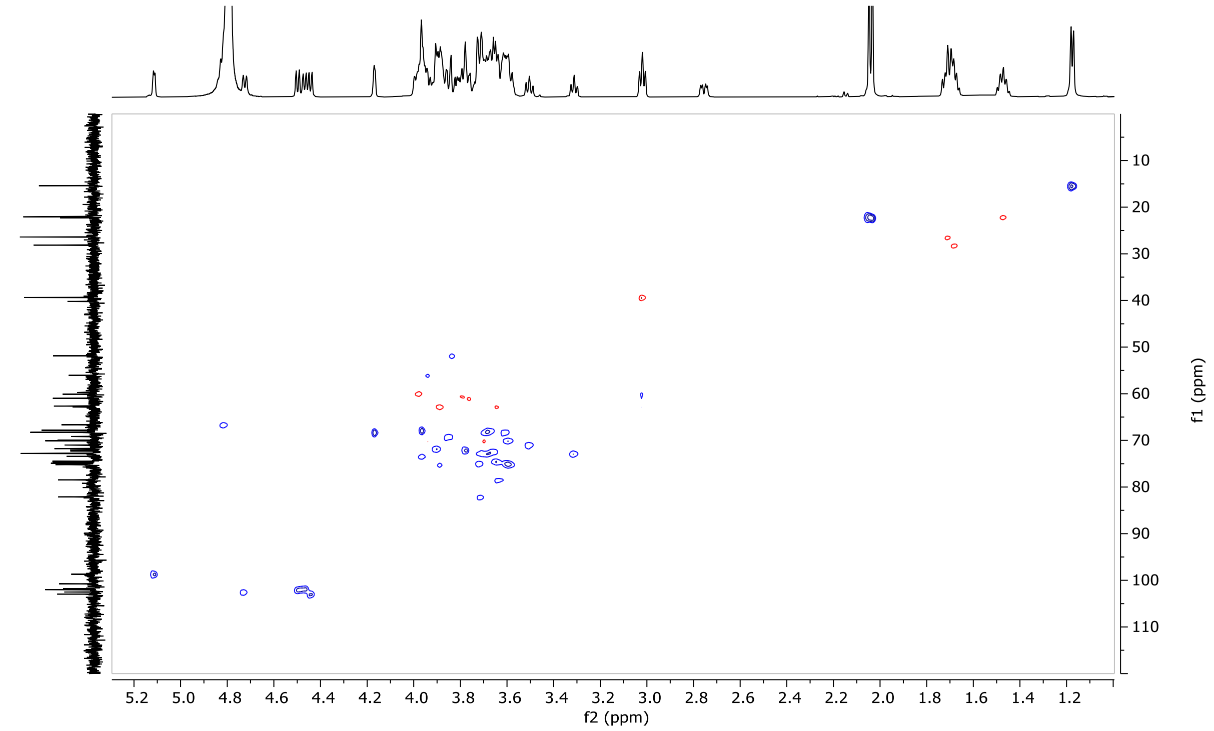

^{13}C-^{1}H HMBC

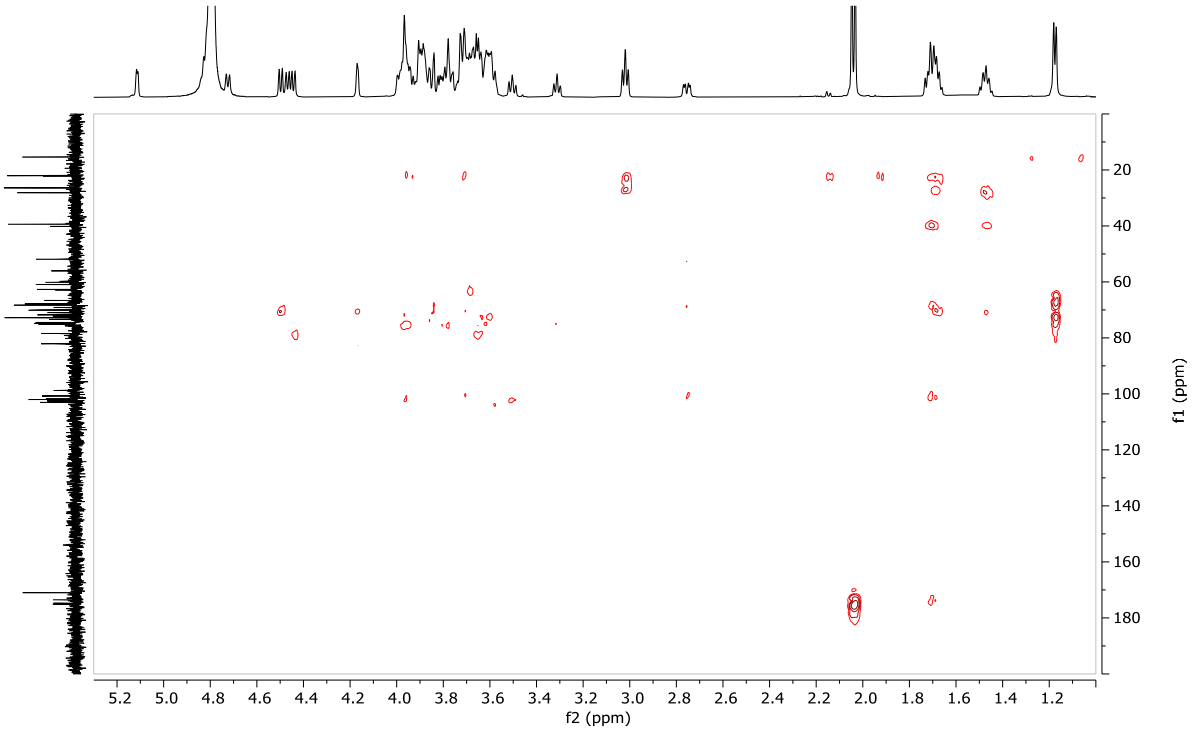

Expansion of ^{13}C-^{1}H HMBC

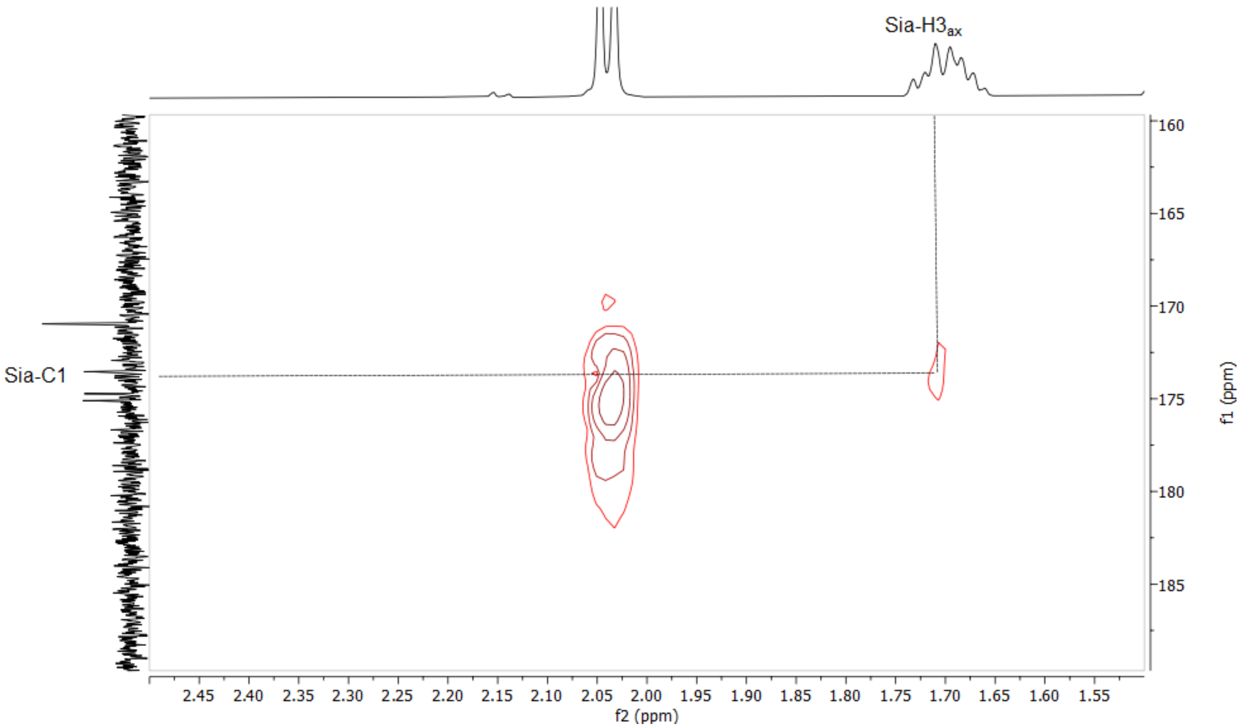

6.10 Sialyllacto-*N*-fucopentaose III (Sialyl LNFP III) **28**

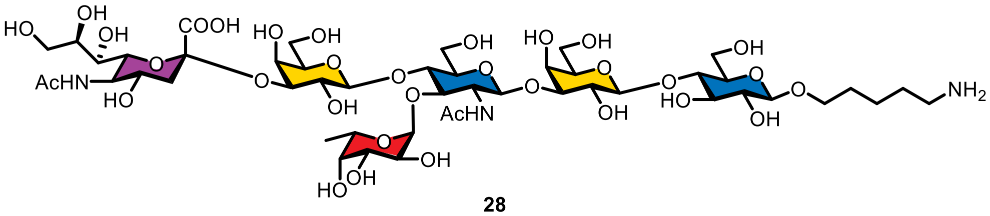

28

Unprotected hexasaccharide **28** (1.6 mg, 1.33 µmol, 36%) was obtained as a colorless oil after global deprotection (**Method C**), followed by purification using preparative RP-HPLC (**Method R3**).

^{1}H NMR (600 MHz, D_2O)**:** δ 5.13 (d, *J* = 4.0 Hz, 1H), 4.88 – 4.82 (m, 1H), 4.72 (d, *J* = 8.4 Hz, 1H), 4.54 (d, *J* = 7.8 Hz, 1H), 4.49 (d, *J* = 8.0 Hz, 1H), 4.44 (d, *J* = 7.9 Hz, 1H), 4.17 (d, *J* = 3.6 Hz, 1H), 4.09 (dd, *J* = 9.9, 3.2 Hz, 1H), 4.01 – 3.93 (m, 6H), 3.93 – 3.84 (m, 6H), 3.82 – 3.75 (m, 4H), 3.75 – 3.67 (m, 8H), 3.67 – 3.63 (m, 3H), 3.63 – 3.56 (m, 5H), 3.54 (dd, *J* = 9.8, 7.8 Hz, 1H), 3.31 (d, *J* = 8.8 Hz, 1H), 3.02 (t, *J* = 7.6 Hz, 2H), 2.77 (dd, *J* = 12.4, 4.6 Hz, 1H), 2.04 (s, 3H), 2.03 (s, 3H), 1.80 (t, *J* = 12.2 Hz, 1H), 1.75 – 1.64 (m, 4H), 1.51 – 1.43 (m, 2H), 1.18 (d, *J* = 6.6 Hz, 3H).

^{13}C NMR (151 MHz, D_2O)**:** δ 175.0, 174.7, 173.8, 169.1, 102.9, 102.6, 102.0, 101.6, 99.6, 98.6, 82.1, 78.4, 75.7, 75.0, 74.9, 74.9, 74.8, 74.6, 74.4, 73.0, 72.9, 72.8, 71.9, 71.8, 70.1, 70.0, 69.9, 69.3, 69.2, 68.3, 68.3, 68.1, 67.7, 67.3, 66.7, 62.6, 61.5, 61.0, 61.0, 60.1, 59.5, 55.9, 51.7, 39.8, 39.3, 28.1, 26.4, 26.4, 22.2, 22.1, 22.0, 15.3.

HRMS (QToF)**:** Calcd for $C_{48}H_{82}N_3O_{33}$ $[M - H]^-$ 1228.4831; found 1228.4795.

Crude analytical RP-HPLC (t_R = 30.6 min)

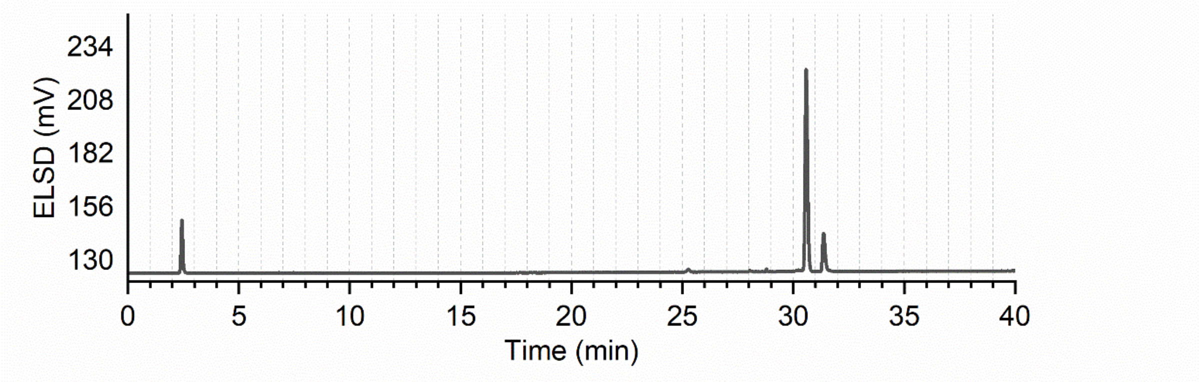

Purified analytical RP-HPLC

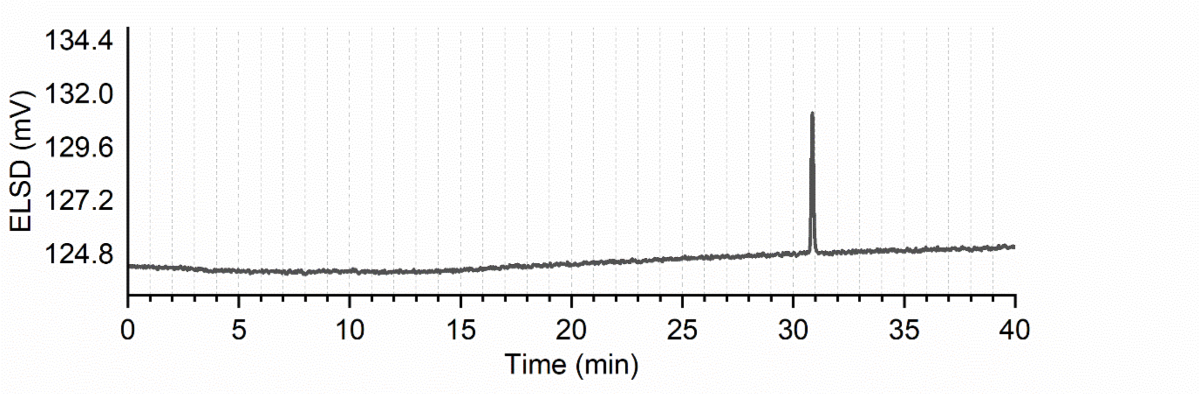

^{1}H-NMR (600 MHz, D_2O)

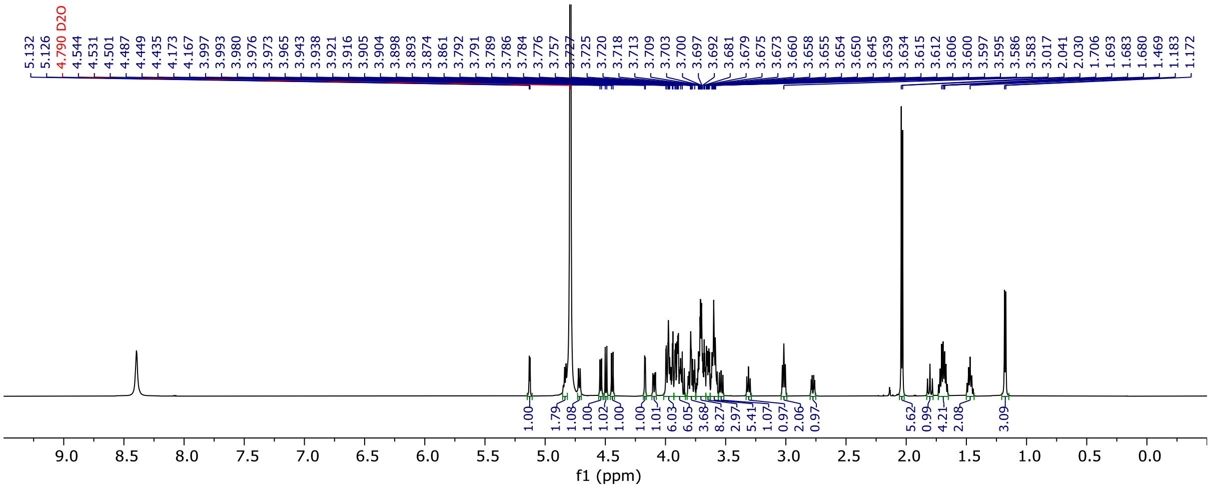

^{13}C-NMR (151 MHz, D_2O)

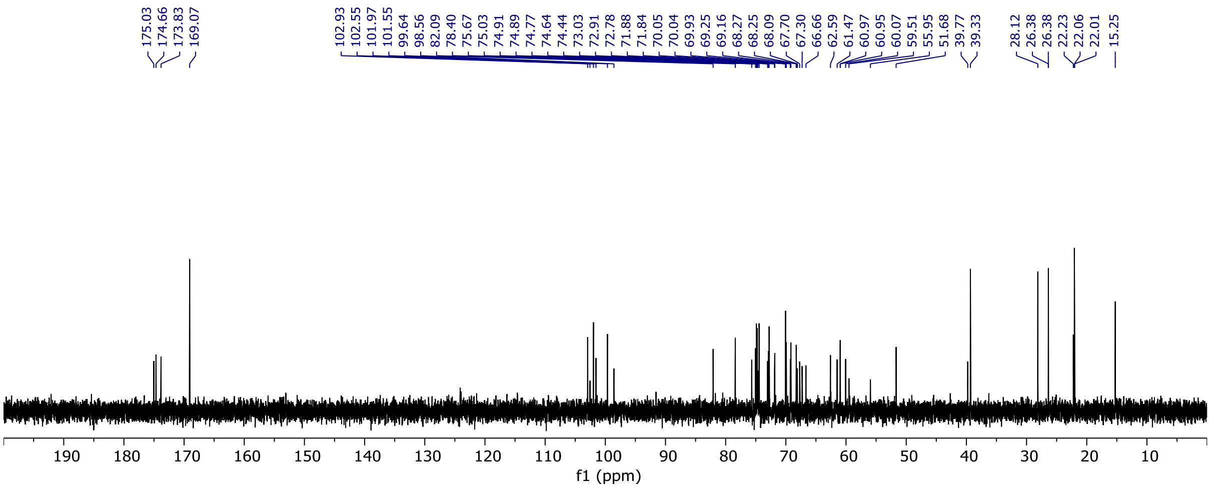

^{1}H -^{1}H COSY

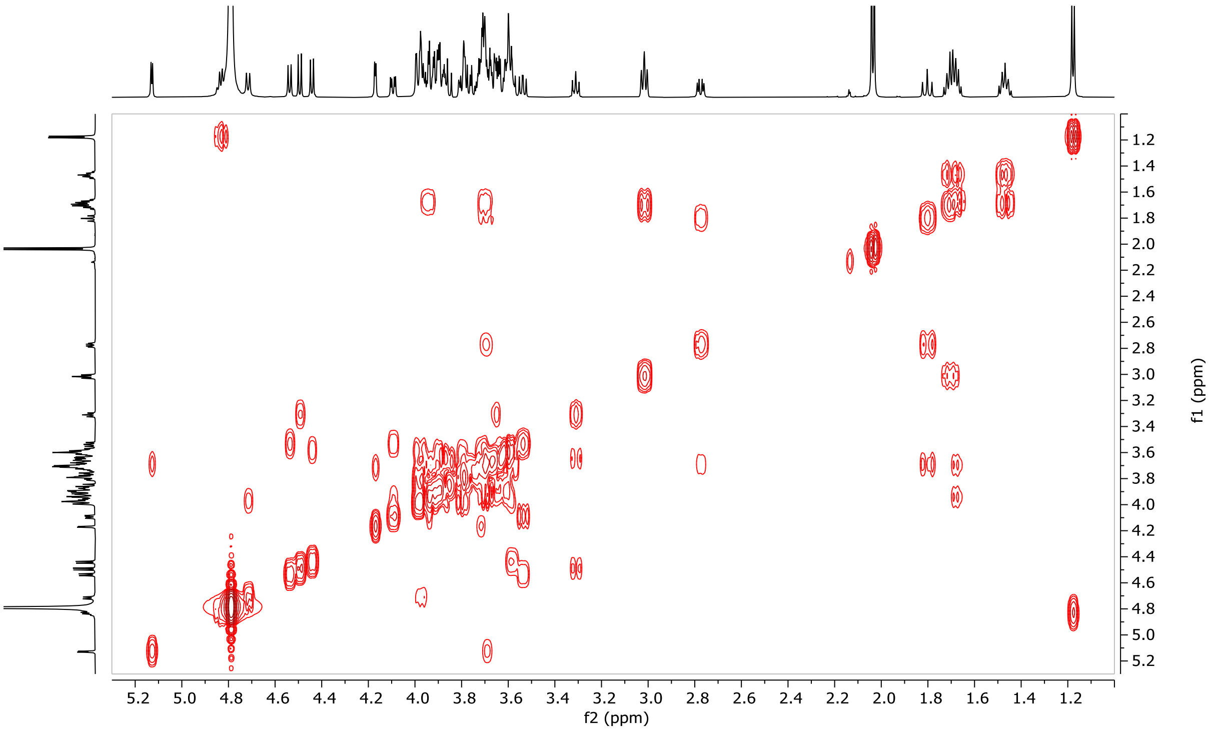

^{13}C-^{1}H HSQC

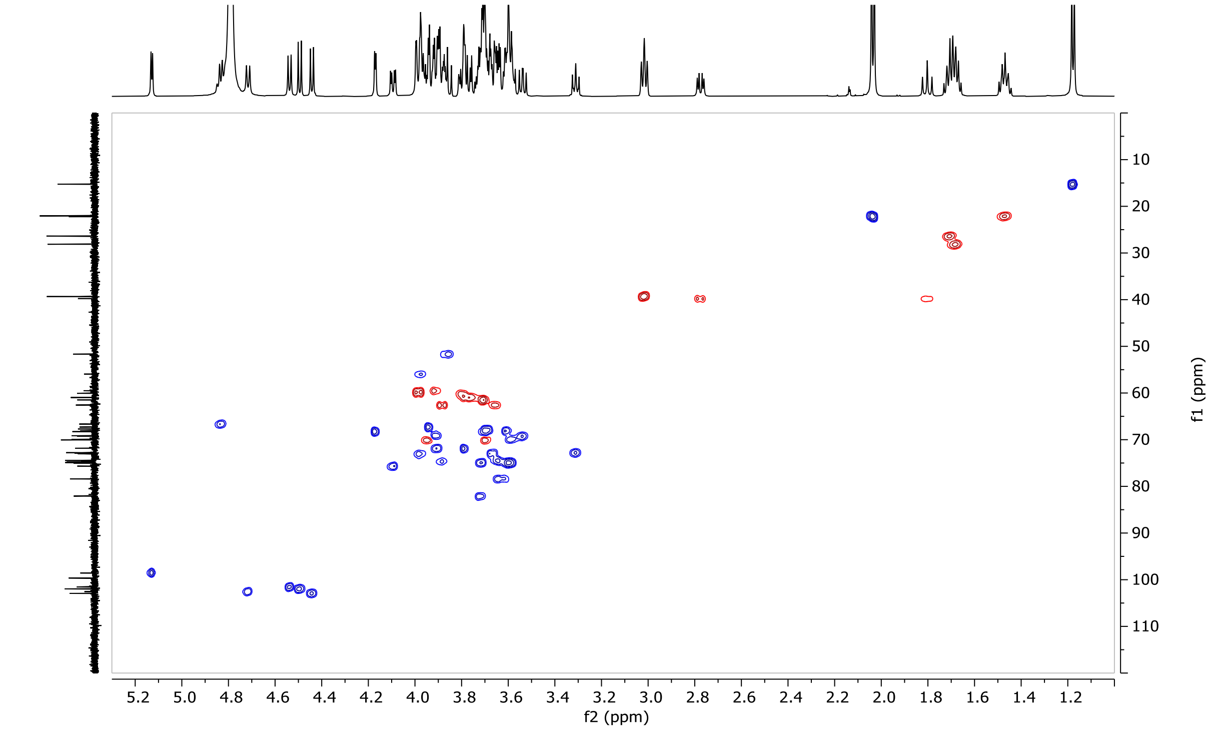

$^{13}C-^{1}H$ HMBC

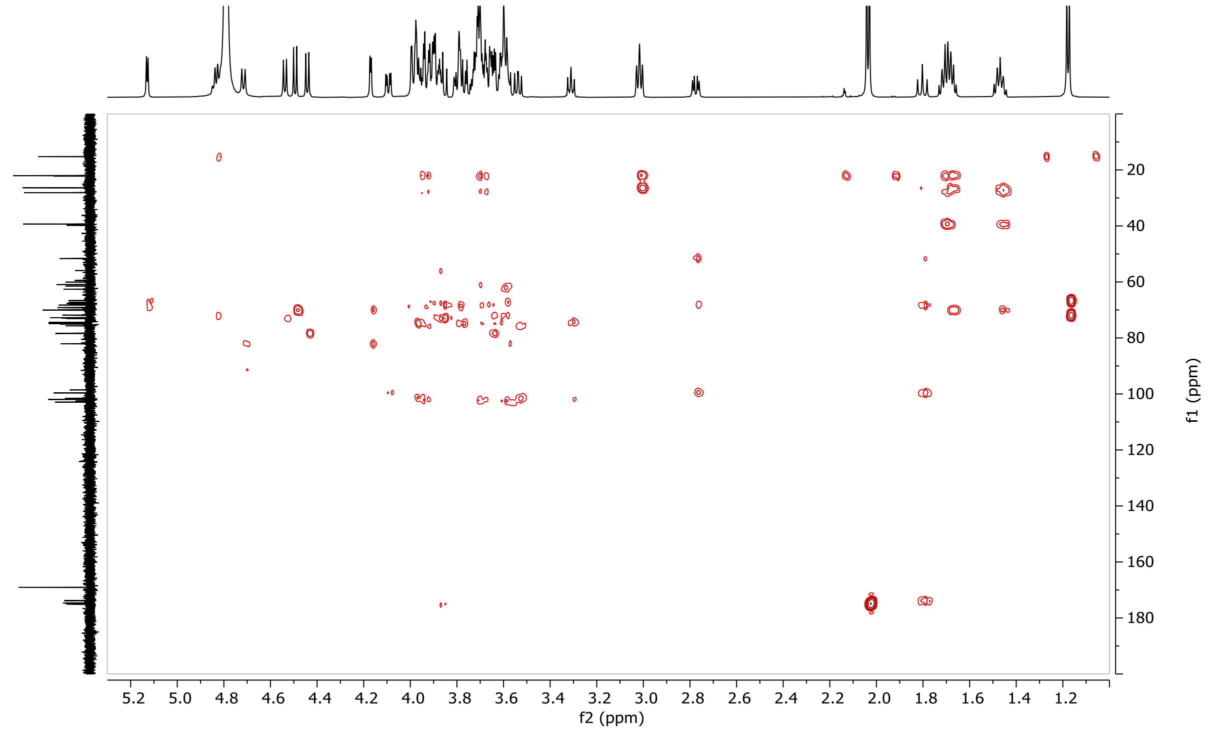

Expansion of $^{13}C-^{1}H$ HMBC

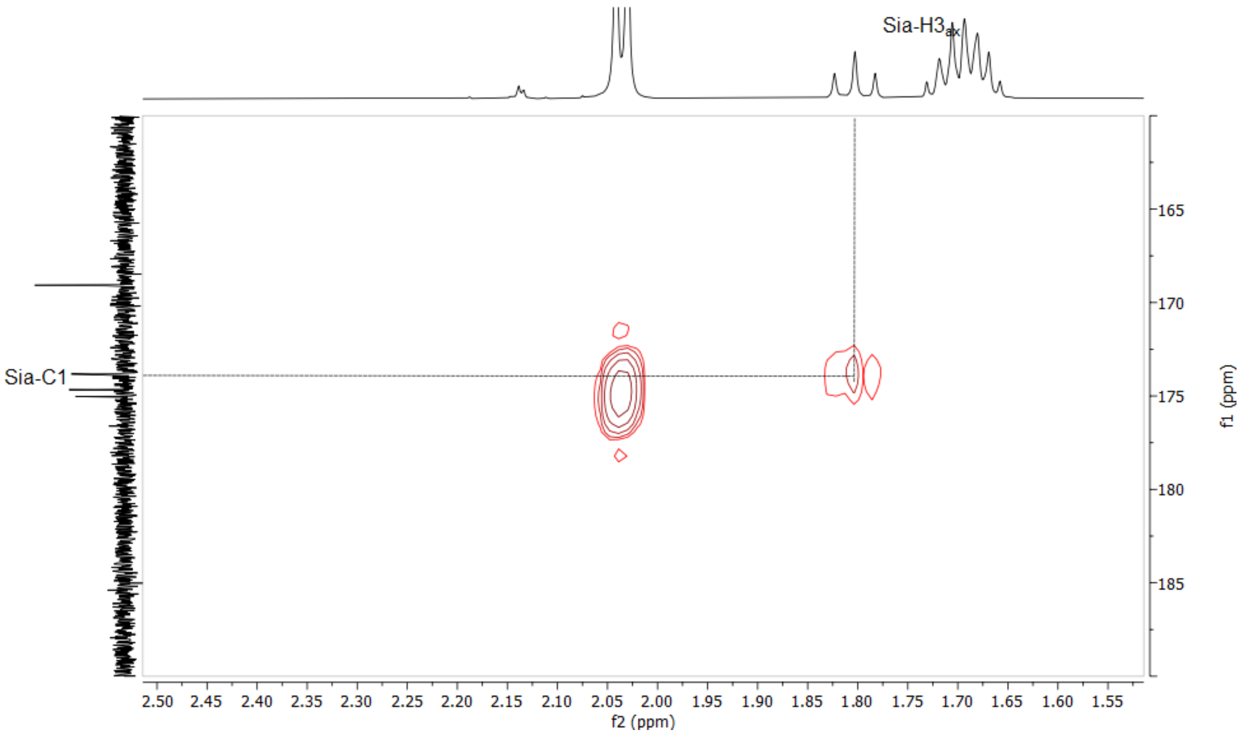

6.11 Fucosyldisialyllacto-N-tetraose (DSLNF II) **29**

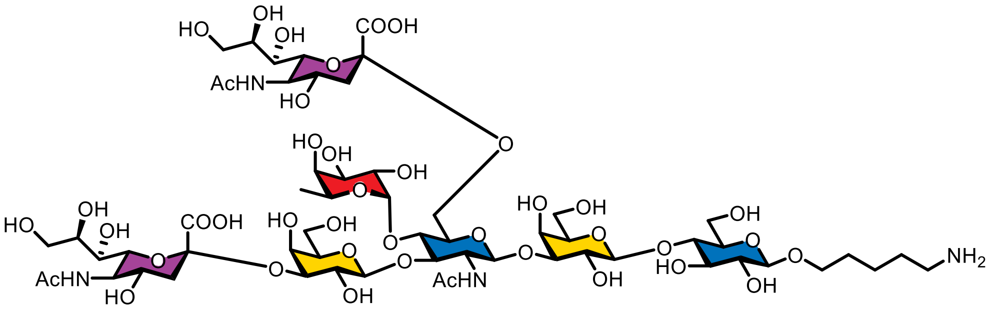

29

f

Unprotected hexasaccharide **29** (1.0 mg, 0.65 µmol, 23%) was obtained as a colorless oil after global deprotection (**Method C**), followed by purification using preparative RP-HPLC (**Method R5**).

^{1}H NMR (700 MHz, D_2O)**:** δ 5.15 (d, *J* = 3.9 Hz, 1H), 4.87 – 4.83 (m, 1H), 4.65 (d, *J* = 8.4 Hz, 1H), 4.50 (d, *J* = 7.7 Hz, 1H), 4.46 (d, *J* = 8.0 Hz, 1H), 4.39 (d, *J* = 7.8 Hz, 1H), 4.14 (d, *J* = 3.3 Hz, 1H), 4.07 – 3.99 (m, 3H), 3.97 – 3.84 (m, 5H), 3.85 – 3.71 (m, 13H), 3.69 – 3.53 (m, 18H), 3.51 – 3.46 (m, 2H), 3.27 (t, *J* = 8.5 Hz, 1H), 2.97 (t, *J* = 7.5 Hz, 2H), 2.75 (dd, *J* = 12.2, 4.8 Hz, 1H), 2.73 (dd, *J* = 12.3, 4.7 Hz, 1H), 2.00 (s, 6H), 1.99 (s, 3H), 1.74 (t, *J* = 12.2 Hz, 1H), 1.71 – 1.61 (m, 5H), 1.46 – 1.40 (m, 2H), 1.14 (d, *J* = 6.6 Hz, 3H).

^{13}C NMR (176 MHz, D_2O)**:** δ 175.0, 174.8, 174.5, 173.9, 173.3, 102.9, 102.8, 102.6, 101.9, 100.4, 99.3, 97.8, 89.3, 82.3, 78.4, 75.4, 74.9, 74.7, 74.7, 74.4, 73.6, 72.7, 72.6, 72.5, 72.0, 71.9, 71.8, 71.7, 70.0, 69.8, 69.1, 68.7, 68.4, 68.4, 68.1, 67.9, 67.6, 66.8, 66.7, 62.6, 62.2, 61.8, 61.6, 61.0, 60.0, 51.7, 51.6, 40.1, 39.9, 39.3, 28.1, 26.3, 22.3, 22.0, 21.9, 15.3.

HRMS (QToF)**:** Calcd for $C_{59}H_{99}N_4O_{41}$ $[M - H]^-$ 1519.5785; found 1519.5746

Crude analytical RP-HPLC (t_R = 15.1 min)

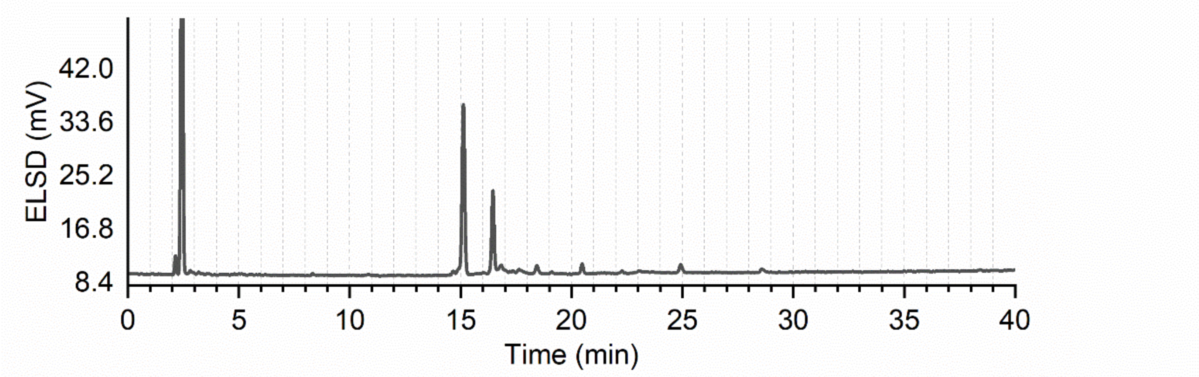

Purified analytical RP-HPLC

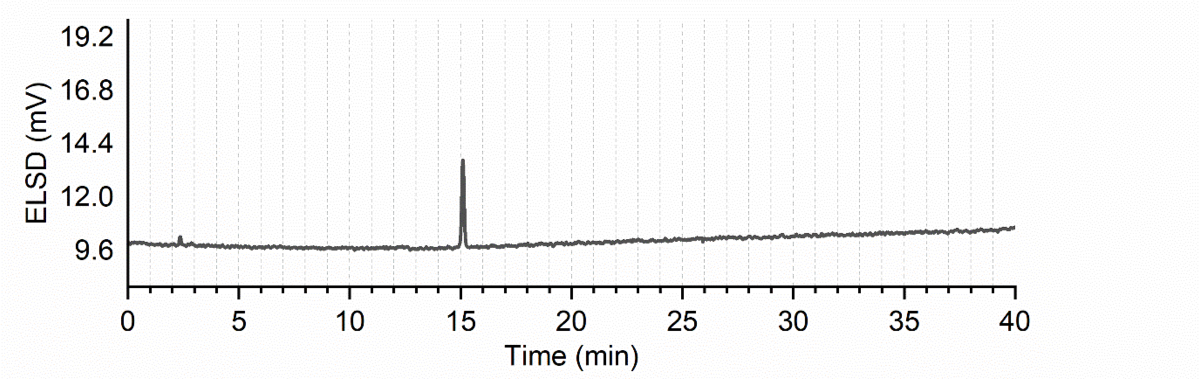

^{1}H-NMR (700 MHz, D_2O)

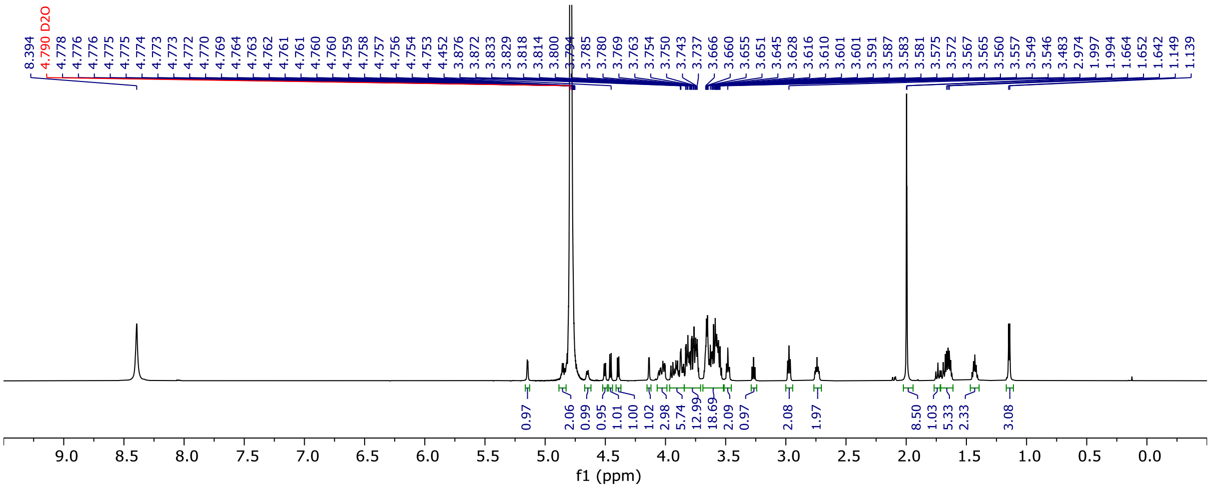

^{13}C-NMR (176 MHz, D_2O)

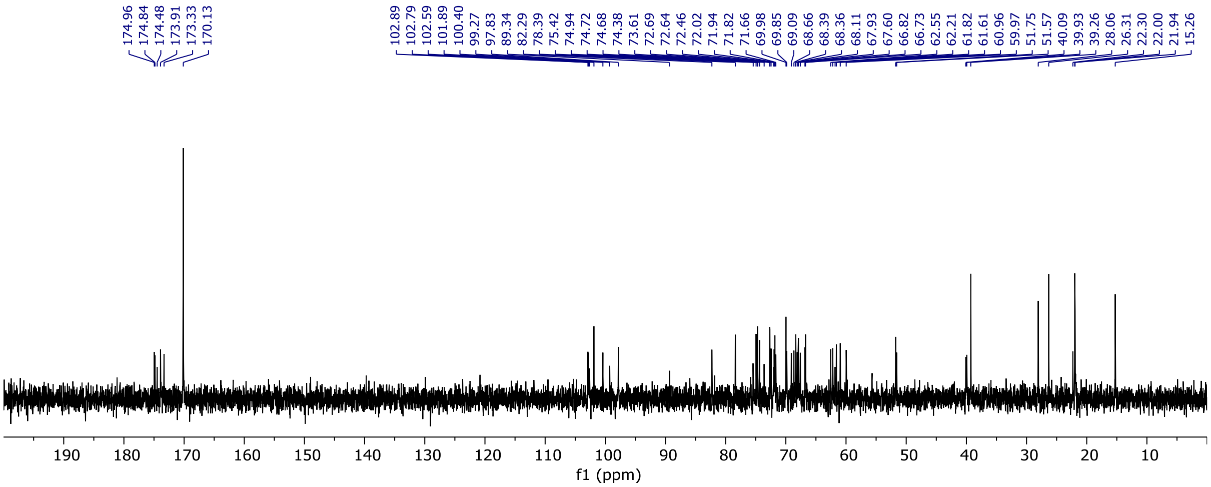

^{1}H -^{1}H COSY

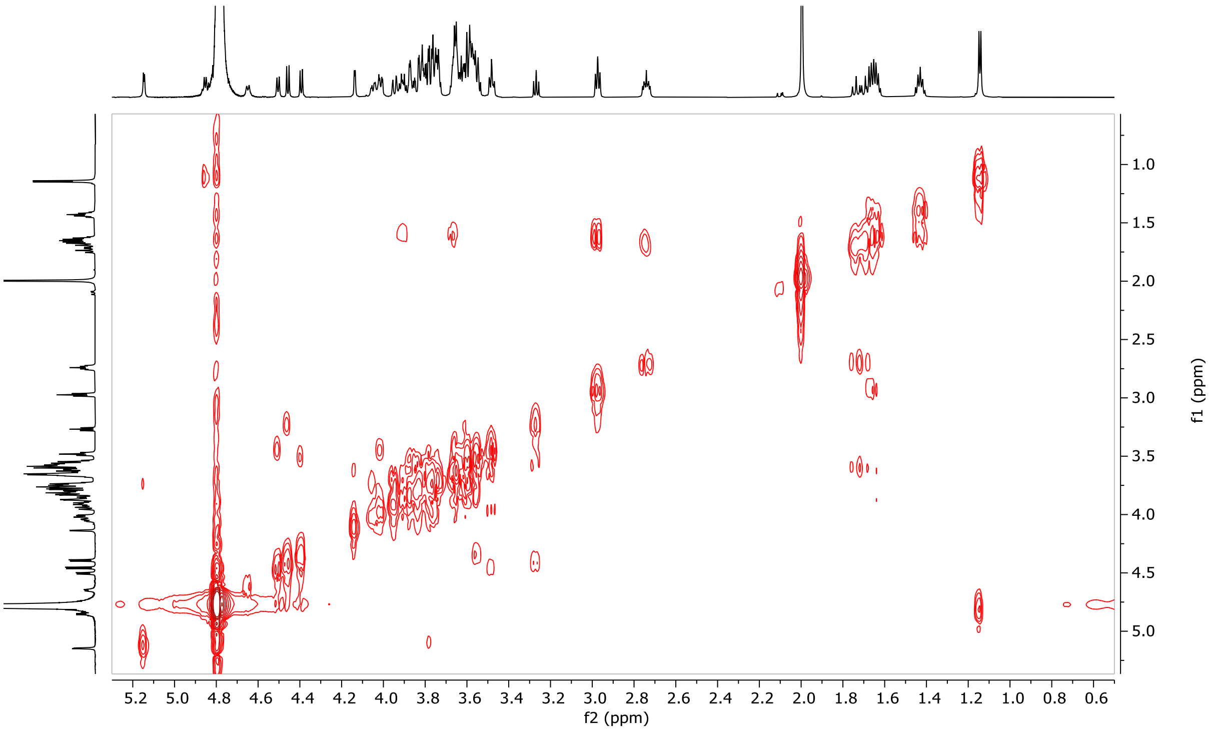

^{13}C-^{1}H HSQC

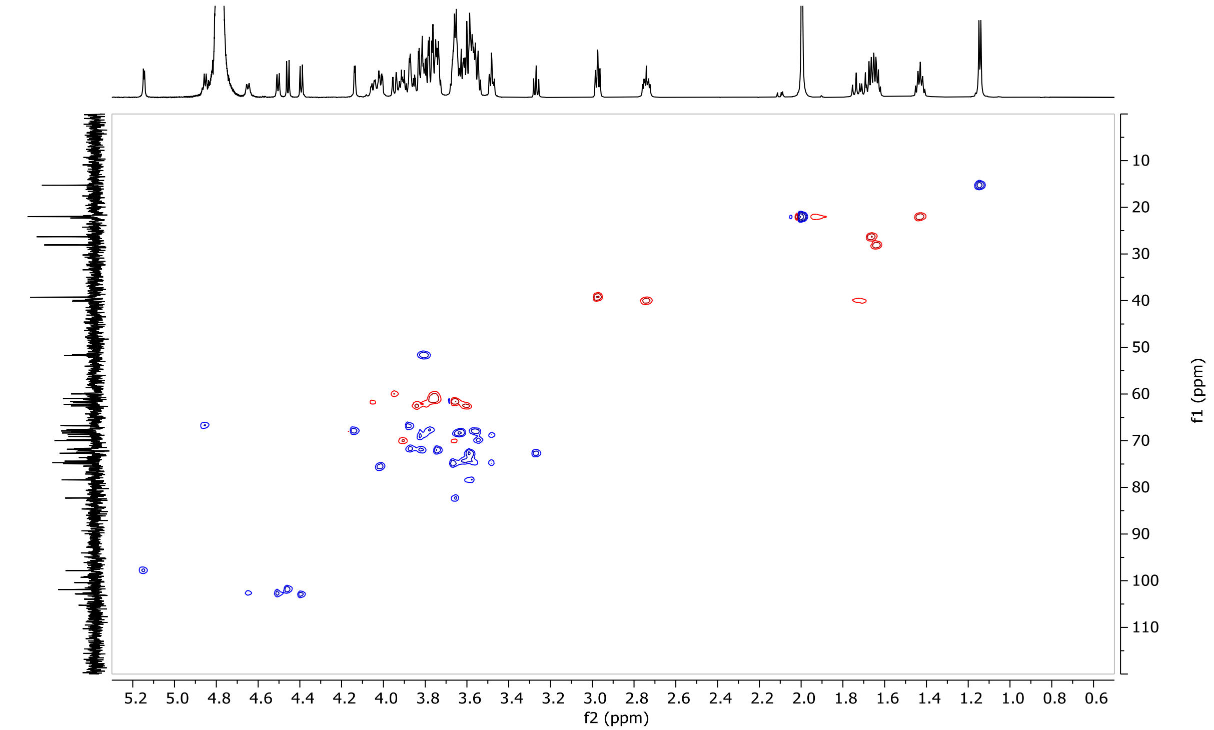

$^{13}C-^{1}H$ HMBC

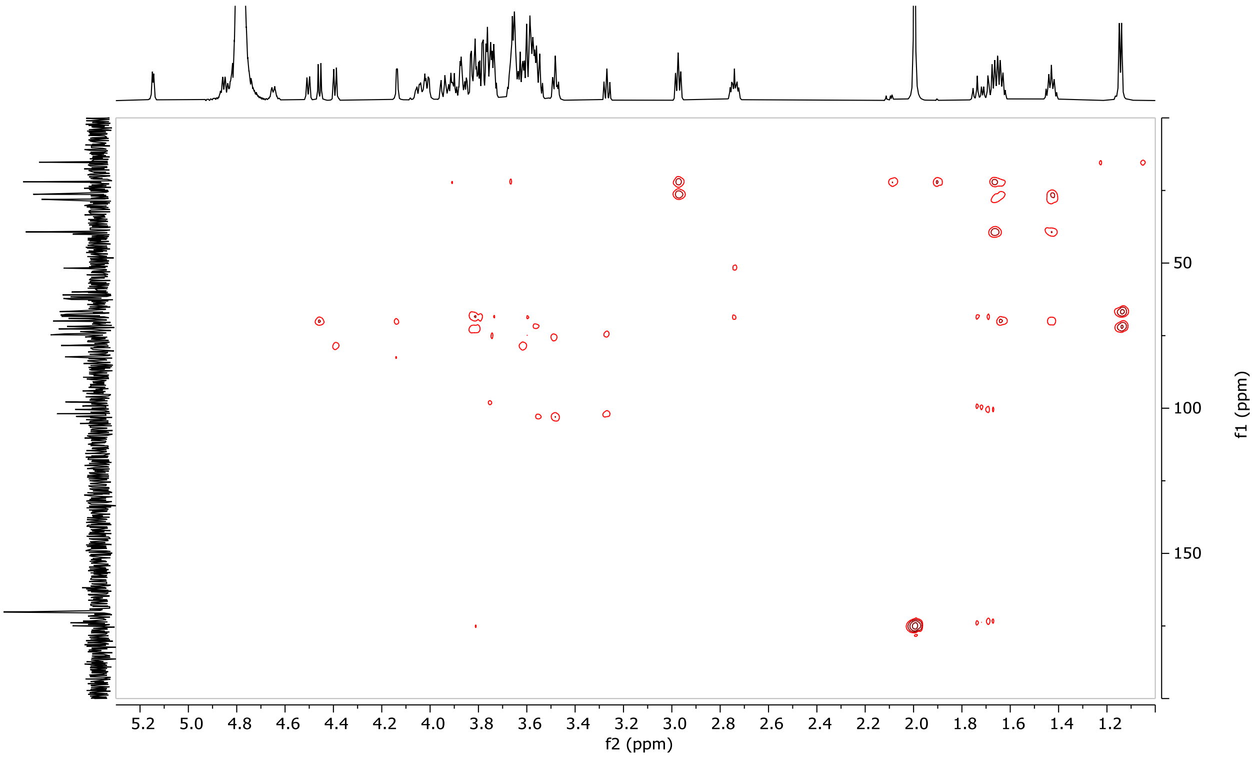

Expansion of $^{13}C-^{1}H$ HMBC

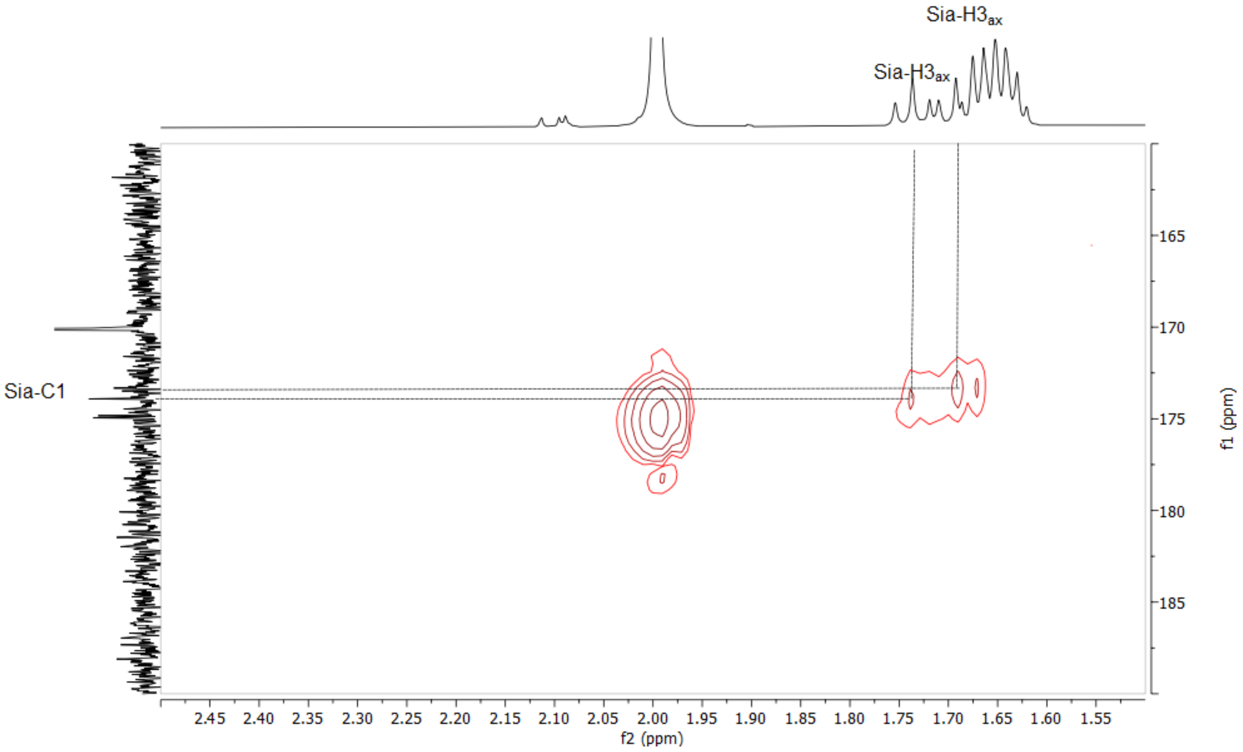

7 References

1. Komura, N. et al. Constrained sialic acid donors enable selective synthesis of α-glycosides. *Science* **364**, 677–680 (2019).
2. Ogura, H., Furuhata, K., Itoh, M. & Shitori, Y. Syntheses of 2-O-glycosyl derivatives of *N*-acetyl-D-neuraminic acid. *Carbohydr. Res.* **158**, 37–51 (1986).
3. Cao, S., Meunier, S. J., Andersson, F. O., Letellier, M. & Roy, R. Mild stereoselective syntheses of thioglycosides under PTC conditions and their use as active and latent glycosyl donors. *Tetrahedron: Asymmetry* **5**, 2303–2312 (1994).
4. Crich, D. & Li, W. α-Selective sialylations at −78 °C in nitrile solvents with a 1-adamantanyl thiosialoside. *J. Org. Chem.* **72**, 7794–7797 (2007).
5. Hassner, A., Strand, G., Rubinstein, M. & Patchornik, A. Levulinic esters. Alcohol protecting group applicable to some nucleosides. *J. Am. Chem. Soc.* **97**, 1614–1615 (1975).
6. Le Mai Hoang, K. et al. Traceless photolabile linker expedites the chemical synthesis of complex oligosaccharides by automated glycan assembly. *J. Am. Chem. Soc.* **141**, 9079–9086 (2019).
